# An Integrated ^12^C/^13^C Exchange Platform for Accessing Isotopically-Labeled Ketones via Dual Catalytic C–C Bond-Functionalization

**DOI:** 10.1021/acscatal.5c06571

**Published:** 2025-12-24

**Authors:** Hui-Qing Geng, Álvaro Velasco-Rubio, Eva Jaramillo-Cassà, Daniel Kócsi, Pablo García-Losada, Oscar de Frutos, Ruben Martin

**Affiliations:** † 202569 Institute of Chemical Research of Catalonia (ICIQ), The Barcelona Institute of Science and Technology, Av. Països Catalans 16, 43007 Tarragona, Spain; ‡ Universitat Rovira i Virgili, Departament de Química Orgànica, c/Marcel·lí Domingo, 1, 43007 Tarragona, Spain; § Centro de Investigación Lilly SA, Avda de la Industria 30, 28108 Alcobendas-Madrid, Spain; ∥ ICREA, Passeig Lluís Companys, 23, 08010 Barcelona, Spain

**Keywords:** C−C bond-cleavage, isotope-labeling, carbon isotope exchange, drug discovery, proaromatic precursors, Ni catalysis

## Abstract

Herein, we describe an integrated diacylation/acylation platform that formally converts simple ketones into their ^13^C-labeled homologues. The method relies on the propensity of proaromatic dihydroquinazolinones to generate open-shell species via C–C bond-functionalization aided by photoinduced single-electron transfer prior to interception with a properly labeled ^13^C-carbonyl derivative. The protocol is characterized by its broad applicability, thus offering a distinct gateway to access ^13^C-labeled ketones from their parent nonlabeled analogues.

## Graphical abstract

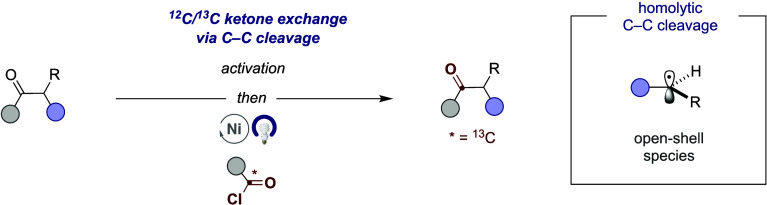

## Introduction

Isotope labeling is of utmost relevance in drug discovery, offering detailed insights into the behavior of lead candidates in biological systems.^1^ Carbon isotopes are the most common elements for obtaining preclinical data due to their high sensitivity and lower risk of metabolic cleavage.^2^ The low availability of ^14^C reagents makes ^14^C labeling prohibitively expensive, creating safety and handling concerns of carrying radioactivity in long sequences.^3^ Driven by the higher stability and availability of ^13^C probes, chemists have recently designed routes that incorporate ^13^C into organic molecules, facilitating the analysis of molecules using nonradioactive techniques such as NMR.^4^ While a ^12^C/^13^C exchange would obviate long-synthetic sequences and offer opportunities in late-stage derivatization, a close look into the literature data reveals a paucity of catalytic ^12^C/^13^C exchanges beyond carboxylic acids or activated carbonyls.^5^

Prompted by the prevalence of ketones in natural products and pharmaceuticals,^6^ a particularly attractive endeavor would consist of a formal catalytic carbonyl metathesis that converts ketones into their ^13^C-labeling analogues. At the outset of our investigations, however, it was unclear whether such technique could ever be implemented due to high bond-strength of C–C bonds adjacent to ketones and the presence of proximal, yet weaker, C­(sp^3^)–H bonds.^7^ If successful, however, such a scenario would not only expedite the access to advanced ^13^C-labeled intermediates without changing the established sequence for preparing the parent nonlabeled skeleton, but also offer new knowledge in the C–C bond-cleavage arena when implementing isotope-labeling endeavors.^7,8^ Our reaction design capitalizes on the propensity of proaromatic dihydroquinazolinones **I**, readily prepared by simple exposure of ketones to 2-aminobenzamides (2-AB), to generate open-shell species **II** via light-induced α C–C cleavage driven by aromaticity (Scheme 1).^9^ We anticipated that the site selectivity en route to **II** could be dictated and predicted by the innate stability of the resulting open-shell intermediate. Interception of **II** by an acyl-Ni­(II) oxidative addition species^10^ generated by reaction of Ni(0)­L_n_ with ^13^C-labeled acid chlorides followed by reductive elimination would result in a formal ^12^C/^13^C exchange. As part of our interest in designing C–C bond-functionalization reactions,^11^ we report herein the successful realization of this goal.

**Scheme 1:**
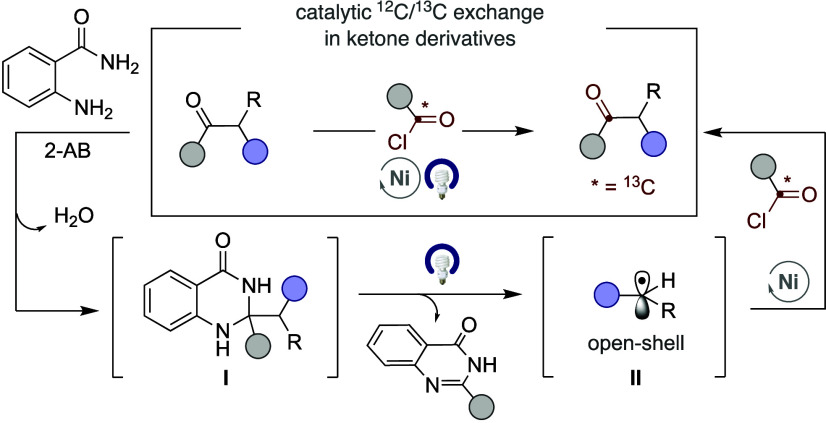
Formal ^12^C/^13^C Exchange in Ketone Derivatives

## Results and Discussion

We began our investigations by evaluating the acylation of **2a**, easily accessed in one step by exposure of 2-aminobenzamide to 1-cycloheptylethan-1-one **1a**, with ^13^C-acetyl chloride. The latter was chosen for our initial studies given its availability among a variety of chemical suppliers. After considerable experimentation,^12^ the best results were found with NiCl_2_·dme, **L1**, Ir­(dFCF_3_ppy)_2_(dtbbpy)­PF_6_ (**PC1**) as photocatalyst in dioxane under blue-LEDs irradiation (456 nm) at room temperature (rt), obtaining 71% isolated yield of **[** ^**13**^ **C]­1a** with >99% ^13^C content (Table 1, entry 1). As expected, the nature of the ligand was particularly important for the reaction to occur. In particular, the inclusion of substituents other than nonyl groups at C4 in 2,2′-bipyridine backbones had a deleterious effect on reactivity (entries 2–4). Similarly, lower yields of **[** ^**13**^ **C]­1a** were systematically found when utilizing C6-substituted **L4**, pyridine-oxazoline ligands of type **L5** (entries 4 and 5) or utilizing solvents other than 1,4-dioxane (entries 9 and 10). As initially anticipated, rigorous control experiments revealed no conversion of **2a** to **[** ^**13**^ **C]­1a** in the absence of either a nickel precatalyst or a photocatalyst (entries 11 and 12).

**Table 1:**
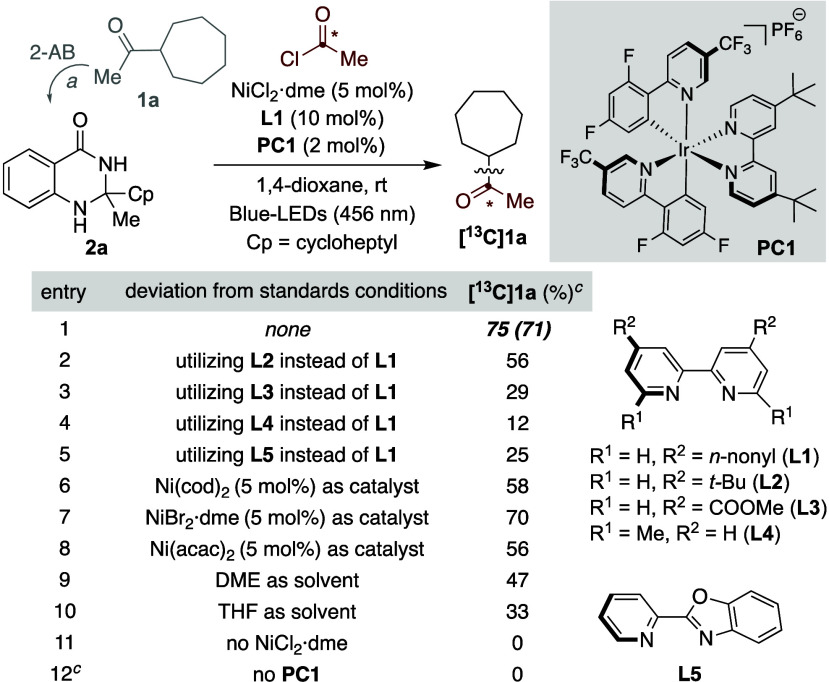
Optimization of the Reaction Conditions^b^

a **1a** (5 mmol), 2-aminobenzamide (2-AB, 5.25 mmol), I_2_ (5 mol %) in DMF (0.50 M), 80 °C, 18h.

b Conditions: **2a** (0.10 mmol), ^13^C-acetyl chloride (0.15 mmol), NiCl_2_·dme (5 mol %), **L1** (10 mol %), Ir­(dF­(CF_3_)­ppy)_2_(dtbpy)­PF_6_ (**PC1**, 2 mol %), 1,4-dioxane (0.10 M), 456 nm Kessil LEDs, rt for 24 h.

c Yields determined by GC with tetradecane as internal standard.

Encouraged by these results, we next explored the generality of our formal ^12^C/^13^C exchange in ketone derivatives via the intermediacy of proaromatic dihydroquinazolinones with commercially available ^13^C-acetyl chloride (Table 2). In line with our expectations, site-selectivity in the targeted α C–C bond-cleavage can be easily predicted by an appropriate selection of the substituents on dihydroquinazolinone and the relative stability of the resulting radicals arising from C–C functionalization. Thus, ketone derivatives possessing methyl and secondary alkyl substituents resulted in the coupling of the latter, affording **[** ^**13**^ **C]­1a-[** ^**13**^ **C]­1l** in good yields and >99% ^13^C content. Interestingly, similar results were accomplished independently of whether cyclic (**[** ^**13**^ **C]­1a-[** ^**13**^ **C]­1h**) or acyclic secondary alkyl radical species (**[** ^**13**^ **C]­1i-[** ^**13**^ **C]­1l**) were generated from the parent ketone derivative. Equally interesting was the observation that the coupling of primary alkyl radicals could also be within reach in the presence of adjacent heteroatoms (**[** ^**13**^ **C]­1m-[** ^**13**^ **C]­1w**) or arene backbones (**[** ^**13**^ **C]­1x**, **[** ^**13**^ **C]­1y**). It is worth noting, however, that the coupling of the latter required the utilization of di­(pyridin-2-yl)­methanone as a ligand. Notably, the protocol could be extended beyond ^13^C-acetyl chloride, as **[13C]­1z** and **[13C]­1za** were obtained in good yields from the corresponding ^13^C-labeled acyl chloride derivatives. In addition, the protocol could perfectly accommodate substrates bearing nitrogen-containing heterocycles (**[** ^**13**^ **C]­1h**, **[** ^**13**^ **C]­1i**, **[** ^**13**^ **C]­1u**), carbamates (**[** ^**13**^ **C]­1d**, **[** ^**13**^ **C]­1f**) or nitriles (**[** ^**13**^ **C]­1p**). Notably, the ^12^C/^13^C-exchange could be conducted in the presence of aryl halides (**[** ^**13**^ **C]­1c**, **[** ^**13**^ **C]­1q**, **[** ^**13**^ **C]­1r**, **[** ^**13**^ **C]­1y**) or aryl boronic esters (**[** ^**13**^ **C]­1o**), thus leaving ample room for additional functionalization via conventional metal-catalyzed cross-coupling reactions.^13^ Notably, the protocol could be applied to advanced synthetic intermediates derived from galactopyranose (**[** ^**13**^ **C]­1zb**), indomethacin (**[** ^**13**^ **C]­1zc**), isoxepac (**[** ^**13**^ **C]­1zd**), or silvex (**[** ^**13**^ **C]­1ze**).

**Table 2:**
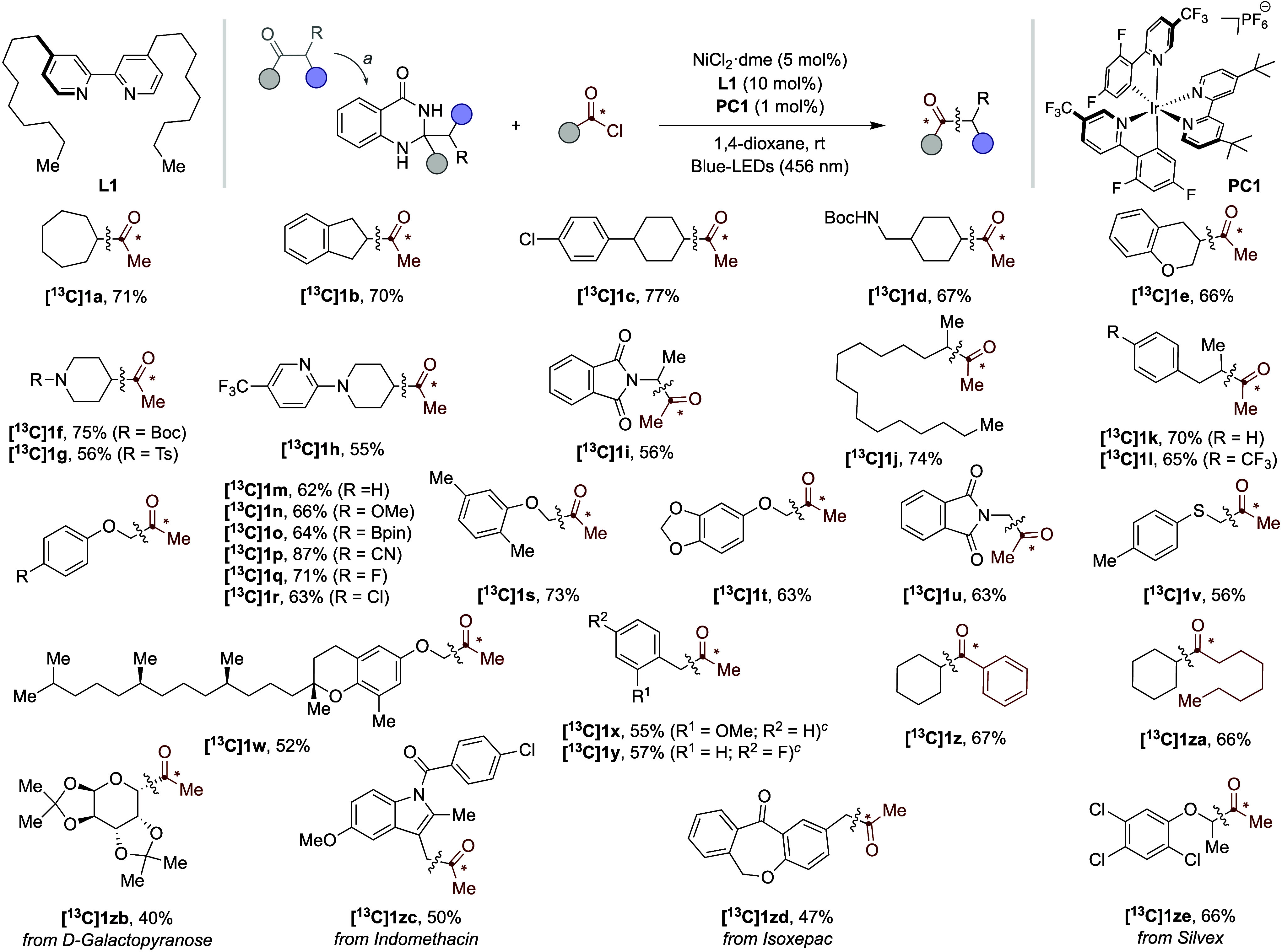
Generality of the Formal ^12^C/^13^C-Exchange in Ketone Derivatives^b^

a Dihydroquinazolinones were obtained by exposure of the parent ketone (1 equiv) to 2-aminobenzamide (1.05 equiv), I_2_ (5 mol %) in DMF (0.50 M) at 80 °C for 18 h.

b As Table 1 (entry 1), yields of isolated compounds, average of two independent runs. R^1^ = Me.

c Di­(pyridin-2-yl)­methanone (10 mol %) was used as ligand instead of **L1**.

Aiming at showing the inherent potential of the ^12^C/^13^C-exchange of ketone derivatives, we wondered whether we could telescope the formation of the intermediate dihydroquinazolinone from their parent ketones. As shown in Scheme 2, this turned out to be the case, and the targeted ^13^C-labeled **[** ^**13**^ **C]­1g** and **[** ^**13**^ **C]­1m** could be obtained in one-pot from their parent ketones in synthetically useful yields. Although in slightly lower yields to those shown in Table 2, these results should be assessed against the challenge that is addressed, providing an unprecedented opportunity in medicinal chemistry settings for converting an alkyl ketone into its ^13^C-labeled homologue in a one-pot manner, and without requiring isolation of the corresponding intermediates.

**Scheme 2:**
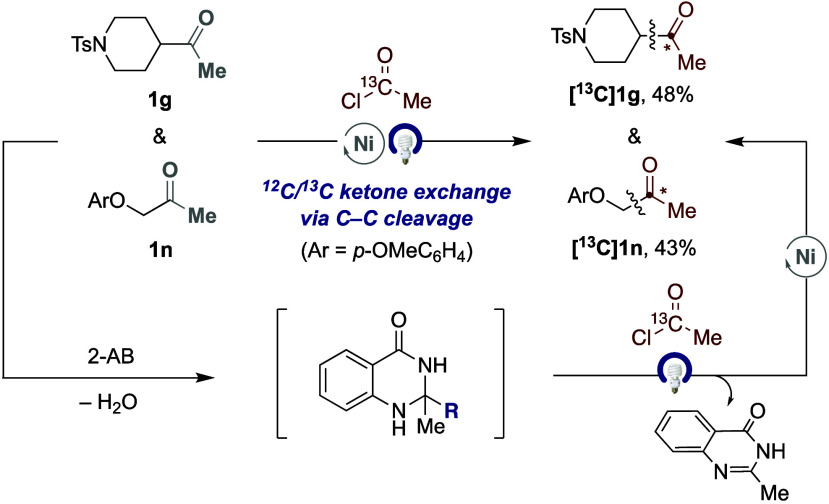
One-Pot ^12^C/^13^C-Exchange of Ketones

Next, we turned our attention to understanding the intricacies of our reaction by conducting stoichiometric experiments with well-defined organometallic Ni complexes (Scheme 3, *top*). To this end, we synthesized **Ni–I** by simple exposure of Ni­(cod)_2_, **L1** and benzoyl chloride in Et_2_O, the structure of which was unambiguously confirmed by X-ray crystallography.^11^ As anticipated, **Ni–I** was found to be catalytically competent as an intermediate in the reaction of **2z** with benzoyl chloride, affording **[** ^**13**^ **C]­1z** in 45% yield. Interestingly, however, a stoichiometric reaction of **2z** and **Ni–I** delivered traces of **[13C]­1z**, even in the presence of Ir­(dFCF_3_ppy)_2_(dtbbpy)­PF_6_ (**PC1**) under 456 nm Blue-LEDs. Given that **Ni–I** has a significant absorption at 456 nm, we believe these results can be interpreted on the basis of a photoexcitation event that releases chlorine or acetyl radicals,^5a,5g,10a,10e,14,15^ thus setting the basis for decomposition pathways via disproportionation at high concentrations.^16^ Taking these observations into account, we believe our results are consistent with an initial oxidative addition of ^13^C-acyl chloride to Ni^0^ en route to **III** (Scheme 3, bottom). The latter can be intercepted by open-shell intermediate **II** generated upon single electron transfer between the photoexcited state of Ir­(dFCF_3_ppy)_2_(dtbbpy)­PF_6_ (+1.21 V vs SCE) and **I**. Reductive elimination from **IV** results in the targeted product while generating Ni­(I) intermediate **V**. A final downhill single-electron transfer reduction of the latter (*E*_1/2_^red^ Ni^I^/Ni^0^ ∼ −1.13 V)^17^ with the reduced form of the Ir photocatalyst (*E*_red_ = −1.37 V vs SCE) might recover back the propagating Ir^III^ and Ni(0) catalysts. At present, however, we cannot rule out an alternative pathway consisting of the intermediacy of chlorine radicals via either photoexcitation of **III** or energy transfer from **PC1** that might be responsible for generating **II** from **I**.

**Scheme 3:**
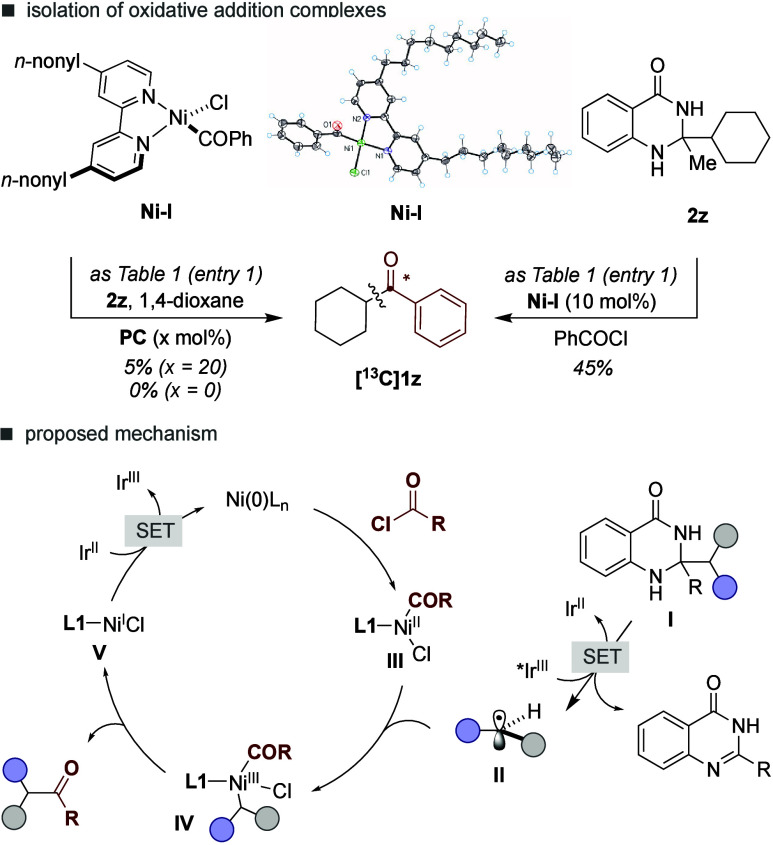
Mechanistic Considerations

## Conclusion

In summary, we have developed a catalytic ^12^C/^13^C exchange that converts regular ketones into their ^13^C-labeling analogues. The method leverages the potential of intermediate proaromatic precursors to generate open-shell species prior to their interception with appropriate ^13^C-labeled sources. Given the ubiquity of ketones in a myriad of advanced intermediates, the ability to promote a formal ^12^C/^13^C carbonyl metathesis without changing the already established sequence en route to the parent compound might offer a new gateway to generate preclinical data in the Drug Discovery pipeline.

## Methods

### General Procedure 1 (GP1)

An oven-dried 8 mL screw-cap test tube containing a stirring bar was charged with **2a** (0.2 mmol), 4,4′-dinonyl-2,2′-bipyridine (8.2 mg, 10 mol %) and Ir­(dF­(CF_3_)­ppy)_2_(dtbpy)­PF_6_ (4.4 mg, 2 mol %). The test tube was introduced in a nitrogen-filled glovebox where NiCl_2_·DME (2.2 mg, 5 mol %) was added. The reaction vessel was sealed with a screw cap and removed from the glovebox. Afterward, ^13^C-acyl chloride (1.5 equiv) and 1,4-dioxane (2 mL) were added. Parafilm was used to reseal the pierced cap. The reaction mixture was exposed to 456 nm LED irradiation at room temperature for 24 h. The reaction mixture was quenched with water/brine (10 mL) and extracted with diethyl ether (3 × 10 mL). The combined organic layer was dried over Na_2_SO_4_, filtered, and concentrated. The crude product was purified by silica gel chromatography.

## Supplementary Material





## Notes

Detailed experimental procedures, characterization data, and NMR spectra for all new compounds (PDF)
Data for **Ni–I** (CCDC-2485402) (CIF)

## References

[1] a Halliday, D. ; Lockhart, I. M. The Use of Stable Isotopes in Medicinal Chemistry. In Progress in Medicinal Chemistry; Elsevier, 1978; Vol. 15, pp 1–86. 400610 10.1016/s0079-6468(08)70253-4

[2] Isin E. M., Elmore C. S., Nilsson G. N., Thompson R. A., Weidolf L. (2012). Use of Radiolabeled
Compounds in Drug Metabolism and Pharmacokinetic Studies. Chem. Res. Toxicol..

[3] Hinsinger K., Pieters G. (2019). The emergence of carbon isotope exchange. Angew. Chem., Int. Ed..

[4] Maxwell B. D. (2018). New Radical
Methods for the Potential Synthesis of Carbon-13 and Carbon-14 Labeled
Complex Products. J. Label. Compd. Radiopharm..

[5] Mühlfenzl K. S., Enemaerke V. J., Gahlawat S., Golbaekdal P. I., Munksgaard-Ottosen N., Neumann K. T., Hopmann K. H., Norrby P.-O., Elmore C. S., Skrydstrup T. (2024). Nickel Catalyzed Carbonylative Cross
Coupling for Direct Access to Isotopically Labeled Alkyl Aryl Ketones. Angew. Chem., Int. Ed..

[6] Morrison K. C., Hergenrother P. J. (2014). Natural
Products as Starting Points
for the Synthesis of Complex and Diverse Compounds. Nat. Prod Rep.

[7] Liang Y.-F., Bilal M., Tang L.-Y., Wang T.-Z., Guan Y.-Q., Cheng Z., Zhu M., Wei J., Jiao N. (2023). Carbon–Carbon
Bond Cleavage for Late-Stage Functionalization. Chem. Rev..

[8] Lutz M. D. R., Morandi B. (2021). Metal-Catalyzed Carbon–Carbon
Bond Cleavage of Unstrained Alcohols. Chem.
Rev..

[9] Ye P., Xiong Y.-Y., Zhang B. (2025). Electrochemical
C–H
alkylation of *N*-heterocycles via aromatization-driven
C–C fragmentation of unstrained ketones. Tetrahedron Lett..

[10] Koo Y., Hong S. (2024). Nickel/photoredox-catalyzed
three-component silylacylation of acrylates *via* chlorine
photoelimination. Chem. Sci..

[11] Cong F., Mega R. S., Chen J., Day C. S., Martin R. (2023). Trifluoromethylation
of carbonyl and unactivated olefin derivatives by C­(sp^3^)-C bond-cleavage. Angew. Chem., Int. Ed..

[12] See Supporting Information for details.

[13] De Meijere, A. ; Diedrich, F. Metal-Catalyzed Cross-Coupling Reactions, 2nd ed.; Wiley-VCH, Weinheim, Germany, 2004.

[14] Shields B. J., Doyle A. G. (2016). Direct
C­(sp3)–H Cross Coupling Enabled by Catalytic Generation of
Chlorine Radicals. J. Am. Chem. Soc..

[15] See Supporting Information for control experiments resulting from the generation of chlorine radical intermediates.

[16] Day C. S., Martin R. (2023). Comproportionation
and disproportionation in nickel
and copper complexes. Chem. Soc. Rev..

[17] Börjesson M., Moragas T., Martin R. (2016). J. Am. Chem. Soc..

