# Supplementary material for: An Integrated 12C/13C Exchange Platform for Accessing Isotopically-Labeled Ketones via Dual Catalytic C–C Bond-Functionalization
Source: ACS Catal. 2025 Dec 24;16(2):960–5. doi: 10.1021/acscatal.5c06571 (PMC12817308; doi:10.1021/acscatal.5c06571)
Supplement: Supplementary file 1 [file cs5c06571_si_001.pdf]

Supplementary Information

An Integrated $^{12}C/^{13}C$ Exchange Platform for Accessing Isotopically-Labelled Ketones via Dual Catalytic C–C Bond-Functionalization

Hui-Qing Geng,[#] Álvaro Velasco-Rubio,[#ξ] Eva Jaramillo-Cassà,[#ςξ] Daniel Kócsi,[#ξ] Pablo García-Losada,[§] Oscar de Frutos[§] and Ruben Martin*[#†]

[#] Institute of Chemical Research of Catalonia (ICIQ), The Barcelona Institute of Science and Technology, Av. Països Catalans 16, 43007 Tarragona, Spain

[ς]Universitat Rovira i Virgili, Departament de Química Orgànica, c/ Marcel·lí Domingo, 1, 43007 Tarragona, Spain

[§] Centro de Investigación Lilly S.A., Avda de la Industria 30, 28108 Alcobendas-Madrid, Spain

[†] ICREA, Passeig Lluís Companys, 23, 08010, Barcelona, Spain

E-mail: rmartinromo@iciq.es

Table of Contents

1. General Information. *3*

2. General procedures *5*

3. Optimization details. *8*

4. Synthesis of Starting Materials *10*

5. $^{12}C/^{13}C$ Exchange via Dual Catalytic C-C Bond-Functionalization *23*

6. Low yielding and unsuccessful examples *38*

7. Mechanistic Experiments *39*

7.1 Experiments with well-defined nickel complex 39

7.2 Radical inhibition experiments 41

7.3 Chlorine radical trapping 42

7.4 UV-Vis. spectroscopy data 44

7.5 Fluorescence Quenching Studies 44

8. Crystal X-ray diffraction data *48*

9. NMR Spectra *58*

10. References *120*

1. General Information.

Reagents and solvents. Commercially available materials were used as received without further purification. Acetyl chloride-1-^{13}C (99.0 atom % ^{13}C) and benzoyl chloride-α-^{13}C (99.0 atom % ^{13}C) were purchased from Sigma-Aldrich, anhydrous 1,4-dioxane (99.5% purity) was purchased from Thermo Scientific Acros. Ir(dF(CF$_3$)ppy)$_2$(dtbpy)PF$_6$ and 4,4'-Dinonyl-2,2'-bipyridine was purchased from BLD Pharm. Nickel chloride dimethoxyethane was purchased from Fluorochem.

Analytical methods. ^{1}H and ^{13}C NMR spectra were recorded on Bruker 400 MHz at 20 °C. Chemical shifts for ^{1}H NMR are reported in parts per million (ppm) downfield from TMS, using residual solvent signals for calibration: water peak (3.33 ppm in DMSO-d$_6$, 1.56 ppm in CDCl$_3$), CHCl$_3$ (7.26 ppm) or DSMO (2.50 ppm), unless otherwise indicated. All ^{13}C NMR chemical shifts were referenced to TMS and calibrated against the residual signals of CHCl$_3$ (77.16 ppm) or DMSO (39.52 ppm). ^{19}F NMR and ^{11}B NMR was obtained with ^{1}H decoupling unless otherwise indicated. Coupling constants (J) are given in hertz (Hz). Infra-red (IR) spectra were recorded on a Bruker Optics FT-IR Alpha spectrometer (DTGS detector, KBr beamsplitter) at a resolution of 4 cm^{-1} using a single-bounce ATR accessory with diamond windows. High-resolution mass spectra (HRMS) were obtained using either a Waters LCT Premier spectrometer or a MicroTOF Focus, Bruker Daltonics spectrometer using electrospray ionisation (ESI). Flash chromatography was performed using silica gel (Sigma-Aldrich, pore size 60 Å, 230–400 mesh). Thin layer chromatography was used to monitor reaction progress and to analyze fractions collected from column chromatography. TLC was performed on Merck Silica gel 60 F$_{254}$ aluminium-backed plates, with visualization under UV irradiation and/or staining with potassium permanganate (KMnO$_4$) solution and and were developed by heating with a heat gun. Gas chromatography (GC) was performed on Agilent 7890A with FID detector and a fused silica column HP–5 (19091J–413; 30 m × 0.32 mm × 0.25 µm), analysis method: 6.5 mL/min flow, 24.7 psi, 70 °C, 1 min isocratic, 70–220 °C, 20 °C/min, 2 min isocratic; 220–300 °C, 35 °C/min, 2 min isocratic; n-decane was used as internal standard. Experiments were carried out at room temperature (25 °C) unless otherwise noted. Sample for UV-Vis. analysis was prepared in a 3 mL quartz cuvette (path length: l = 1.0 cm) equipped with a rubber septum screwcap. A 20 µM solution of **Ni-I** was prepared in a nitrogen-filled glovebox from dry and degassed MeCN. UV-Vis measurements were carried out on an Agilent Cary 60 UV-Vis spectrophotometer. The melting points were determined using open

glass capillaries with a Büchi B540 apparatus and are reported without correction. Unless otherwise specified, the samples were recrystallized by evaporating a solution of dichloromethane (CH_2Cl_2).

Light source. A custom-designed photoreactor was fabricated using a 3D printer, following established protocols.[1] The reactor was equipped with a 456 nm Kessil lamp (PR160L-456 nm, 40 W), operated at full intensity. To maintain a stable reaction temperature, an integrated cooling system was employed: a fan positioned beneath the setup directed airflow through a narrow slit at the base of the reactor. The interior walls were lined with UV-shielding paper to enhance safety and minimize light leakage. The reactor accommodated up to eight reaction tubes simultaneously and was mounted on a magnetic stirrer operating at 600–650 rpm. Additional cooling was provided by an external fan positioned approximately 20 cm in front of the reactor.

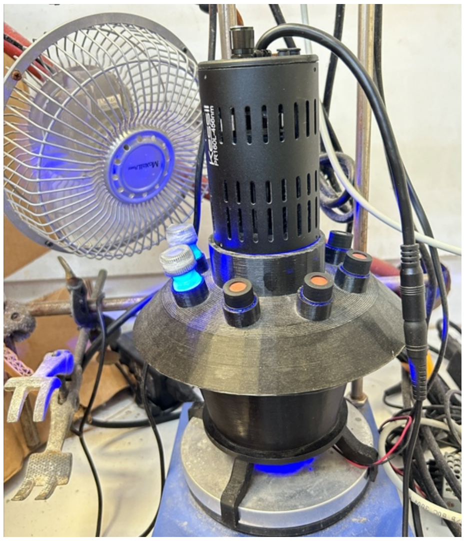

Figure S1. Photochemical reaction equipment.

2. General procedures

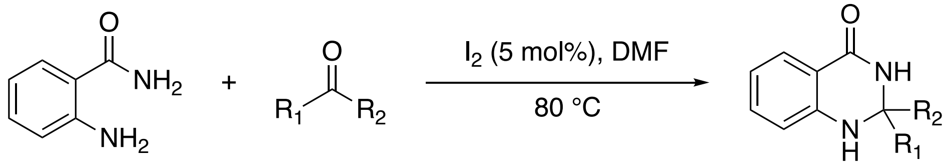

General procedure (GP1): synthesis 2,2-disubstituted dihydroquinazolinones. A 100 mL round-bottom flask equipped with a magnetic stirring bar was charged with 2-aminobenzamide (1.05 equiv.), ketone (1.0 equiv.), iodine (5 mol%) and DMF (0.67 M). The reaction mixture was stirred at 80 °C for 24 hours. After the reaction mixture was cooled to room temperature (20 °C), water (50 mL) was added, resulting in the formation of the precipitate. The solid was collected by suction filtration, washed with water, and recrystallized from diethyl ether (Et_2O) and *n*-hexane to afford the desired product. The yields, structures and characterization data are listed below in section 4 *"Synthesis of starting materials"*. The following dihydroquinazolinones were prepared according to literature procedures: **2a**,[2] **2b**,[3] **2c**,[2] **2f**,[2] **2g**,[4] **2k**,[2] **2m**,[5] **2n**,[2] **2s**,[5] **2t**,[6] **2u**,[2] **2x**,[4] **2y**,[4] **2z**.[7]
Note: For one-pot reactions the formation of the dihydroquinazolinone was carried out in the presence of Cp_2TiCl_2 (2 mol%) in EtOH at 70 ºC.[7]

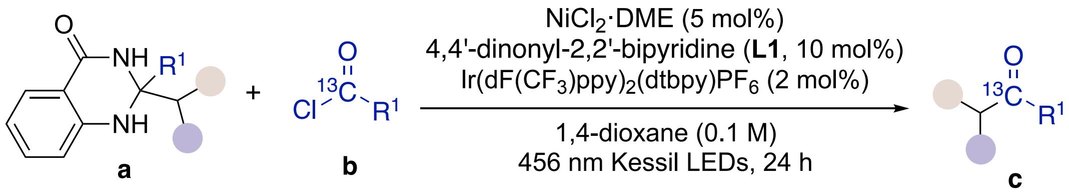

General procedure (GP2): Photoredox/nickel dual-catalyzed ^{13}C labeling acylation. An oven-dried 8 mL screw-cap test tube containing a stirring bar was charged with **a** (0.2 mmol), 4,4'-dinonyl-2,2'-bipyridine (8.2 mg, 10 mol%), $Ir(dF(CF_3)ppy)_2(dtbpy)PF_6$ (4.4 mg, 2 mol%). The test tube was introduced in a nitrogen-filled glovebox where $NiCl_2 \cdot DME$ (2.2 mg, 5 mol%) was added. The reaction vessel was sealed with a screw cap and removed from the glovebox. Afterwards, acyl chloride **b** (1.5 equiv.) and 1,4-dioxane (2 mL) were added by syringe. Parafilm was used to reseal the pierced cap. The reaction mixture was exposed to 456 nm LED irradiation at room temperature for 24 hours. The reaction mixture was quenched with a mixture of water and brine (1:1, 10 mL) and extracted with diethyl ether (3 x 10 mL). The combined organic phase was dried over Na_2SO_4,

filtered and concentrated under reduced pressure. The crude product was purified by silica gel chromatography.

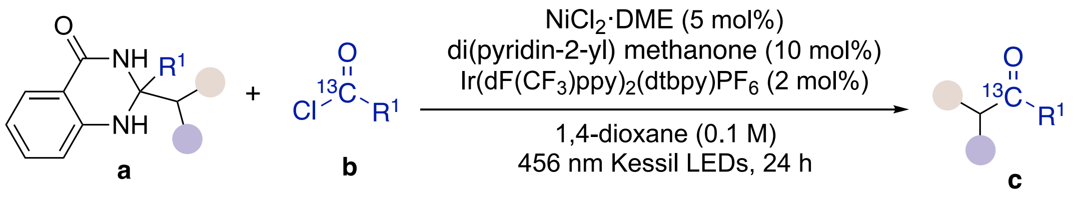

General procedure (GP3): Photoredox/nickel dual-catalyzed ^{13}C labeling acylation. An oven-dried 8 mL screw-cap test tube containing a stirring bar was charged with **a** (0.2 mmol), di(pyridin-2-yl) methanone (3.7 mg, 10 mol%), $Ir(dF(CF_3)ppy)_2(dtbpy)PF_6$ (4.4 mg, 2 mol%). The test tube was introduced in a nitrogen-filled glovebox where $NiCl_2$·dme (2.2 mg, 5 mol%) was added. The reaction vessel was sealed with a screw cap and removed from the glovebox. Afterwards, acyl chloride **b** (1.5 equiv.) and 1,4-dioxane (2 mL) were added by syringe. Parafilm was used to reseal the pierced cap. The reaction mixture was exposed to 456 nm LED irradiation at room temperature for 24 hours. The reaction mixture was quenched with a mixture of water and brine (1:1, 10 mL) and extracted with diethyl ether (3 x 10 mL). The combined organic phase was dried over Na_2SO_4, filtered and concentrated under reduced pressure. The crude product was purified by silica gel chromatography.

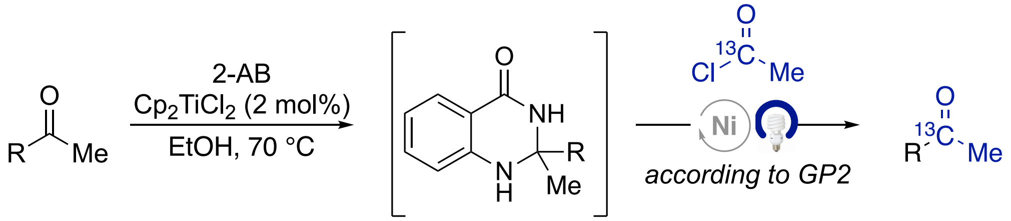

General procedure (GP4): Procedure for One-Pot ^{13}C-labelled acylation. An oven-dried 8 mL screw-cap test tube equipped with a magnetic stirring bar was charged with ketone (0.2 mmol), 2-aminobenzamide (1.05 equiv.), Cp_2TiCl_2 (2 mol%), EtOH (0.1 M) was added, and the mixture was stirred at 70 °C. Full consumption of the ketone usually took 2-3 h. The reaction mixture was filtered through a mixture of celite and Florisil®, washed with EtOH and concentrated in vacuum to give the crude dihydroquinazolinone. Then the oven-dried 8 mL screw-cap test tube containing a stirring bar was charged with the crude dihydroquinazolinone, 4,4'-dinonyl-2,2'-bipyridine (8.2 mg, 10 mol%), $Ir(dF(CF_3)ppy)_2(dtbpy)PF_6$ (4.4 mg, 2 mol%). The test tube was introduced in a nitrogen-filled glovebox where $NiCl_2$·dme (2.2 mg, 5 mol%) was added. The reaction vessel was sealed with a screw cap and removed from the glovebox. Afterwards, acetyl-1-^{13}C chloride (1.5

equiv., 23.4 mg) and 1,4-dioxane (2 mL) were added by syringe. Parafilm was used to reseal the pierced cap. The reaction mixture was exposed to 456 nm LED irradiation at room temperature for 24 hours. The reaction mixture was quenched with water/brine (10 mL) and extracted with diethyl ether (3 x 10 mL). The combined organic phase was dried over Na_2SO_4, filtered and concentrated under reduced pressure. The crude product was purified by silica gel chromatography.

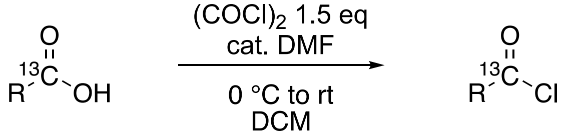

General procedure (GP5): synthesis of ^{13}C-acyl chloride. A solution of the ^{13}C-labeled carboxylic acid (1.0 equiv.) was dissolved in DCM (0.3 M), 5-10 drops of DMF was added and cooled to 0 ºC with an ice bath. Oxalyl chloride (1.5 equiv) was added dropwise (caution: gas evolution). The mixture was stirred at 0 ºC for 30 minutes, the ice bath was removed and continued stirring overnight while slowly let it warm up to rt. The mixture was concentrated under vacuum to remove volatiles. The residue was taken up in DCM, solid Na_2CO_3 was added to remove any acidic impurities. The mixture was further dried over Na_2SO_4, filtered and concentrated to yield ^{13}C-acyl chloride. The crude product was used without further purification.

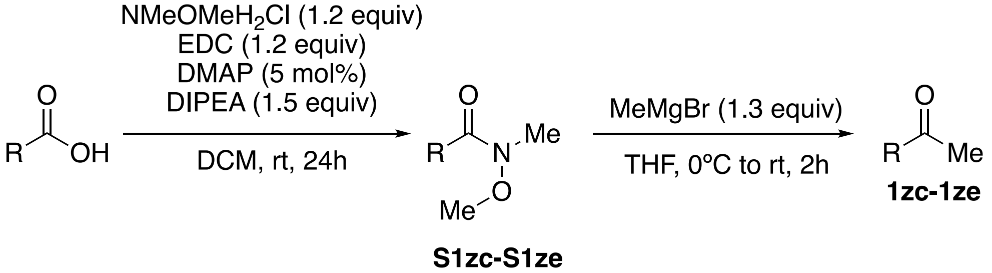

General procedure (GP 6): synthesis of Weinreib amides (S1zc-S1ze).

To a solution of a carboxylic acid (1 equiv) and *N*,*O*-dimethylhydroylamine hydrochloride (1.2 equiv) in DCM (0.2 M) was added *N*-(3-dimethylaminopropyl)-*N'*-ethylcarbodiimide hydrochloride (EDC) (1.2 equiv), 4-(dimethylamino)pyridine (DMAP) (5 mol%) and *N*,*N*-diisopropylethylamine (1.2 equiv) at 0 ºC. The reaction was allowed to reach room temperature and then stir for 24 h at room temperature. Upon completion, the organic layer was washed with 0.2 M HCl (aq.) and sat. Na_2CO_3 (aq.). The combined organic layers were dried over anhydrous Na_2SO_4, filtered, and concentrated *in vacuo*. The crude product was purified by flash column chromatography using a mixture of *n*-hexanes:EtOAc to provide the desired Weinreb amide (**S1zc-S1ze**).

General procedure (GP 7): synthesis of methyl ketones (1zc-1ze).

To a solution of the Weinreb amide (1 equiv) in THF (0.2 M), MeMgBr (1.3 equiv, 3 M in Et_2O) was added dropwise at 0 ºC. The reaction was stirred at 0 ºC for 30 min and then 2 h at rt. Afterwards, the reaction was cool-down to 0 ºC and a saturated solution of NH_4Cl_{aq} (10 ml) was added, and stirred for 15 min. The reaction was extracted with EtOAc (3 x 30 mL), the combination of the organic phases was washed with a saturated solution of NH_4Cl_{aq} (30 mLl), dried over $MgSO_4$ and the crude product was purified by flash column chromatography using a mixture of *n*-hexanes:EtOAc as eluent to provide the desired ketones (**1zc-1ze**).

3. Optimization details.

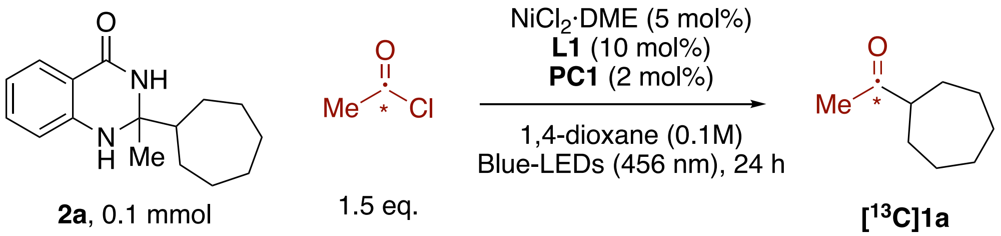

General Procedure for the optimization. An oven-dried 8 mL screw-cap test tube containing a stirring bar was charged with **2a** (0.1 mmol, 24.4 mg), 4,4'-dinonyl-2,2'-bipyridine (**L1**, 4.1 mg, 10 mol%), $Ir(dF(CF_3)ppy)_2(dtbpy)PF_6$ (2.2 mg, 2 mol%). The test tube was introduced in a nitrogen-filled glovebox where $NiCl_2{\cdot}DME$ (1.1 mg, 5 mol%) was added. The reaction vessel was sealed with a screw cap and removed from the glovebox. Afterwards, ^{13}C-acetyl chloride (1.5 equiv., 11.9 mg) and 1,4-dioxane (1 mL) were added by syringe. Parafilm was used to reseal the pierced cap. The reaction mixture was exposed to 456 nm LED irradiation at room temperature for 24 hours. After the reaction was completed, the mixture was filtered through celite and analyzed by GC using dodecane (17.0 mg, 0.1 mmol) as internal standard.

Table S1. Optimization of the reaction conditions.

entry	deviation from standard conditions.	**[13C]1a (%)[a]**
1	none	75
2	utilizing **L2** instead of **L1**	56
3	utilizing **L3** instead of **L1**	29
4	utilizing **L4** instead of **L1**	12
5	utilizing **L5** instead of **L1**	25
6	$Ni(COD)_2$ as catalyst	58
7	$NiBr_2$•dme as catalyst	70
8	$Ni(acac)_2$ as catalyst	56
9	DME as solvent	47
10	THF as solvent	33
11	Anisole as solvent	37
12	**PC2** as photocatalyst	26
13	4-CzIPN as photocatalyst	15
14	no $NiCl_2$•dme	0
15	no **PC1**	0

[a] GC yield using dodecane as internal standard

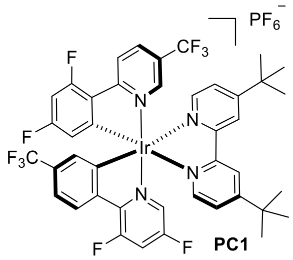

PC1

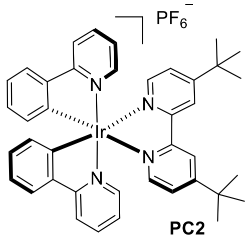

PC2

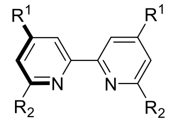

R^1 = H, R^2 = *n*-nonyl (**L1**)
R^1 = H, R^2 = *t*-Bu (**L2**)
R^1 = H, R^2 = COOMe (**L3**)
R^1 = Me, R^2 = H (**L4**)

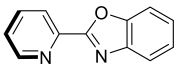

L5

4. Synthesis of Starting Materials

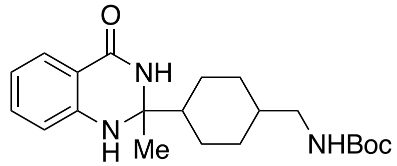

***tert*-Butyl-((4-(2-methyl-4-oxo-1,2,3,4-tetrahydroquinazolin-2-yl)cyclohexyl)methyl) carbamate (2d).** Following GP1, using 2-aminobenzamide (0.68 g, 5 mmol), *tert*-butyl ((4-acetylcyclohexyl)methyl)carbamate (1.34 g, 5.25 mmol) and iodine (63 mg, 5 mol%) in DMF (7.5 mL) at 80 °C for 24 hours. The product was obtained as a yellow solid (1.08 g, 58% yield, melting point: 217 °C). **^{1}H NMR** (400 MHz, DMSO-d_6) δ 8.15 – 7.71 (m, 1H), 7.52 (dd, J = 7.7, 1.6 Hz, 1H), 7.18 (ddd, J = 8.6, 7.2, 1.7 Hz, 1H), 6.76 (t, J = 5.7 Hz, 1H), 6.66 (dd, J = 8.1, 1.1 Hz, 1H), 6.60 – 6.45 (m, 2H), 2.72 (t, J = 6.1 Hz, 2H), 1.74 (dt, J = 24.9, 13.8 Hz, 4H), 1.50 (t, J = 12.0 Hz, 1H), 1.36 (s, 9H), 1.30 (s, 2H), 1.27 – 1.18 (m, 2H), 1.05 (q, J = 12.3 Hz, 2H), 0.80 – 0.66 (m, 2H). **^{13}C NMR** (101 MHz, DMSO-d_6) δ 163.3, 156.2, 147.5, 133.6, 127.5, 116.2, 114.1, 113.9, 77.7, 71.6, 48.3, 46.0, 38.2, 30.6, 30.5, 28.7, 26.5, 26.2, 25.6. **IR** (neat, cm^{-1}): 3307, 3977, 2925, 2857, 1692, 1652, 1614, 1516, 1487, 1366, 1275, 1250, 1171, 754. **HRMS** (ESI$^+$) calcd. for $C_{21}H_{31}N_3NaO_3$ [M+Na]$^+$ 396.2263, found 396.2258.

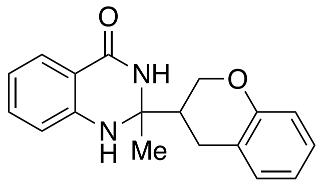

2-(Chroman-3-yl)-2-methyl-2,3-dihydroquinazolin-4(1*H*)-one (2e). Following GP1, using 2-aminobenzamide (0.68 g, 5 mmol), 1-(chroman-3-yl)ethan-1-one (0.92 g, 5.25 mmol) and iodine (63 mg, 5 mol%) in DMF (7.5 mL) at 80 °C for 24 hours. The product was obtained as a white solid (1.28 g, 65% yield, d.r. = 1:1, melting point: 105 °C). **^{1}H NMR** (400 MHz, DMSO-d_6) δ 8.17 (d, J = 15.2 Hz, 1H), 7.58 (d, J = 7.7 Hz, 1H), 7.28 – 7.16 (m, 1H), 7.12 – 6.95 (m, 2H), 6.85 – 6.77 (m, 2H), 6.73 (ddd, J = 8.0, 3.9, 2.1 Hz, 2H), 6.67 – 6.57 (m, 1H), 4.55 – 4.22 (m, 1H), 3.80 (td, J = 10.6, 5.5 Hz, 1H), 2.87 (dd, J = 16.1, 12.0 Hz, 1H), 2.72 (ddd, J = 30.9, 16.3, 3.7 Hz, 1H), 2.36 – 2.16 (m, 1H), 1.40 (s, 3H). **^{13}C NMR** (101 MHz, DMSO-d_6) δ 163.4, 154.5, 147.1, 133.9, 130.5, 127.6, 127.5, 122.3, 120.7, 117.1, 116.5, 114.6, 114.1, 70.2, 66.7, 42.5, 26.3, 25.5. **IR** (neat, cm^{-1}): 3294, 2986, 2896, 1705, 1648, 1611, 1583, 1512, 1487, 1362, 1226, 1151, 1015, 750, 710. **HRMS** (ESI$^+$) calcd. for $C_{18}H_{19}N_2O_2$ [M+H]$^+$ 295.1441, found 295.1443.

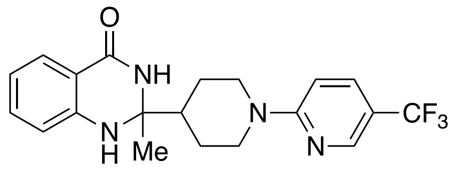

2-Methyl-2-(1-(5-(trifluoromethyl)pyridin-2-yl)piperidin-4-yl)-2,3-dihydroquinazolin-4(1*H*)-one (2h). Following GP1, using 2-aminobenzamide (0.68 g, 5 mmol), 1-(1-(5-(trifluoromethyl)pyridin-2-yl)piperidin-4-yl)ethan-1-one (1.42 g, 5.25 mmol) and iodine (63 mg, 5 mol%) in DMF (7.5 mL) at 80 °C for 24 hours. The product was obtained as a yellow solid (1.13 g, 58% yield, melting point: 226 °C). **^{1}H NMR** (400 MHz, DMSO-d_6) δ 8.43 – 8.31 (m, 1H), 7.97 (s, 1H), 7.73 (dd, J = 9.2, 2.5 Hz, 1H), 7.54 (dd, J = 7.7, 1.5 Hz, 1H), 7.19 (ddd, J = 8.6, 7.3, 1.6 Hz, 1H), 6.94 (d, J = 9.2 Hz, 1H), 6.66 (d, J = 8.2 Hz, 2H), 6.60 – 6.49 (m, 1H), 4.52 (t, J = 14.4 Hz, 2H), 2.76 (q, J = 12.9 Hz, 2H), 1.94 – 1.83 (m, 1H), 1.77 (t, J = 15.3 Hz, 2H), 1.40 – 1.24 (m, 5H). **^{13}C NMR** (101 MHz, DMSO-d_6) δ 162.8, 159.8, 146.9, 145.3 (q, J = 8.6Hz), 134.4 (q, J = 6.2 Hz), 133.3, 127.0, 124.9 (q, J = 270.0 Hz), 115.9, 113.6, 113.3, 112.5 (q, J = 32.6 Hz), 106.0, 70.8, 46.7, 44.4, 30.7, 25.7, 25.4, 25.1. ^{19}F NMR (376 MHz, DMSO-d_6) δ -59.2. **IR** (neat, cm^{-1}): 3297, 2943, 2851, 1654, 1613, 1515, 1331, 1316, 1241, 1112, 1081, 756. **HRMS** (ESI$^+$) calcd. for $C_{20}H_{22}F_3N_4O$ [M+H]$^+$ 391.1740, found 391.1750.

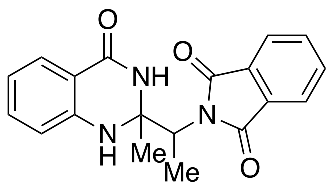

2-(1-(2-Methyl-4-oxo-1,2,3,4-tetrahydroquinazolin-2-yl)ethyl)isoindoline-1,3-dione (2i). Following GP1, using 2-aminobenzamide (0.68 g, 5 mmol), 2-(3-oxobutan-2-yl)isoindoline-1,3-dione (1.14 g, 5.25 mmol) and iodine (63 mg, 5 mol%) in DMF (7.5 mL) at 80 °C for 24 hours. The product was obtained as a yellow solid (1.09 g, 65% yield, d.r. = 1:1, melting point: 102 °C). **^{1}H NMR** (400 MHz, DMSO-d_6) δ 8.17 – 8.05 (m, 1H), 7.82 (d, J = 6.9 Hz, 4H), 7.53 (ddd, J = 8.0, 6.6, 1.6 Hz, 1H), 7.19 (dddd, J = 8.6, 7.4, 5.7, 1.6 Hz, 1H), 7.03 – 6.89 (m, 1H), 6.68 – 6.42 (m, 2H), 4.47 (dq, J = 11.3, 7.3 Hz, 1H), 1.67 – 1.36 (m, 6H). **^{13}C NMR** (101 MHz, DMSO-d_6) δ 167.1, 162.9, 146.4, 134.7, 133.5, 129.2, 127.6, 123.4, 116.6, 114.9, 114.1, 72.1, 53.8, 24.9, 12.5.

IR (neat, cm^{-1}): 3338, 1775, 1706, 1651, 1614, 1519, 1486, 1357, 1332, 1044, 755, 723. **HRMS** (ESI^+) calcd. for $C_{19}H_{18}N_3O_3$ $[M+H]^+$ 336.1343, found 336.1342.

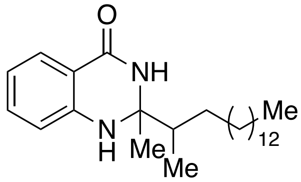

2-(Hexadecan-2-yl)-2-methyl-2,3-dihydroquinazolin-4(1*H*)-one (2j). Following GP1, using 2-aminobenzamide (0.68 g, 5 mmol), 3-methylheptadecan-2-one (1.41 g, 5.25 mmol) and iodine (63 mg, 5 mol%) in DMF (7.5 mL) at 80 °C for 24 hours. The product was obtained as a white solid (1.31 g, 68% yield, melting point: 141 °C). **^{1}H NMR** (400 MHz, DMSO-d_6) δ 7.94 (d, *J* = 13.4 Hz, 1H), 7.52 (dd, *J* = 7.7, 1.6 Hz, 1H), 7.25 – 7.04 (m, 1H), 6.70 – 6.60 (m, 2H), 6.56 (tt, *J* = 7.3, 1.8 Hz, 1H), 1.69 (d, *J* = 7.5 Hz, 1H), 1.61 – 1.50 (m, 1H), 1.36 – 1.16 (m, 27H), 1.01 – 0.90 (m, 1H), 0.86 (td, *J* = 7.1, 5.2 Hz, 6H). **^{13}C NMR** (101 MHz, DMSO-d_6) δ 163.3, 147.4, 133.6, 127.4, 116.3, 114.4, 114.3, 71.9, 42.4, 31.8, 30.4, 29.7, 29.5, 29.5, 29.4, 29.3, 29.2, 28.1, 24.2, 24.0, 22.6, 14.4, 14.0. **IR** (neat, cm^{-1}): 3310, 3186, 2921, 2852, 1653, 1633, 1611, 1514, 1399, 1331, 1276, 1151, 753. **HRMS** (ESI^+) calcd. for $C_{25}H_{43}N_2O$ $[M+H]^+$ 387.3370, found 387.3370.

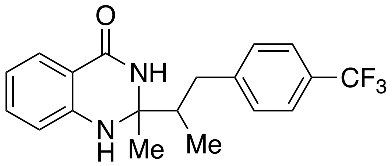

2-Methyl-2-(1-(4-(trifluoromethyl)phenyl)propan-2-yl)-2,3-dihydroquinazolin-4(1*H*)-one (2l). Following GP1, using 2-aminobenzamide (0.68 g, 5 mmol), 3-methyl-4-(4-(trifluoromethyl)phenyl)butan-2-one (1.21 g, 5.25 mmol) and iodine (63 mg, 5 mol%) in DMF (7.5 mL) at 80 °C for 24 hours. The product was obtained as a brown solid (1.22 g, 70% yield, d.r. = 1:1, melting point: 185 °C). **^{1}H NMR** (400 MHz, DMSO-d_6) δ 8.28 – 8.05 (m, 1H), 7.69 – 7.55 (m, 3H), 7.32 (t, *J* = 7.5 Hz, 2H), 7.23 (dddd, *J* = 13.6, 8.4, 7.2, 1.7 Hz, 1H), 6.85 – 6.68 (m, 2H), 6.61 (qd, *J* = 7.4, 1.1 Hz, 1H), 3.13 (t, *J* = 12.1 Hz, 1H), 2.30 (q, *J* = 12.1 Hz, 1H), 2.18 – 1.90 (m, 1H), 1.40 (s, 3H), 0.77 (dd, *J* = 13.6, 6.8 Hz, 3H). **^{13}C NMR** (101 MHz, DMSO-d_6) δ 162.9, 146.8, 146.4, 133.3, 129.6, 127.1, 126.5 (d, *J* = 30.4 Hz), 125.1 (q, *J* = 4.0 Hz), 123.1, 116.2, 114.0, 113.8, 71.5, 44.7, 37.0, 24.5, 13.2. **^{19}F NMR** (376 MHz, DMSO-d_6) δ -61.02. **IR** (neat, cm^{-1}): 3297, 2975, 1655, 1615, 1511, 1486, 1390, 1325, 1162, 1116, 1067, 1019, 755. **HRMS** (ESI^+) calcd. for $C_{19}H_{20}F_3N_2O$ $[M+H]^+$ 349.1522, found 349.1522.

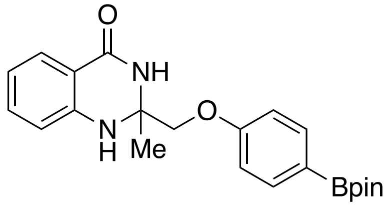

2-Methyl-2-((4-(4,4,5,5-tetramethyl-1,3,2-dioxaborolan-2-yl)phenoxy)methyl)-2,3-dihydroquinazolin-4(1*H*)-one (2o). Following GP1, using 2-aminobenzamide (0.68 g, 5 mmol), 1-(4-(4,4,5,5-tetramethyl-1,3,2-dioxaborolan-2-yl)phenoxy)propan-2-one (1.50 g, 5.25 mmol) and iodine (63 mg, 5 mol%) in DMF (7.5 mL) at 80 ºC for 24 hours. The product was obtained as a brown solid (1.16 g, 59% yield, melting point: 196 °C). ^{1}**H NMR** (400 MHz, $CDCl_3$) δ 7.87 (dd, *J* = 7.8, 1.6 Hz, 1H), 7.71 (d, *J* = 8.8 Hz, 2H), 7.29 (ddd, *J* = 8.7, 7.5, 1.8 Hz, 1H), 6.90 – 6.76 (m, 3H), 6.62 (d, *J* = 8.6 Hz, 1H), 6.44 (s, 1H), 4.16 (d, *J* = 8.8 Hz, 1H), 3.87 (d, *J* = 8.8 Hz, 1H), 1.68 (s, 3H), 1.32 (s, 12H). ^{13}**C NMR** (101 MHz, $CDCl_3$) δ 164.1, 160.7, 149.7, 145.6, 136.8, 134.4, 128.5, 119.4, 115.0, 114.6, 114.1, 83.8, 71.8, 68.9, 25.8, 25.0. ^{11}B NMR (128 MHz, $CDCl_3$) δ 31.07. **IR** (neat, cm^{-1}): 3293, 2979, 2931, 1660, 1604, 1511, 1468, 1392, 1360, 1319, 1244, 1143, 1091, 1045, 860, 840, 756, 733, 655. **HRMS** (ESI^+) calcd. for $C_{22}H_{28}N_2O_4{}^{10}B$ $[M+H]^+$ 394.2173, found 394.2171.

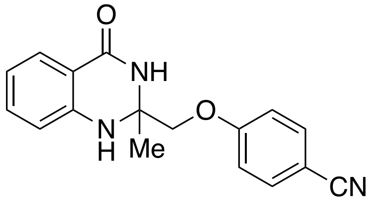

4-((2-Methyl-4-oxo-1,2,3,4-tetrahydroquinazolin-2-yl)methoxy)benzonitrile (2p). Following GP1, using 2-aminobenzamide (0.68 g, 5 mmol), 4-(2-oxopropoxy)benzonitrile (0.92 g, 5.25 mmol) and iodine (63 mg, 5 mol%) in DMF (7.5 mL) at 80 ºC for 24 hours. The product was obtained as a white solid (1.03 g, 70% yield, melting point: 198 °C). ^{1}**H NMR** (400 MHz, DMSO-*d*$_6$) δ 8.11 (s, 1H), 7.78 – 7.65 (m, 2H), 7.57 (dd, *J* = 8.0, 1.6 Hz, 1H), 7.30 – 7.14 (m, 1H), 7.05 – 6.97 (m, 2H), 6.90 (s, 1H), 6.66 – 6.57 (m, 2H), 4.09 – 3.90 (m, 2H), 1.48 (s, 3H). ^{13}**C NMR** (101 MHz, DMSO-*d*$_6$) δ 163.0, 161.8, 146.7, 134.1, 133.3, 127.0, 119.0, 116.6, 115.8, 113.9, 113.7, 103.1, 73.4, 68.1, 24.8. **IR** (neat, cm^{-1}): 3295, 2224, 1657, 1605, 1508, 1486, 1367, 1304, 1257, 1171, 1040, 835, 757. **HRMS** (ESI^+) calcd. for $C_{17}H_{16}N_3O_2$ $[M+H]^+$ 294.1237, found 294.1231.

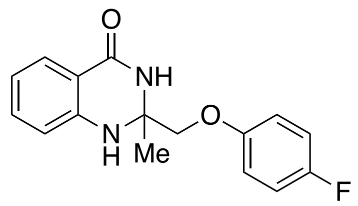

2-((4-Fluorophenoxy)methyl)-2-methyl-2,3-dihydroquinazolin-4(1*H*)-one (2q). Following GP1, using 2-aminobenzamide (0.68 g, 5 mmol), 1-(4-fluorophenoxy)propan-2-one (0.88 g, 5.25 mmol) and iodine (63 mg, 5 mol%) in DMF (7.5 mL) at 80 °C for 24 hours. The product was obtained as a brown solid (0.92 g, 64% yield, melting point: 158 °C). **^{1}H NMR** (400 MHz, DMSO-*d_6*) δ 8.07 (d, *J* = 1.7 Hz, 1H), 7.57 (dd, *J* = 7.7, 1.6 Hz, 1H), 7.20 (ddd, *J* = 8.1, 7.2, 1.7 Hz, 1H), 7.11 – 6.98 (m, 2H), 6.92 – 6.76 (m, 3H), 6.74 – 6.47 (m, 2H), 3.87 (dd, *J* = 48.6, 9.4 Hz, 2H), 1.47 (s, 3H). **^{13}C NMR** (101 MHz, DMSO-*d_6*) δ 163.1, 156.6 (d, *J* = 236.0 Hz), 154.8 (d, *J* = 2.1 Hz), 146.8, 133.3, 127.0, 116.5, 116.1 (d, *J* = 8.1 Hz), 115.8 (d, *J* = 22.9 Hz), 113.9, 113.7, 73.9, 68.2, 24.8. ^{19}F NMR (376 MHz, DMSO-*d_6*) δ -123.60. **IR** (neat, cm^{-1}): 3291, 3061, 2928, 1655, 1615, 1505, 1486, 1368, 1247, 1206, 1050, 828, 756. **HRMS** (ESI$^+$) calcd. for $C_{16}H_{16}FN_2O_2$ [M+H]$^+$ 287.1190, found 287.1187.

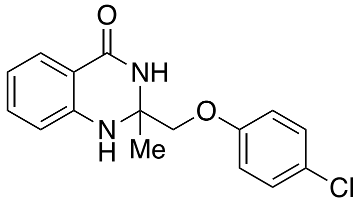

2-((4-Chlorophenoxy)methyl)-2-methyl-2,3-dihydroquinazolin-4(1*H*)-one (2r). Following GP1, using 2-aminobenzamide (0.68 g, 5 mmol), 1-(4-chlorophenoxy)propan-2-one (0.97 g, 5.25 mmol) and iodine (63 mg, 5 mol%) in DMF (7.5 mL) at 80 °C for 24 hours. The product was obtained as a white solid (1.00 g, 66% yield, melting point: 168 °C). **^{1}H NMR** (400 MHz, DMSO-*d_6*) δ 8.08 (d, *J* = 1.6 Hz, 1H), 7.57 (dd, *J* = 7.7, 1.6 Hz, 1H), 7.30 – 7.23 (m, 2H), 7.20 (ddd, *J* = 8.2, 7.2, 1.6 Hz, 1H), 6.91 – 6.83 (m, 3H), 6.71 – 6.53 (m, 2H), 3.88 (dd, *J* = 46.3, 9.5 Hz, 2H), 1.47 (s, 3H). **^{13}C NMR** (101 MHz, DMSO-*d_6*) δ 163.0, 157.3, 146.8, 133.3, 129.2, 127.0, 124.5, 116.6, 116.5, 113.9, 113.7, 73.5, 68.1, 24.8. **IR** (neat, cm^{-1}): 3299, 1657, 1616, 1490, 1373, 1282, 1243, 1169, 1093, 1048, 823, 756. **HRMS** (ESI$^+$) calcd. for $C_{16}H_{16}ClN_2O_2$ [M+H]$^+$ 303.0895, found 303.0895.

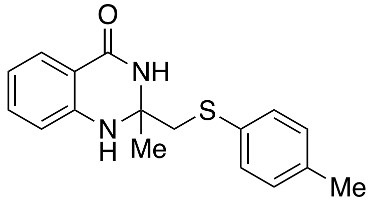

2-methyl-2-((*p*-tolylthio)methyl)-2,3-dihydroquinazolin-4(1*H*)-one (2v). Following GP1, using 2-aminobenzamide (0.68 g, 5 mmol), 1-(*p*-tolylthio)propan-2-one (0.95 g, 5.25 mmol) and iodine (63 mg, 5 mol%) in DMF (7.5 mL) at 80 ºC for 24 hours. The product was obtained as a brown solid (1.04 g, 70% yield, melting point: 92 °C). **^{1}H NMR** (400 MHz, DMSO-d_6) δ 8.07 (d, *J* = 1.7 Hz, 1H), 7.56 (dd, *J* = 7.7, 1.6 Hz, 1H), 7.28 – 7.11 (m, 3H), 7.07 (d, *J* = 7.9 Hz, 2H), 6.82 (s, 1H), 6.67 – 6.52 (m, 2H), 3.27 – 3.14 (m, 2H), 2.24 (s, 3H), 1.44 (s, 3H). **^{13}C NMR** (101 MHz, DMSO-d_6) δ 163.2, 146.8, 135.8, 133.7, 133.5, 130.1, 129.7, 127.5, 117.0, 114.6, 114.0, 69.7, 45.7, 27.6, 21.0. **IR** (neat, cm^{-1}): 3294, 3040, 2919, 1655, 1614, 1485, 1381, 1276, 1163, 806, 754. **HRMS** (ESI$^+$) calcd. for $C_{17}H_{18}N_2OSNa$ [M+Na]$^+$ 321.1032, found 321.1034.

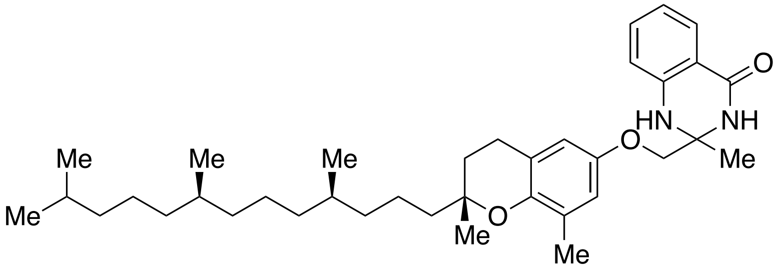

2-((((*R*)-2,8-dimethyl-2-((4*R*,8*R*)-4,8,12-trimethyltridecyl)chroman-6-yl)oxy)methyl)-2-methyl-2,3-dihydroquinazolin-4(1*H*)-one (2w). Following GP1, using 2-aminobenzamide (0.68 g, 5 mmol), 1-(((*R*)-2,8-dimethyl-2-((4*R*,8*R*)-4,8,12-trimethyltridecyl)chroman-6-yl)oxy)propan-2-one (2.41 g, 5.25 mmol) and iodine (63 mg, 5 mol%) in DMF (7.5 mL) at 80 ºC for 24 hours. The product was obtained as an orange oil (2.02 g, 70% yield). **^{1}H NMR** (400 MHz, DMSO-d_6) δ 8.04 (d, *J* = 1.6 Hz, 1H), 7.57 (dd, *J* = 7.7, 1.6 Hz, 1H), 7.19 (ddd, *J* = 8.5, 7.2, 1.7 Hz, 1H), 6.94 – 6.76 (m, 1H), 6.71 – 6.57 (m, 2H), 6.46 (d, *J* = 2.9 Hz, 1H), 6.37 (d, *J* = 3.0 Hz, 1H), 3.77 (dd, *J* = 60.0, 9.3 Hz, 2H), 2.59 (q, *J* = 6.2 Hz, 2H), 1.99 (s, 3H), 1.75 – 1.58 (m, 2H), 1.51 – 1.41 (m, 5H), 1.35 (d, *J* = 19.1 Hz, 5H), 1.29 – 1.17 (m, 7H), 1.14 (s, 3H), 1.12 – 0.96 (m, 7H), 0.86 – 0.78 (m, 12H). **^{13}C NMR** (101 MHz, DMSO-d_6) δ 163.1, 150.9, 146.8, 145.6, 133.2, 127.1, 125.8, 120.7, 116.4, 115.4, 114.0, 113.8, 112.2, 75.1, 73.4, 68.2, 36.8, 36.7, 36.7, 36.6, 32.0, 31.9, 30.8, 29.0, 27.3, 24.7, 24.1, 23.8, 23.7, 22.5, 22.4, 21.9, 20.3, 19.6, 19.5, 15.8. **IR** (neat, cm^{-1}): 3297, 2925, 2866, 1660, 1615, 1509, 1481, 1464, 1376, 1275, 1152, 1065, 753. **HRMS** (ESI$^+$) calcd. for $C_{37}H_{56}N_2O_3Na$ [M+Na]$^+$ 599.4183, found 599.4185.

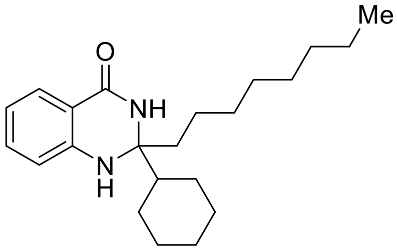

2-Cyclohexyl-2-octyl-2,3-dihydroquinazolin-4(1*H*)-one (**2za**).A screw-cap vial equipped with a magnetic stirring bar was charged with ketone (520 mg, 2.47 mmol, 1.05 equiv), 2-aminobenzamide (353 mg, 2.60 mmol, 1.0 equiv), Cp_2TiCl_2 (12 mg, 0.05 mmol, 0.02 equiv), EtOH (0.1 M) was added, and the mixture was stirred at 70 °C overnight. The solvent was evaporated and the crude product was directly purified by column chromatography using *n*-hexane:EtOAc (9:1) as the eluent. The product was obtained as an off-white amorphous solid (100 mg, 12% yield). **^{1}H NMR (**500 MHz, $CDCl_3$) δ 7.82 (dd, *J* = 7.8, 1.6 Hz, 1H), 7.25 – 7.21 (m, 1H), 6.72 (ddd, *J* = 7.9, 7.3, 1.0 Hz, 1H), 6.54 (dd, *J* = 8.1, 1.0 Hz, 1H), 5.77 (s, 1H), 4.02 (s, 1H), 1.92 – 1.75 (m, 4H), 1.70 – 1.54 (m, 6H), 1.44 – 1.32 (m, 3H), 1.29 – 1.05 (m, 10H), 0.90 – 0.81 (m, 3H). **^{13}C NMR** (126 MHz, $CDCl_3$) δ 164.2, 146.3, 134.0, 128.2, 117.7, 113.6, 74.5, 53.4, 47.5, 38.0, 31.8, 29.7, 29.2, 26.9, 26.8, 26.6, 26.2, 26.2, 23.3, 22.6, 14.1. **HRMS** (ESI$^+$) calcd. for $C_{21}H_{33}N_2O$ [M+H]$^+$ 329.2587, found 329.2578.

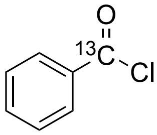

Benzoyl chloride-α-^{13}C (2zb).[8] Following GP5, using benzoic acid-α-^{13}C (300 mg, 2.44 mmol) affording the product as a pale-yellow liquid (145 mg, 42% yield). **^{1}H NMR** (500 MHz, $CDCl_3$) δ 8.17 – 8.09 (m, 2H), 7.72 – 7.64 (m, 1H), 7.52 (ddd, *J* = 8.8, 7.4, 1.5 Hz, 2H). **^{13}C NMR** (126 MHz, $CDCl_3$) δ 168.6, 135.5, 133.4 (d, *J* = 74.1 Hz), 131.6 (d, *J* = 3.3 Hz), 129.1 (d, *J* = 5.3 Hz). Spectral data was in agreement with the literature. [11]

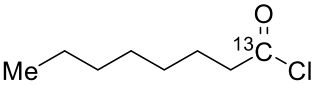

Octanoyl-1-^{13}C chloride (2zc). Following GP5, using octanoic acid-α-^{13}C (200 mg, 1.38 mmol) affording the product as a colorless liquid (200 mg, 89% yield). **^{1}H NMR** (500 MHz, $CDCl_3$) δ 2.88 (q, *J* = 7.5 Hz, 2H), 1.71 (td, *J* = 7.4, 5.8 Hz, 2H), 1.44 – 1.22 (m, 8H), 0.95 – 0.84 (m, 3H). **^{13}C NMR** (126 MHz, $CDCl_3$) δ 174.0, 47.2 (d, *J* = 52.9 Hz), 31.7, 28.9, 28.5 (d, *J* = 4.1 Hz), 25.2 (d, *J* = 2.2 Hz), 22.7, 14.2. Spectral data was in agreement with the literature.[12]

Synthesis of 1-((3a*R*,5*S*,5a*R*,8a*S*,8b*R*)-2,2,7,7-tetramethyltetrahydro-5*H*-bis([1,3]dioxolo)[4,5-*b*:4',5'-*d*]pyran-5-yl)ethan-1-one (1zb)

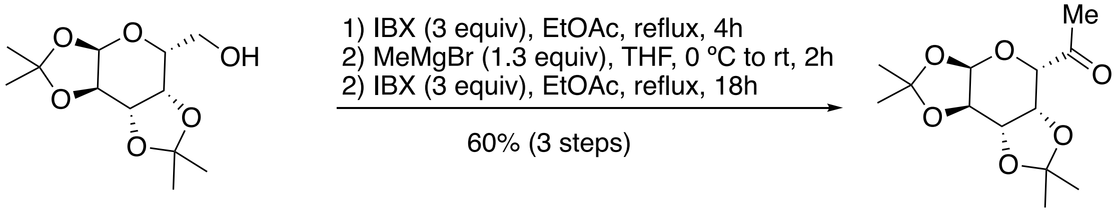

1zb

IBX (8.4 g, 40 mmol) was carefully added to a stirred solution of 1,2:3,4-Di-O-isopropylidene-D-galactopyranose (2.6 g, 10 mmol, 1 equiv) in EtOAc (70 mL, 0.14 M) at rt, and the reaction was stirred at reflux for 4 h. Afterwards, the reaction was allowed to cool-down to rt, and filtered through celite plug and the residue was washed with EtOAc (20 mL). The volatiles were removed in vacuo to provide the desired aldehyde that was used in the next step without further purification. To a solution in THF (50 mL, 0.2 M) of the resulting crude under argon atmosphere, MeMgBr (4.3 mL (3 M in Et_2O), 13 mmol, 1.3 equiv) was added dropwise. The reaction was stirred for 30 min at 0 ºC and then 1 h at rt. Afterwards, the reaction was cool-down to 0 ºC and a saturated solution of NH_4Cl_{aq} (30 ml) was added, and stirred for 15 min. The reaction was extracted with EtOAc (3 x 30 mL), the combination of the organic phases was washed with a saturated solution of NH_4Cl_{aq} (30 ml), dried over $MgSO_4$ and the volatiles were removed under vacuo to provide the desired alcohol that was used in the next step without further purification. IBX (8.4 g, 30 mmol) was carefully added to a stirred solution of obtained alcohol in EtOAc (70 mL, 0.14 M) at rt, and the reaction was stirred at reflux for 18 h. Afterwards, the reaction was allowed to cool-down to rt and filtered through celite plug and the residue was washed with EtOAc (20 mL). The volatiles were removed in vacuo and crude product was directly purified by column chromatography using *n*-hexane:EtOAc (8:2) as the eluent, to provide **1zb** as a gummy colorless oil (1.63 g, 60% yield over 3 steps). **^{1}H NMR** (400 MHz, $CDCl_3$) δ 5.64 (d, J = 5.0 Hz, 1H), 4.63 (dd, J = 7.8, 2.5 Hz, 1H), 4.55 (dd, J = 7.8, 2.2 Hz, 1H), 4.35 (dd, J = 5.0, 2.5 Hz, 1H), 4.16 (d, J = 2.2 Hz, 1H), 2.25 (s, 3H), 1.50 (s, 3H), 1.44 (s, 3H), 1.33 (s3H), 1.31 (s, 3H). **^{13}C NMR** (101 MHz, $CDCl_3$) δ 207.8, 109.8, 109.1, 96.5, 74.0, 72.6, 70.8, 70.5, 28.1, 26.1, 26.0, 25.0, 24.4. **IR** (neat, cm^{-1}): 2990, 2935, 1740, 1455, 1373, 1254, 1209, 1062, 1005, 892, 774. **HRMS** (ESI^+) calcd. for $C_{13}H_{21}O_6$ $[M+H]^+$ 273.1333, found 273.1326.

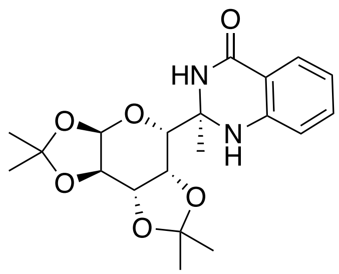

(*R*)-2-methyl-2-((3a*R*,5*R*,5a*S*,8a*S*,8b*R*)-2,2,7,7-tetramethyltetrahydro-5*H*-bis([1,3]dioxolo)[4,5-*b*:4',5'-*d*]pyran-5-yl)-2,3-dihydroquinazolin-4(1*H*)-one (2zd). 1zb (500 mg, 1.84 mmol, 1 equiv), 2-aminobenzamide (263 mg, 1.93 mmol, 1.05 mmol) and Cp_2TiCl_2 (9 mg, 0.036 mmol, 2 mol%) and EtOH (8 mL) were added to a 25 mL round-bottom flask equipped with a magnetic stirring bar and the reaction was heated to 80 ºC for 4 h. Afterwards the reaction as cooled to rt and the volatile were removed in vacuo. Then, water (20 mL) was added, and the suspension was filtered to provide **2zd** as a white-off solid (500 mg, 70%, melting point: 190 °C). **^{1}H NMR** (500 MHz, DMSO) δ 8.08 (d, *J* = 1.7 Hz, 1H), 7.52 (dd, *J* = 7.7, 1.6 Hz, 1H), 7.23 – 7.14 (m, 1H), 6.73 – 6.65 (m, 2H), 6.63 – 6.55 (m, 1H), 5.44 (d, *J* = 4.9 Hz, 1H), 4.57 (dd, *J* = 8.0, 2.1 Hz, 1H), 4.38 (dd, *J* = 8.0, 1.5 Hz, 1H), 4.23 (dd, *J* = 5.0, 2.1 Hz, 1H), 3.87 (d, *J* = 1.5 Hz, 1H), 1.47 (s, 3H), 1.37 (s, 3H), 1.28 (s, 3H), 1.14 (s, 3H), 0.70 (s, 3H). **^{13}C NMR (**126 MHz, DMSO) δ 162.9, 146.7, 133.1, 126.9, 116.5, 114.6, 114.1, 108.5, 107.6, 96.2, 70.8, 70.3, 69.7, 69.6, 68.9, 25.9, 24.8, 24.7, 24.1, 23.1. **IR** (neat, cm^{-1}): 3369, 2985, 2935, 1664, 1614, 1485, 1375, 1206, 1063, 996, 751. **HRMS** (ESI^+) calcd. for $C_{20}H_{27}N_2O_6$ $[M+H]^+$ 391.1864, found 391.1870.

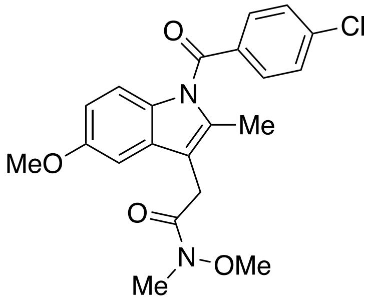

2-(1-(4-chlorobenzoyl)-5-methoxy-2-methyl-1*H*-indol-3-yl)-*N*-methoxy-*N*-methylacetamide (S1zc). Following GP6, using Indomethacin (3.58 g, 10 mmol, 1 equiv), N,O-dimethylhydroxylamine hydrochloride (1.17 g, 12 mmol, 1.2 equiv) *N*-(3-dimethylaminopropyl)-*N'*-ethylcarbodiimide hydrochloride (2.3 g, 12 mmol, 1.2 equiv), 4-(dimethylamino)pyridine (12 mg, 0.1 mmol, 5 mol%) and *N*,*N*- diisopropylethylamine (2.61 mL, 15 mmol, 1.2 equiv) and DCM (50 mL). Flash column chromatography using *n*-hexanes:EtOAc (7:3) provided **S1zc** (3.1 g, 92%

yield) as an white solid. **^{1}H NMR** (500 MHz, $CDCl_3$) δ 7.68 – 7.64 (m, 2H), 7.48 – 7.43 (m, 2H), 7.04 (d, *J* = 2.5 Hz, 1H), 6.84 (d, *J* = 9.0 Hz, 1H), 6.64 (dd, *J* = 9.0, 2.5 Hz, 1H), 3.82 (s, 3H), 3.80 (s, 2H), 3.69 (s, 3H), 3.21 (s, 3H), 2.38 (s, 3H). ^{13}C NMR (126 MHz, $CDCl_3$) δ 171.6, 168.4, 156.1, 139.2, 135.9, 134.1, 131.3, 131.1, 130.1, 129.2, 114.9, 113.3, 111.6, 101.8, 61.4, 55.8, 32.5, 28.7, 13.6. **IR** (neat, cm^{-1}): 3074, 2967, 2933, 2850, 1677, 1659, 1585, 1220, 1152, 1171, 1070, 1000, 926, 851, 756, 693, 601. **HRMS** (ESI$^+$) calcd. for $C_{21}H_{22}ClN_2O_4$ [M+H]$^+$ 401.1265, found 401.1263.

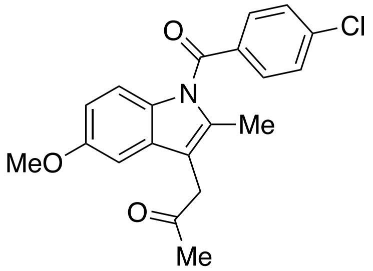

1-(1-(4-chlorobenzoyl)-5-methoxy-2-methyl-1*H*-indol-3-yl)propan-2-one (1zc). Following GP7, using **S1zc** (3.1 g, 7.7 mmol, 1 equiv), MeMgBr (3.35 mL, 10.1 mmol, 1.3 equiv) and THF (39 mL). Flash column chromatography using *n*-hexane: EtOAc (7:3) provided **1zc** (2.5 g, 91% yield, melting point: 76ºC) as a white-off solid. **^{1}H NMR** (400 MHz, $CDCl_3$) δ 7.69 – 7.64 (m, 2H), 7.50 – 7.45 (m, 2H), 6.88 – 6.84 (m, 2H), 6.67 (dd, *J* = 9.1, 2.5 Hz, 1H), 3.82 (s, 3H), 3.71 (s, 2H), 2.38 (s, 3H), 2.18 (s, 3H). ^{13}C NMR (101 MHz, $CDCl_3$) δ 205.8, 168.4, 156.3, 139.5, 136.0, 134.0, 131.3, 131.0, 130.8, 129.3, 115.2, 112.9, 111.8, 101.3, 55.9, 40.0, 29.2, 13.5. **IR** (neat, cm^{-1}): 3068, 2930, 2831, 1707, 1676, 1596, 1464, 1351, 1328, 1233, 1044, 1030, 849, 753. **HRMS** (ESI$^+$) calcd. for $C_{20}H_{18}ClNO_3$ [M+H]$^+$ 356.1048, found 356.1046.

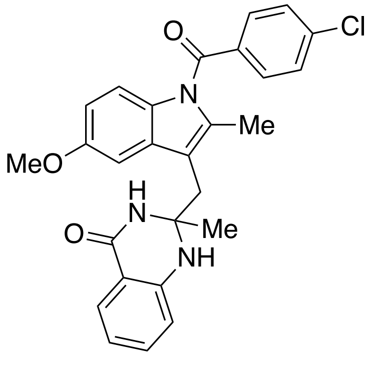

2-((1-(4-chlorobenzoyl)-5-methoxy-2-methyl-1*H*-indol-3-yl)methyl)-2-methyl-2,3-dihydroquinazolin-4(1*H*)-one (2ze). 1zc (1.0 g, 1.84 mmol, 1 equiv), 2-aminobenzamide (402 mg, 1.93 mmol, 1.05 mmol) and Cp_2TiCl_2 (14 mg, 0.036 mmol, 2 mol%) and EtOH (11.5 mL) were added to a 25 mL round-bottom flask equipped with a magnetic stirring bar and the reaction

was heated to 80 ºC for 4 h. Afterwards the reaction as cooled-down to rt and the volatiles were removed in vacuo. Then, water (20 mL) was added, and the suspension was filtered to provide **2ze** as a white-off solid (1.00 g, 80% yield, melting point: 201 ºC). ^{1}**H NMR** (500 MHz, DMSO-*d*$_6$) δ 7.89 (s, 1H), 7.62 (d, *J* = 8.2 Hz, 2H), 7.50 (d, *J* = 8.2 Hz, 2H), 7.39 – 7.32 (m, 1H), 7.08 (d, *J* = 2.5 Hz, 1H), 7.06 – 6.99 (m, 1H), 6.89 (d, *J* = 8.9 Hz, 1H), 6.78 (s, 1H), 6.60 (dd, *J* = 9.0, 2.5 Hz, 1H), 6.48 – 6.38 (m, 2H), 3.79 (s, 3H), 3.00 – 2.84 (m, 2H), 2.07 (s, 3H), 1.55 (s, 3H). ^{13}**C NMR** (126 MHz, DMSO-*d*$_6$) δ 167.7, 162.6, 155.5, 146.6, 137.6, 135.2, 134.0, 132.6, 131.5, 131.3, 130.3, 128.9, 126.3, 115.5, 114.8, 114.1, 113.0, 112.9, 111.3, 102.1, 70.8, 55.34, 37. 5, 29.0, 13.9. **IR** (neat, cm^{-1}): 3398, 3192, 2927, 1688, 1611, 1480, 1372, 1320, 1148, 1064, 764. **HRMS** (ESI$^+$) calcd. for $C_{27}H_{25}ClN_3O_3$ [M+H]$^+$ 474.1579, found 474.1585.

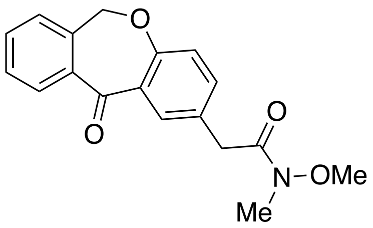

***N*-methoxy-*N*-methyl-2-(11-oxo-6,11-dihydrodibenzo[*b*,*e*]oxepin-2-yl)acetamide (S1ze).** Following GP6, using Isoxepac (2.68 g, 10 mmol, 1 equiv), *N*,O-dimethylhydroxylamine hydrochloride (1.17 g, 12 mmol, 1.2 equiv) *N*-(3-dimethylaminopropyl)-*N'*-ethylcarbodiimide hydrochloride (2.3 g, 12mmol, 1.2 equiv), 4-(dimethylamino)pyridine (12 mg, 0.1 mmol, 5 mol%) and *N*,*N*- diisopropylethylamine (2.61 mL, 15 mmol, 1.2 equiv) and DCM (50 mL). Flash column chromatography using *n*-hexane:EtOAc (gradient 7:3 to 2:3) provided **S1zd** (2.9 g, 94 % yield) as a yellow oil. ^{1}**H NMR** (500 MHz, $CDCl_3$) δ 8.10 (d, *J* = 2.4 Hz, 1H), 7.89 (dd, *J* = 7.7, 1.4 Hz, 1H), 7.55 (td, *J* = 7.4, 1.3 Hz, 1H), 7.46 (ddd, *J* = 8.4, 4.9, 1.9 Hz, 2H), 7.35 (dd, *J* = 7.4, 1.3 Hz, 1H), 7.02 (d, *J* = 8.4 Hz, 1H), 5.18 (s, 2H), 3.79 (s, 2H), 3.70 (s, 3H), 3.20 (s, 3H). ^{13}**C NMR** (126 MHz, $CDCl_3$) δ 190.8, 172.1, 171.1, 160.3, 140.4, 136.6, 135.7, 132.7, 129.4, 129.2, 127.8, 125.1, 120.9, 77.4, 73.6, 61.4, 38.0, 32.3. **IR** (neat, cm^{-1}): 2965, 2933, 2902, 1660, 1638, 1511, 1488, 1455, 1380, 1131, 1006, 767. **HRMS** (ESI$^+$) calcd. for $C_{18}H_{18}NO_4$ [M+H]$^+$ 312.1230, found 312.1219.

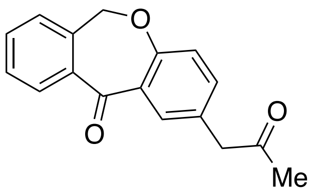

2-(2-oxopropyl)dibenzo[*b*,*e*]oxepin-11(6*H*)-one (1zd). Following GP7, using **S1zd** (2.90 g, 9.4 mmol, 1 equiv), MeMgBr (4.10 mL, 12.2. mmol, 1.3 equiv) and THF (47 mL). Flash column chromatography using *n*-hexane: EtOAc (3:1) provided **1zd** (2.2 g, 89% yield) as brown oil. **^{1}H NMR** (500 MHz, CDCl3) δ 8.04 (d, *J* = 2.4 Hz, 1H), 7.89 (dd, *J* = 7.7, 1.4 Hz, 1H), 7.56 (td, *J* = 7.4, 1.3 Hz, 1H), 7.47 (td, J = 7.6, 1.3 Hz, 1H), 7.36 (dd, J = 7.5, 1.3 Hz, 1H), 7.32 (dd, J = 8.4, 2.4 Hz, 1H), 7.03 (d, J = 8.4 Hz, 1H), 5.19 (s, 2H), 3.73 (d, J = 6.3 Hz, 2H), 2.19 (d, J = 5.8 Hz, 3H). **^{13}C NMR** (126 MHz, $CDCl_3$) δ 206.1, 191.0, 160.6, 140.6, 136.6, 135.7, 132.9, 132.7, 129.6, 129.4, 128.0, 125.4, 121.3, 73.8, 49.6, 29.8. **IR** (neat, cm^{-1}): 3478, 3012, 2970, 2925, 1708, 1610, 1454, 1412, 1356, 1284, 1120, 1014, 912, 833, 753. **HRMS** (ESI^+) calcd. for $C_{17}H_{14}NaO_3$ $[M+Na]^+$ 289.0835, found 289.0845.

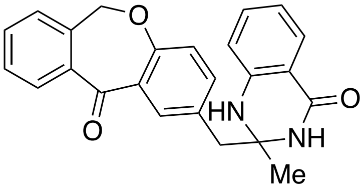

2-methyl-2-((11-oxo-6,11-dihydrodibenzo[*b*,*e*]oxepin-2-yl)methyl)-2,3-dihydroquinazolin-4(1*H*)-one (2zf). 1zd (2.50 g, 9.39 mmol, 1 equiv), 2-aminobenzamide (1.30 g, 9.86 mmol, 1.05 equiv) and Cp_2TiCl_2 (23 mg, 0.094 mmol, 2 mol%) and EtOH (47.5 mL) were added to a 100 mL round-bottom flask equipped with a magnetic stirring bar and the reaction was heated to 80 ºC for 4 h. Afterwards the reaction as cooled-down to rt and the volatiles were removed in vacuo. Then, water (20 mL) was added and the suspension was filtered to provide **2zf** as a brown solid. The crude reaction was used in the subsequent step without further purification following the one-pot labeling procedure (GP4).

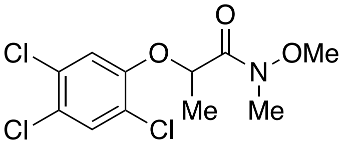

2-(3,4-dichlorophenoxy)-*N*-methoxy-*N*-methylpropanamide (S1ze). Following GP6, using 2-(2,4,5-trichlorophenoxy)propanoic acid (2.70 g, 10 mmol, 1 equiv), *N*,O-dimethylhydroxylamine hydrochloride (1.17 g, 12 mmol, 1.2 equiv) *N*-(3-dimethylaminopropyl)-*N'*-ethylcarbodiimide

hydrochloride (2.30 g, 12mmol, 1.2 equiv), 4-(dimethylamino)pyridine (12 mg, 0.1 mmol, 5 mol%) and *N*,*N*- diisopropylethylamine (2.61 mL, 15 mmol, 1.2 equiv) and DCM (50 mL). Flash column chromatography using n-hexanes:EtOAc 3:1 provided **S1ze** as a white solid (2.8 g, 90% yield, melting point: 82 ºC). **^{1}H NMR** (500 MHz, $CDCl_3$) δ 7.46 (s, 1H), 6.95 (s, 1H), 5.09 (d, *J* = 6.8 Hz, 1H), 3.75 (s, 3H), 3.23 (s, 3H), 1.63 (d, *J* = 6.6 Hz, 3H). **^{13}C NMR** (126 MHz, $CDCl_3$) δ 171.3, 152.6, 131.3, 125.4, 123.2, 116.9, 61.8, 60.5, 21.2, 17.7, 14.3. **IR** (neat, cm^{-1}): 3096, 3071, 2996, 2979, 2937, 2171, 1676, 1580, 1460, 1079, 984, 678. **HRMS** (ESI$^+$) calcd. for $C_{11}H_{13}Cl_3NO_3$ $[M+H]^+$ 311.9956, found 311.9949.

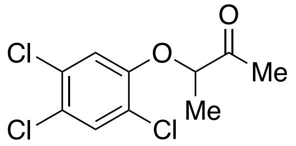

3-(3,4-dichlorophenoxy)butan-2-one (1ze). Following GP7, using **S1ze** (2.70 g, 8.6 mmol, 1 equiv), MeMgBr (3.74 mL, 11.2 mmol, 1.3 equiv) and THF (43 mL). Flash column chromatography using n-hexanes: EtOAc (6:1) provided **1ze** (2.0 g, 85% yield) as a colorless oil. **^{1}H NMR** (500 MHz, $CDCl_3$) δ 7.49 (s, 1H), 6.87 (s, 1H), 4.68-4.40 (m, 1H), 2.25 (s, 3H), 1.54 (d, *J* = 6.8 Hz, 3H). **^{13}C NMR** (126 MHz, $CDCl_3$) δ 171.3, 152.6, 131.3, 125.4, 123.2, 116.9, 61.8, 60.5, 21.2, 17.7, 14.3. **IR** (neat, cm^{-1}): 3097, 2988, 2925, 2854, 1721, 1584, 1459, 1280, 1134, 1045, 992, 937, 874, 595. **HRMS** (ESI$^+$) calcd. for $C_{10}H_9Cl_3NaO_2$ $[M+Na]^+$ 288.9560, found 288.9559.

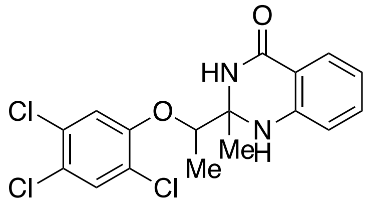

2-(1-(3,4-dichlorophenoxy)ethyl)-2-methyl-2,3-dihydroquinazolin-4(1*H*)-one (2zf). 2-(1-(3,4-dichlorophenoxy)ethyl)-2-methyl-2,3-dihydroquinazolin-4(1*H*)-one (2zg). 1ze (1.90 g, 7.10 mmol, 1 equiv), 2-aminobenzamide (1.02 g, 7.46 mmol, 1.05 equiv) and Cp_2TiCl_2 (18 mg, 0.071 mmol, 2 mol%) and EtOH (36 mL) were added to a 100 mL round-bottom flask equipped with a magnetic stirring bar and the reaction was heated to 80 ºC for 4 h. Afterwards the reaction as cooled to rt and the volatile were removed in vacuo. Then, water (20 mL) was added, and the suspension was filtered to provide **2zg** as a brown solid (2.10 g, 77% yield, melting point: 129 ºC). **^{1}H NMR** (500 MHz, $CDCl_3$): The compound exists as a rotamers mixture in 1.5:1 ratio. *Signals for major*

rotamer: δ 7.89 (td, *J* = 8.1, 1.6 Hz, 1H), 7.48 (s, 1H), 7.35 (ddd, *J* = 8.0, 7.3, 1.6 Hz, 1H), 6.98 (s, 1H), 6.85 (tdd, *J* = 6.9, 5.7, 1.0 Hz, 1H)j, 6.67 (dd, *J* = 8.1, 1.0 Hz, 1H), 6.64 (bs, 1H), 4.47 (q, *J* = 6.3 Hz, 1H), 4.45 (d, *J* = 2.2 Hz, 1H), 1.69 (s, 3H), 1.39 (d, *J* = 6.2 Hz, 3H). *Representative signals for minor rotamer*: δ 7.49 (s, 1H), 7.28-7.25 (m, 1H), 6.81 (s, 1H), 6.76 (s, 1H), 6.45 (dd, *J* = 8.1, 1.0 Hz, 1H), 4.68 (s, 1H), 4.55 (q, *J* = 6.3 Hz, 1H), 1.42 (d, *J* = 6.3 Hz, 3H). **^{13}C-NMR** (500 MHz, CDCl3): *Signals for major rotamer*: δ 151.9, 145.2, 134.4, 131.4, 131.2, 128.5, 125.3, 123.2, 119.0, 116.7, 114.2, 80.0, 79.4, 71.5, 24.2, 22.1, 14.0. *Representative signals for minor rotamer*: δ.152.3, 134.5, 131.5, 131.0, 128.3, 125.4, 123.1, 119.2, 117.3, 114.4, 71.3, 14.1. **IR** (neat, cm^{-1}): 3402, 2964, 2933, 1659, 1639, 1612, 1595, 1486, 1380, 1217, 1093, 764, 704, 628. **HRMS** (ESI$^+$) calcd. for $C_{17}H_{16}Cl_3N_2O_2$ [M+H]$^+$ 385.0272, found 385.0279.

5. ^{12}C/^{13}C Exchange via Dual Catalytic C-C Bond-Functionalization

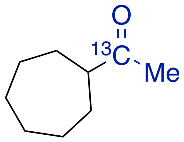

1-Cycloheptylethan-1-one-1-^{13}*C* ([13C]1a). Following GP2, 2-cycloheptyl-2-methyl-2,3-dihydroquinazolin-4(1*H*)-one (51.6 mg, 0.20 mmol) and acetyl-1-^{13}*C* chloride (23.1 mg, 0.30 mmol) were used, affording the title compound as a colorless oil (20.0 mg, 71% yield), by using hexanes/Et_2O (10:1) as chromatography eluent. In a second independent experiment, 20.8 mg (74%) were obtained, giving an average yield of 72%. **^{1}H NMR** (400 MHz, $CDCl_3$) δ 2.58 – 2.45 (m, 1H), 2.12 (d, *J* = 5.6 Hz, 3H), 1.91 – 1.81 (m, 2H), 1.70 (dtd, *J* = 13.7, 6.7, 3.1 Hz, 2H), 1.63 – 1.44 (m, 8H). **^{13}C NMR** (101 MHz, $CDCl_3$) δ 212.7, 53.3 (d, *J* = 39.1 Hz), 29.9 (d, *J* = 1.1 Hz), 28.4, 28.0 (d, *J* = 39.4 Hz), 26.8 (d, *J* = 4.0 Hz). **IR** (neat, cm^{-1}): 2922, 2855, 1668, 1460, 1351, 1144, 946. **HRMS** (ESI$^+$) calcd. for $C_8{}^{13}CH_{16}ONa$ [M+Na]$^+$ 164.1127, found 164.1129.

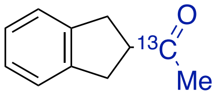

1-(2,3-Dihydro-1*H*-inden-2-yl)ethan-1-one-1-^{13}*C* ([13C]1b). Following GP2, 2-(2,3-dihydro-1*H*-inden-2-yl)-2-methyl-2,3-dihydroquinazolin-4(1*H*)-one (55.6 mg, 0.20 mmol) and acetyl-1-^{13}*C* chloride (23.1 mg, 0.30 mmol) were used, affording the title compound as a colorless oil (22.5 mg, 70% yield), by using hexanes/Et_2O (10:1) as chromatography eluent. In a second independent

experiment, 22.7 mg (70%) were obtained, giving an average yield of 70%. ^{1}**H NMR** (400 MHz, $CDCl_3$) δ 7.22 – 7.14 (m, 4H), 3.44 (ddd, J = 12.7, 8.2, 4.1 Hz, 1H), 3.22 – 3.12 (m, 4H), 2.24 (d, J = 5.7 Hz, 3H). ^{13}**C NMR** (101 MHz, $CDCl_3$) δ 209.3, 141.5, 126.7, 124.4, 51.9 (d, J = 40.7 Hz), 35.0, 28.5 (d, J = 40.6 Hz). **IR** (neat, cm^{-1}): 2955, 2924, 2854, 2801, 1655, 1459, 1260, 1097, 1024. **HRMS** (ESI^+) calcd. for $C_{10}{}^{13}CH_{12}ONa$ $[M+Na]^+$ 184.0814, found 184.0817.

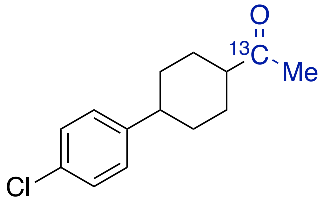

1-(4-(4-Chlorophenyl)cyclohexyl)ethan-1-one-1-^{13}*C* ([13C]1c). Following GP2, 2-(4-(4-chlorophenyl)cyclohexyl)-2-methyl-2,3-dihydroquinazolin-4(1*H*)-one (70.8 mg, 0.20 mmol) and acetyl-1-^{13}*C* chloride (23.1 mg, 0.30 mmol) were used, affording the title compound as a yellow solid (36.5 mg, 77% yield, melting point: 72 °C), by using hexanes/EtOAc (20:1) as chromatography eluent. In a second independent experiment, 36.9 mg (77%) were obtained, giving an average yield of 76%. ^{1}**H NMR** (400 MHz, $CDCl_3$) δ 7.26 (d, J = 8.4 Hz, 2H), 7.12 (d, J = 8.4 Hz, 2H), 2.52 – 2.34 (m, 2H), 2.18 (d, J = 5.7 Hz, 3H), 2.08 – 1.93 (m, 4H), 1.53 – 1.40 (m, 4H). ^{13}**C NMR** (101 MHz, $CDCl_3$) δ 211.9, 145.4, 131.8, 128.6, 128.2, 51.0 (d, J = 40.2 Hz), 43.2, 33.5 (d, J = 4.1 Hz), 28.7 (d, J = 1.4 Hz), 28.2 (d, J = 39.7 Hz). **IR** (neat, cm^{-1}): 2930, 2853, 1655, 1491, 1449, 1353, 1158, 1090, 1012, 820, 606, 529. **HRMS** (ESI^+) calcd. for $C_{13}{}^{13}CH_{17}ClONa$ $[M+Na]^+$ 260.0894, found 260.0901.

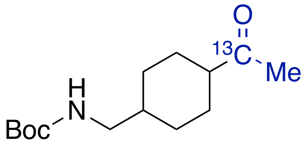

***tert*-Butyl ((4-(acetyl-1-^{13}*C*)cyclohexyl)methyl)carbamate ([13C]1d).** Following GP2, *tert*-butyl (4-(2-methyl-4-oxo-1,2,3,4-tetrahydroquinazolin-2-yl)cyclohexyl)carbamate (71.8 mg, 0.20 mmol) and acetyl-1-^{13}*C* chloride (23.1 mg, 0.30 mmol) were used, affording the title compound as a yellow solid (34.3 mg, 67% yield, melting point: 66 °C) by using hexanes/EtOAc (2:1) as chromatography eluent. In a second independent experiment, 34.8 mg (68%) were obtained, giving an average yield of 67%. ^{1}**H NMR** (400 MHz, $CDCl_3$) δ 4.59 (s, 1H), 3.00 (t, J = 6.5 Hz, 2H), 2.30 (dddd, J = 12.1, 8.6, 5.0, 2.7 Hz, 1H), 2.15 (d, J = 5.7 Hz, 3H), 1.99 – 1.83 (m, 4H), 1.46 (s,

9H), 1.42 – 1.22 (m, 3H), 0.99 (qd, J = 13.0, 3.4 Hz, 2H). **^{13}C NMR** (101 MHz, $CDCl_3$) δ 211.9, 156.1, 79.1, 51.4 (d, J = 40.1 Hz), 46.5, 37.9, 29.8 (d, J = 3.9 Hz), 28.4, 28.2, 27.8. **IR** (neat, cm^{-1}): 3366, 2975, 2928, 2856, 1699, 1665, 1521, 1451, 1391, 1365, 1248, 1170. **HRMS** (ESI$^+$) calcd. for $C_{13}{}^{13}CH_{25}NNaO_3$ [M+Na]$^+$ 279.1766, found 279.1765.

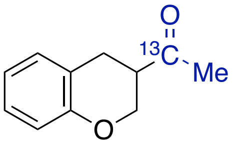

1-(Chroman-3-yl)ethan-1-one-1-^{13}*C* ([13C]1e). Following GP2, 2-(chroman-3-yl)-2-methyl-2,3-dihydroquinazolin-4(1*H*)-one (58.8 mg, 0.20 mmol) and acetyl-1-^{13}*C* chloride (23.1 mg, 0.30 mmol) were used, affording the title compound as a colorless oil (23.4 mg, 66% yield), by using hexanes/Et_2O (10:1) as chromatography eluent. In a second independent experiment, 24.9 mg (70%) were obtained, giving an average yield of 68%. **^{1}H NMR** (400 MHz, $CDCl_3$) δ 7.17 – 7.04 (m, 2H), 6.88 (td, J = 7.6, 1.2 Hz, 1H), 6.82 (d, J = 7.9 Hz, 1H), 4.48 – 4.40 (m, 1H), 4.14 – 4.02 (m, 1H), 3.14 – 2.89 (m, 3H), 2.29 (d, J = 5.8 Hz, 3H). **^{13}C NMR** (101 MHz, $CDCl_3$) δ 207.9, 154.2, 137.9, 129.9, 127.7, 120.9, 116.8, 66.5, 46.0 (d, J = 39.8 Hz), 28.9 (d, J = 40.8 Hz), 27.4. **IR** (neat, cm^{-1}): 3059, 2922, 2853, 1667, 1583, 1481, 1439, 1354, 1201, 1133, 740, 690. **HRMS** (ESI$^+$) calcd. for $C_{10}{}^{13}CH_{12}O_2Na$ [M+Na]$^+$ 200.0763, found 200.0765.

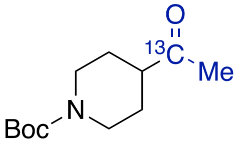

***tert*-Butyl 4-(acetyl-1-^{13}*C*)piperidine-1-carboxylate ([13C]1f).** Following GP2, *tert*-butyl 4-(2-methyl-4-oxo-1,2,3,4-tetrahydroquinazolin-2-yl)piperidine-1-carboxylate (69.0 mg, 0.20 mmol) and acetyl-1-^{13}*C* chloride (23.1 mg, 0.30 mmol) were used, affording the title compound as a colourless oil (34.2 mg, 75% yield), by using hexanes/Et_2O (1:2) as chromatography eluent. In a second independent experiment, 36.0 mg (79%) were obtained, giving an average yield of 77%.

One-pot labeling: following GP4, 2-aminobenzamide (29 mg, 0.21 mmol) and *tert*-butyl 4-acetylpiperidine-1-carboxylate (45 mg, 0.20 mmol) were used followed by acetyl-1-^{13}*C* chloride (24 mg, 0.30 mmol) affording the title compound as a colorless oil (22.0 mg, 48% yield), by using hexanes/Et_2O (10:1) as chromatography eluent. In a second independent experiment, 22.3 mg (48%) were obtained, giving an average yield of 77%. **^{1}H NMR** (400 MHz, $CDCl_3$) δ 4.09 (brs,

2H), 2.78 (t, J = 12.6 Hz, 2H), 2.49 – 1.40 (m, 1H), 2.16 (d, J = 5.7 Hz, 3H), 1.83 (d, J = 13.3 Hz, 2H), 1.57 – 1.49 (m, 2H), 1.45 (s, 9H). **^{13}C NMR** (101 MHz, $CDCl_3$) δ 210.2, 154.7, 79.6, 49.2 (d, J = 40.4 Hz), 43.2, 28.4, 27.8 (d, J = 40.1 Hz), 27.4. **IR** (neat, cm^{-1}): 2975, 2930, 2856, 1687, 1666, 1419, 1365, 1305, 1277, 1235, 1150, 1123, 1030, 957, 862, 769. **HRMS** (ESI$^+$) calcd. for $C_{11}{}^{13}CH_{21}NO_3Na$ [M+Na]$^+$ 251.1447, found 251.1450.

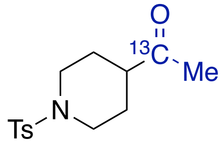

1-(1-Tosylpiperidin-4-yl)ethan-1-one-1-^{13}*C* ([13C]1g). Following GP2, 2-methyl-2-(1-tosylpiperidin-4-yl)-2,3-dihydroquinazolin-4(1*H*)-one (79.8 mg, 0.20 mmol) and acetyl-1-^{13}*C* chloride (23.1 mg, 0.30 mmol) were used, affording the title compound as a colorless oil (31.6 mg, 56% yield), by using hexanes/Et_2O (1:2) as chromatography eluent. In a second independent experiment, 32.1 mg (57%) were obtained, giving an average yield of 56%. **^{1}H NMR** (400 MHz, $CDCl_3$) δ 7.63 (d, J = 8.3 Hz, 2H), 7.32 (d, J = 8.0 Hz, 2H), 3.69 (dt, J = 11.0, 3.7 Hz, 2H), 2.47 – 2.38 (m, 5H), 2.24 (ddt, J = 15.0, 11.0, 4.5 Hz, 1H), 2.11 (d, J = 5.7 Hz, 3H), 1.92 (dd, J = 13.3, 3.7 Hz, 2H), 1.72 (dddd, J = 15.4, 11.2, 6.6, 2.8 Hz, 2H). **^{13}C NMR** (101 MHz, $CDCl_3$) δ 209.4, 143.8, 133.3, 129.8, 127.8, 48.1 (d, J = 40.3 Hz), 45.6 (d, J = 3.8 Hz), 27.9 (d, J = 40.3 Hz), 27.0 (d, J = 1.1 Hz), 21.7. **IR** (neat, cm^{-1}): 2926, 2852, 1667, 1597, 1447, 1351, 1335, 1305, 1250, 1162, 1093, 1053, 927, 817, 725, 650, 611, 580, 549. **HRMS** (ESI$^+$) calcd. for $C_{13}{}^{13}CH_{19}NO_3SNa$ [M+Na]$^+$ 305.1011, found 305.1019.

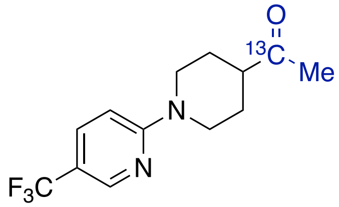

1-(1-(5-(Trifluoromethyl)pyridin-2-yl)piperidin-4-yl)ethan-1-one-1-^{13}*C* ([13C]1h). Following GP2, 2-methyl-2-(1-(5-(trifluoromethyl)pyridin-2-yl)piperidin-4-yl)-2,3-dihydroquinazolin-4(1*H*)-one (78.0 mg, 0.20 mmol) and acetyl-1-^{13}*C* chloride (23.1 mg, 0.30 mmol) were used, affording the title compound as a yellow solid (30.0 mg, 55% yield, melting point: 57 °C), by using hexanes/Et_2O (1:2) as chromatography eluent. In a second independent experiment, 32.2 mg (59%) were obtained, giving an average yield of 57%. **^{1}H NMR** (400 MHz, $CDCl_3$) δ 8.38 (s, 1H), 7.61 (dd, J = 9.1, 2.6 Hz, 1H), 6.65 (d, J = 9.0 Hz, 1H), 4.39 (dt, J = 13.5, 3.6 Hz, 2H), 3.02 (ddd, J = 13.7, 11.8, 2.9 Hz, 2H), 2.61 (ddt, J = 15.4, 11.3, 4.2 Hz, 1H), 2.19 (d, J = 5.7 Hz, 3H), 2.04 – 1.84

(m, 2H), 1.72 – 1.58 (m, 2H). **^{13}C NMR** (101 MHz, $CDCl_3$) δ 210.1, 160.3, 145.9 (q, J = 4.4 Hz, 134.6 (q, J = 2.9 Hz), 124.8 (q, J = 270.4 Hz), 105.8, 49.3 (d, J = 40.3 Hz), 44.6 (d, J = 3.7 Hz), 28.0 (d, J = 40.3 Hz), 27.2 (d, J = 1.4 Hz). **^{19}F NMR** (376 MHz, $CDCl_3$) δ -61.3. **IR** (neat, cm^{-1}): 2927, 2851, 1669, 1613, 1559, 1513, 1425, 1329, 1316, 1284, 1232, 1154, 1110, 1080, 1029, 937, 814. **HRMS** (ESI^+) calcd. for $C_{12}{}^{13}CH_{16}F_3N_2O$ $[M+H]^+$ 274.1243, found 274.1245.

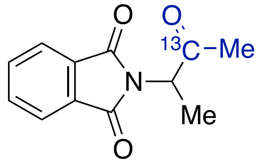

2-(3-oxobutan-2-yl-3-^{13}*C*)isoindoline-1,3-dione ([13C]1i). Following GP2, 2-(1-(2-methyl-4-oxo-1,2,3,4-tetrahydroquinazolin-2-yl)ethyl)isoindoline-1,3-dione (67.0 mg, 0.20 mmol) and acetyl-1-^{13}*C* chloride (23.1 mg, 0.30 mmol) were used, affording the title compound as a white solid (24.4 mg, 56% yield), by using hexanes/Et_2O (2:1) as chromatography eluent. In a second independent experiment, 26.1 mg (60%) were obtained, giving an average yield of 58%. **^{1}H NMR** (400 MHz, $CDCl_3$) δ 7.86 (dd, J = 5.6, 3.0 Hz, 2H), 7.74 (dd, J = 5.5, 3.0 Hz, 2H), 4.80 (qd, J = 7.3, 5.8 Hz, 1H), 2.20 (d, J = 6.0 Hz, 3H), 1.66 (dd, J = 7.3, 4.0 Hz, 3H). **^{13}C NMR** (101 MHz, $CDCl_3$) δ 203.2, 167.8, 134.4, 132.0, 123.7, 54.9 (d, J = 40.4 Hz), 26.5 (d, J = 42.4 Hz), 14.5. **IR** (neat, cm^{-1}): 2994, 2928, 1776, 1709, 1685, 1612, 1468, 1208, 1179, 1136, 1073, 1027, 879, 722, 531. **HRMS** (ESI^+) calcd. for $C_{11}{}^{13}CH_{12}NO_3$ $[M+H]^+$ 219.0845, found 219.0853.

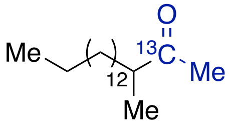

3-Methylheptadecan-2-one-2-^{13}*C* ([13C]1j). Following GP2, 2-(Hexadecan-2-yl)-2-methyl-2,3-dihydroquinazolin-4(1H)-one (77.3 mg, 0.20 mmol) and acetyl-1-^{13}*C* chloride (23.1 mg, 0.30 mmol) were used, affording the title compound as a colorless oil (39.8 mg, 74% yield), by using hexanes/Et_2O (10:1) as chromatography eluent. In a second independent experiment, 40.0 mg (74%) were obtained, giving an average yield of 74%. **^{1}H NMR** (400 MHz, $CDCl_3$) δ 2.63 – 2.38 (m, 1H), 2.12 (d, J = 5.6 Hz, 3H), 1.25 (s, 26H), 1.07 (dd, J = 6.9, 4.6 Hz, 3H), 0.90 – 0.85 (m, 3H). **^{13}C NMR** (101 MHz, $CDCl_3$) δ 213.2, 47.4 (d, J = 39.3 Hz), 33.1 (d, J = 1.4 Hz), 32.1, 29.8, 29.8, 29.8, 29.8, 29.7, 29.6, 29.5, 28.3, 27.9, 27.4 (d, J = 2.3 Hz), 22.8, 16.3 (d, J = 1.5 Hz), 14.3. **IR** (neat, cm^{-1}): 2922, 2853, 1673, 1460, 1353, 721. **HRMS** (ESI^+) calcd. for $C_{17}{}^{13}CH_{37}O$ $[M+H]^+$ 270.2872, found 270.2866.

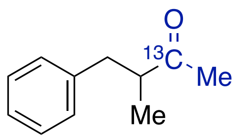

3-Methyl-4-phenylbutan-2-one-2-^{13}*C* ([13C]1k). Following GP2, 2-methyl-2-(1-phenylpropan-2-yl)-2,3-dihydroquinazolin-4(1*H*)-one (56.0 mg, 0.20 mmol) and acetyl-1-^{13}*C* chloride (23.1 mg, 0.30 mmol) were used, affording the title compound as a colorless oil (22.8 mg, 70% yield), by using hexanes/Et_2O (10:1) as chromatography eluent. In a second independent experiment, 23.8 mg (73%) were obtained, giving an average yield of 72%. **^{1}H NMR** (400 MHz, $CDCl_3$) δ 7.3 – 7.2 (m, 2H), 7.2 – 7.1 (m, 3H), 3.0 (ddd, *J* = 13.6, 6.8, 2.9 Hz, 1H), 2.8 (qd, *J* = 7.1, 4.6 Hz, 1H), 2.6 (ddd, *J* = 13.6, 7.7, 4.5 Hz, 1H), 2.1 (d, *J* = 5.9 Hz, 3H), 1.1 (dd, *J* = 7.0, 4.6 Hz, 3H). **^{13}C NMR** (101 MHz, $CDCl_3$) δ 212.3, 139.8 (d, *J* = 2.6 Hz), 129.1, 128.5, 126.4, 48.9 (d, *J* = 39.3 Hz), 39.0 (d, *J* = 1.4 Hz), 29.0 (d, *J* = 39.7 Hz), 16.4 (d, *J* = 1.5 Hz). **IR** (neat, cm^{-1}): 3028, 2969, 2930, 1713, 1671, 1603, 1454, 1358, 1153, 736, 700. **HRMS** (ESI$^+$) calcd. for $C_{10}{}^{13}CH_{14}ONa$ $[M+Na]^+$ 186.0970, found 186.0973.

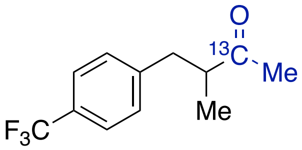

3-Methyl-4-(4-(trifluoromethyl)phenyl)butan-2-one-2-^{13}*C* ([13C]1l). Following GP2, 2-methyl-2-(1-(4-(trifluoromethyl)phenyl)propan-2-yl)-2,3-dihydroquinazolin-4(1*H*)-one (69.6 mg, 0.20 mmol) and acetyl-1-^{13}*C* chloride (23.1 mg, 0.30 mmol) were used, affording the title compound as a colorless oil (30.0 mg, 65% yield), by using hexanes/Et_2O (10:1) as chromatography eluent. In a second independent experiment, 30.9 mg (67%) were obtained, giving an average yield of 66%. **^{1}H NMR** (400 MHz, $CDCl_3$) δ 7.53 (d, *J* = 8.5 Hz, 2H), 7.27 (d, *J* = 8.0 Hz, 2H), 3.07 (ddd, *J* = 13.7, 7.0, 2.9 Hz, 1H), 2.84 (m, 1H), 2.69 – 2.55 (m, 1H), 2.11 (d, *J* = 5.7 Hz, 3H), 1.11 (dd, *J* = 7.0, 4.5 Hz, 3H). **^{13}C NMR** (101 MHz, $CDCl_3$) δ 211.5, 144.1 (q, *J* = 1.0 Hz), 128.8 (d, *J* = 32.6 Hz), 125.5 (q, *J* = 3.8 Hz), 124.4 (q, *J* = 272.7 Hz), 112.0, 48.6 (d, *J* = 39.2 Hz), 38.5, 28.9 (d, *J* = 40.0 Hz), 16.6 (d, *J* = 1.5 Hz). **^{19}F NMR** (376 MHz, $CDCl_3$) δ -62.52. **IR** (neat, cm^{-1}): 2930, 1672, 1619, 1323, 1162, 1114, 1067, 1019, 817, 600. **HRMS** (ESI$^+$) calcd. for $C_{11}{}^{13}CH_{13}F_3ONa$ $[M+Na]^+$ 254.0844, found 254.0850.

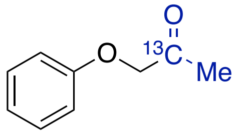

1-Phenoxypropan-2-one-2-^{13}*C* ([13C]1m). Following GP2, 2-methyl-2-(phenoxymethyl)-2,3-dihydroquinazolin-4(1*H*)-one (53.6 mg, 0.20 mmol) and acetyl-1-^{13}*C* chloride (18.7 mg, 0.30 mmol) were used, affording the title compound as a yellow oil (18.7 mg, 62% yield), by using hexanes/Et_2O (6:1) as chromatography eluent. In a second independent experiment, 18.4 mg (61%) were obtained, giving an average yield of 62%.

One-pot labeling: following GP4, 2-aminobenzamide (34 mg, 0.25 mmol) and 1-Phenoxy-propan-2-one (36 mg, 0.24 mmol) were used followed by acetyl-1-^{13}*C* chloride (29 mg, 0.36 mmol) affording the title compound as a colorless oil (14.8 mg, 41% yield), by using hexanes/Et_2O (10:1) as chromatography eluent. In a second independent experiment, 16.3 mg (45%) were obtained, giving an average yield of 43%. **^{1}H NMR** (400 MHz, $CDCl_3$) δ 7.39 – 7.27 (m, 2H), 7.00 (t, *J* = 7.4 Hz, 1H), 6.89 (d, *J* = 8.7 Hz, 2H), 4.54 (d, *J* = 4.0 Hz, 2H), 2.28 (d, *J* = 6.1 Hz, 3H). **^{13}C NMR** (101 MHz, $CDCl_3$) δ 206.1, 157.9 (d, *J* = 2.4 Hz), 129.8, 121.9, 114.7, 73.2 (d, *J* = 43.3 Hz), 26.7 (d, *J* = 41.4 Hz). **IR** (neat, cm^{-1}): 2923, 1691, 1598, 1589, 1494, 1432, 1294, 1226, 1152, 1064, 752, 691, 507. **HRMS** (ESI$^+$) calcd. for $C_8{}^{13}CH_{10}O_2Na$ $[M+Na]^+$ 174.0607, found 174.0613.

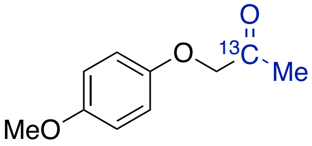

1-(4-Methoxyphenoxy)propan-2-one-2-^{13}*C* ([13C]1n). Following GP2, 2-((4-methoxyphenoxy)methyl)-2-methyl-2,3-dihydroquinazolin-4(1*H*)-one (59.6 mg, 0.20 mmol) and acetyl-1-^{13}*C* chloride (18.7 mg, 0.30 mmol) were used, affording the title compound as a colorless oil (23.9 mg, 66% yield), by using hexanes/Et_2O (5:1) as chromatography eluent. In a second independent experiment, 25.3 mg (70%) were obtained, giving an average yield of 68%. **^{1}H NMR** (400 MHz, $CDCl_3$) δ 6.83 (s, 4H), 4.49 (d, *J* = 4.0 Hz, 2H), 3.77 (s, 3H), 2.27 (d, *J* = 6.1 Hz, 3H). **^{13}C NMR** (101 MHz, $CDCl_3$) δ 206.3, 154.6, 152.1 (d, *J* = 2.4 Hz), 115.7, 115.0, 74.0 (d, *J* = 43.4 Hz), 55.8, 26.7 (d, *J* = 41.5 Hz). **IR** (neat, cm^{-1}): 2925, 2836, 1679, 1508, 1435, 1225, 1033, 823, 716, 519. **HRMS** (ESI$^+$) calcd. for $C_9{}^{13}CH_{12}O_3Na$ $[M+Na]^+$ 204.0712, found 204.0715.

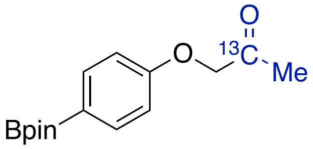

1-(4-(4,4,5,5-tetramethyl-1,3,2-dioxaborolan-2-yl)phenoxy)propan-2-one-2-^{13}*C* ([13C]1o). Following GP2, 2-methyl-2-((4-(4,4,5,5-tetramethyl-1,3,2-dioxaborolan-2-yl)phenoxy)methyl)-2,3-dihydroquinazolin-4(1*H*)-one (78.8 mg, 0.20 mmol) and acetyl-1-^{13}*C* chloride (18.7 mg, 0.30 mmol) were used, affording the title compound as a yellow solid (35.5 mg, 64% yield), by using hexanes/Et_2O (3:1) as chromatography eluent. In a second independent experiment, 36.0 mg (65%) were obtained, giving an average yield of 65%. **^{1}H NMR** (400 MHz, $CDCl_3$) δ 7.76 (d, *J* = 8.7 Hz, 2H), 6.88 (d, *J* = 8.6 Hz, 2H), 4.55 (d, *J* = 4.1 Hz, 2H), 2.28 (d, *J* = 6.1 Hz, 3H), 1.33 (s, 12H). **^{13}C NMR** (101 MHz, $CDCl_3$) δ 205.6, 160.2 (d, *J* = 2.5 Hz), 136.7, 113.8, 83.7, 72.8 (d, *J* = 43.5 Hz), 26.6 (d, *J* = 41.7 Hz), 24.8. **^{11}B NMR** (128 MHz, $CDCl_3$) δ 30.9. **IR** (neat, cm^{-1}): 2943, 2864, 1731, 1666, 1453, 1361, 1242, 1148, 1043, 1026, 963, 894, 607. **HRMS** (ESI$^+$) calcd. for $C_{14}{}^{13}CH_{22}BO_4$ $[M+H]^+$ 278.1645, found 278.1635.

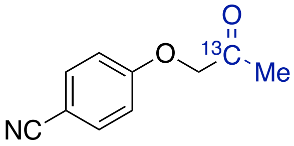

4-(2-oxopropoxy-2-^{13}*C*)benzonitrile ([13C]1p). Following GP2, 4-((2-methyl-4-oxo-1,2,3,4-tetrahydroquinazolin-2-yl)methoxy)benzonitrile (58.6 mg, 0.20 mmol) and acetyl-1-^{13}*C* chloride (18.7 mg, 0.30 mmol) were used, affording the title compound as a colorless oil (30.6 mg, 87% yield), by using hexanes/Et_2O (2:1) as chromatography eluent. In a second independent experiment, 30.2 mg (86%) were obtained, giving an average yield of 87%. **^{1}H NMR** (400 MHz, $CDCl_3$) δ 7.60 (d, *J* = 8.9 Hz, 2H), 6.94 (d, *J* = 8.9 Hz, 2H), 4.61 (d, *J* = 3.9 Hz, 2H), 2.28 (d, *J* = 6.1 Hz, 3H). **^{13}C NMR** (101 MHz, $CDCl_3$) δ 203.6, 161.0 (d, *J* = 2.3 Hz), 134.3, 118.9, 115.4, 105.4, 72.9 (d, *J* = 42.8 Hz), 26.7 (d, *J* = 41.8 Hz). **IR** (neat, cm^{-1}): 3103, 2920, 2225, 1692, 1604, 1507, 1433, 1359, 1305, 1261, 1240, 1173, 1155, 1066, 835, 549. **HRMS** (ESI$^+$) calcd. for $C_9{}^{13}CH_9NO_2Na$ $[M+Na]^+$ 199.0559, found 199.0566.

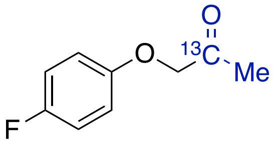

1-(4-fluorophenoxy)propan-2-one-2-^{13}*C* ([13C]1q). Following GP2, 2-((4-fluorophenoxy)methyl)-2-methyl-2,3-dihydroquinazolin-4(1*H*)-one (57.2 mg, 0.20 mmol) and

acetyl-1-^{13}C chloride (18.7 mg, 0.30 mmol) were used, affording the title compound as a yellow oil (24.0 mg, 71% yield), by using hexanes/Et_2O (6:1) as chromatography eluent. In a second independent experiment, 24.7 mg (73%) were obtained, giving an average yield of 72%. **^{1}H NMR** (400 MHz, $CDCl_3$) δ 7.04 – 6.94 (m, 2H), 6.88 – 6.78 (m, 2H), 4.51 (d, J = 4.0 Hz, 2H), 2.27 (d, J = 6.1 Hz, 3H). **^{13}C NMR** (101 MHz, $CDCl_3$) δ 205.4, 159.0, 155.4 (d, J = 272.1 Hz), 116.1 (d, J = 23.1 Hz), 115.7 (d, J = 8.1 Hz), 73.7 (d, J = 43.4 Hz), 26.6 (d, J = 41.6 Hz). **^{19}F NMR** (376 MHz, $CDCl_3$) δ -122.69. **IR** (neat, cm^{-1}): 2923, 2853, 1693, 1505, 1434, 1358, 1252, 1208, 1153, 1066, 828, 759, 725. **HRMS** (ESI^+) calcd. for $C_8{}^{13}CH_9FO_2Na$ $[M+Na]^+$ 192.0512, found 192.0515.

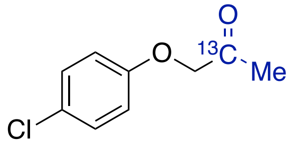

1-(4-Chlorophenoxy)propan-2-one-2-^{13}*C* ([13C]1r). Following GP2, 2-((4-chlorophenoxy)methyl)-2-methyl-2,3-dihydroquinazolin-4(1*H*)-one (60.4 mg, 0.20 mmol) and acetyl-1-^{13}C chloride (18.7 mg, 0.30 mmol) were used, affording the title compound as a colorless oil (23.3 mg, 63% yield), by using hexanes/Et_2O (6:1) as chromatography eluent. In a second independent experiment, 25.2 mg (68%) were obtained, giving an average yield of 66%. **^{1}H NMR** (400 MHz, $CDCl_3$) δ 7.4 – 7.1 (m, 2H), 6.8 (d, J = 9.0 Hz, 2H), 4.5 (d, J = 4.0 Hz, 2H), 2.3 (d, J = 6.1 Hz, 3H). **^{13}C NMR** (101 MHz, $CDCl_3$) δ 205.2, 156.5 (d, J = 2.6 Hz), 129.7, 126.9, 116.0, 73.4 (d, J = 43.2 Hz), 26.7 (d, J = 41.7 Hz). **IR** (neat, cm^{-1}): 2923, 1692, 1583, 1489, 1432, 1357, 1231, 1154, 1064, 823, 672, 636, 507. **HRMS** (ESI^+) calcd. for $C_8{}^{13}CH_9ClNaO_2$ $[M+Na]^+$ 208.0222, found 208.0217.

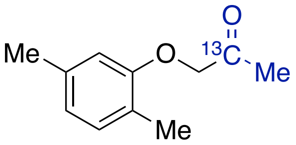

1-(2,5-Dimethylphenoxy)propan-2-one-2-^{13}*C* ([13C]1s). Following GP2, 2-((2,5-dimethylphenoxy)methyl)-2-methyl-2,3-dihydroquinazolin-4(1*H*)-one (59.2 mg, 0.20 mmol) and acetyl-1-^{13}C chloride (18.7 mg, 0.30 mmol) were used, affording the title compound as a colorless oil (26.1 mg, 73% yield), by using hexanes/Et_2O (6:1) as chromatography eluent. In a second independent experiment, 26.5 mg (74%) were obtained, giving an average yield of 73%. **^{1}H NMR** (400 MHz, $CDCl_3$) δ 7.05 (d, J = 7.5 Hz, 1H), 6.73 (d, J = 7.5 Hz, 1H), 6.48 (s, 1H), 4.50 (d, J = 4.2 Hz, 2H), 2.32 (d, J = 6.2 Hz, 3H), 2.30 (s, 3H), 2.26 (s, 3H). **^{13}C NMR** (101 MHz, $CDCl_3$) δ

206.9, 155.9 (d, J = 2.2 Hz), 137.0, 130.9, 123.8, 122.1, 111.9, 73.3 (d, J = 44.0 Hz), 26.9 (d, J = 41.6 Hz), 21.5, 16.0. **IR** (neat, cm^{-1}): 2923, 1679, 1585, 1509, 1413, 1354, 1264, 1154, 1130, 1063, 804. **HRMS** (ESI^+) calcd. for $C_{10}{}^{13}CH_{14}O_2Na$ $[M+Na]^+$ 202.0920, found 202.0916.

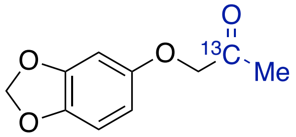

1-(Benzo[*d*][1,3]dioxol-5-yloxy)propan-2-one-2-^{13}*C* ([13C]1t). Following GP2, 2-((benzo[*d*][1,3]dioxol-5-yloxy)methyl)-2-methyl-2,3-dihydroquinazolin-4(1*H*)-one (62.4 mg, 0.20 mmol) and acetyl-1-^{13}*C* chloride (18.7 mg, 0.30 mmol) were used, affording the title compound as a colorless oil (24.6 mg, 63% yield), by using hexanes/Et_2O (4:1) as chromatography eluent. In a second independent experiment, 26.5 mg (68%) were obtained, giving an average yield of 65%. **^{1}H NMR** (400 MHz, $CDCl_3$) δ 6.70 (d, J = 8.5 Hz, 1H), 6.51 (d, J = 2.6 Hz, 1H), 6.27 (dd, J = 8.5, 2.6 Hz, 1H), 5.93 (s, 2H), 4.46 (d, J = 3.9 Hz, 2H), 2.26 (d, J = 6.1 Hz, 3H). **^{13}C NMR** (101 MHz, $CDCl_3$) δ 205.8, 153.2 (d, J = 2.6 Hz), 148.5, 142.4, 108.0, 105.5, 101.3, 98.3, 74.0 (d, J = 43.2 Hz), 26.6 (d, J = 41.6 Hz). **IR** (neat, cm^{-1}): 2897, 1679, 1629, 1503, 1486, 1431, 1356, 1244, 1182, 1136, 1136, 923, 816. **HRMS** (ESI^+) calcd. for $C_9{}^{13}CH_{10}O_4Na$ $[M+Na]^+$ 218.0505, found 218.0504.

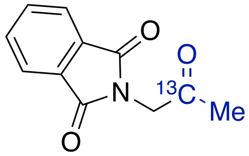

2-(2-oxopropyl-2-^{13}*C*)isoindoline-1,3-dione ([13C]1u). Following GP2, 2-((2-methyl-4-oxo-1,2,3,4-tetrahydroquinazolin-2-yl)methyl)isoindoline-1,3-dione (64.2 mg, 0.20 mmol) and acetyl-1-^{13}*C* chloride (18.7 mg, 0.30 mmol) were used, affording the title compound as a yellow solid (25.7 mg, 63% yield, melting point: 120 °C), by using hexanes/Et_2O (2:1) as chromatography eluent. In a second independent experiment, 26.9 mg (66%) were obtained, giving an average yield of 64%. **^{1}H NMR** (400 MHz, $CDCl_3$) δ 7.91 – 7.79 (m, 2H), 7.79 – 7.61 (m, 2H), 4.49 (d, J = 4.0 Hz, 2H), 2.26 (d, J = 6.0 Hz, 3H). **^{13}C NMR** (101 MHz, $CDCl_3$) δ 199.6, 167.6, 134.1, 132.0, 123.5, 47.1 (d, J = 39.0 Hz), 26.9 (d, J = 42.4 Hz). **IR** (neat, cm^{-1}): 3473, 2927, 1771, 1714, 1683, 1467, 1405, 1156, 1009, 714, 501. **HRMS** (ESI^+) calcd. for $C_{10}{}^{13}CH_9NO_3Na$ $[M+Na]^+$ 227.0508, found 227.0513.

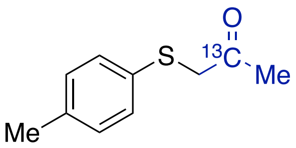

1-(*p*-Tolylthio)propan-2-one-2-^{13}C ([13C]1v). Following GP2, 2-methyl-2-((*p*-tolylthio)methyl)-2,3-dihydroquinazolin-4(1*H*)-one (59.6 mg, 0.20 mmol) and acetyl-1-^{13}C chloride (18.7 mg, 0.30 mmol) were used, affording the title compound as a yellow oil (20.3 mg, 56% yield), by using hexanes/Et_2O (5:1) as chromatography eluent. In a second independent experiment, 20.0 mg (56%) were obtained, giving an average yield of 56%. **^{1}H NMR** (400 MHz, $CDCl_3$) δ 7.28 – 7.24 (m, 2H), 7.10 (d, *J* = 7.9 Hz, 2H), 3.61 (d, *J* = 4.4 Hz, 2H), 2.31 (s, 3H), 2.26 (d, *J* = 5.9 Hz, 3H). **^{13}C NMR** (101 MHz, $CDCl_3$) δ 203.8, 137.5, 131.0, 130.6, 130.1, 45.5 (d, *J* = 39.9 Hz), 28.1 (d, *J* = 42.5 Hz), 21.2. **IR** (neat, cm^{-1}): 3059, 2922, 2851, 1667, 1583, 1481, 1439, 1354, 1201, 1133, 740, 690. **HRMS** (ESI^+) calcd. for $C_9{}^{13}CH_{12}NaOS$ $[M+Na]^+$ 204.0535, found 204.0528.

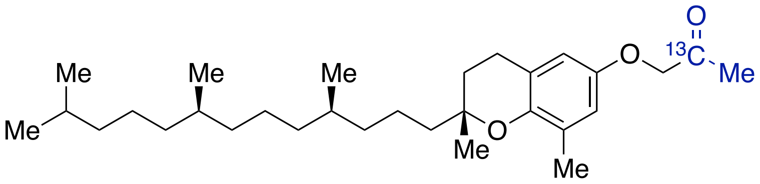

1-(((*R*)-2,8-dimethyl-2-((4*R*,8*R*)-4,8,12-trimethyltridecyl)chroman-6-yl)oxy)propan-2-one-2-^{13}C ([13C]1w). Following GP2, 2-((((*R*)-2,8-dimethyl-2-((4*R*,8*R*)-4,8,12-trimethyltridecyl)chroman-6-yl)oxy)methyl)-2-methyl-2,3-dihydroquinazolin-4(1*H*)-one (115.2 mg, 0.20 mmol) and acetyl-1-^{13}C chloride (18.7 mg, 0.30 mmol) were used, affording the title compound as an orange oil (47.7 mg, 52% yield), by using hexanes/Et_2O (8:1) as chromatography eluent. In a second independent experiment, 49.6 mg (54%) were obtained, giving an average yield of 53%. **^{1}H NMR** (400 MHz, $CDCl_3$) δ 6.6 (d, *J* = 2.4 Hz, 1H), 6.4 (d, *J* = 3.1 Hz, 1H), 4.4 (d, *J* = 4.0 Hz, 2H), 2.7 (td, *J* = 6.6, 2.6 Hz, 2H), 2.3 (d, *J* = 6.1 Hz, 3H), 2.1 (s, 3H), 1.8 (dq, *J* = 20.6, 6.8 Hz, 2H), 1.6 – 1.5 (m, 3H), 1.4 – 1.2 (m, 14H), 1.1 (ddtd, *J* = 33.5, 9.9, 7.3, 4.8 Hz, 7H), 0.9 – 0.8 (m, 12H). **^{13}C NMR** (101 MHz, $CDCl_3$) δ 207.1, 150.5 (d, *J* = 2.5 Hz), 147.0, 127.7, 121.3, 115.6, 112.0, 75.9, 74.0 (d, *J* = 43.5 Hz), 40.2, 39.5, 37.6, 37.6, 37.4, 32.9, 32.8, 31.4, 28.1, 26.8 (d, *J* = 41.3 Hz), 24.9, 24.6, 24.3, 22.9, 22.8, 22.8, 21.1, 19.9, 19.8, 16.4. **IR** (neat, cm^{-1}): 2924, 2867, 1606, 1479, 1377, 1355, 1220, 1150, 1075, 857. **HRMS** (ESI^+) calcd. for $C_{29}{}^{13}CH_{51}O_3$ $[M+H]^+$ 460.3866, found 460.3866.

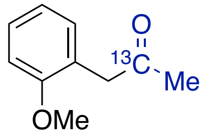

1-(2-Methoxyphenyl)propan-2-one-2-^{13}*C* ([13C]1x). Following GP3, 2-(2-methoxybenzyl)-2-methyl-2,3-dihydroquinazolin-4(1*H*)-one (56.4 mg, 0.20 mmol) and acetyl-1-^{13}*C* chloride (18.7 mg, 0.30 mmol) were used, affording the title compound as a colorless oil (18.1 mg, 55% yield), by using hexanes/Et_2O (4:1) as chromatography eluent. In a second independent experiment, 18.8 mg (57%) were obtained, giving an average yield of 56%. **^{1}H NMR** (400 MHz, $CDCl_3$) δ 7.32 – 7.22 (m, 1H), 7.13 (dd, *J* = 7.4, 1.7 Hz, 1H), 6.96 – 6.85 (m, 2H), 3.81 (s, 3H), 3.67 (d, *J* = 6.6 Hz, 2H), 2.13 (d, *J* = 5.8 Hz, 3H). **^{13}C NMR** (101 MHz, $CDCl_3$) δ 207.2, 157.5 (d, *J* = 1.4 Hz), 131.3 (d, *J* = 1.8 Hz), 128.7, 123.8 (d, *J* = 2.9 Hz), 120.8, 110.6, 55.5, 45.7 (d, *J* = 38.6 Hz), 29.4 (d, *J* = 41.2 Hz). **IR** (neat, cm^{-1}): 3005, 2924, 2838, 1670, 1496, 1464, 1246, 1114, 1030, 754. **HRMS** (ESI^+) calcd. for $C_9{}^{13}CH_{13}O_2$ $[M+H]^+$ 166.0944, found 166.0948.

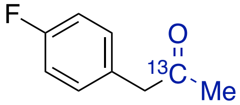

1-(4-Fluorophenyl)propan-2-one-2-^{13}*C* ([13C]1y). Following GP3, 2-(4-fluorobenzyl)-2-methyl-2,3-dihydroquinazolin-4(1*H*)-one (54.0 mg, 0.20 mmol) and acetyl-1-^{13}*C* chloride (18.7 mg, 0.30 mmol) were used, affording the title compound as a colorless oil (17.4 mg, 57% yield), by using hexanes/Et_2O (6:1) as chromatography eluent. In a second independent experiment, 18.4 mg (60%) were obtained, giving an average yield of 58%. **^{1}H NMR** (400 MHz, $CDCl_3$) δ 7.2 – 7.1 (m, 2H), 7.1 – 7.0 (m, 2H), 3.7 (d, *J* = 6.3 Hz, 2H), 2.2 (d, *J* = 5.8 Hz, 3H). **^{13}C NMR** (101 MHz, $CDCl_3$) δ 206.2, 131.1 (d, *J* = 1.7 Hz), 131.0 (d, *J* = 1.7 Hz), 130.0 (d, *J* = 2.7 Hz), 115.7 (d, *J* = 21.3 Hz), 50.1 (d, *J* = 38.1 Hz), 29.4 (d, *J* = 41.1 Hz). **^{19}F NMR** (376 MHz, $CDCl_3$) δ -115.61. **IR** (neat, cm^{-1}): 2925, 2854, 1726, 1677, 1603, 1509, 1224, 1140, 840. **HRMS** (ESI^+) calcd. for $C_8{}^{13}CH_{10}FO$ $[M+H]^+$ 154.0744, found 154.0743.

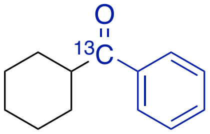

Cyclohexyl(phenyl)methanone-^{13}*C* ([13C]1z). Following GP2, 2-cyclohexyl-2-phenyl-2,3-dihydroquinazolin-4(1*H*)-one (74 mg, 0.24 mmol) and benzoyl chloride-1-^{13}*C* (51 mg, 0.36 mmol) were used, affording the title compound as a colorless oil (25.3 mg, 67% yield), by using hexanes/Et_2O (9:1) as chromatography eluent. In a second independent experiment, 26.1 mg (69%)

were obtained, giving an average yield of 68%. **^{1}H NMR** (400 MHz, $CDCl_3$) δ 7.94 (ddd, *J* = 8.5, 3.5, 1.4 Hz, 2H), 7.59 – 7.51 (m, 1H), 7.45 (tt, *J* = 8.2, 0.9 Hz, 2H), 3.26 (ddq, *J* = 15.0, 7.5, 3.9 Hz, 1H), 2.00 – 1.67 (m, 4H), 1.57 – 1.13 (m, 6H). **^{13}C NMR** (101 MHz, $CDCl_3$) δ 204.0, 136.5 (d, *J* = 51.3 Hz), 132.8, 128.7 (d, *J* = 3.7 Hz), 128.4 (d, *J* = 2.6 Hz), 45.8 (d, *J* = 42.4 Hz), 29.6 (d, *J* = 1.6 Hz), 26.1, 26.0 (d, *J* = 3.7 Hz). **IR** (neat, cm^{-1}): 3061, 2928, 2853, 1639, 1596, 1578, 1447, 1245, 1202, 1171, 966, 764, 694. **HRMS** (ESI^+) calcd. for $C_{12}{}^{13}CH_{16}ONa$ $[M+Na]^+$ 212.1127, found 212.1128.

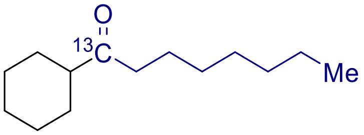

1-Cyclohexyloctan-1-one-1-^{13}*C* ([13C]1za). Following GP2, 2-cyclohexyl-2-heptyl-2,3-dihydroquinazolin-4(1*H*)-one (48 mg, 0.15 mmol) and octanoyl chloride-α-^{13}C (37 mg, 0.23 mmol) were used, affording the title compound as a colorless oil (21.0 mg, 66% yield), by using hexanes/Et_2O (95:5) as chromatography eluent. In a second independent experiment, 21.5 mg (68%) were obtained, giving an average yield of 67%. **^{1}H NMR** (500 MHz, $CDCl_3$) δ 2.40 (td, *J* = 7.4, 5.3 Hz, 2H), 2.35 – 2.28 (m, 1H), 1.84 – 1.70 (m, 4H), 1.68 – 1.66 (m, 1H), 1.54 (dq, *J* = 7.3, 3.4 Hz, 2H), 1.34 – 1.21 (m, 13H), 0.89 – 0.84 (m, 3H). **^{13}C NMR** (101 MHz, $CDCl_3$) δ 214.7, 51.0 (d, *J* = 39.9 Hz), 40.8 (d, *J* = 38.5 Hz), 31.8, 29.4 (d, *J* = 3.0 Hz), 29.3, 28.7 (d, *J* = 1.6 Hz), 26.0, 25.9 (d, *J* = 3.7 Hz), 23. 9 (d, *J* = 2.0 Hz), 22.8, 14.2. **HRMS** (ESI^+) calcd. for $C_{13}{}^{13}CH_{27}O$ $[M+H]^+$ 212.2090, found 212.2082.

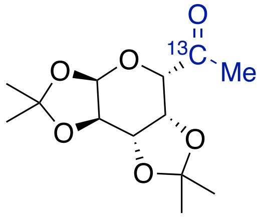

1-((3a*R*,5*S*,5a*R*,8a*S*,8b*R*)-2,2,7,7-tetramethyltetrahydro-5*H*-bis([1,3]dioxolo)[4,5-*b*:4',5'-*d*]pyran-5-yl)ethan-1-one-1-^{13}*C* ([13C]1zb). Following GP2, (*R*)-2-methyl-2-((3a*R*,5*R*,5a*S*,8a*S*,8b*R*)-2,2,7,7-tetramethyltetrahydro-5*H*-bis([1,3]dioxolo)[4,5-*b*:4',5'-*d*]pyran-5-yl)-2,3-dihydroquinazolin-4(1*H*)-one (**2zc**) (78.1 mg, 0.2 mmol) and acetyl-1-^{13}*C* chloride (23.9 mg, 0.30 mmol) were used, affording the title compound as a colorless oil (22.0 mg, 40% yield), by using hexanes/EtOAc (90:10) as eluent. In a second independent experiment, 21.5 mg (39.5%) were obtained, giving an average yield of 40%. **^{1}H NMR** (500 MHz, $CDCl_3$) δ 5.64 (d, *J* = 5.0 Hz, 1H), 4.63 (dd, *J* = 7.8, 2.5 Hz, 1H), 4.55 (dd, *J* = 7.8, 2.2 Hz, 1H), 4.35 (dd, *J* = 5.0, 2.5 Hz, 1H),

4.16 (d, J = 2.3 Hz, 1H), 2.25 (s, 3H), 1.51 – 1.48 (m, 3H), 1.44 (d, J = 0.7 Hz, 3H), 1.33 (d, J = 0.8 Hz, 3H), 1.30 (d, J = 0.7 Hz, 3H). **^{13}C NMR** (126 MHz, $CDCl_3$) δ 207.8, 109.8, 109.0, 96.5, 73.9, 72.6, 70.7, 70.5, 28.1, 26.1, 26.0, 24.9, 24.4. **IR** (neat, cm^{-1}): 2988, 2937, 1740, 1457, 1373, 1254, 1209, 1065, 1005, 892, 774. **HRMS** (ESI$^+$) calcd. for $C_{12}{}^{13}CH_{21}O$ [M+H]$^+$ 274.1366, found 274.1365.

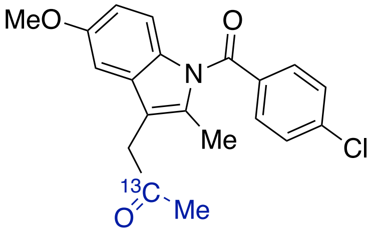

1-(1-(4-chlorobenzoyl)-5-methoxy-2-methyl-1*H*-indol-3-yl)propan-2-one-2-^{13}*C* ([13C]1zc).). Following GP2, 2-((1-(4-chlorobenzoyl)-5-methoxy-2-methyl-1*H*-indol-3-yl)methyl)-2-methyl-2,3-dihydroquinazolin-4(1*H*)-one **(2zd)** (94.8 mg, 0.2 mmol) and acetyl-1-^{13}*C* chloride (23.9 mg, 0.30 mmol) were used, affording the title compound as a brown oil (36.1 mg, 50% yield), by using hexane/EtOAc (4:1) as eluent. In a second independent experiment, 35.5 mg (49.7% yield) were obtained, giving an average yield of 50%. **^{1}H NMR** (500 MHz, $CDCl_3$) δ 7.69-7.64 (m, 2H), 7.50-7.45 (m, 2H), 6.89-6.83 (m, 2H), 6.67 (dd, J = 9.1, 2.5 Hz, 1H), 3.83 (s, 3H), 3.71 (d, J = 6.4 Hz, 2H), 2.38 (d, J = 0.8 Hz, 3H), 2.18 (d, J = 5.8 Hz, 3H).**^{13}C NMR** (126 MHz, $CDCl_3$) δ 205.7, 169.5, 168.3, 156.2, 151.1, 149.3, 146.3, 139.4, 133.8, 131.2, 129.2, 115.1, 112.8, 111.7, 101.2, 55.7, 39.7, 29.2, 13.4. **IR** (neat, cm^{-1}): 2924, 1670, 1590, 1476, 1455, 1476, 1455, 1353, 1312, 1087, 752. **HRMS** (ESI$^+$) calcd. for $C_{19}{}^{13}CH_{19}ClNO_3$ [M+H]$^+$ 357.1082, found 357.1091.

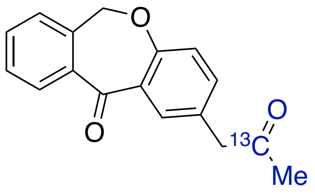

2-(2-oxopropyl-2-13*C*)dibenzo[*b,e*]oxepin-11(6*H*)-one ([13C]1zd). Following the one-pot labeling procedure (GP4), 2-methyl-2-((11-oxo-6,11-dihydrodibenzo[*b*,*e*]oxepin-9-yl)methyl)-2,3-dihydroquinazolin-4(1*H*)-one **(2ze)** (76.9 mg, 0.2 mmol) and acetyl-1-^{13}*C* chloride (23.9 mg, 0.30 mmol) were used, affording the title compound as a yellow oil (25.5 mg, 48% yield), by using hexanes/EtOAc (3:1) as eluent. In a second independent experiment, 24.3 mg (46% yield) were obtained, giving an average yield of 47%. **^{1}H NMR** (500 MHz, $CDCl_3$) δ 8.06 (d, J = 2.4 Hz, 1H), 7.91 (dd, J = 7.7, 1.4 Hz, 1H), 7.58 (td, J = 7.5, 1.4 Hz, 1H), 7.49 (td, J = 7.6, 1.3 Hz, 1H), 7.38

(dd, J = 7.5, 1.3 Hz, 1H), 7.34 (dd, J = 8.4, 2.4 Hz, 1H), 7.05 (d, J = 8.4 Hz, 1H), 5.21 (s, 2H), 3.75 (d, J = 6.3 Hz, 2H), 2.21 (d, J = 5.8 Hz, 3H).**^{13}C NMR** (126 MHz, $CDCl_3$) δ 205.9, 190.8, 160.3, 140.3, 136.3, 135.4, 132.7, 132.4, 129.4, 129.1, 127.7, 125.1, 121.1, 73.5, 49.4, 29.5. **IR** (neat, cm^{-1}): 2924, 1720, 1675, 1640, 1609, 1597, 1568, 1455, 1345, 1299, 1240, 1121, 1014, 834, 758, 642. **HRMS** (ESI^+) calcd. for $C_{16}{}^{13}CH_{15}O_3$ $[M+H]^+$ 268.1049, found 268.1041.

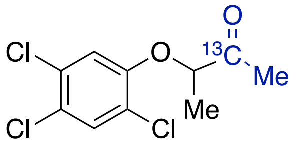

3-(3,4-dichlorophenoxy)butan-2-one-2-^{13}*C* ([13C]1ze. Following GP2, 2-(1-(3,4-dichlorophenoxy)ethyl)-2-methyl-2,3-dihydroquinazolin-4(1*H*)-one **(2zf)** (77.1 mg, 0.2 mmol) and acetyl-1-^{13}*C* chloride (23.9 mg, 0.30 mmol) were used, affording the title compound as a white solid (35.7 mg, 67% yield), by using hexanes/EtOAc (6:1) as eluent. In a second independent experiment, 34.9 mg (65.0%) were obtained, giving an average yield of 66% (melting point: 62 ºC). **^{1}H NMR** (500 MHz, $CDCl_3$) δ 7.49 (s, 1H), 6.88 (s, 1H), 4.69-4.53 (m, 1H), 2.25 (d, J = 6.1 Hz, 3H), 1.55 (dd, J = 6.8, 4.0 Hz, 3H).**^{13}C NMR** (126 MHz, $CDCl_3$) δ 208.0, 152.1, 131.4, 125.4, 122.9, 115.9, 81.0, 80.7, 25.1, 17.2. **IR** (neat, cm^{-1}): 3095, 2990, 2930, 2855, 1720, 1588, 1450, 1271, 1133, 1040, 998, 940, 873, 590. **HRMS** (ESI^+) calcd. for $C_9{}^{13}CH_9Cl_3NaO_2$ $[M+H]^+$ 289.9594, found 289.9596.

6. Low yielding and unsuccessful examples

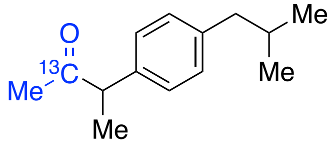

28%

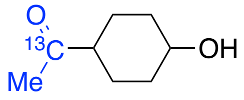

0%

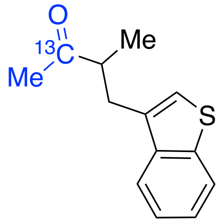

0%

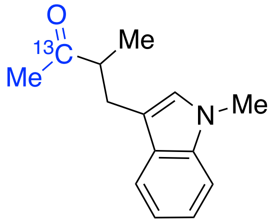

0%

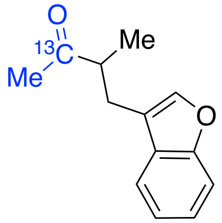

0%

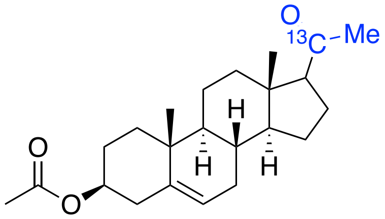

16%
From Pregnenolone acetate

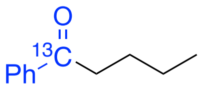

0%

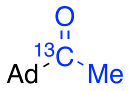

28%

The low yields or lack of reactivity observed with these substrates can be attributed to the (1) proclivity of benzyl radicals to homodimerization pathways, (2) the presence of hydroxyl groups might compromise the targeted C–C bond-formation via competitive acylation events, and (3) the presence of adjacent hindered backbones compromises efficiency and reactivity.

7. Mechanistic Experiments

7.1 Experiments with well-defined nickel complexes

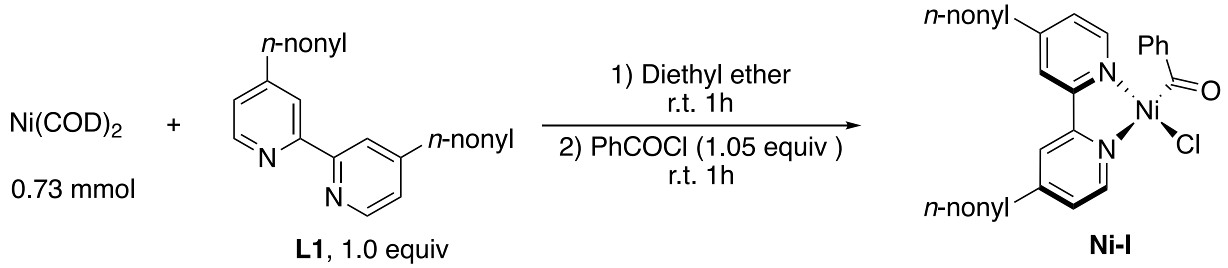

Synthesis of Nickel Complex (Ni-I).[9] A flame-dried 20 mL test tube equipped with a magnetic stirring bar was charged with $Ni(COD)_2$ (200 mg, 0.73 mmol, 1.0 equiv.) and 4,4′-dinonyl-2,2’-pyridine (**L1**) (297 mg, 0.73 mmol, 1.0 equiv.) in a glove box. To the mixture anhydrous diethyl ether (8.0 mL) was added. The resulting purple solution was stirred for 1 h at room temperature. To the reaction tube was added benzoyl chloride (89 µL, 0.76 mmol, 1.05 equiv.) and stirred for 1 h. The resulting dark red suspension was kept in the freezer at – 20 °C overnight. The solid was filtered, washed with pre-cooled diethyl ether (2 x 1 mL) and dried under high vacuum affording **Ni-I** (330 mg, 75%) as a red powder. **Ni-I** was stored at -20 °C in glove box. **^{1}H NMR** (400 MHz, Acetone-d_6) δ 8.76 (d, J = 5.4 Hz, 1H), 8.60 – 8.52 (m, 2H), 8.31 (d, J = 12.6 Hz, 2H), 7.63 (d, J = 5.8 Hz, 1H), 7.49 (d, J = 5.6 Hz, 1H), 7.47 – 7.36 (m, 3H), 7.19 (d, J = 5.7 Hz, 1H), 2.82 (t, J = 7.7 Hz, 2H), 2.74 (t, J = 7.7 Hz, 2H), 1.79 – 1.62 (m, 4H), 1.48 – 1.18 (m, 24H), 0.90 – 0.80 (m, 6H). **^{13}C NMR** (101 MHz, Acetone-d_6) δ 209.9, 156.9, 156.3, 156.2, 152.8, 150.7, 148.9, 136.9, 131.7, 129.6, 128.6, 127.8, 126.9, 123.3, 122.1, 69.2, 36.1, 35.9, 32.6, 32.6, 31.0, 30.5, 30.5, 30.3, 30.2, 30.1, 30.1, 30.0, 29.9, 29.9, 29.7, 23.3, 23.3, 14.4, 14.3.

Catalytic Competency of Ni-I

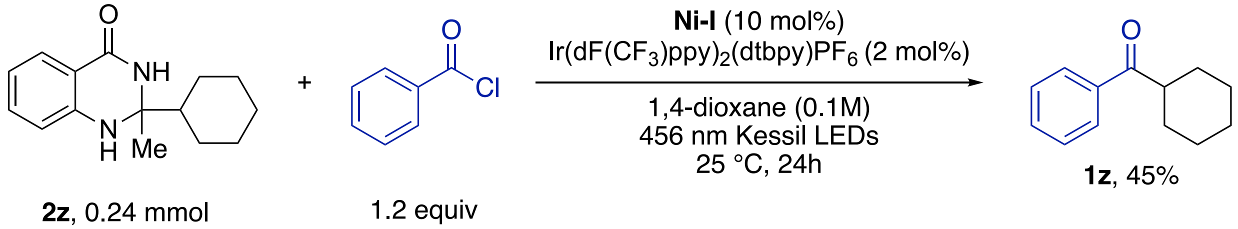

An oven-dried screw-cap test tube containing a stirring bar was charged with 2-cyclohexyl-2-methyl-2,3-dihydroquinazolin-4(1H)-one (59 mg, 0.24 mmol, 1.0 equiv.) and $Ir(dF(CF_3)ppy)_2(dtbpy)PF_6$ (5.4 mg, 2 mol%). The reaction vessel was taken into a nitrogen-filled

glove box, then sequentially charged with **Ni-I** (15.0 mg, 10 mol%), 1,4-dioxane (2 mL) and benzoyl chloride (33 µL, 0.29 mmol, 1.2 equiv.). The reaction mixture was stirred for 3 minutes, then the reaction vessel was sealed, removed from the glovebox and the screw cap was further sealed with parafilm. The reaction mixture was stirred while exposed to blue LED irradiation for 24 hours at 25 °C. Internal standard decane (47 µL, 0.24 mmol, 1.0 equiv.) was added followed by EtOAc (3 mL). An aliquot (~200 µL) was filtered through a mixture of silica, celite and Florisil®, and sequentially washed with EtOAc. The sample was analyzed by GC-FID showing the formation of cyclohexyl phenyl ketone (45%).

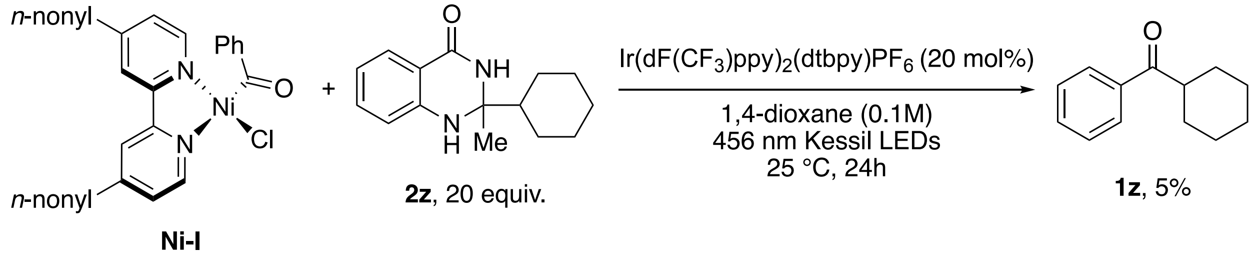

An oven-dried screw-cap test tube containing a stirring bar was charged with 2-cyclohexyl-2-methyl-2,3-dihydroquinazolin-4(1*H*)-one (160 mg, 0.66 mmol, 20 equiv.) and Ir(dF(CF_3)ppy)$_2$(dtbpy)PF_6 (7.4 mg, 20 mol%). The reaction vessel was taken into a nitrogen-filled glove box, then sequentially charged with **Ni-I** (20 mg, 0.033 mmol, 1.0 equiv.) and 1,4-dioxane (3 mL). The reaction mixture was stirred for 3 minutes, then the reaction vessel was sealed, removed from the glovebox and the screw cap was further sealed with parafilm. The reaction mixture was stirred while exposed to blue LED irradiation for 24 hours at 25 °C. Internal standard decane (6.4 µL, 0.033 mmol, 1.0 equiv.) was added followed by EtOAc (3 mL). An aliquot (~300 µL) was filtered through a mixture of silica, celite and Florisil®, and sequentially washed with EtOAc. The sample was analyzed by GC-FID showing the formation of cyclohexyl phenyl ketone (5%). The reaction was repeated in the absence of Ir(dF(CF_3)ppy)$_2$(dtbpy)PF_6 and GC-FID analysis showed no formation of cyclohexyl phenyl ketone (0%).

Stoichiometric reaction with Ni-I

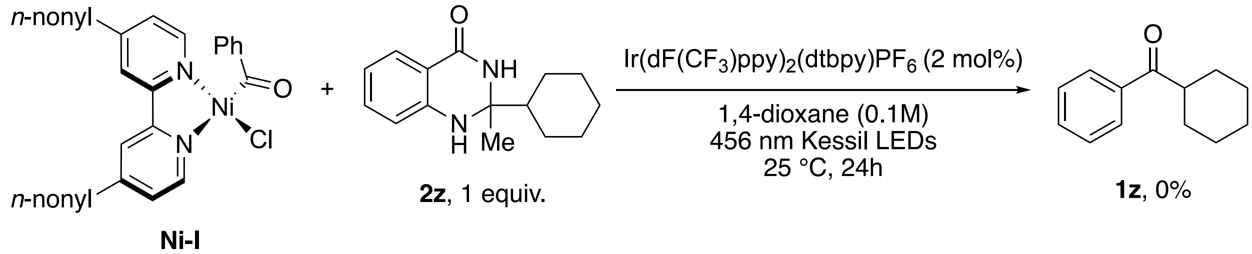

An oven-dried screw-cap test tube containing a stirring bar was charged with 2-cyclohexyl-2-methyl-2,3-dihydroquinazolin-4(1H)-one (16 mg, 0.066 mmol, 1.0 equiv.) and Ir(dF(CF_3)ppy)$_2$(dtbpy)PF_6 (1.5 mg, 2 mol%). The reaction vessel was taken into a nitrogen-filled glove box, then sequentially charged with **Ni-I** (40 mg, 0.066 mmol, 1.0 equiv.) and 1,4-dioxane (2 mL). The reaction mixture was stirred for 3 minutes, then the reaction vessel was sealed, removed from the glovebox and the screw cap was further sealed with parafilm. The reaction mixture was stirred while exposed to blue LED irradiation for 24 hours at 25 °C. Internal standard decane (13 µL, 0.066 mmol, 1.0 equiv.) was added followed by EtOAc (3 mL). An aliquot (~300 µL) was filtered through a mixture of silica, celite and Florisil®, and sequentially washed with EtOAc. The sample was analyzed by GC-FID showing no formation of cyclohexyl phenyl ketone (0%). The reaction was repeated in the presence of increased amount of Ir(dF(CF_3)ppy)$_2$(dtbpy)PF_6 (30 mol%) and GC-FID analysis showed no formation of cyclohexyl phenyl ketone (0%).

7.2 Radical inhibition experiments

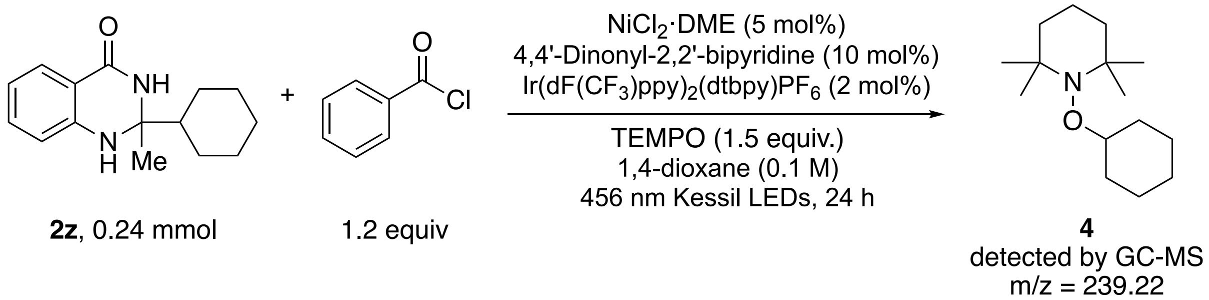

An oven-dried screw-cap test tube containing a stirring bar was charged with 2-cyclohexyl-2-methyl-2,3-dihydroquinazolin-4(1H)-one (59 mg, 0.24 mmol, 1.0 equiv.), Ir(dF(CF_3)ppy)$_2$(dtbpy)PF_6 (5.4 mg, 2 mol%) and TEMPO (56 mg, 0.36 mmol, 1.5 equiv.) and **L1** (9.8 mg, 10 mol%). The reaction vessel was taken into a nitrogen-filled glove box, then sequentially charged with $NiCl_2$·DME (2.6 mg, 5 mol%), 1,4-dioxane (2 mL) and benzoyl chloride (33 µL, 0.29 mmol, 1.2 equiv.). The reaction mixture was stirred for 3 minutes, then the reaction

vessel was sealed, removed from the glovebox and the screw cap was further sealed with parafilm. The reaction mixture was stirred while exposed to blue LED irradiation for 24 hours at 25 °C. The mixture was diluted with EtOAc (3 mL), an aliquot (~200 μL) was filtered through a mixture of silica, celite and Florisil®, and sequentially washed with EtOAc. The sample was analyzed by GC-MS showing the formation of the corresponding TEMPO-adduct **4**.

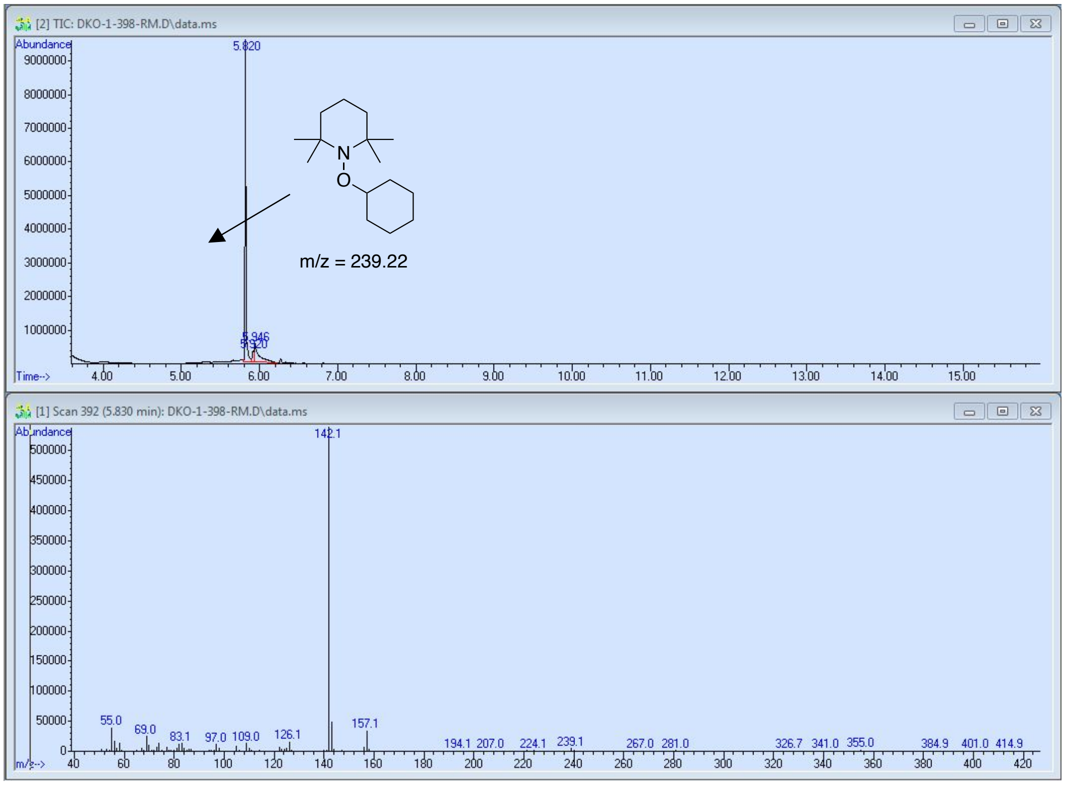

Figure S2. GC-MS spectra of TEMPO radical trapping experiment.

7.3 Chlorine radical trapping

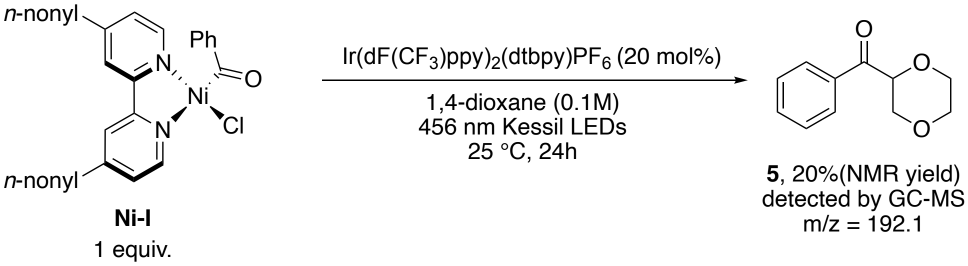

An oven-dried screw-cap test tube containing a stirring bar was charged with $Ir(dF(CF_3)ppy)_2(dtbpy)PF_6$ (7.4 mg, 0.0066 mmol, 0.2 equiv) The reaction vessel was taken into a nitrogen-filled glove box, then sequentially charged with **Ni-I** (20 mg, 0.033 mmol, 1.0 equiv.) and 1,4-dioxane (2 mL). The reaction mixture was stirred for 3 minutes, then the reaction vessel was sealed, removed from the glovebox and the screw cap was further sealed with parafilm. The

reaction mixture was stirred while exposed to blue LED irradiation for 24 hours at 25 °C. The reaction mixture was quenched with a mixture of water and brine (1:1, 10 mL) and extracted with diethyl ether (3 x 10 mL). The combined organic phase was dried over Na_2SO_4, filtered and concentrated under reduced pressure. The reaction was analyzed by ^{1}H NMR using as internal standard CH_2Br_2 (4.6 µL, 0.066 mmol, 2.0 equiv.) showing the formation of **5**[13] in 20% yield. This result confirms the generation of Cl· under standard conditions that led to HAT type mechanism to yield the acetylated product **5**.

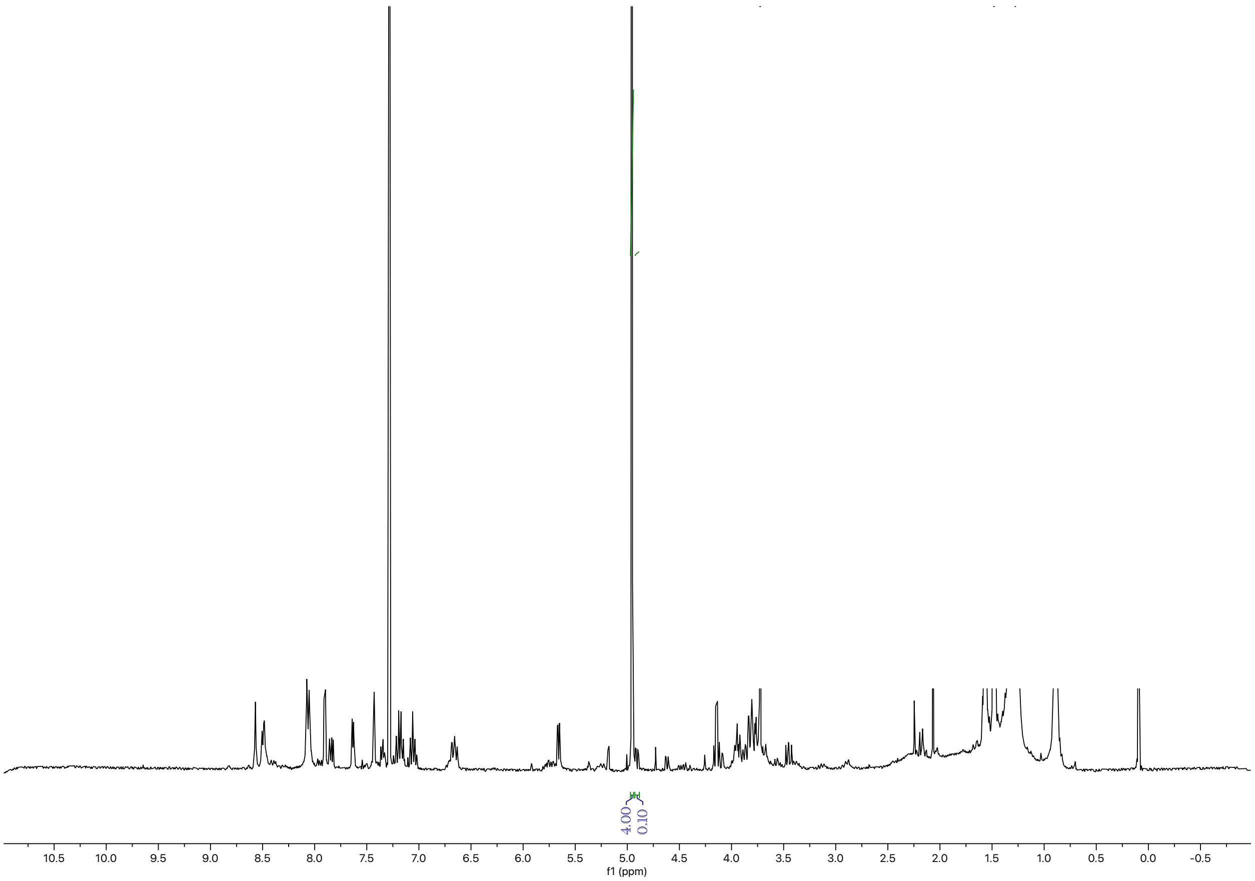

Figure S3. ^{1}H NMR spectra of Cl· radical trapping

7.4 UV-Vis. spectroscopy data

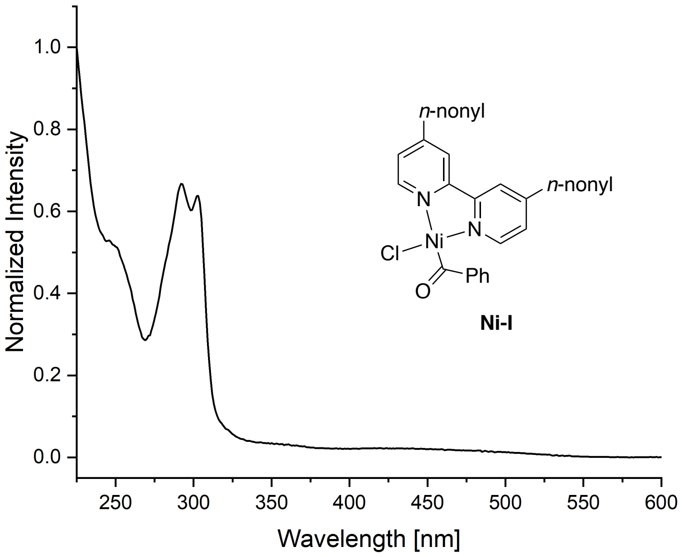

Figure S4. UV-Vis. spectrum of a 20 μM solution of Ni-I in MeCN. Sample for UV-Vis. analysis was prepared in a 3 mL quartz cuvette (path length: l = 1.0 cm) equipped with a rubber septum screwcap. A 20 μM solution of **Ni-I** was prepared in a nitrogen-filled glovebox from dry and degassed MeCN. UV-Vis measurements were carried out on an Agilent Cary 60 UV-Vis spectrophotometer.

7.5 Fluorescence Quenching Studies

UV-Vis measurements were carried out on an Agilent Cary 60 UV-Vis spectrophotometer. Steady-state emission quenching studies were performed using an Fluorolog Horiba Jobin Yvon spectrofluorimeter. Samples were prepared in an Argon filled glove-box. 1,4-dioxane and acetonitrile were previously degassed by freeze-pump-thaw. Stock solutions of $Ir[dF(CF_3)ppy]_2(dtbpy)PF_6$ and the appropriate quenchers were prepared at a concentration of 1 mM. In a typical experiment, a 10 μM cuvette solution of $Ir[dF(CF_3)ppy]_2(dtbpy)PF_6$ was prepared with the desired quencher concentration in a 1.0 cm screw capped quartz cuvette. Samples with various quencher concentrations were irradiated at λ_{exc} = 420 nm and emission intensity was measured at λ_{em} = 560 nm. Sample absorbance was never above 0.1 OD to avoid inner filter effect and establish single photon emission behavior. The relative intensity I_0/I was calculated as a function of quencher concentration.

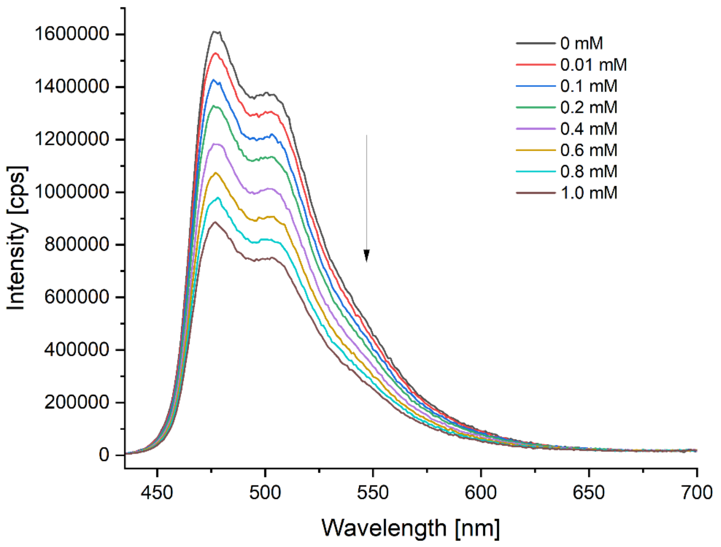

Figure S5. Steady-state emission of Ir[dF(CF_3)ppy]$_2$(dtbpy) [10 μM] in the presence of **1a** in various concentrations in 1,4-dioxane. λ_{exc} = 420 nm.

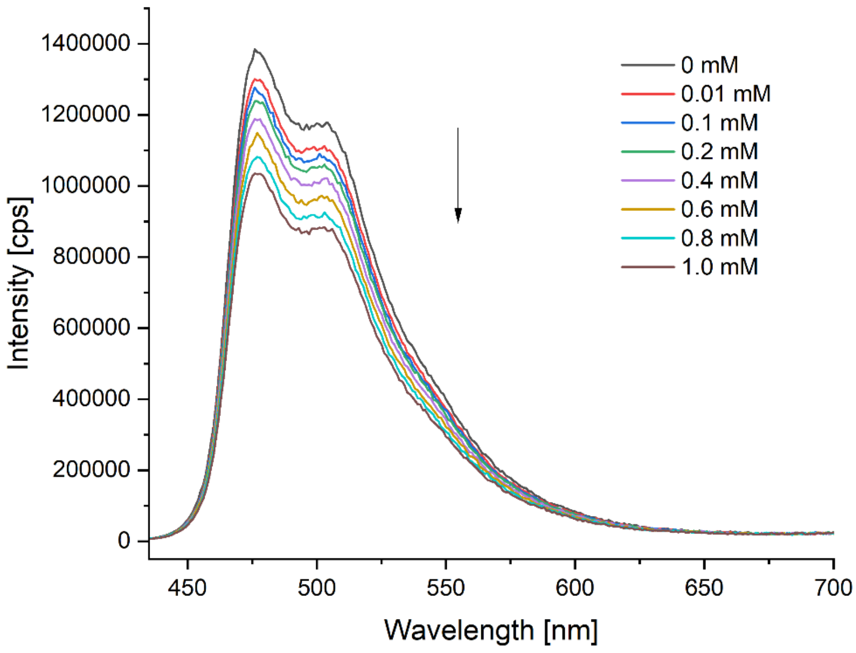

Figure S6. Steady-state emission of Ir[dF(CF_3)ppy]$_2$(dtbpy) [10 μM] in the presence of **1b** in various concentrations in 1,4-dioxane. λ_{exc} = 420 nm.

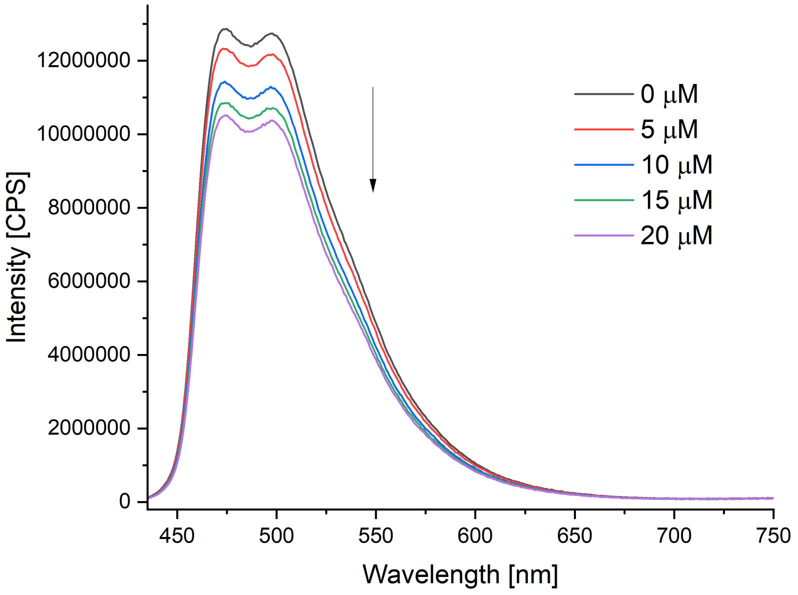

Figure S7. Steady-state emission of Ir[dF(CF_3)ppy]$_2$(dtbpy) [10 μM] in the presence of **Ni-I** in various concentrations in 1,4-dioxane. λ_{exc} = 420 nm.

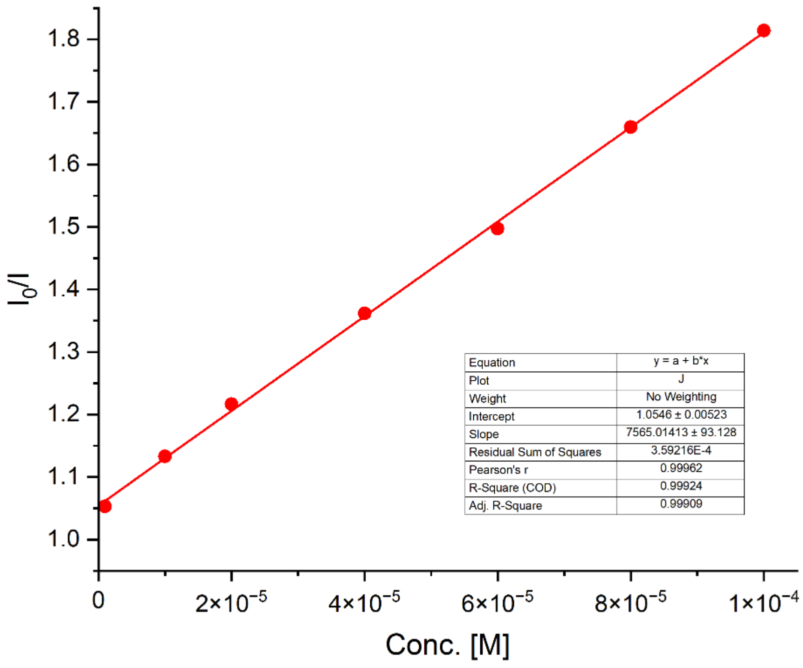

Figure S8. Stern-Volmer plot of **1a**

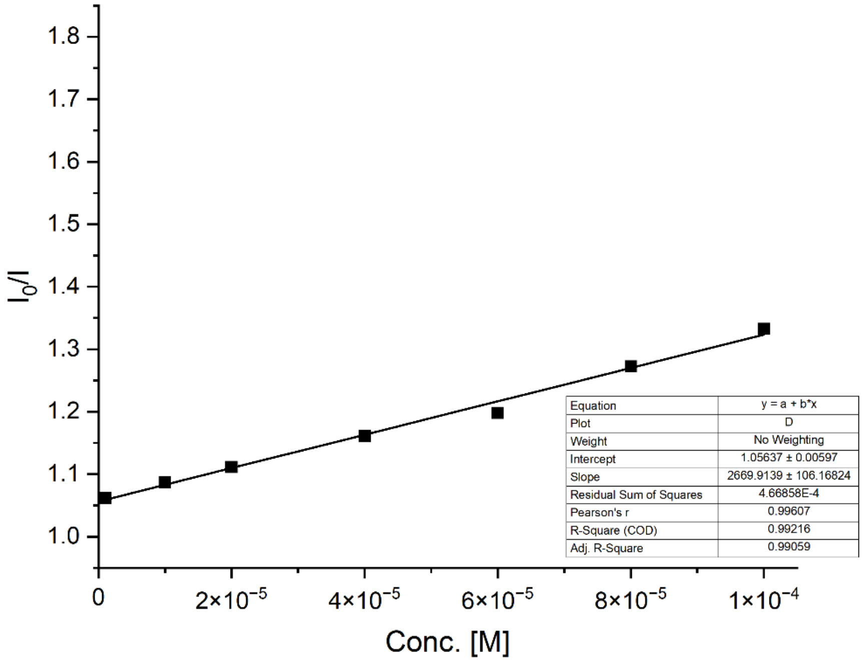

Figure S9. Stern-Volmer plot of benzoyl chloride.

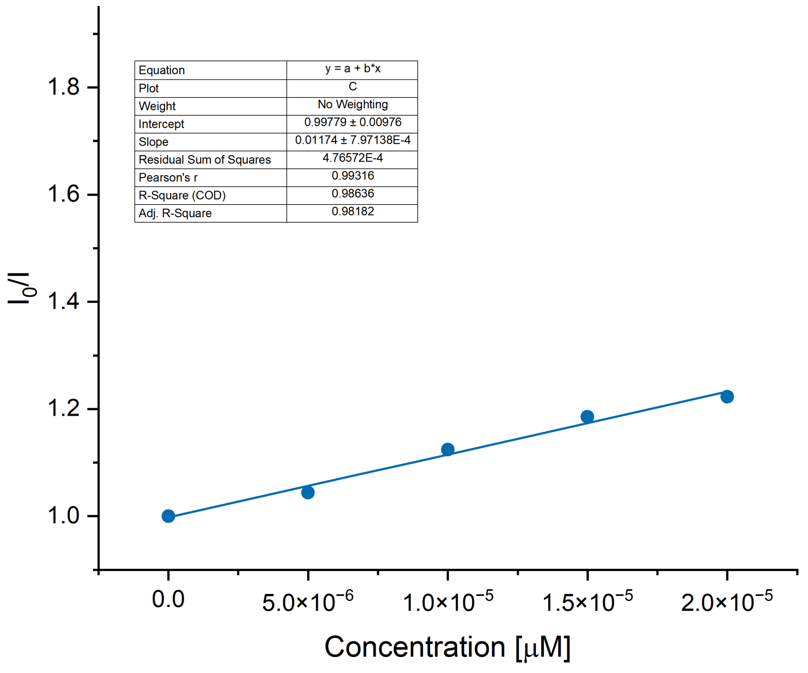

Figure S10. Stern-Volmer plot of **Ni-I**

8. Crystal X-ray diffraction data

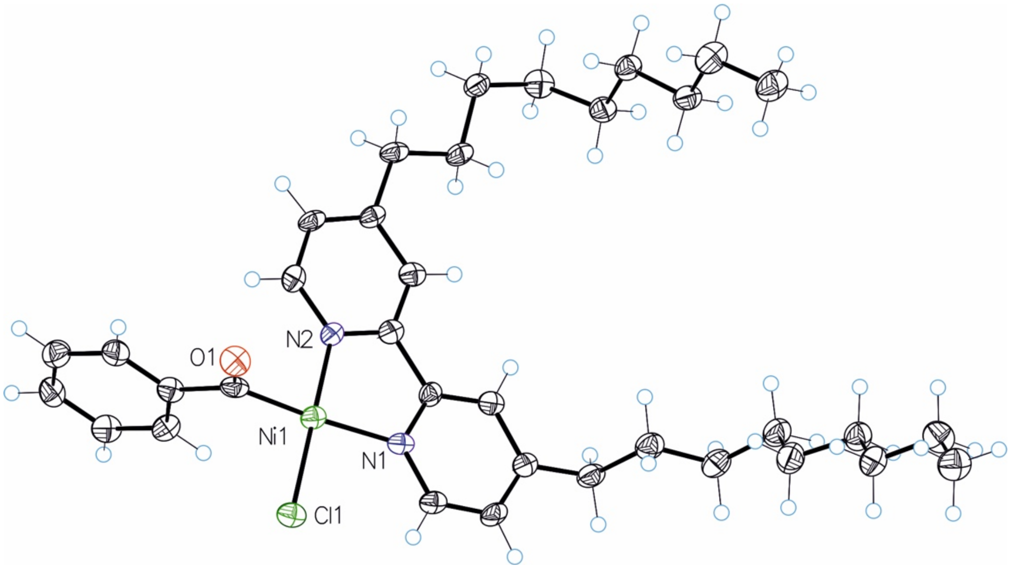

Table S2. Crystal data and structure refinement for **Ni-I**.

Identification code	mo_DKO1396_0m_5
Empirical formula	$C_{35}H_{49}ClN_2NiO$
Formula weight	607.92
Temperature/K	99.96
Crystal system	triclinic
Space group	P-1
a/Å	7.2452(18)
b/Å	11.945(3)
c/Å	19.588(5)
α/°	81.348(3)
β/°	84.948(3)
γ/°	73.127(3)
Volume/Å^3	1602.0(7)
Z	2
ρ_{calc}g/cm^3	1.260
μ/mm^{-1}	0.718
F(000)	652.0
Crystal size/mm^3	0.3 × 0.1 × 0.05
Radiation	MoKα (λ = 0.71073)

2Θ range for data collection/°	3.594 to 51.79
Index ranges	$-8 \leq h \leq 8$, $-14 \leq k \leq 14$, $0 \leq l \leq 24$
Reflections collected	5902
Independent reflections	5902 [R_{int} = ?, R_{sigma} = 0.0948]
Data/restraints/parameters	5902/0/364
Goodness-of-fit on F^2	1.069
Final R indexes [I>=2σ (I)]	R_1 = 0.0784, wR_2 = 0.1963
Final R indexes [all data]	R_1 = 0.1049, wR_2 = 0.2161
Largest diff. peak/hole / e Å^{-3}	1.88/-0.74

Table S3 Fractional Atomic Coordinates (×10^4) and Equivalent Isotropic Displacement Parameters (Å^2×10^3) for mo_DKO1396_0m_5. U_{eq} is defined as 1/3 of the trace of the orthogonalized U_{IJ} tensor.

**Atom**	***x***	***y***	***z***	**U(eq)**
Ni1	2772.4(9)	6796.4(5)	4056.4(3)	23.2(2)
Cl1	2140.7(18)	8678.4(10)	3703.3(6)	28.4(3)
O1	5760(5)	6426(3)	3101.5(19)	32.4(9)
N1	1626(6)	6972(3)	5011(2)	22.8(9)
N2	3149(6)	5149(3)	4373(2)	22.0(9)
C1	4011(8)	6549(4)	3192(3)	27.2(11)
C2	2871(7)	6403(4)	2619(3)	26.6(11)
C3	3865(8)	5934(4)	2036(3)	31.4(12)
C4	2815(8)	5771(5)	1509(3)	34.2(13)
C5	809(8)	6086(4)	1562(3)	32.1(13)
C6	-175(8)	6549(5)	2139(3)	31.4(12)
C7	863(8)	6711(4)	2672(3)	28.5(12)
C8	867(7)	7966(4)	5301(3)	24.9(11)
C9	119(8)	7970(4)	5970(3)	27.9(11)
C10	110(7)	6914(4)	6387(3)	25.6(11)
C11	932(7)	5875(4)	6079(3)	25.4(11)
C12	1652(7)	5927(4)	5407(2)	23.0(11)
C13	2533(7)	4889(4)	5041(2)	23.6(11)
C14	2753(7)	3737(4)	5347(3)	25.3(11)
C15	3585(7)	2783(4)	4972(3)	23.0(10)
C16	4149(7)	3074(4)	4289(3)	25.2(11)

Table S3 Fractional Atomic Coordinates (×10[4]) and Equivalent Isotropic Displacement Parameters (Å[2]×10[3]) for mo_DKO1396_0m_5. U_{eq} is defined as 1/3 of the trace of the orthogonalized U_{IJ} tensor.

Atom	*x*	*y*	*z*	U(eq)
C17	3939(7)	4230(4)	4011(3)	24.3(11)
C18	-686(8)	6878(5)	7115(3)	31.1(12)
C19	872(8)	6577(5)	7647(3)	32.9(12)
C20	13(8)	6657(5)	8377(3)	33.2(13)
C21	1467(9)	6301(5)	8941(3)	35.4(13)
C22	2764(9)	7119(5)	8927(3)	38.8(14)
C23	3921(8)	6874(5)	9568(3)	32.8(12)
C24	5213(8)	7678(5)	9572(3)	34.2(13)
C25	6424(9)	7395(5)	10194(3)	40.6(14)
C26	7667(9)	8239(5)	10203(3)	42.8(15)
C27	3902(7)	1516(4)	5289(3)	26.3(11)
C28	3092(8)	1294(4)	6030(2)	25.8(11)
C29	3605(8)	-26(4)	6294(2)	27.6(11)
C30	2736(8)	-328(5)	7014(3)	33.4(13)
C31	3244(8)	253(5)	7585(3)	29.9(12)
C32	5369(7)	-135(4)	7747(3)	28.2(11)
C33	5893(8)	549(4)	8261(3)	30.7(12)
C34	7993(9)	198(6)	8406(3)	40.9(14)
C35	8548(9)	840(5)	8936(3)	40.8(14)

Table S4 Anisotropic Displacement Parameters (Å[2]×10[3]) for mo_DKO1396_0m_5. The Anisotropic displacement factor exponent takes the form: $-2\pi^2[h^2a^{*2}U_{11}+2hka^*b^*U_{12}+\ldots]$.

Atom	U_{11}	U_{22}	U_{33}	U_{23}	U_{13}	U_{12}
Ni1	20.6(4)	21.5(3)	30.0(4)	-6.6(2)	-4.3(3)	-7.2(3)
Cl1	25.5(7)	23.5(6)	37.8(7)	-3.4(5)	-6.2(6)	-8.4(5)
O1	17.9(19)	41(2)	41(2)	-7.0(16)	-4.1(17)	-10.4(16)
N1	18(2)	18(2)	36(2)	-6.2(16)	-10.3(18)	-6.2(16)
N2	21(2)	23(2)	23(2)	-6.5(16)	0.3(18)	-5.7(17)
C1	28(3)	17(2)	37(3)	-3(2)	-8(2)	-4(2)
C2	24(3)	26(3)	32(3)	-3(2)	-5(2)	-8(2)
C3	28(3)	31(3)	32(3)	-3(2)	-3(2)	-3(2)
C4	32(3)	35(3)	34(3)	-8(2)	-1(3)	-5(2)

Table S4 Anisotropic Displacement Parameters (Å^2×10^3) for mo_DKO1396_0m_5. The Anisotropic displacement factor exponent takes the form: $-2\pi^2[h^2a^{*2}U_{11}+2hka^*b^*U_{12}+\ldots]$.

Atom	U_{11}	U_{22}	U_{33}	U_{23}	U_{13}	U_{12}
C5	40(3)	30(3)	33(3)	-1(2)	-14(3)	-19(2)
C6	25(3)	31(3)	41(3)	-6(2)	-6(3)	-10(2)
C7	26(3)	29(3)	33(3)	-9(2)	2(2)	-11(2)
C8	22(3)	20(2)	36(3)	-7(2)	-7(2)	-8(2)
C9	28(3)	22(3)	38(3)	-14(2)	-8(2)	-7(2)
C10	18(3)	28(3)	33(3)	-11(2)	-9(2)	-4(2)
C11	23(3)	22(2)	33(3)	-5(2)	-10(2)	-7(2)
C12	23(3)	24(3)	24(3)	-6.8(19)	-3(2)	-8(2)
C13	15(2)	27(3)	32(3)	-10(2)	-6(2)	-6(2)
C14	19(3)	25(3)	33(3)	-6(2)	-5(2)	-6(2)
C15	18(3)	22(2)	32(3)	-11.5(19)	-4(2)	-6(2)
C16	22(3)	20(2)	36(3)	-13(2)	-5(2)	-4(2)
C17	23(3)	29(3)	27(3)	-8(2)	-4(2)	-13(2)
C18	31(3)	25(3)	38(3)	-13(2)	-7(2)	-3(2)
C19	34(3)	29(3)	38(3)	-10(2)	-5(3)	-9(2)
C20	37(3)	30(3)	37(3)	-11(2)	1(3)	-14(2)
C21	46(4)	29(3)	33(3)	-7(2)	0(3)	-13(3)
C22	47(4)	39(3)	33(3)	-8(2)	-1(3)	-13(3)
C23	37(3)	32(3)	32(3)	-6(2)	-2(3)	-13(2)
C24	34(3)	38(3)	31(3)	-9(2)	2(3)	-11(3)
C25	42(4)	39(3)	43(3)	-1(3)	-6(3)	-15(3)
C26	40(4)	42(3)	50(4)	-9(3)	-6(3)	-15(3)
C27	23(3)	18(2)	40(3)	-9(2)	-6(2)	-4(2)
C28	25(3)	21(2)	34(3)	-11(2)	-1(2)	-7(2)
C29	28(3)	25(3)	30(3)	-10(2)	-2(2)	-4(2)
C30	29(3)	33(3)	39(3)	-5(2)	-3(3)	-10(2)
C31	25(3)	31(3)	30(3)	-4(2)	1(2)	-3(2)
C32	25(3)	29(3)	33(3)	-9(2)	-3(2)	-7(2)
C33	31(3)	26(3)	36(3)	-8(2)	-3(2)	-5(2)
C34	30(3)	48(4)	48(3)	-19(3)	-2(3)	-10(3)
C35	43(4)	38(3)	48(3)	-15(3)	-5(3)	-17(3)

Table S5 Bond Lengths for mo_DKO1396_0m_5.

Atom	Atom	Length/Å	Atom	Atom	Length/Å
Ni1	Cl1	2.1755(14)	C13	C14	1.386(7)
Ni1	N1	1.994(4)	C14	C15	1.406(7)
Ni1	N2	1.919(4)	C15	C16	1.387(7)
Ni1	C1	1.868(5)	C15	C27	1.507(6)
O1	C1	1.232(6)	C16	C17	1.375(7)
N1	C8	1.344(6)	C18	C19	1.533(8)
N1	C12	1.363(6)	C19	C20	1.514(7)
N2	C13	1.364(6)	C20	C21	1.522(8)
N2	C17	1.354(6)	C21	C22	1.536(8)
C1	C2	1.509(7)	C22	C23	1.516(8)
C2	C3	1.397(7)	C23	C24	1.524(8)
C2	C7	1.392(7)	C24	C25	1.503(8)
C3	C4	1.401(8)	C25	C26	1.537(8)
C4	C5	1.390(8)	C27	C28	1.531(7)
C5	C6	1.382(8)	C28	C29	1.530(6)
C6	C7	1.405(8)	C29	C30	1.528(7)
C8	C9	1.373(7)	C30	C31	1.525(8)
C9	C10	1.397(7)	C31	C32	1.521(7)
C10	C11	1.411(7)	C32	C33	1.523(7)
C10	C18	1.490(7)	C33	C34	1.497(8)
C11	C12	1.370(7)	C34	C35	1.522(8)
C12	C13	1.479(7)			

Table S6 Bond Angles for mo_DKO1396_0m_5.

Atom	Atom	Atom	Angle/°	Atom	Atom	Atom	Angle/°
N1	Ni1	Cl1	95.65(12)	N1	C12	C11	122.0(4)
N2	Ni1	Cl1	176.21(13)	N1	C12	C13	113.3(4)
N2	Ni1	N1	82.82(16)	C11	C12	C13	124.8(4)
C1	Ni1	Cl1	87.28(15)	N2	C13	C12	114.7(4)
C1	Ni1	N1	175.63(19)	N2	C13	C14	122.0(4)
C1	Ni1	N2	94.45(18)	C14	C13	C12	123.3(4)
C8	N1	Ni1	128.5(3)	C13	C14	C15	120.9(4)
C8	N1	C12	117.7(4)	C14	C15	C27	122.7(4)
C12	N1	Ni1	113.8(3)	C16	C15	C14	115.9(4)
C13	N2	Ni1	115.4(3)	C16	C15	C27	121.4(4)

Table S6 Bond Angles for mo_DKO1396_0m_5.

Atom	Atom	Atom	Angle/°	Atom	Atom	Atom	Angle/°
C17	N2	Ni1	127.6(3)	C17	C16	C15	121.1(5)
C17	N2	C13	117.0(4)	N2	C17	C16	123.0(4)
O1	C1	Ni1	120.7(4)	C10	C18	C19	113.3(4)
O1	C1	C2	120.2(5)	C20	C19	C18	112.1(5)
C2	C1	Ni1	118.9(4)	C19	C20	C21	115.4(5)
C3	C2	C1	118.9(5)	C20	C21	C22	114.8(4)
C7	C2	C1	120.9(5)	C23	C22	C21	113.0(4)
C7	C2	C3	120.1(5)	C22	C23	C24	114.4(4)
C2	C3	C4	119.2(5)	C25	C24	C23	114.2(4)
C5	C4	C3	120.5(5)	C24	C25	C26	113.5(5)
C6	C5	C4	120.3(5)	C15	C27	C28	117.1(4)
C5	C6	C7	119.7(5)	C29	C28	C27	111.2(4)
C2	C7	C6	120.2(5)	C30	C29	C28	114.5(4)
N1	C8	C9	123.0(4)	C31	C30	C29	115.4(4)
C8	C9	C10	120.6(4)	C32	C31	C30	114.9(4)
C9	C10	C11	115.8(4)	C31	C32	C33	114.3(4)
C9	C10	C18	122.3(4)	C34	C33	C32	114.7(4)
C11	C10	C18	121.8(4)	C33	C34	C35	115.8(5)
C12	C11	C10	120.9(4)				

Table S7 Torsion Angles for mo_DKO1396_0m_5.

A	B	C	D	Angle/°	A	B	C	D	Angle/°
Ni1	N1	C8	C9	-179.9(4)	C10	C11	C12	N1	0.6(8)
Ni1	N1	C12	C11	179.8(4)	C10	C11	C12	C13	-179.8(5)
Ni1	N1	C12	C13	0.1(5)	C10	C18	C19	C20	174.8(4)
Ni1	N2	C13	C12	0.7(5)	C11	C10	C18	C19	79.6(6)
Ni1	N2	C13	C14	-178.3(4)	C11	C12	C13	N2	179.8(5)
Ni1	N2	C17	C16	179.8(4)	C11	C12	C13	C14	-1.2(8)
Ni1	C1	C2	C3	-164.3(4)	C12	N1	C8	C9	-0.3(8)
Ni1	C1	C2	C7	14.7(6)	C12	C13	C14	C15	179.4(5)
Cl1	Ni1	C1	O1	93.0(4)	C13	N2	C17	C16	-0.5(7)
Cl1	Ni1	C1	C2	-91.9(4)	C13	C14	C15	C16	-0.2(7)
O1	C1	C2	C3	10.8(7)	C13	C14	C15	C27	178.3(5)
O1	C1	C2	C7	-170.2(4)	C14	C15	C16	C17	1.6(7)
N1	C8	C9	C10	-0.3(8)	C14	C15	C27	C28	6.0(7)
N1	C12	C13	N2	-0.5(6)	C15	C16	C17	N2	-1.3(8)

Table S7 Torsion Angles for mo_DKO1396_0m_5.

A	B	C	D	Angle/°	A	B	C	D	Angle/°
N1	C12	C13	C14	178.5(4)	C15	C27	C28	C29	-177.3(4)
N2	Ni1	C1	O1	-90.4(4)	C16	C15	C27	C28	-175.6(5)
N2	Ni1	C1	C2	84.7(4)	C17	N2	C13	C12	-179.0(5)
N2	C13	C14	C15	-1.7(8)	C17	N2	C13	C14	2.0(7)
C1	C2	C3	C4	178.5(4)	C18	C10	C11	C12	179.8(5)
C1	C2	C7	C6	-178.8(5)	C18	C19	C20	C21	176.6(4)
C2	C3	C4	C5	0.8(8)	C19	C20	C21	C22	66.2(6)
C3	C2	C7	C6	0.2(7)	C20	C21	C22	C23	169.2(5)
C3	C4	C5	C6	-0.9(8)	C21	C22	C23	C24	-179.6(5)
C4	C5	C6	C7	0.6(8)	C22	C23	C24	C25	-177.3(5)
C5	C6	C7	C2	-0.2(8)	C23	C24	C25	C26	-178.1(5)
C7	C2	C3	C4	-0.5(7)	C27	C15	C16	C17	-176.9(5)
C8	N1	C12	C11	0.1(7)	C27	C28	C29	C30	-176.1(4)
C8	N1	C12	C13	-179.5(4)	C28	C29	C30	C31	-57.0(6)
C8	C9	C10	C11	1.0(7)	C29	C30	C31	C32	-65.3(6)
C8	C9	C10	C18	180.0(5)	C30	C31	C32	C33	173.6(4)
C9	C10	C11	C12	-1.2(8)	C31	C32	C33	C34	-178.2(4)
C9	C10	C18	C19	-99.3(6)	C32	C33	C34	C35	-178.2(5)

Table S8 Hydrogen Atom Coordinates (Å×10^4) and Isotropic Displacement Parameters (Å^2×10^3) for mo_DKO1396_0m_5.

Atom	*x*	*y*	*z*	U(eq)
H3	5235.05	5727.98	1996.38	38
H4	3479.49	5442.58	1113.73	41
H5	110.78	5982.03	1199.26	39
H6	-1545.11	6757.37	2175.64	38
H7	192.17	7031.61	3068.78	34
H8	845.81	8699.85	5030.92	30
H9	-395.42	8698.1	6151.57	33
H11	987.42	5130.05	6340.21	30
H14	2337.27	3590.03	5817.23	30
H16	4689.29	2465.39	4007.96	30
H17	4368.53	4391.32	3544.41	29
H18A	-1417.68	6283.9	7202.95	37
H18B	-1601.04	7656.3	7177.06	37

Table S8 Hydrogen Atom Coordinates (Å×10^4) and Isotropic Displacement Parameters (Å^2×10^3) for mo_DKO1396_0m_5.

**Atom**	***x***	***y***	***z***	**U(eq)**
H19A	1715.39	5766.65	7617.21	39
H19B	1681.61	7126.05	7535.42	39
H20A	-866.65	6147.86	8470.24	40
H20B	-774.79	7479.12	8405.72	40
H21A	2298.71	5492.36	8899.2	43
H21B	755.7	6277.54	9396.14	43
H22A	3664.56	7025.25	8515.34	47
H22B	1948.53	7948.09	8880.78	47
H23A	4735.67	6045.74	9610.72	39
H23B	3014.42	6959.71	9978.05	39
H24A	6077.91	7621.46	9151.02	41
H24B	4391.91	8503.65	9552.46	41
H25A	7281.38	6579.08	10205.68	49
H25B	5564.72	7425.48	10616.96	49
H26A	8347.4	8044.26	10634.45	64
H26B	6836.27	9053.17	10169.17	64
H26C	8609.9	8157.89	9809.42	64
H27A	5309.18	1128.53	5280.56	32
H27B	3325.17	1121.05	4990.62	32
H28A	3623.5	1700.28	6336.28	31
H28B	1670.49	1626.89	6044.44	31
H29A	3159.46	-431.49	5963.78	33
H29B	5027.9	-339.23	6302.76	33
H30A	1313.22	-96.06	6990.52	40
H30B	3170.35	-1194.52	7142.21	40
H31A	2849.16	1118.92	7450.58	36
H31B	2481.33	79.88	8012.04	36
H32A	6144.53	-45.32	7310.53	34
H32B	5728.83	-982.78	7933.36	34
H33A	5491.45	1399.43	8081.24	37
H33B	5146.09	438.2	8701.73	37
H34A	8730.45	332.19	7966.7	49
H34B	8399.3	-658.63	8569.48	49
H35A	9921.74	501.09	9023.81	61
H35B	7777.77	753.77	9367.1	61
H35C	8300.57	1679.11	8756.54	61

Experimental

A suitable crystal was selected and measured on a Bruker APEX-II CCD diffractometer. The crystal was kept at 99.96 K during data collection. Using Olex2 [Dolomanov, O.V., Bourhis, L.J., Gildea, R.J, Howard, J.A.K. & Puschmann, H., *J. Appl. Cryst*. **2009,** *42*, 339-341], the structure was solved with the SHELXT [Sheldrick, G.M. *Acta Cryst*. **2015,** A71, 3-8] structure solution program using Intrinsic Phasing and refined with the XL [Sheldrick, G.M. *Acta Cryst.* **2008,** A64, 112-122] refinement package using Least Squares minimisation.

Crystal structure determination of [mo_DKO1396_0m_5]

Crystal Data for $C_{35}H_{49}ClN_2NiO$ (M =607.92 g/mol): triclinic, space group P-1 (no. 2), a = 7.2452(18) Å, b = 11.945(3) Å, c = 19.588(5) Å, α = 81.348(3)°, β = 84.948(3)°, γ = 73.127(3)°, V = 1602.0(7) Å^3, Z = 2, T = 99.96 K, μ(MoKα) = 0.718 mm^{-1}, $Dcalc$ = 1.260 g/cm^3, 5902 reflections measured (3.594° ≤ 2Θ ≤ 51.79°), 5902 unique (R_{int} = ?, R_{sigma} = 0.0948) which were used in all calculations. The final R_1 was 0.0784 (I > 2σ(I)) and wR_2 was 0.2161 (all data).

Refinement model description

Number of restraints - 0, number of constraints - unknown.

Details:

1. Twinned data refinement
 Scales: 0.546(3)
 0.454(3)
2. Fixed Uiso
 At 1.2 times of:
  All C(H) groups, All C(H,H) groups
 At 1.5 times of:
  All C(H,H,H) groups
3.a Secondary CH2 refined with riding coordinates:
 C18(H18A,H18B), C19(H19A,H19B), C20(H20A,H20B), C21(H21A,H21B), C22(H22A, H22B), C23(H23A,H23B), C24(H24A,H24B), C25(H25A,H25B), C27(H27A,H27B), C28(H28A,H28B), C29(H29A,H29B), C30(H30A,H30B), C31(H31A,H31B), C32(H32A,H32B),

C33(H33A,H33B), C34(H34A,H34B)

3.b Aromatic/amide H refined with riding coordinates:

C3(H3), C4(H4), C5(H5), C6(H6), C7(H7), C8(H8), C9(H9), C11(H11), C14(H14), C16(H16), C17(H17)

3.c Idealised Me refined as rotating group:

C26(H26A,H26B,H26C), C35(H35A,H35B,H35C)

9. NMR Spectra

^{1}H NMR of **S1zc**

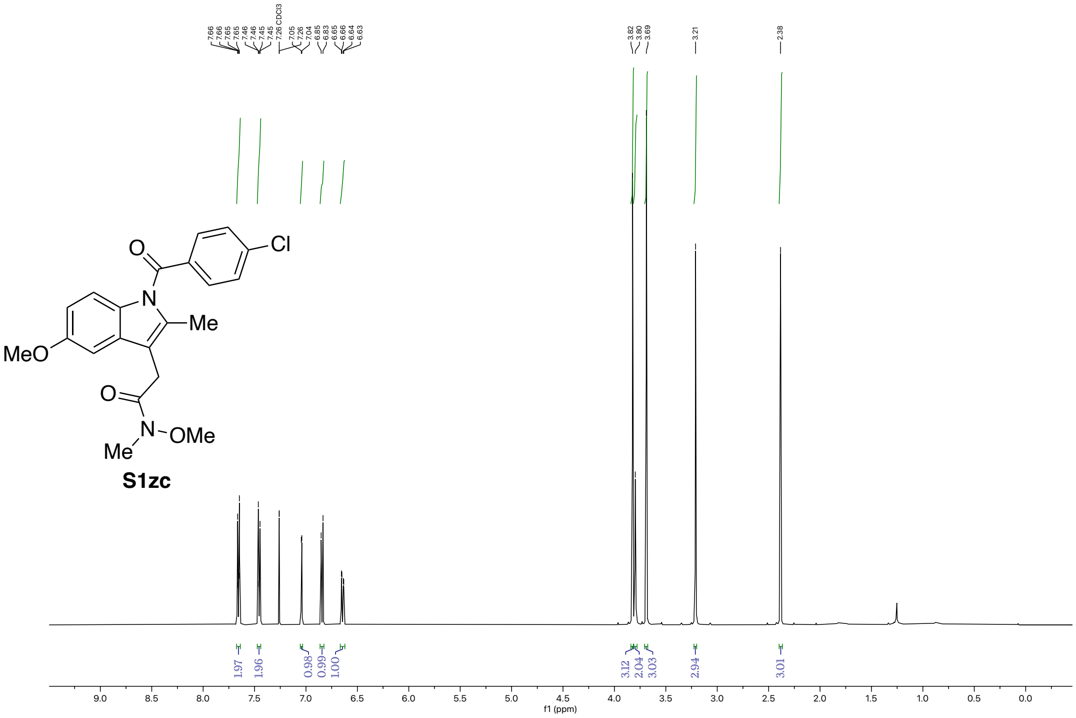

^{13}C NMR of **S1zc**

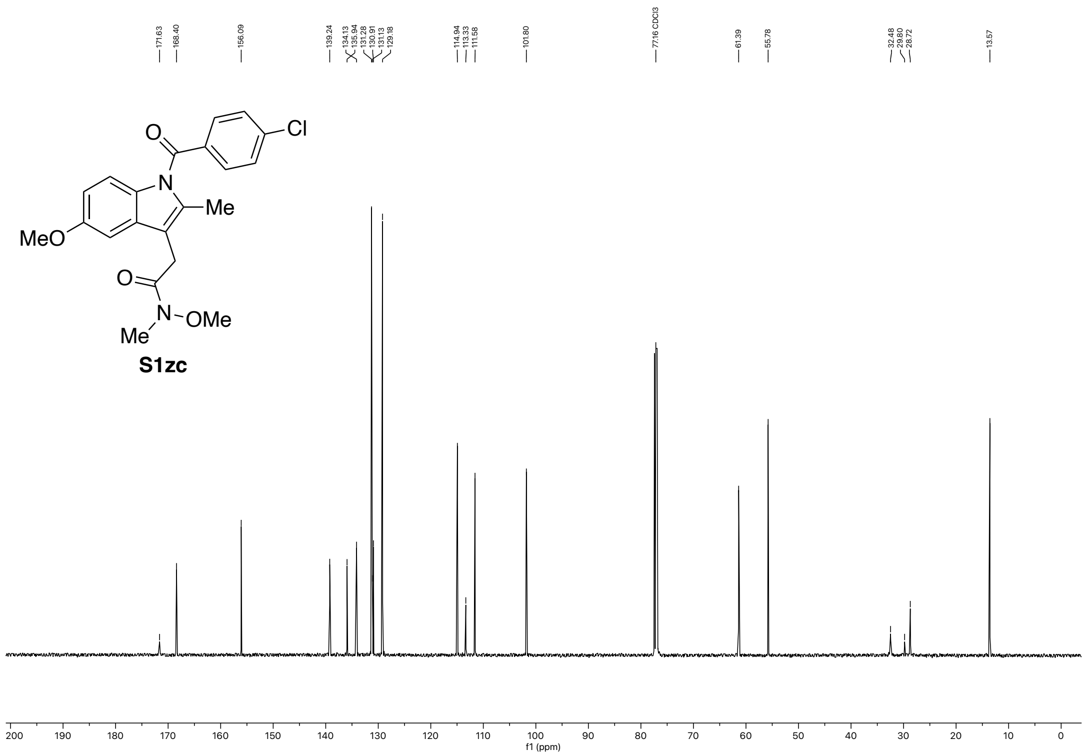

^{1}H NMR of **S1zd**

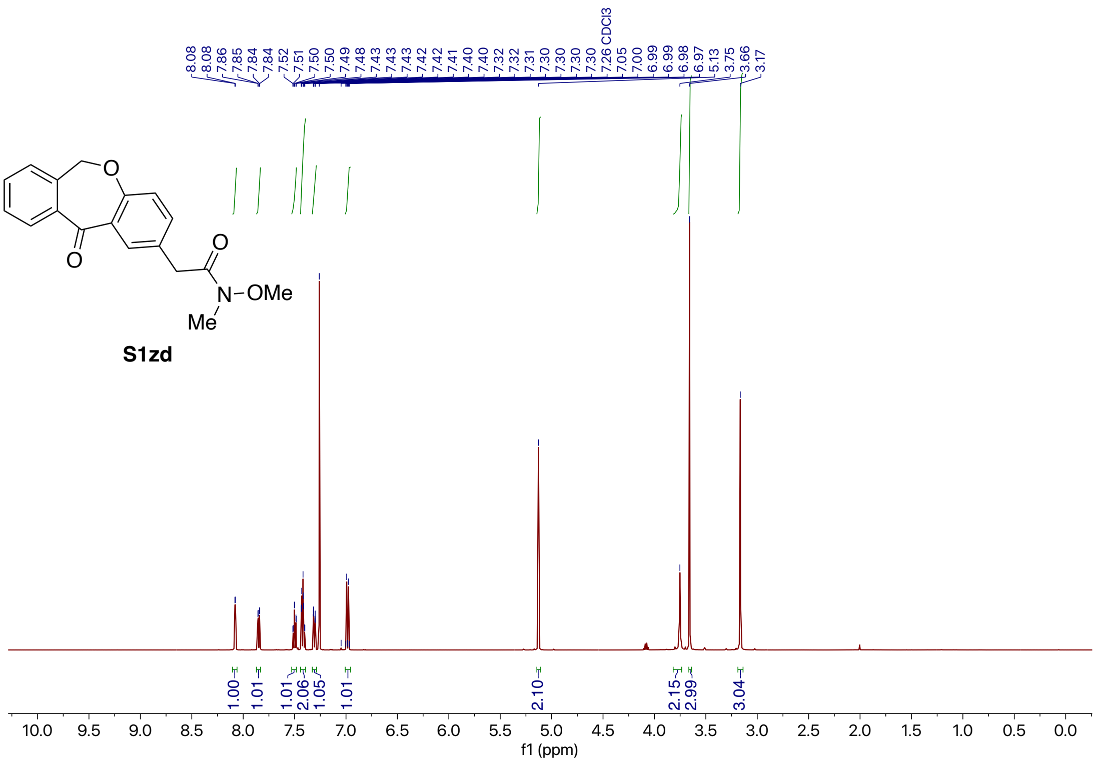

^{13}C NMR of **S1zd**

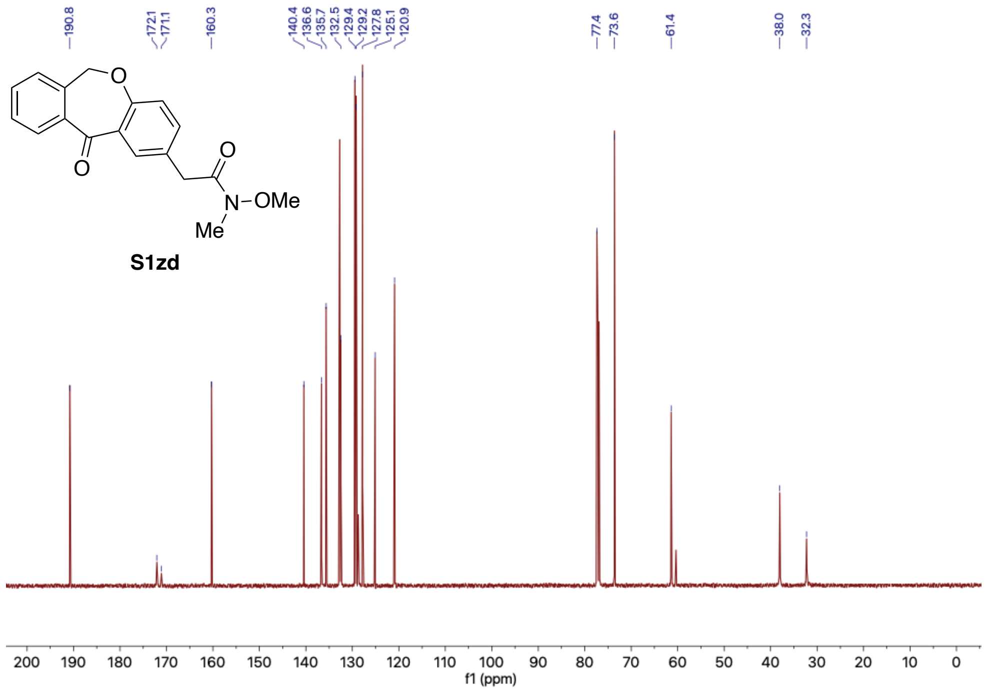

^{1}H NMR of **S1ze**

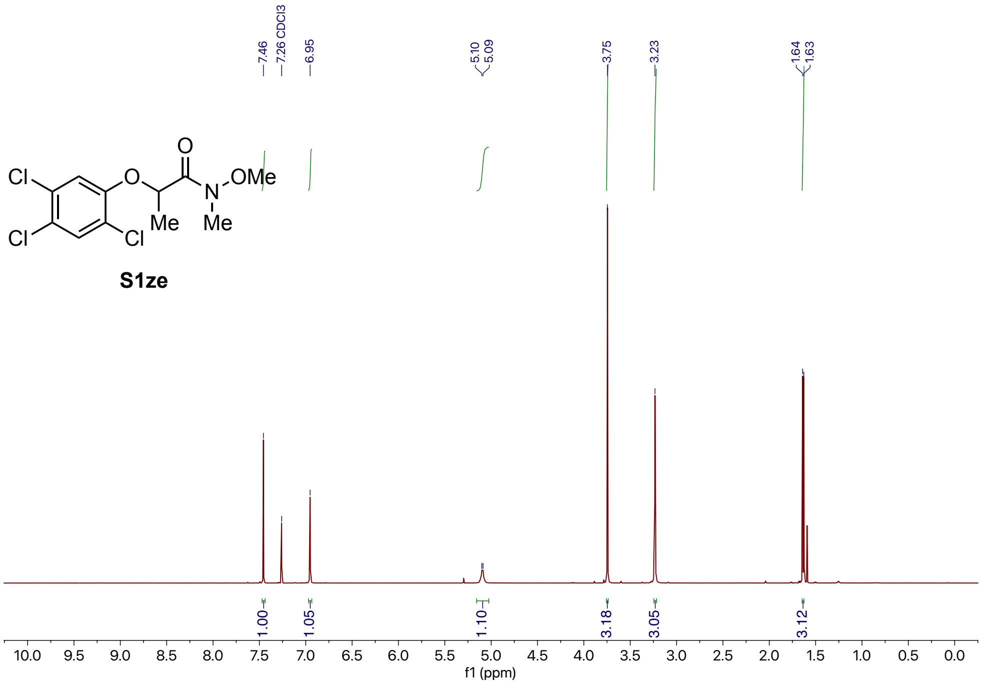

^{13}C NMR of **S1ze**

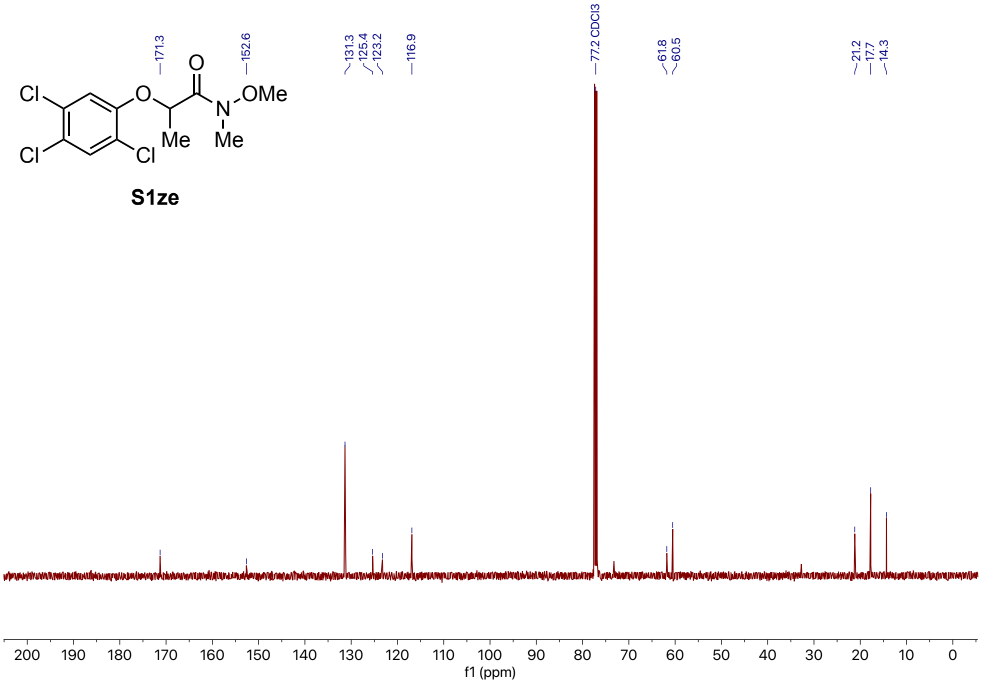

^{1}H NMR of **1zb**

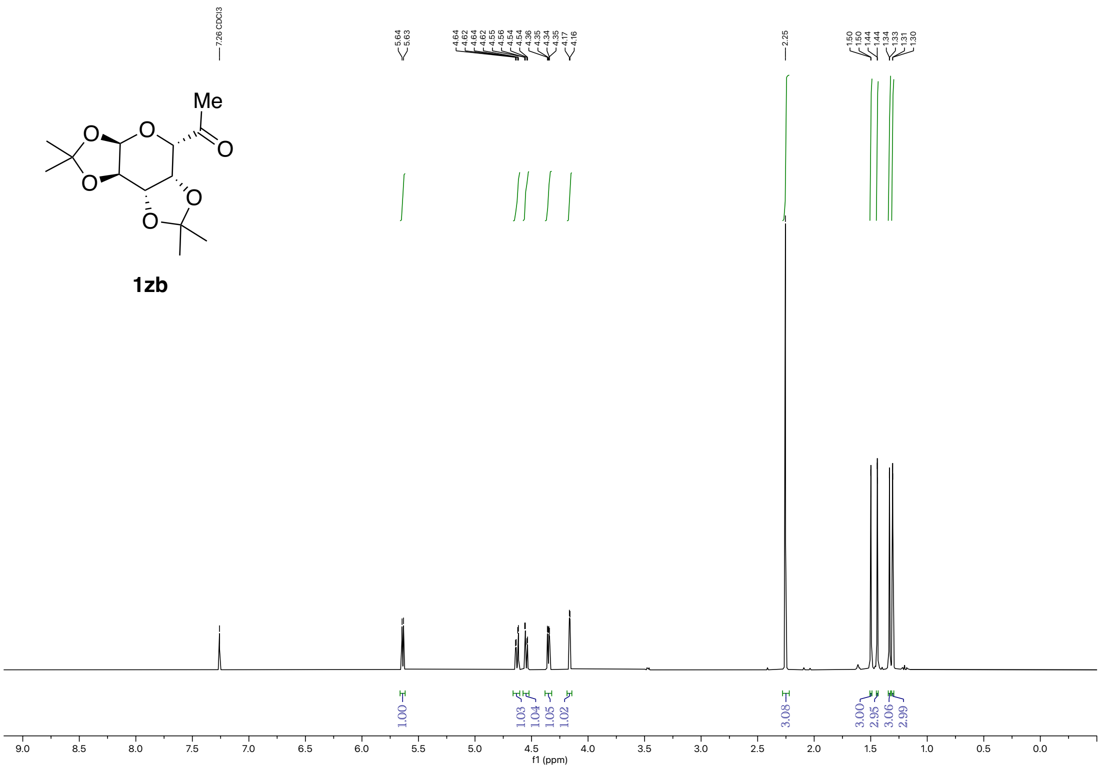

^{13}C NMR of **1zb**

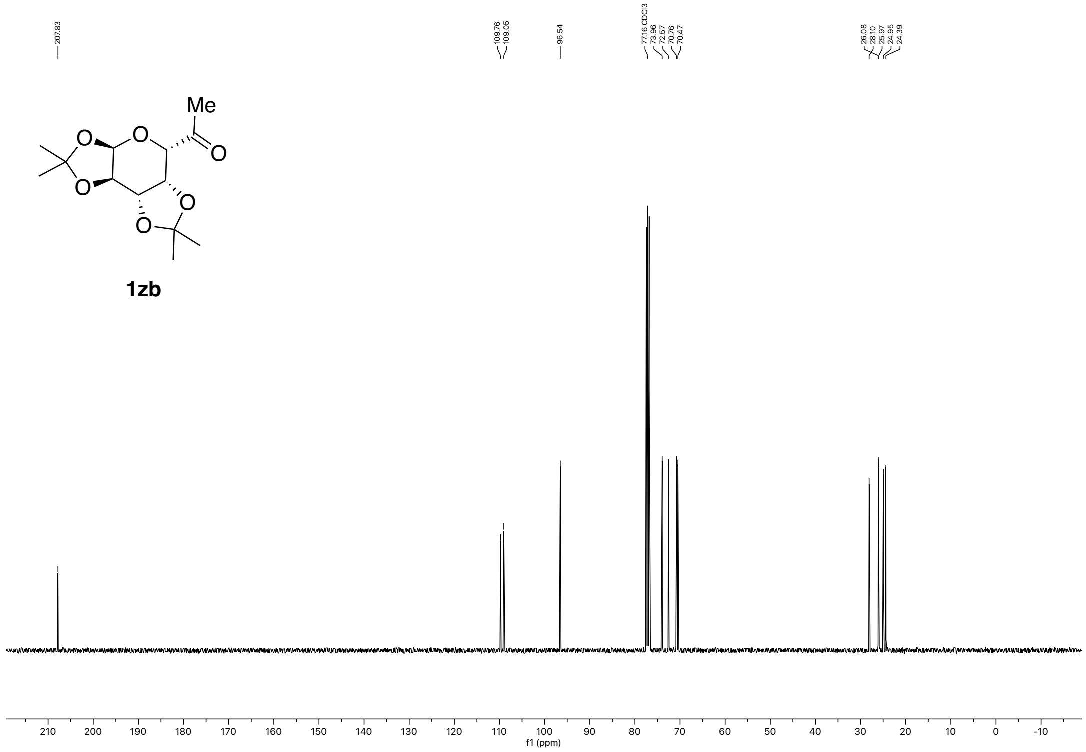

^{1}H NMR of **1zc**

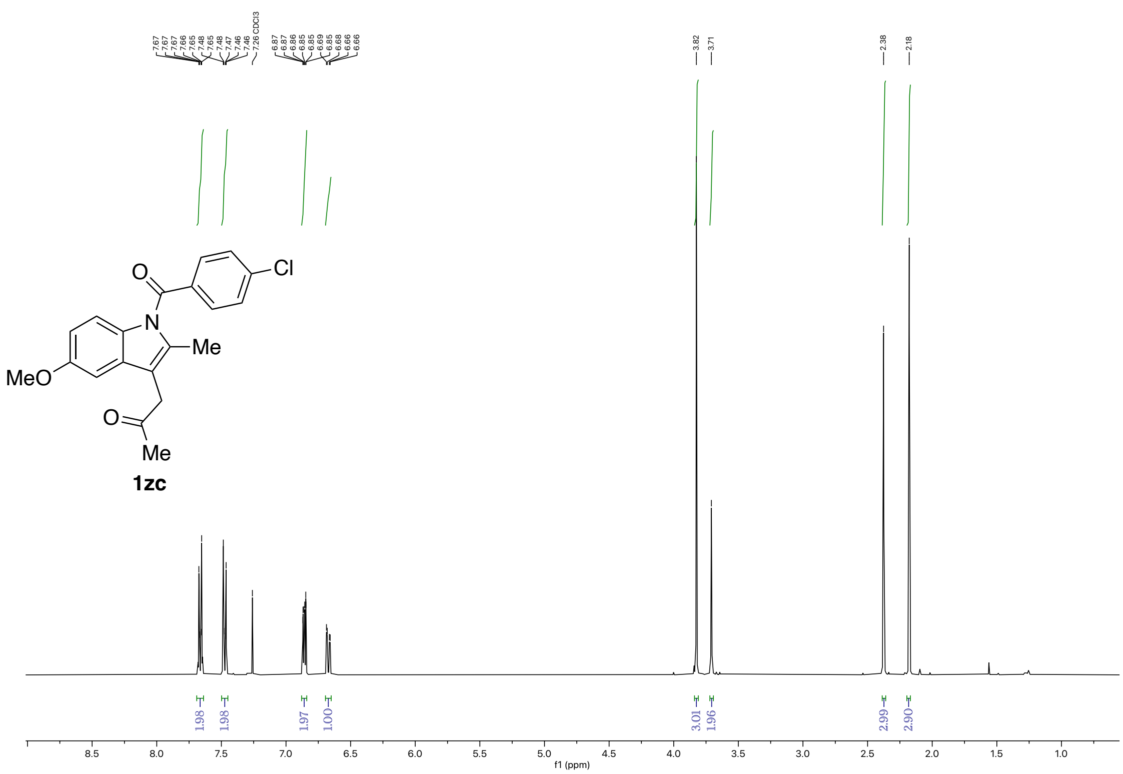

^{13}C NMR of **1zc**

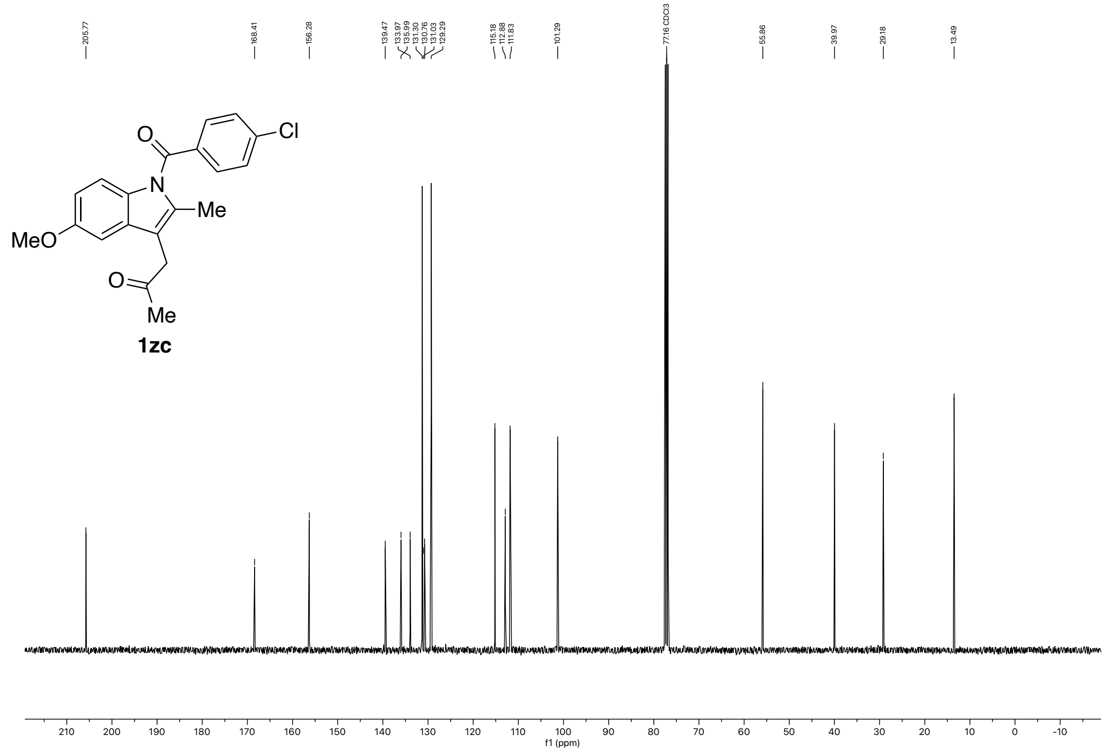

^{1}H NMR of **1zd**

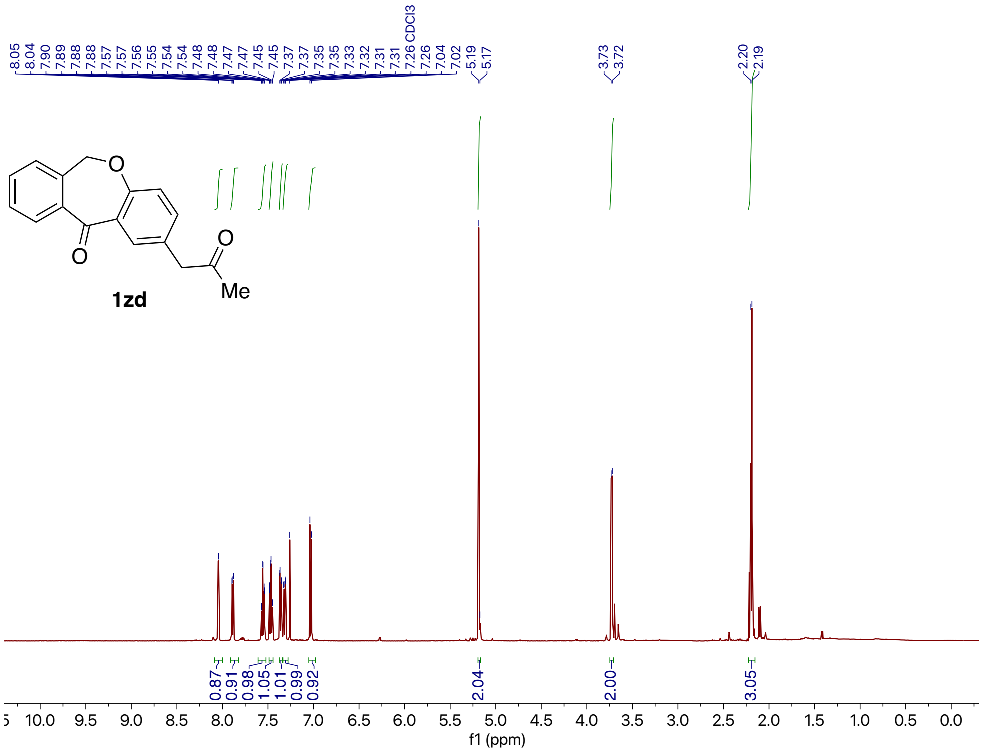

^{13}C NMR of **1zd**

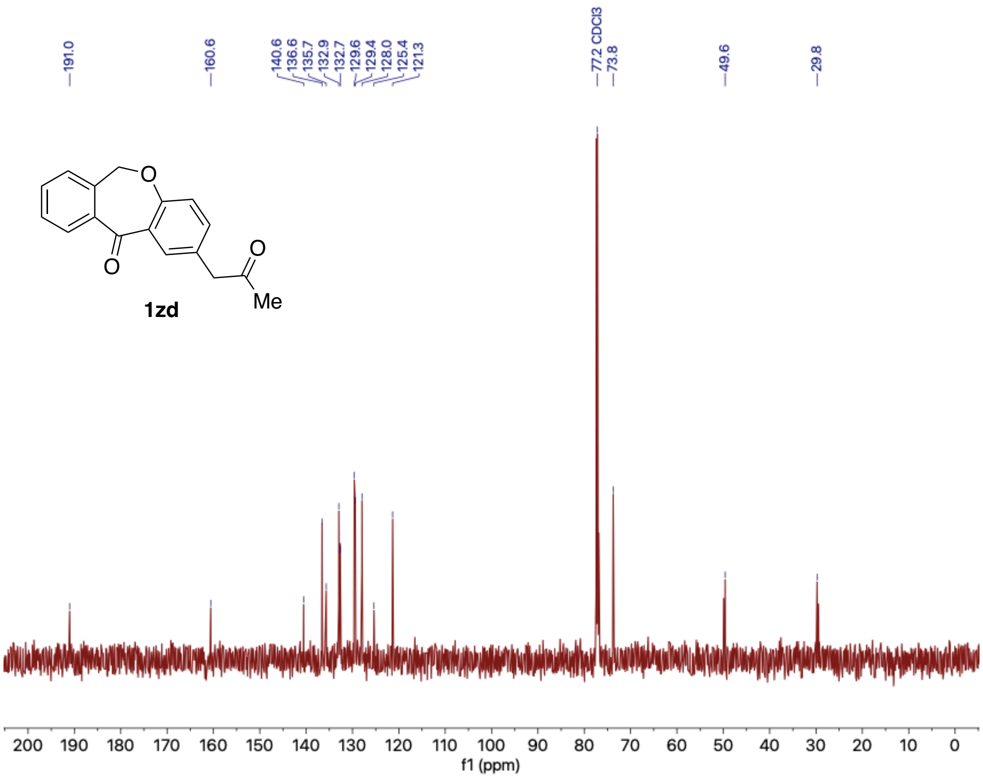

^1H NMR of **1ze**

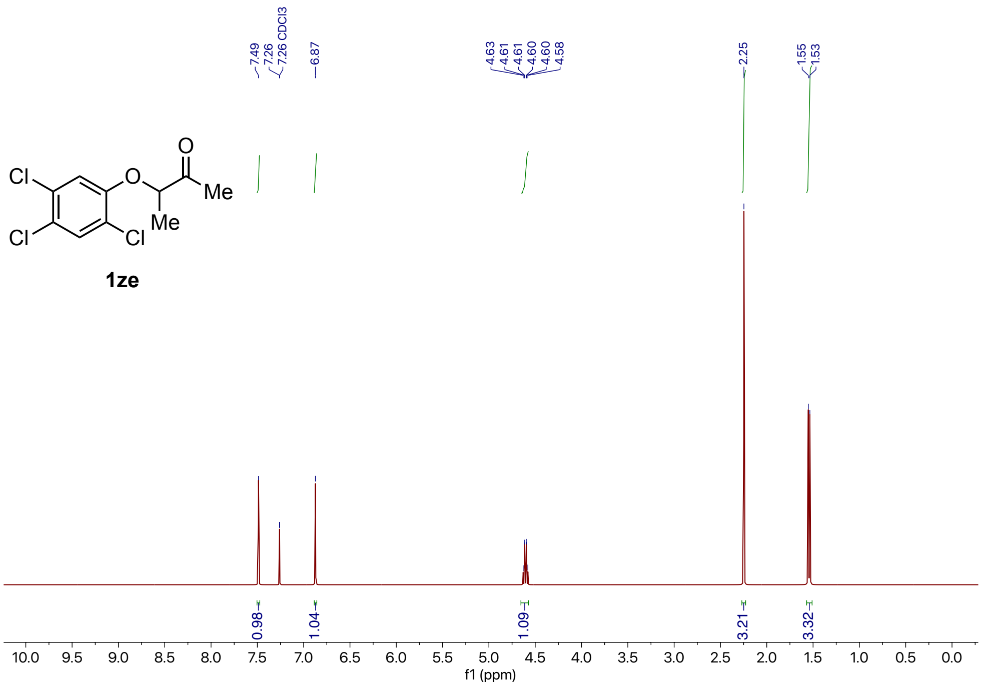

^{13}C NMR of **1ze**

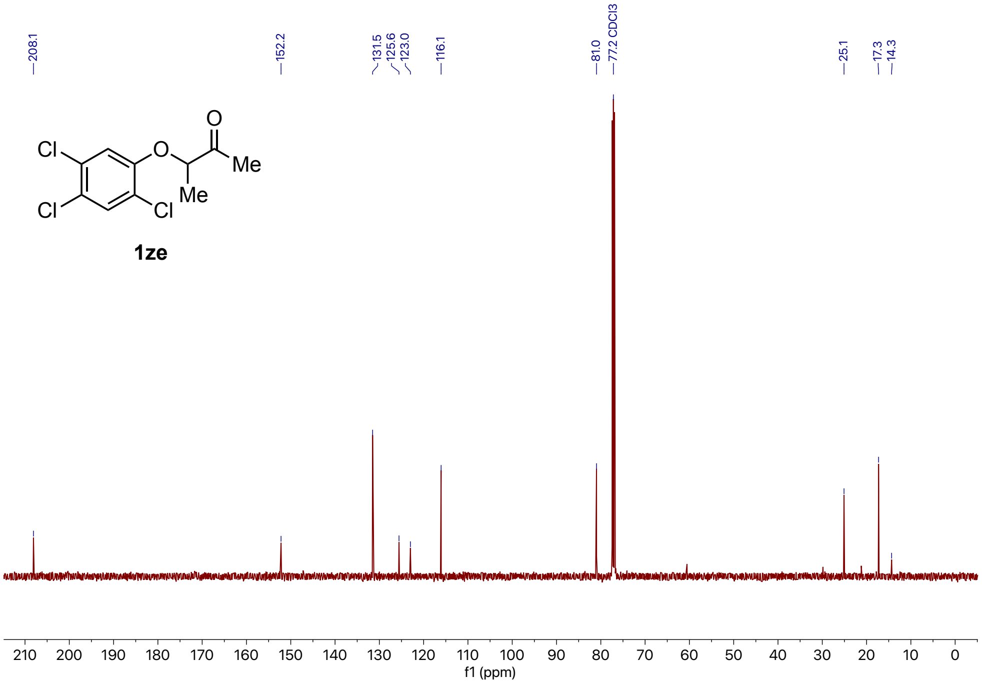

^{1}H NMR of **2d**

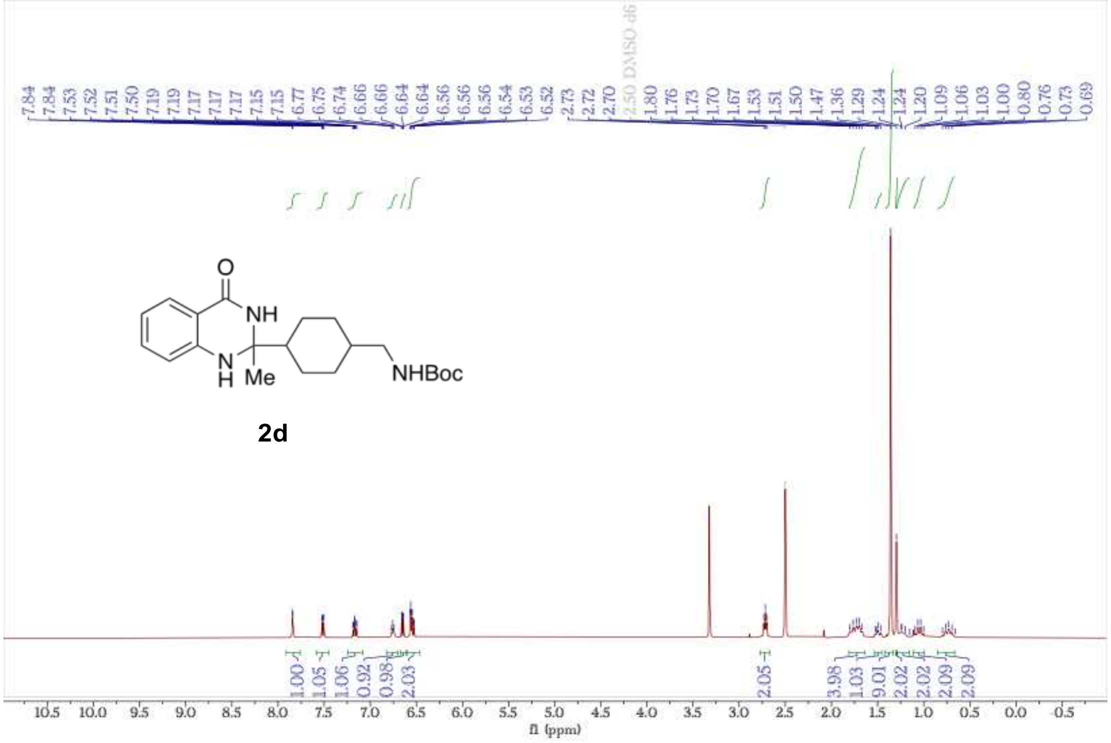

^{13}C NMR of **2d**

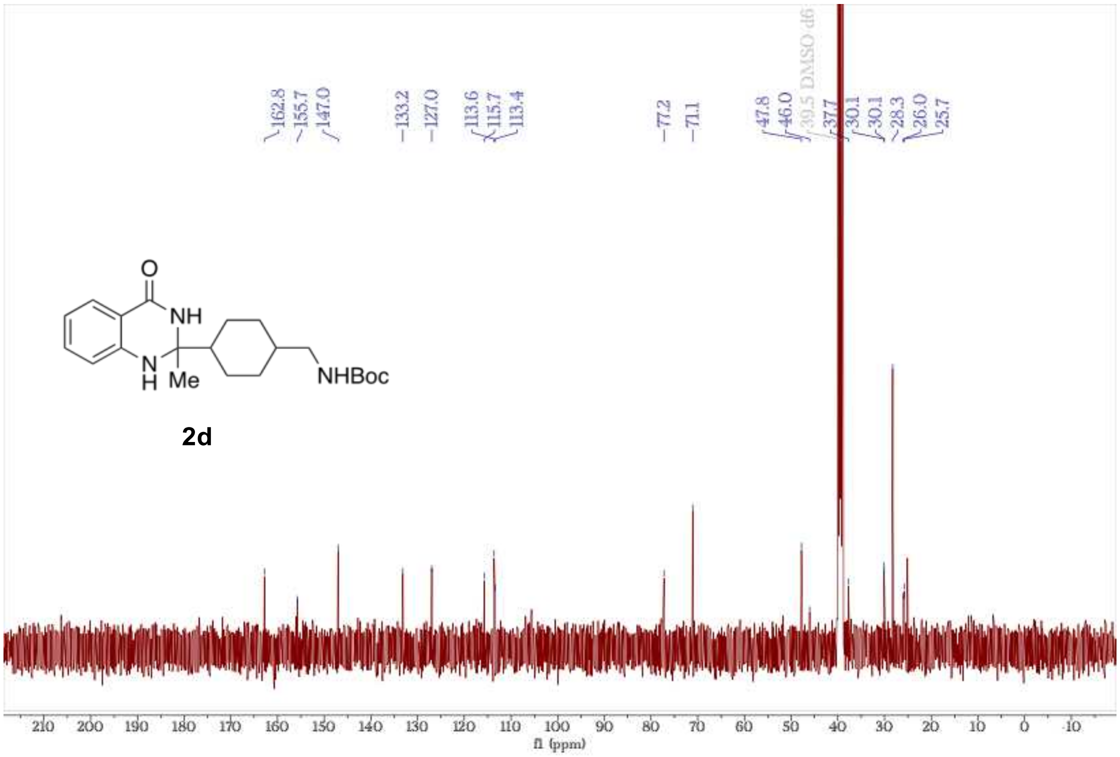

^{1}H NMR of **2e**

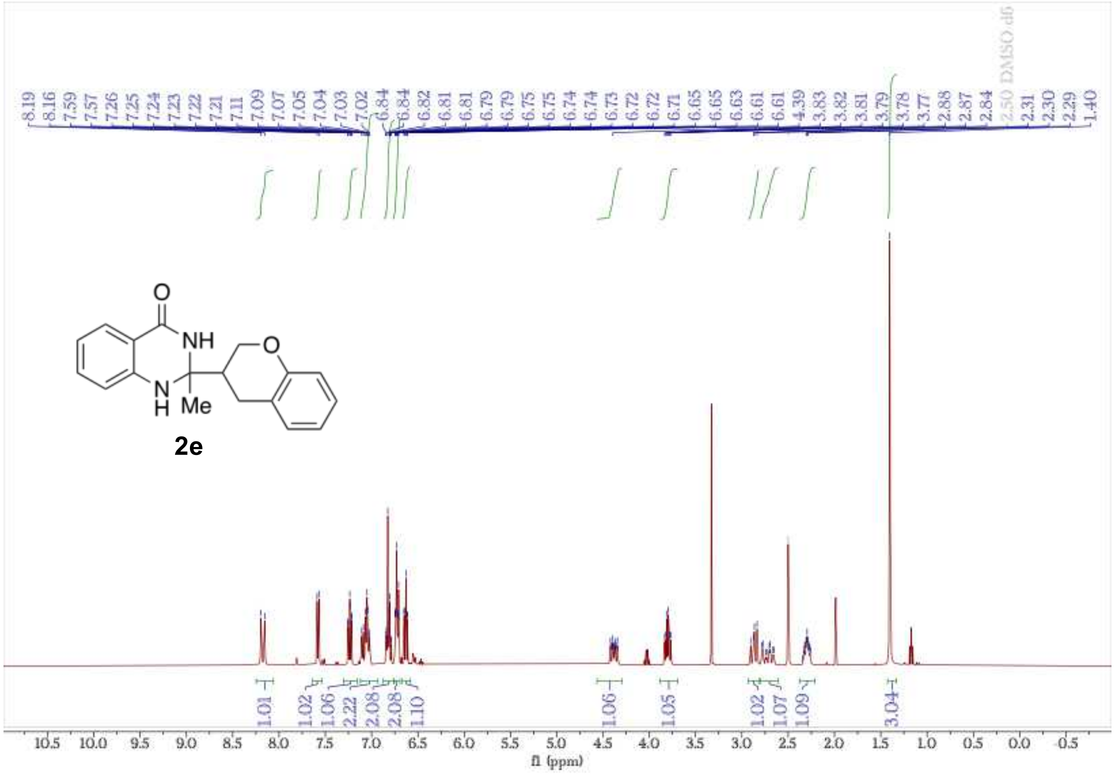

^{13}C NMR of **2e**

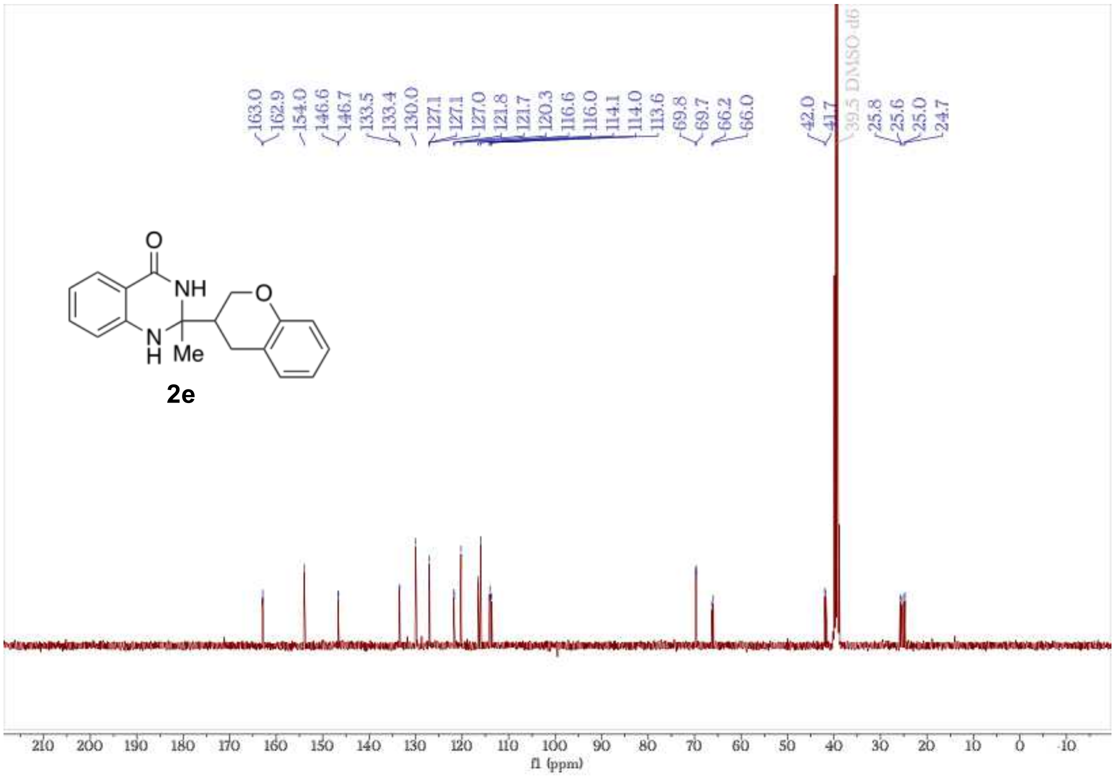

^{1}H NMR of **2h**

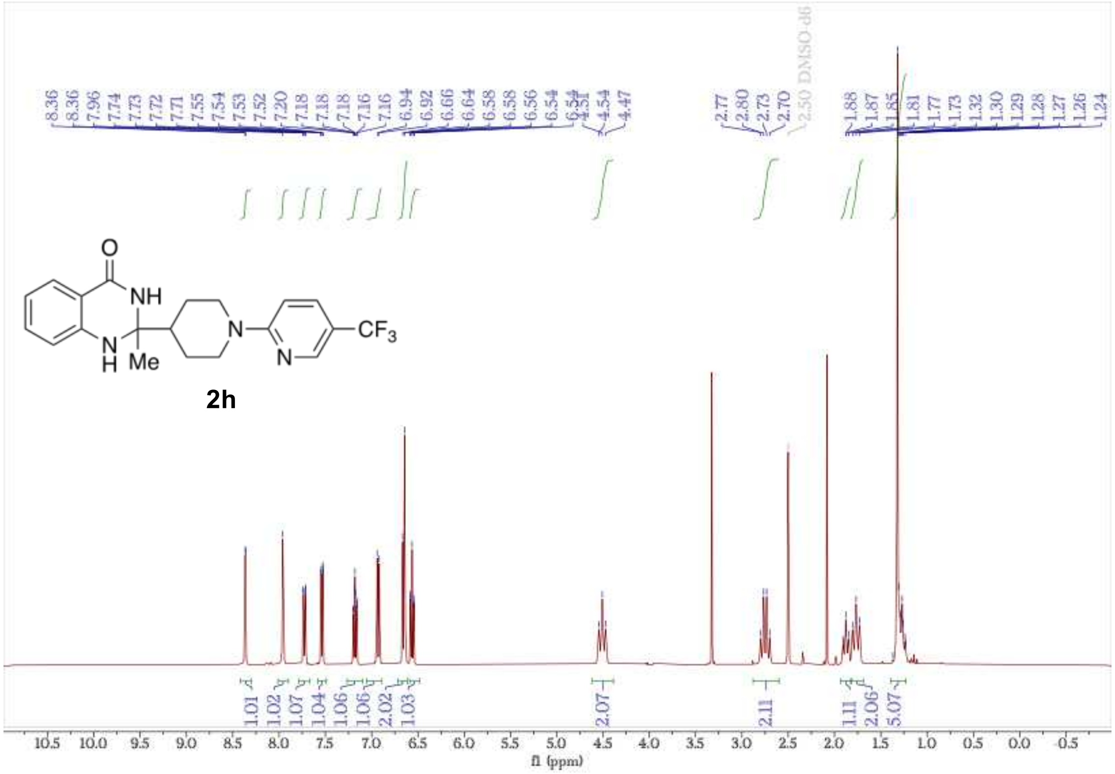

^{13}C NMR of **2h**

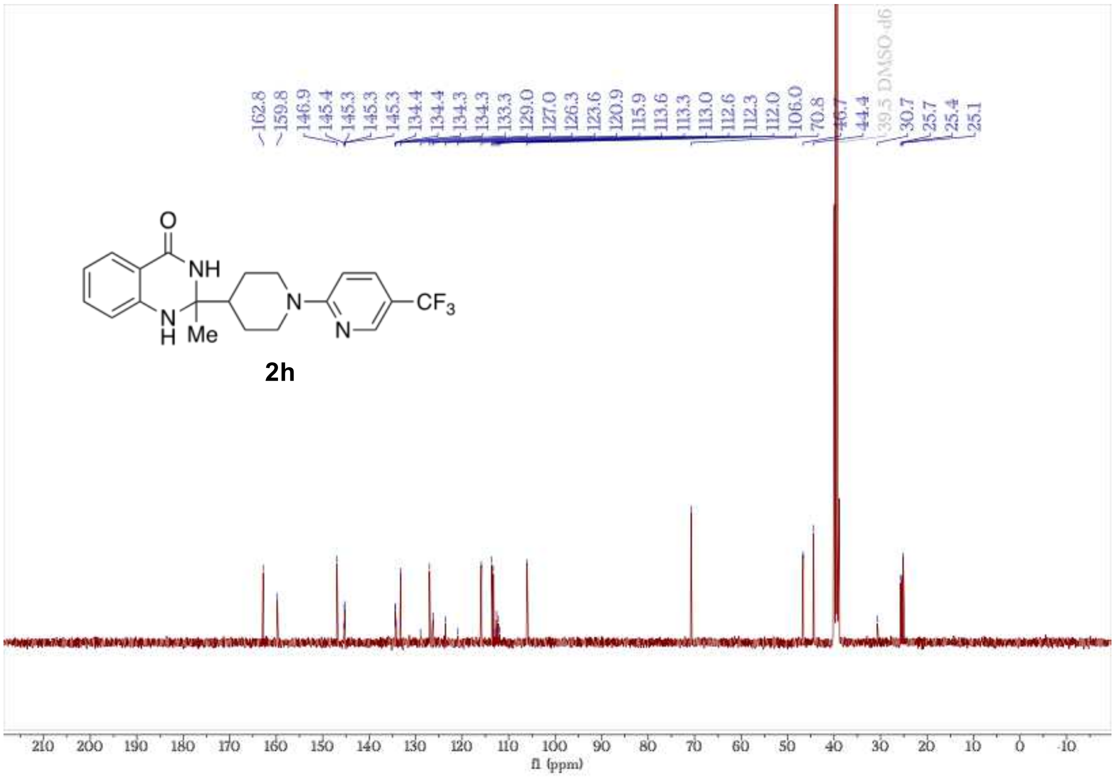

^{19}F NMR of **2h**

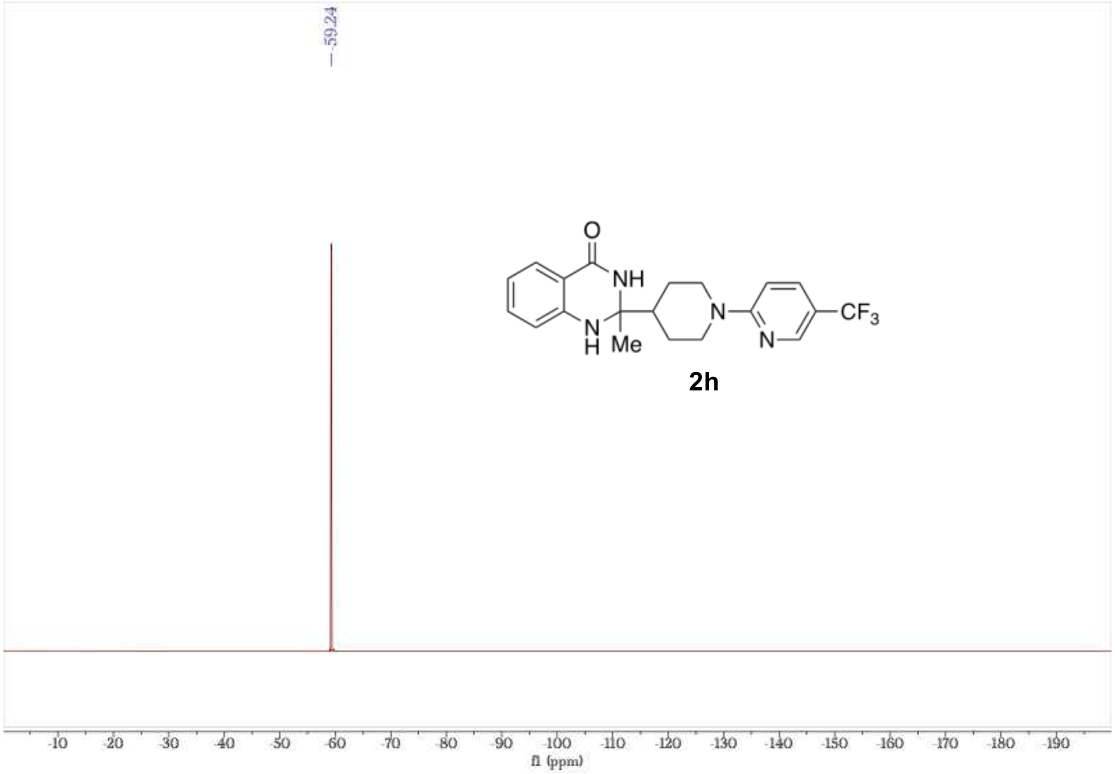

-59.24
2h
f1 (ppm)

^{1}H NMR of **2i**

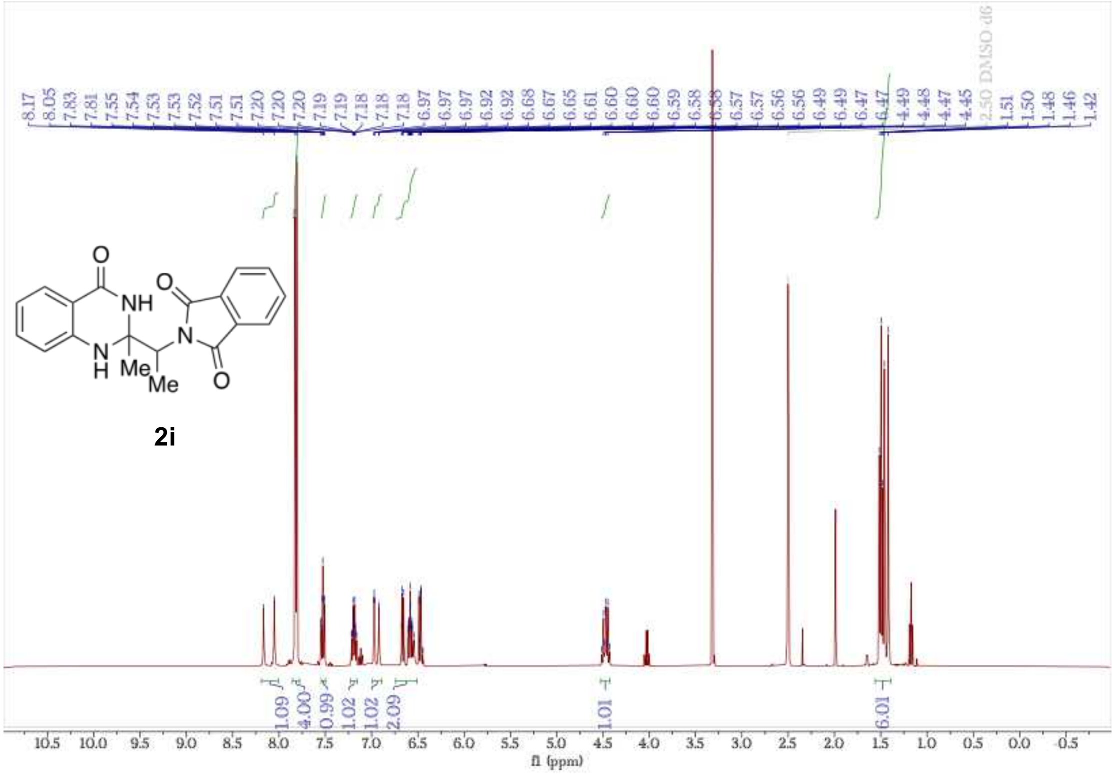

8.17 8.05 7.83 7.81 7.55 7.54 7.53 7.53 7.52 7.51 7.51 7.20 7.20 7.20 7.19 7.19 7.18 7.18 7.18 6.97 6.97 6.97 6.97 6.92 6.92 6.68 6.67 6.65 6.61 6.60 6.60 6.60 6.59 6.58 6.58 6.57 6.57 6.56 6.56 6.49 6.49 6.49 6.47 6.47 4.49 4.48 4.47 4.45 2.50 DMSO d6 1.51 1.50 1.48 1.46 1.42
2i
1.09 4.00 0.99 1.02 1.02 2.09 1.01 6.01
f1 (ppm)

^{13}C NMR of **2i**

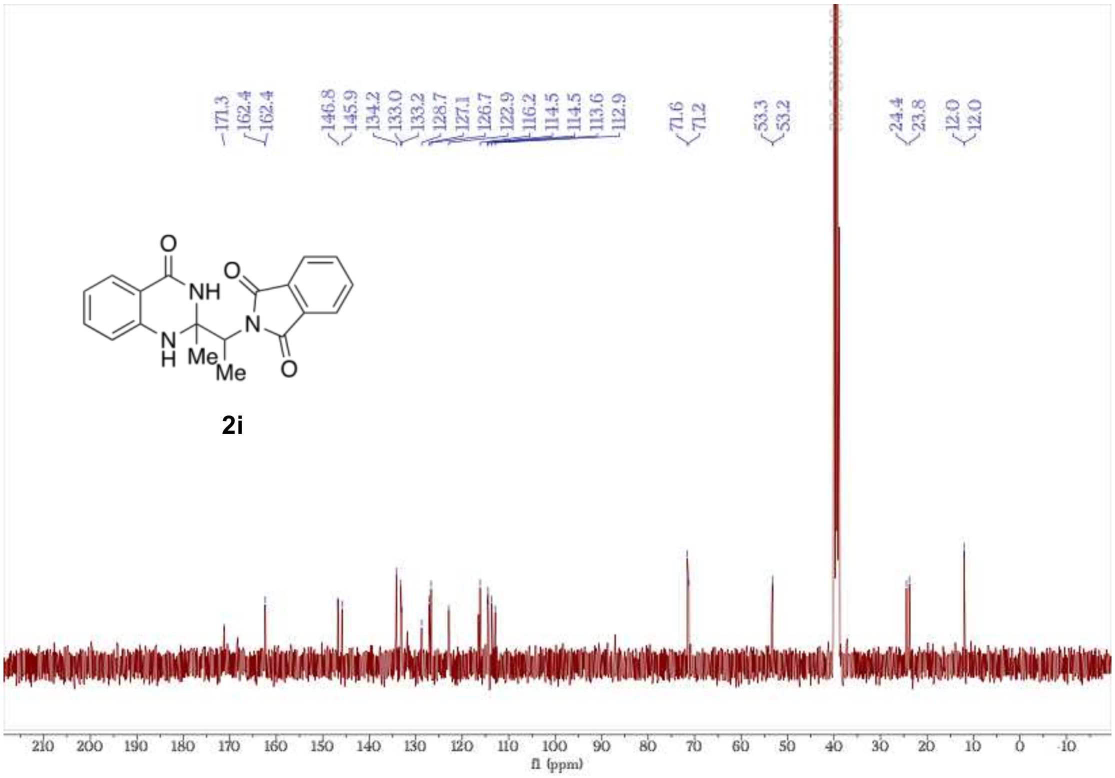

^{1}H NMR of **2j**

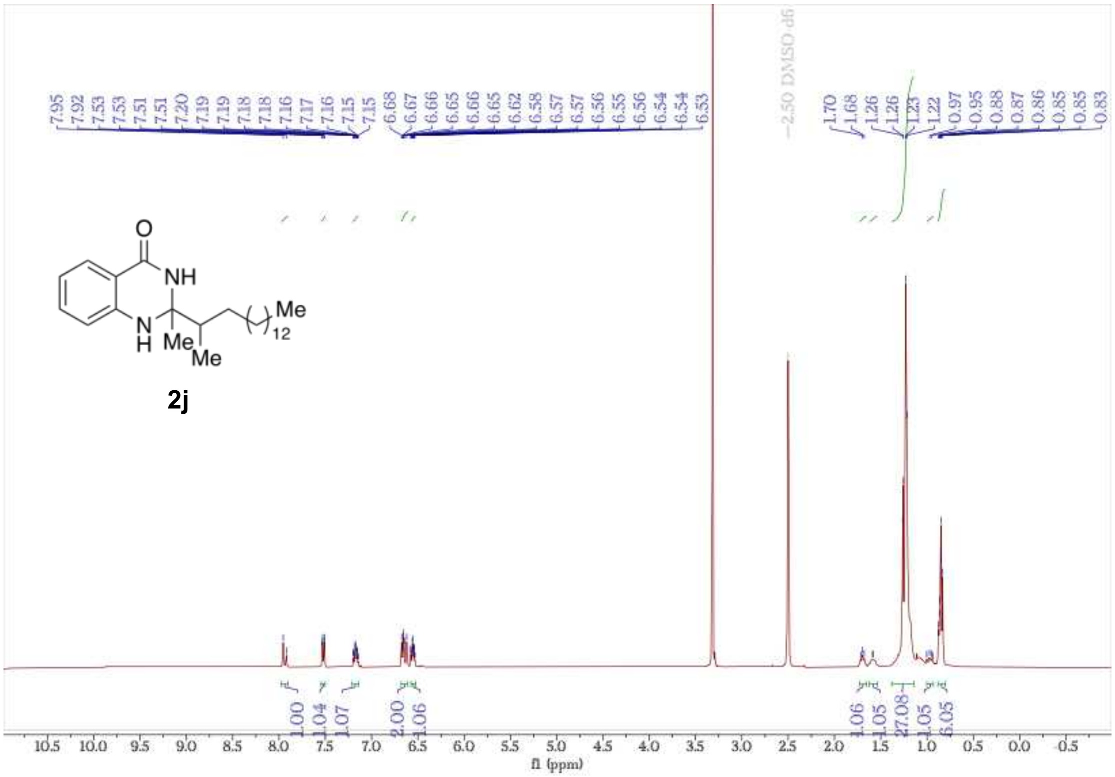

^{13}C NMR of **2j**

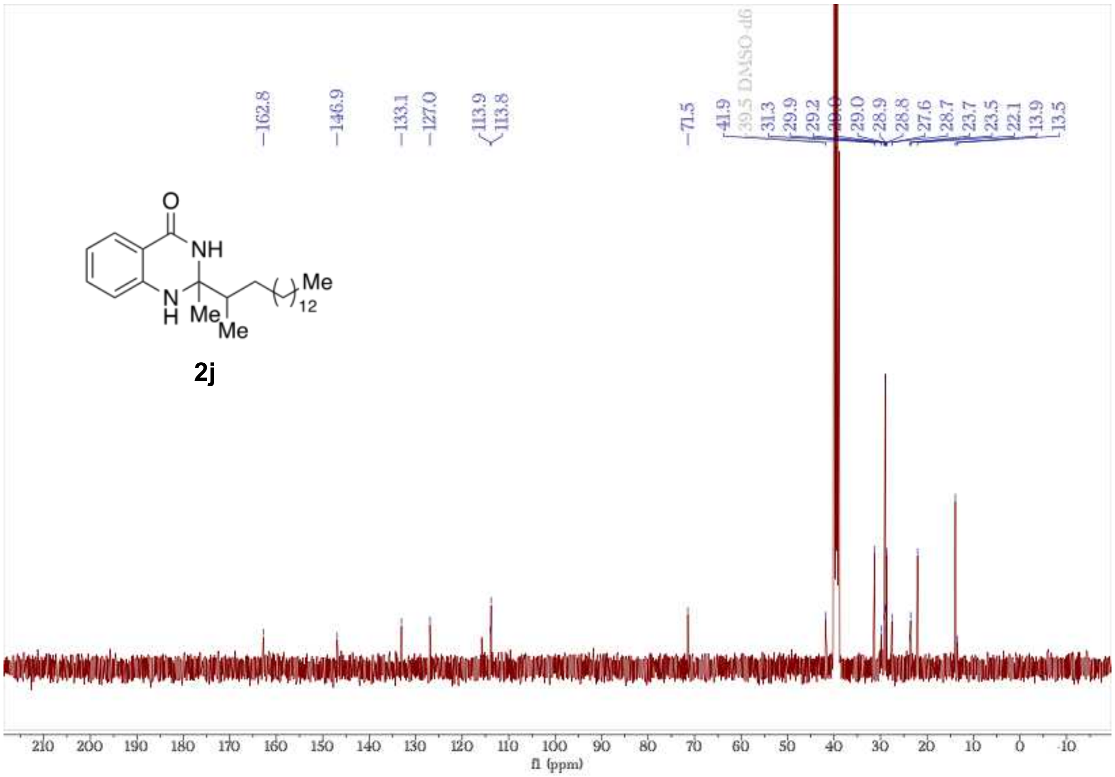

^{1}H NMR of **2l**

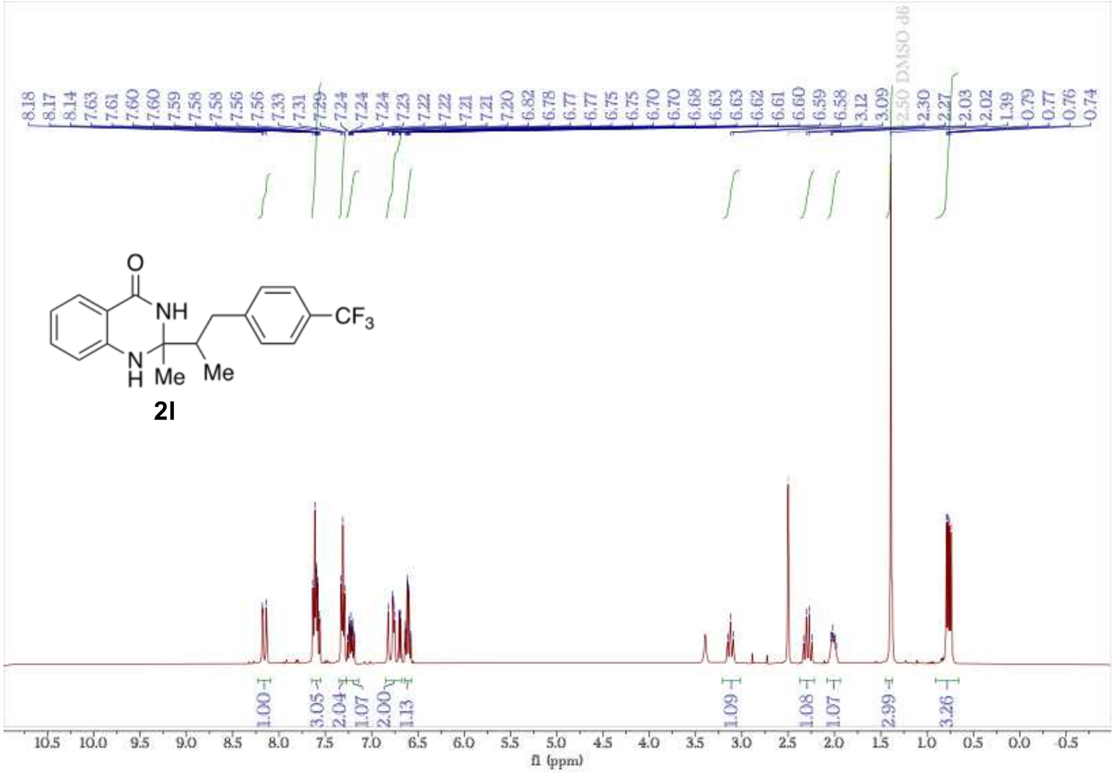

^{13}C NMR of **2l**

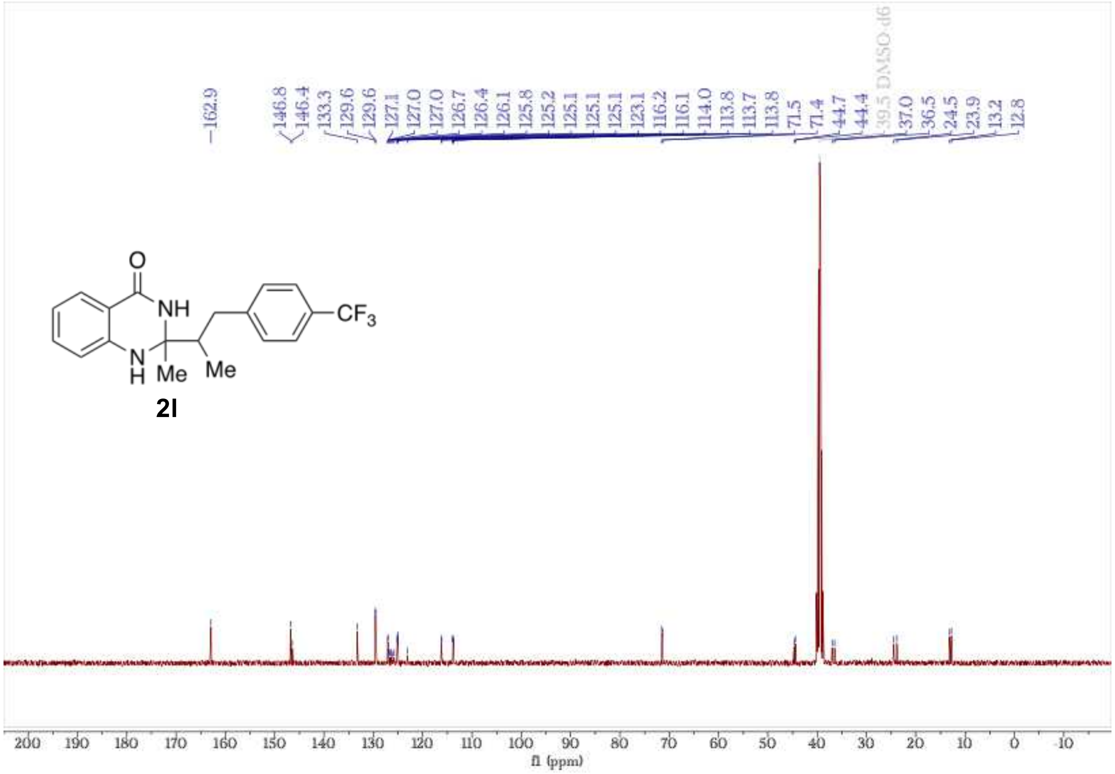

^{19}F NMR of **2l**

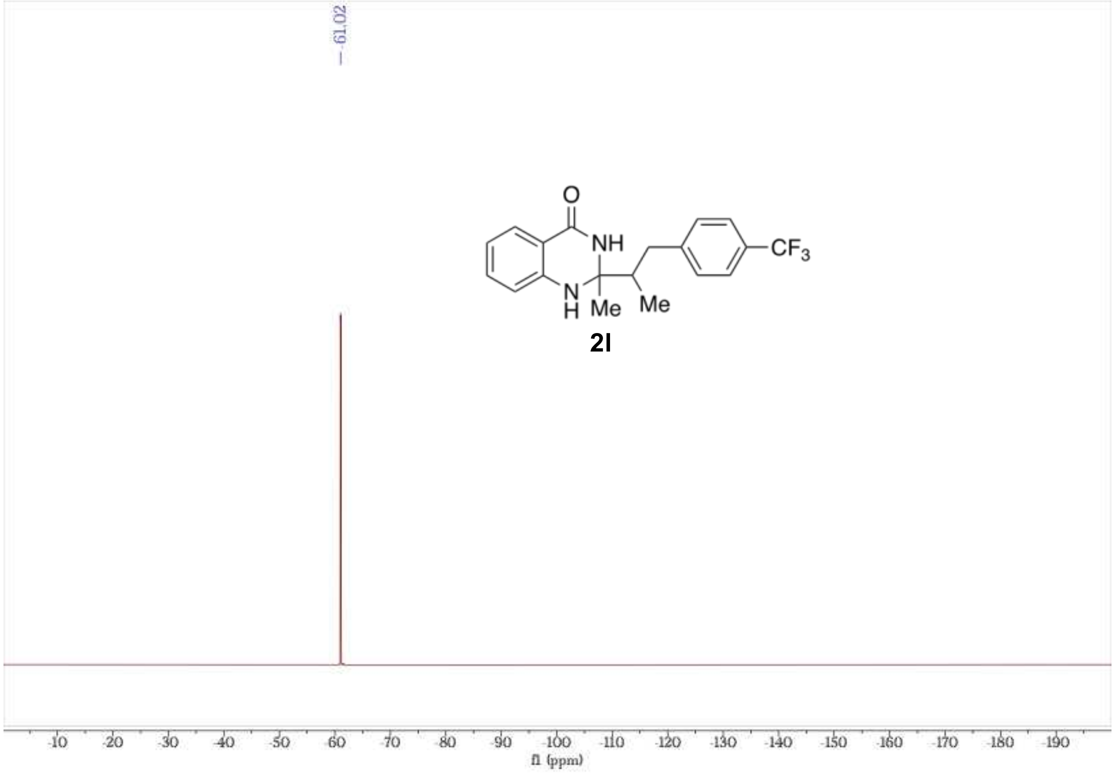

^{1}H NMR of **2o**

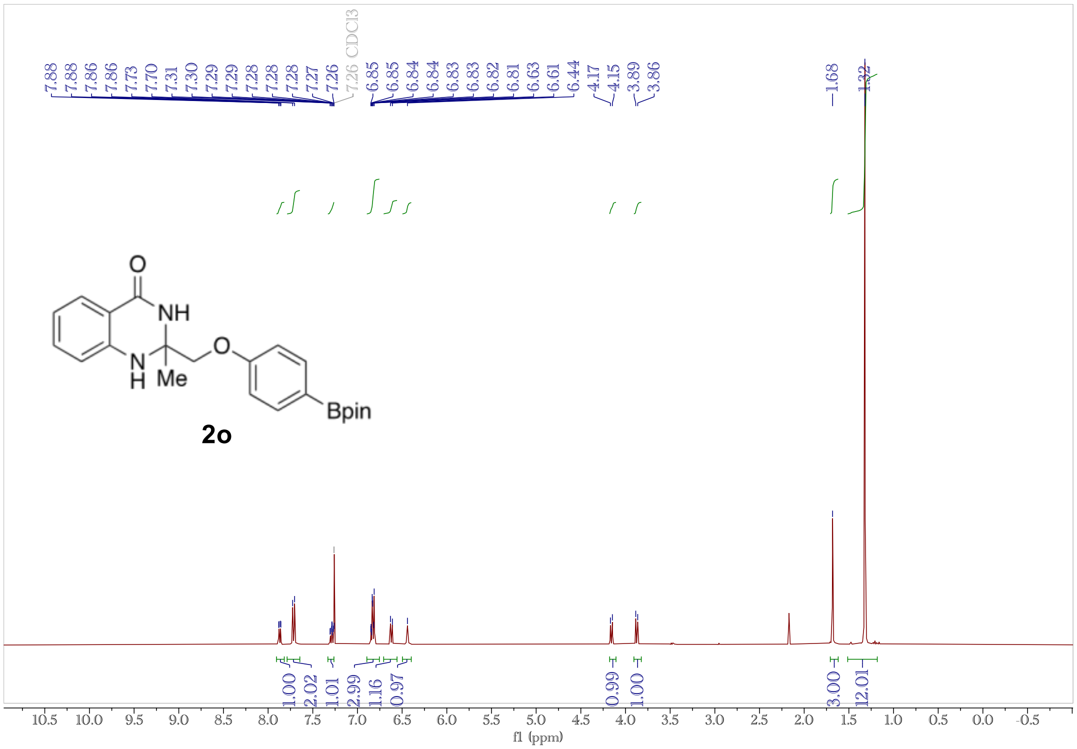
7.88 7.88 7.86 7.86 7.73 7.70 7.31 7.30 7.29 7.29 7.28 7.28 7.28 7.27 7.26
7.26 CDCl3
6.85 6.85 6.84 6.84 6.83 6.83 6.82 6.81 6.63 6.61 6.44
4.17 4.15 3.89 3.86
1.68
1.32
O
NH
O
N
H
Me
Bpin
2o
1.00 2.02 1.01 2.99 1.16 0.97
0.99 1.00
3.00 12.01
10.5 10.0 9.5 9.0 8.5 8.0 7.5 7.0 6.5 6.0 5.5 5.0 4.5 4.0 3.5 3.0 2.5 2.0 1.5 1.0 0.5 0.0 -0.5
f1 (ppm)

^{13}C NMR of **2o**

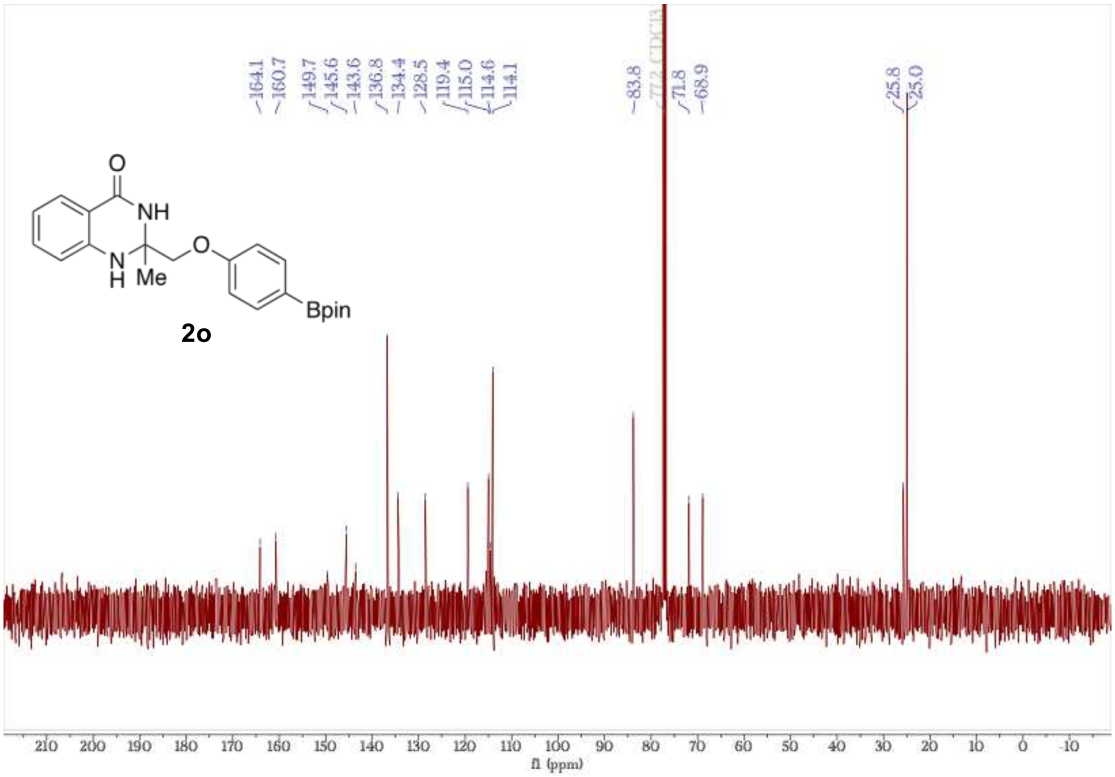
164.1 160.7 149.7 145.6 143.6 136.8 134.4 128.5 119.4 115.0 114.6 114.1
83.8
77.2 CDCl3
71.8 68.9
25.8 25.0
O
NH
O
N
H
Me
Bpin
2o
210 200 190 180 170 160 150 140 130 120 110 100 90 80 70 60 50 40 30 20 10 0 -10
f1 (ppm)

^{11}B NMR of **2o**

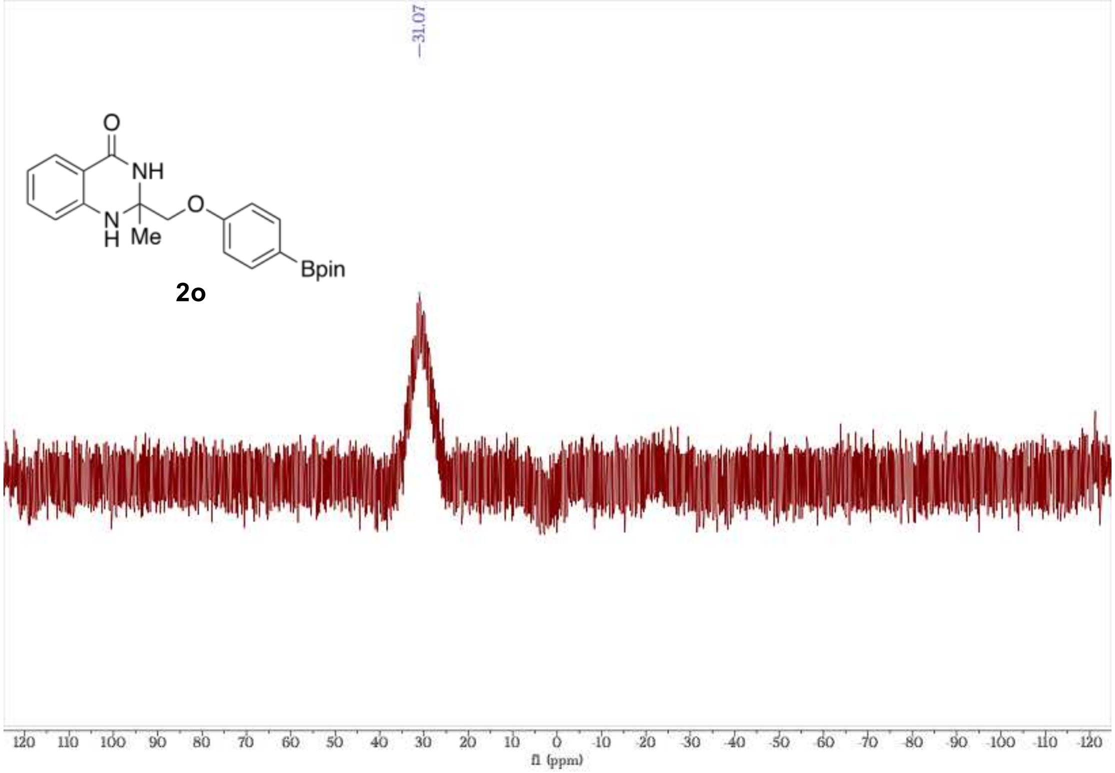

^{1}H NMR of **2p**

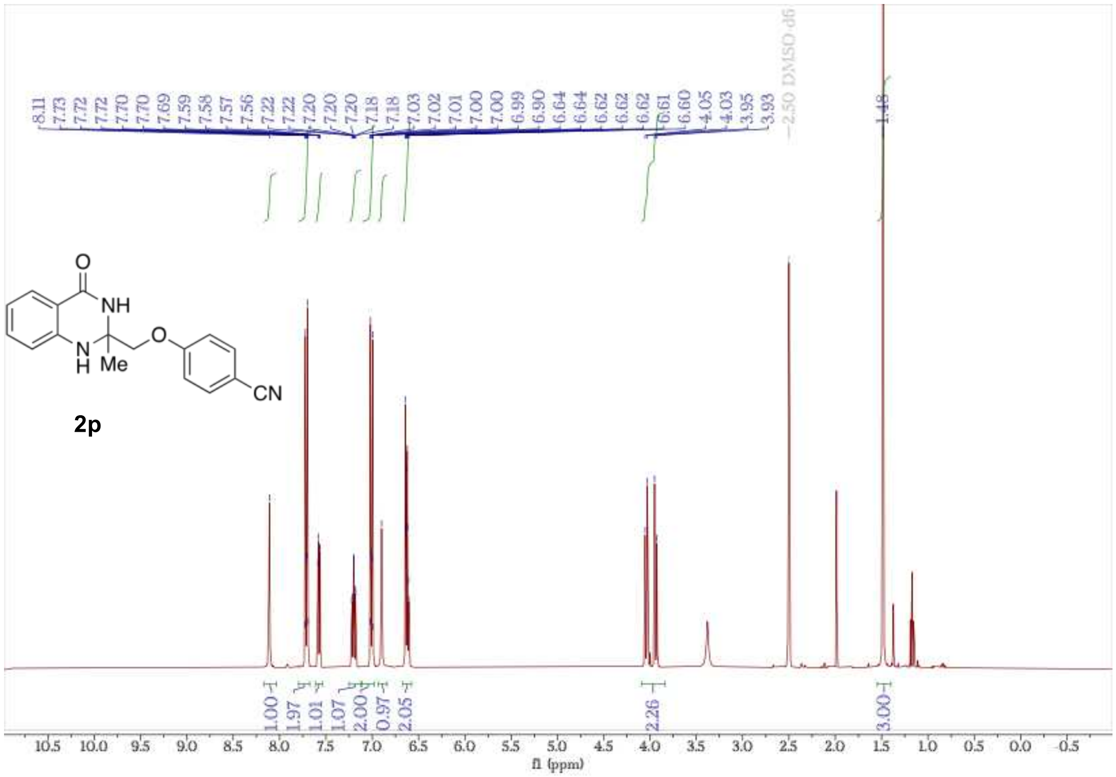

^{13}C NMR of **2p**

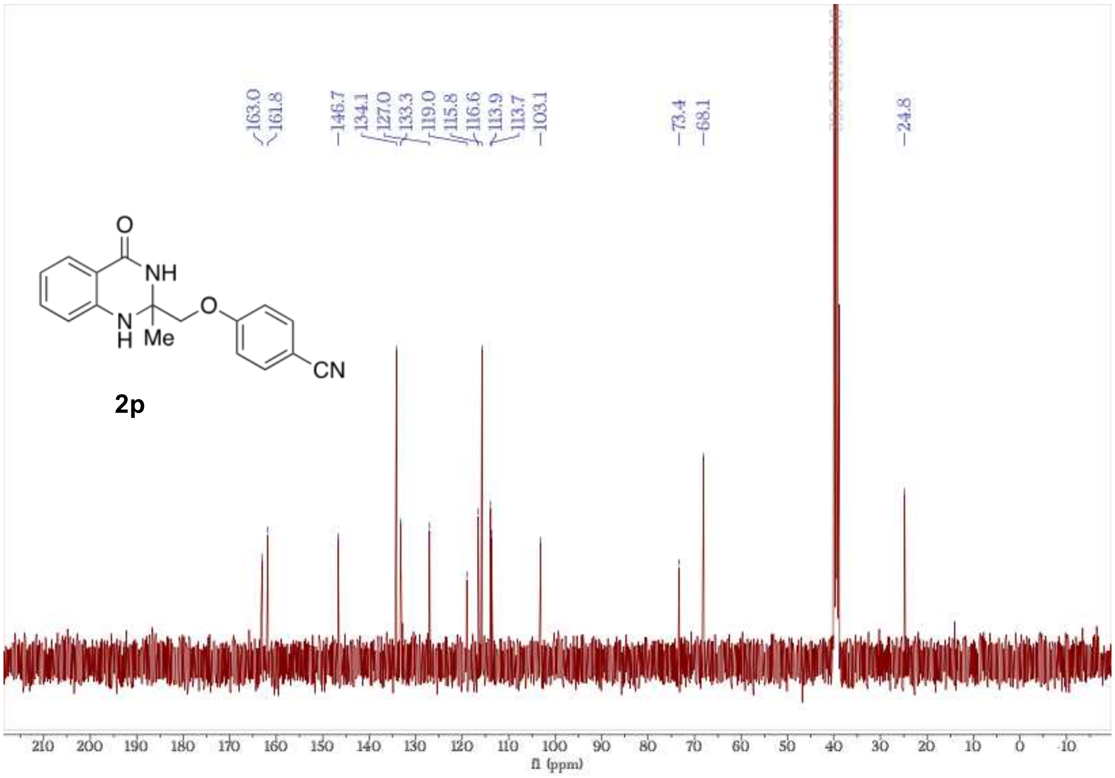

^{1}H NMR of **2q**

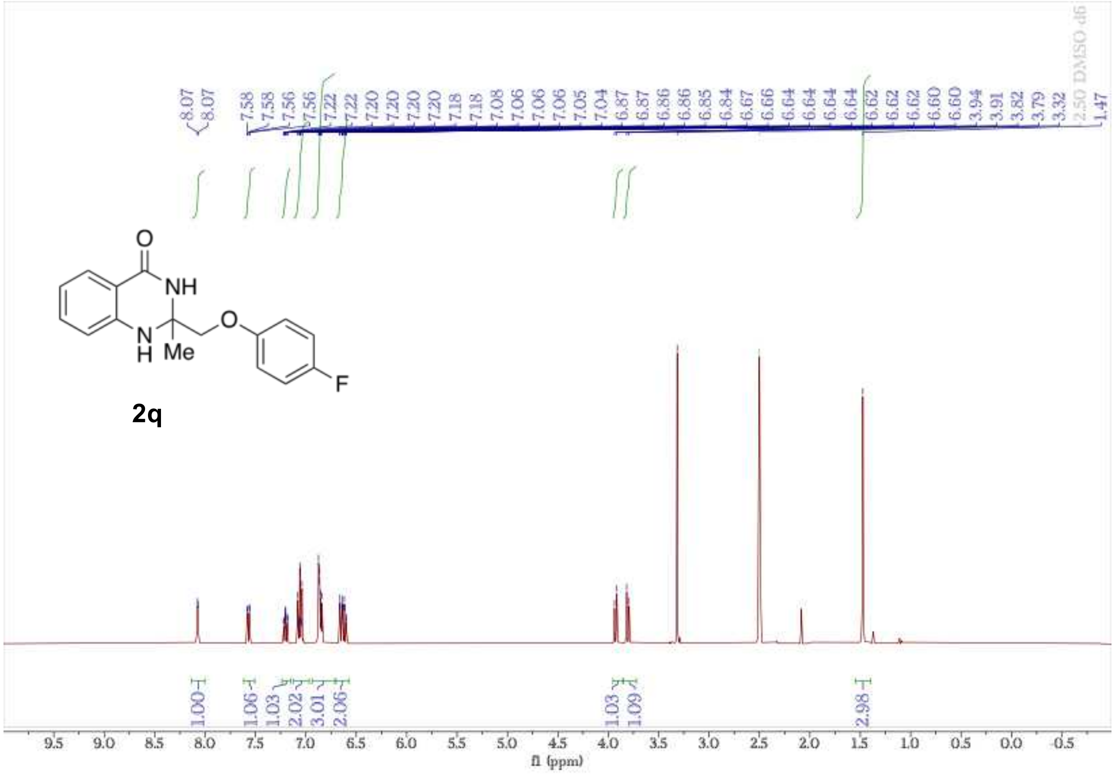

^{13}C NMR of **2q**

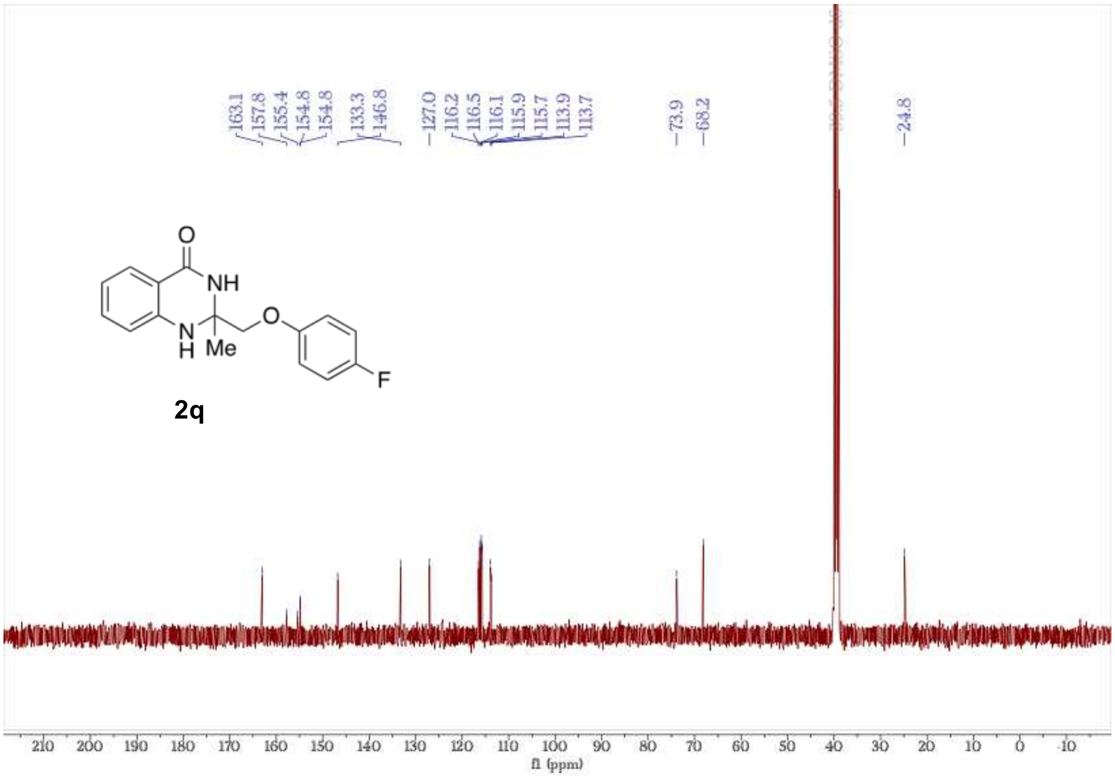

^{19}F NMR of **2q**

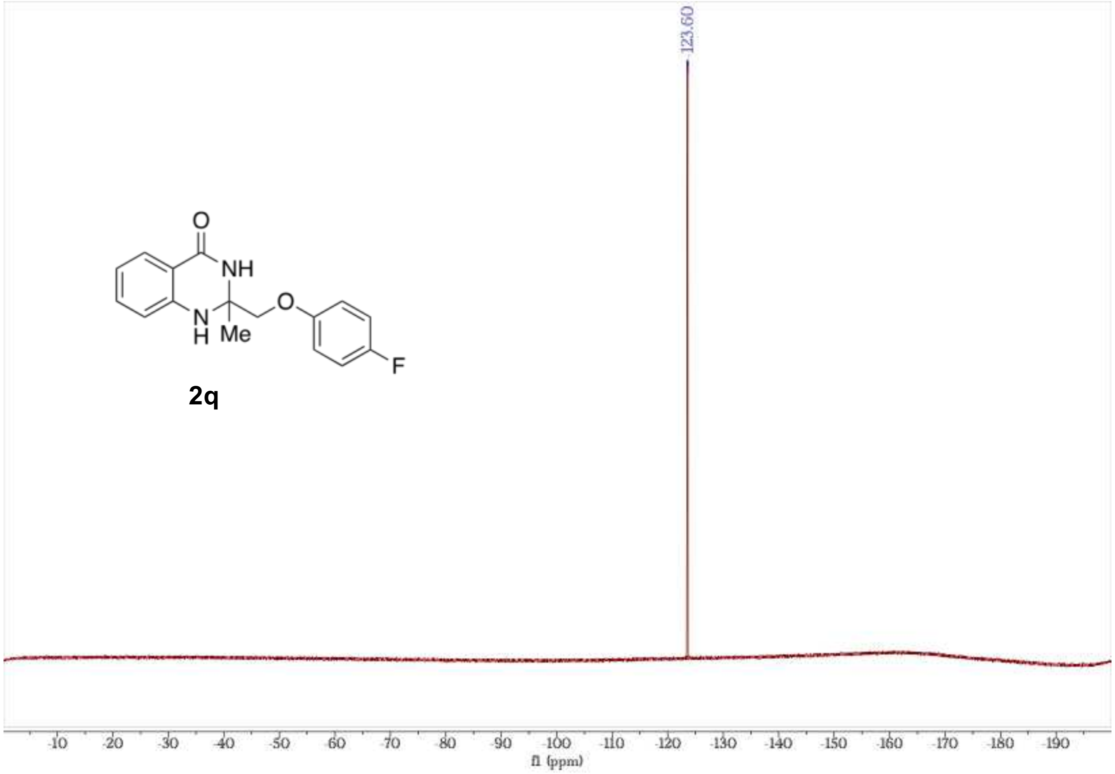

^{1}H NMR of **2r**

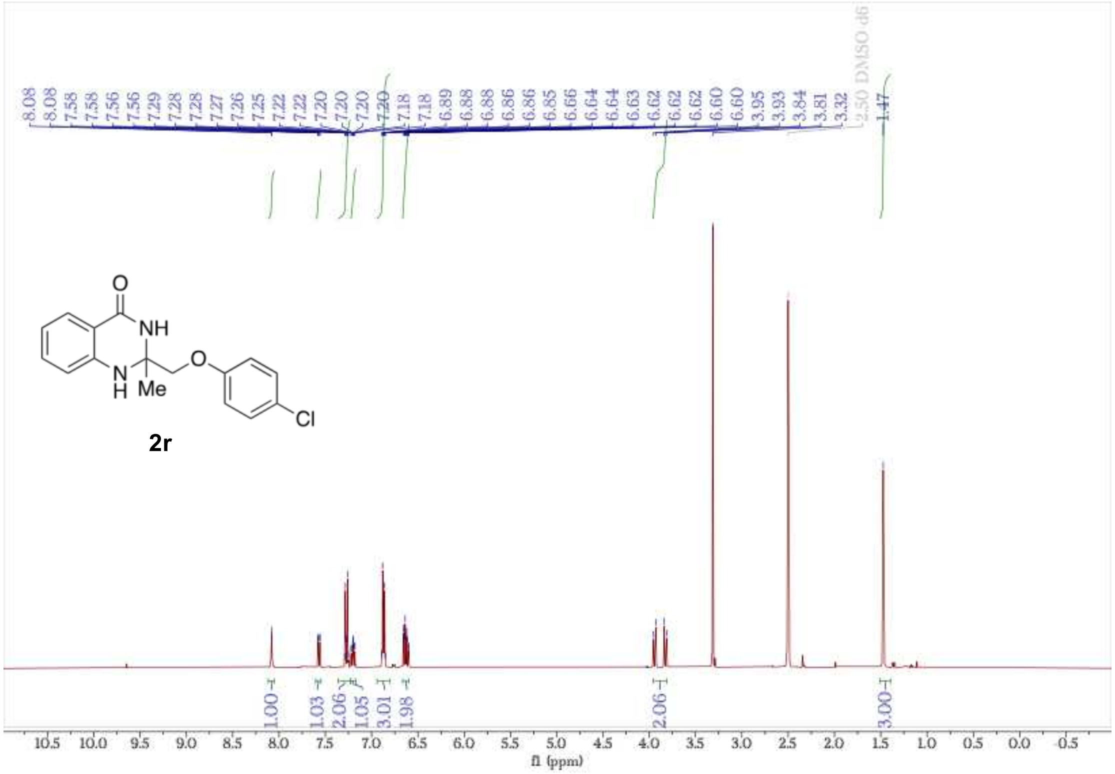

^{13}C NMR of **2r**

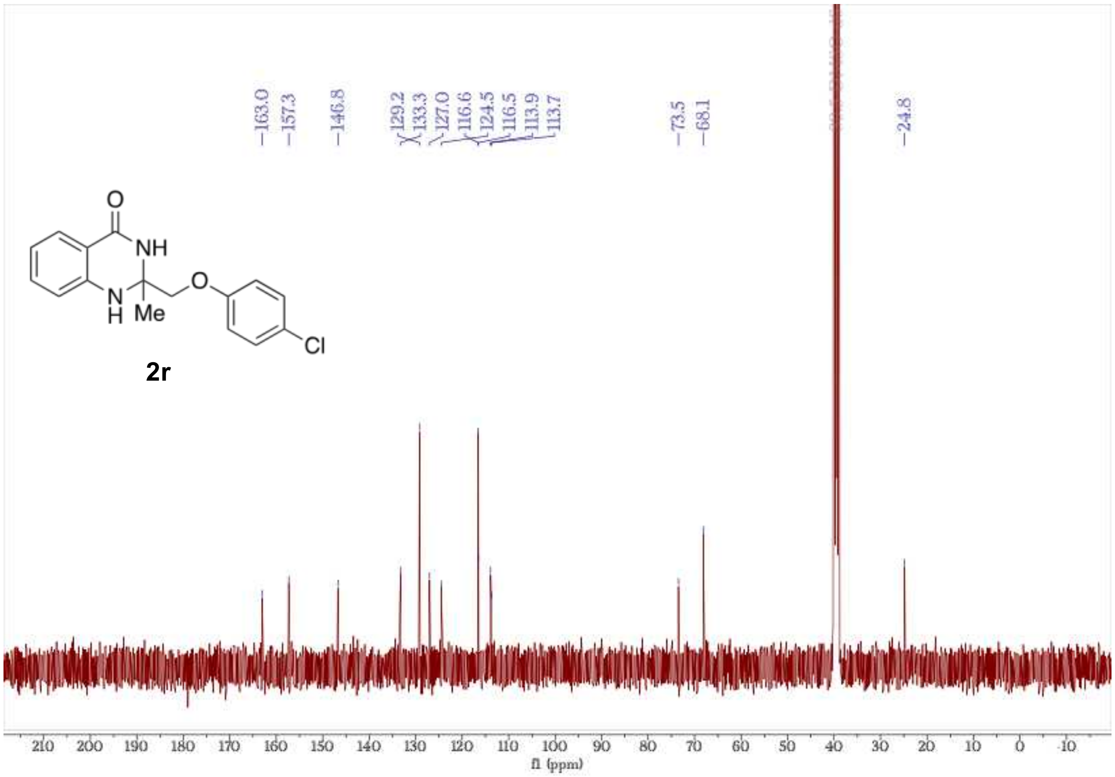

^{1}H NMR of **2v**

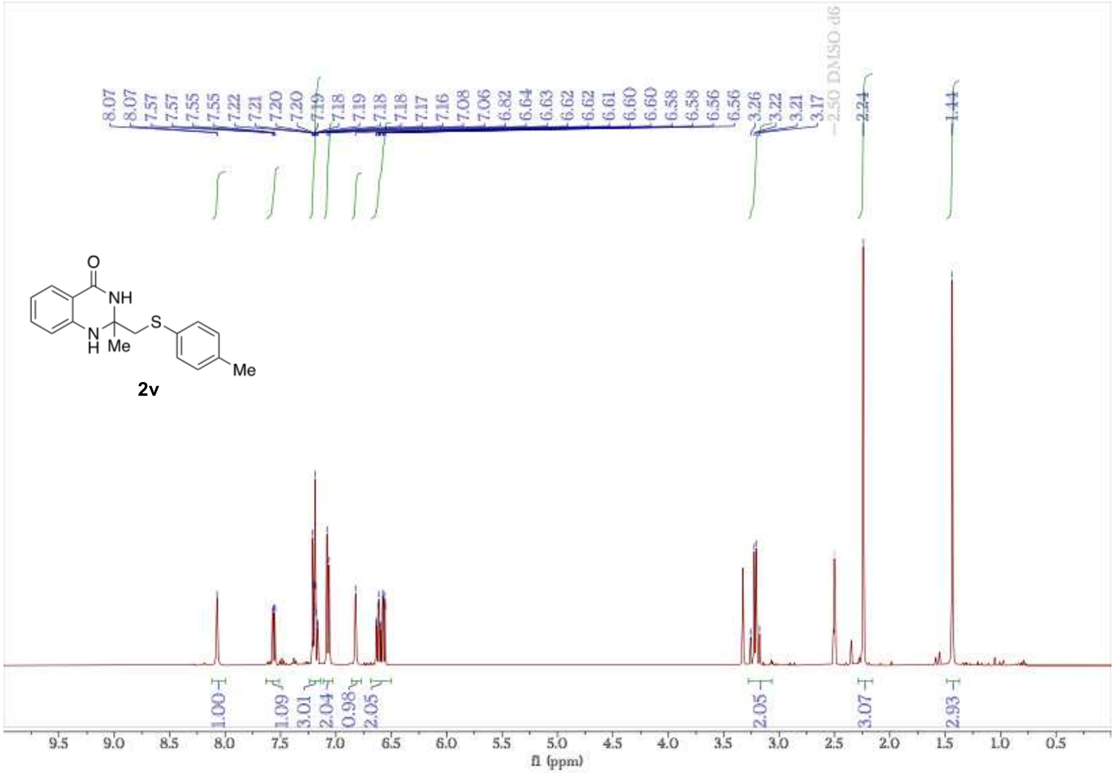

^{13}C NMR of **2v**

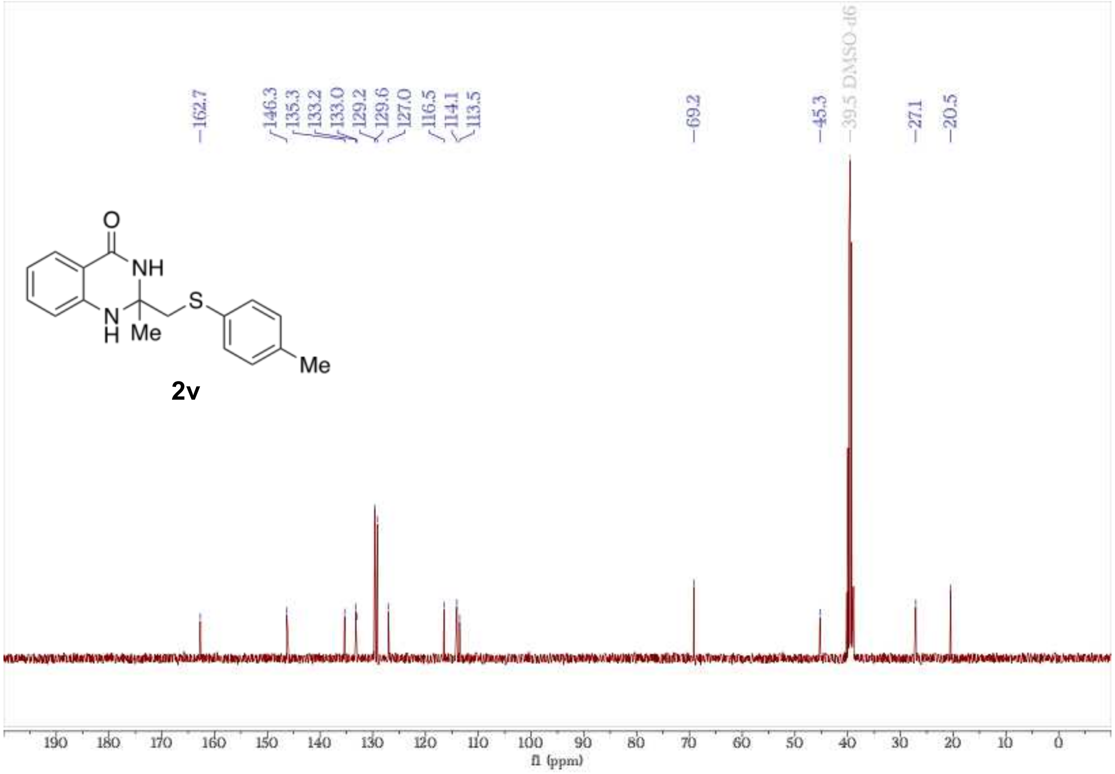

^{1}H NMR of **2w**

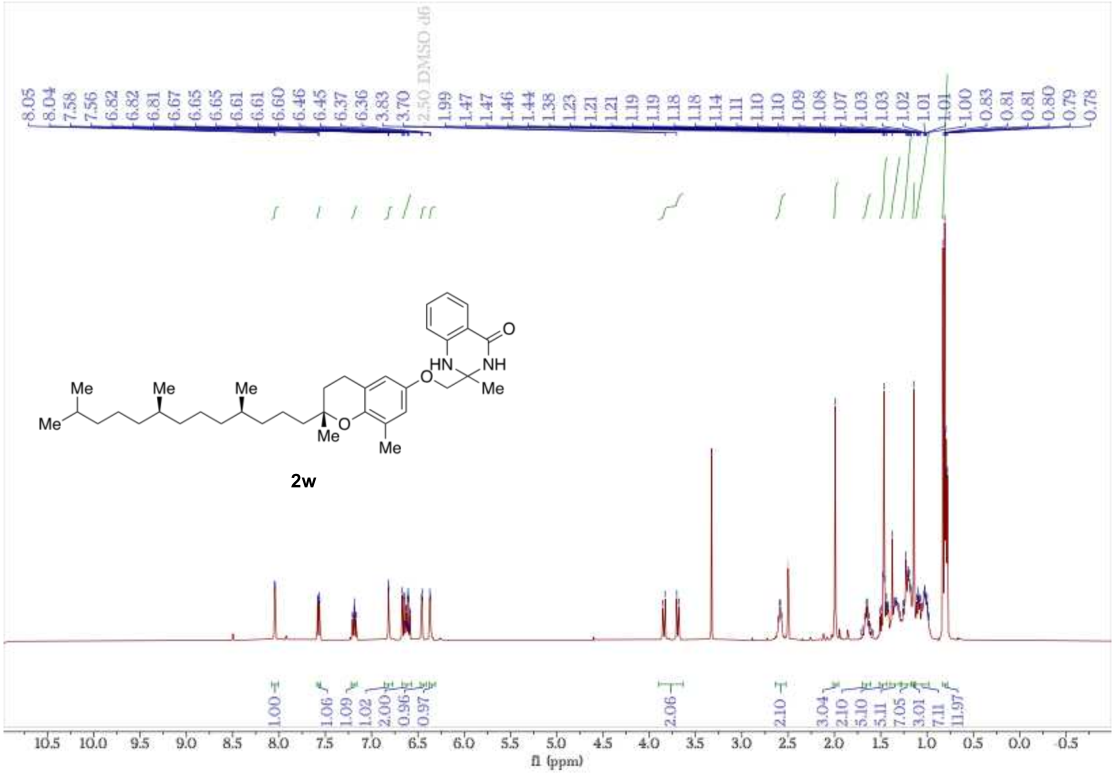
8.05 8.04 7.58 7.56 6.82 6.82 6.81 6.67 6.65 6.65 6.61 6.61 6.60 6.46 6.45 6.37 6.36 3.83 3.70 2.50 DMSO d6 1.99 1.47 1.47 1.46 1.44 1.38 1.23 1.21 1.21 1.19 1.19 1.18 1.18 1.14 1.11 1.10 1.10 1.09 1.08 1.07 1.03 1.03 1.02 1.01 1.01 1.00 0.83 0.81 0.81 0.80 0.79 0.78
1.00 1.06 1.09 1.02 2.00 0.96 0.97 2.06 2.10 3.04 2.10 5.10 5.11 7.05 3.01 7.11 11.97
2w
f1 (ppm)

^{13}C NMR of **2w**

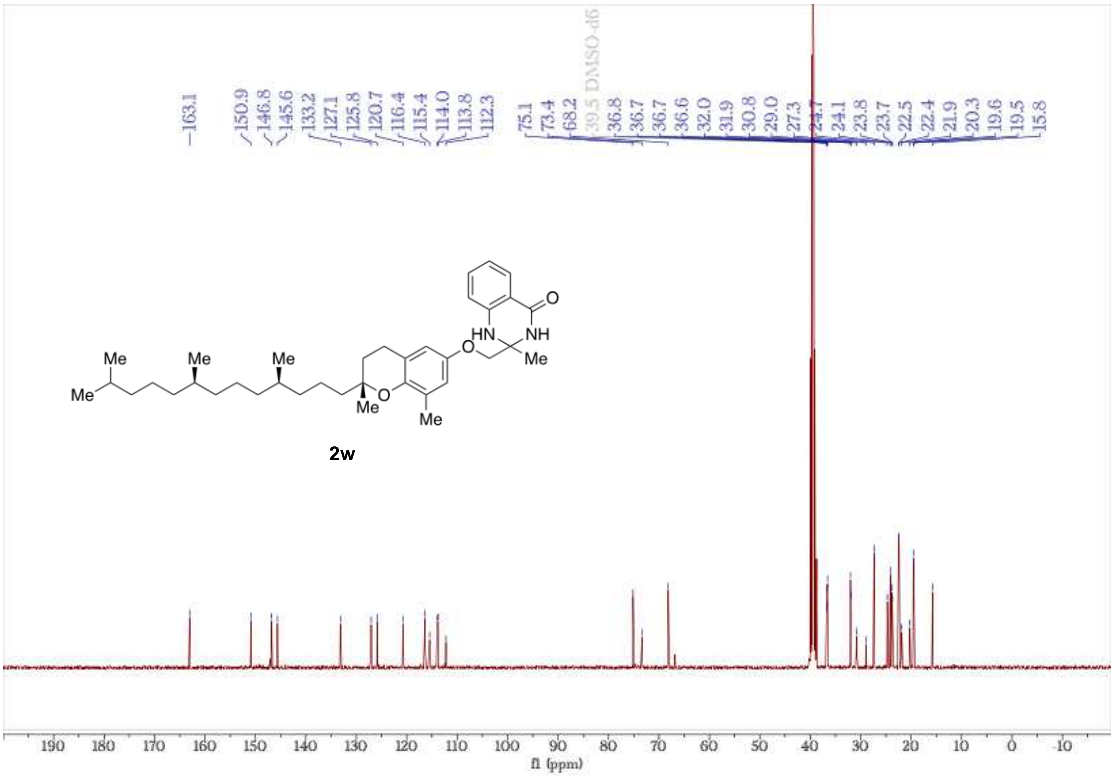
163.1 150.9 146.8 145.6 133.2 127.1 125.8 120.7 116.4 115.4 114.0 113.8 112.3 75.1 73.4 68.2 39.5 DMSO d6 36.8 36.7 36.7 36.6 32.0 31.9 30.8 29.0 27.3 24.7 24.1 23.8 23.7 22.5 22.4 21.9 20.3 19.6 19.5 15.8
2w
f1 (ppm)

^{1}H NMR of **2za**

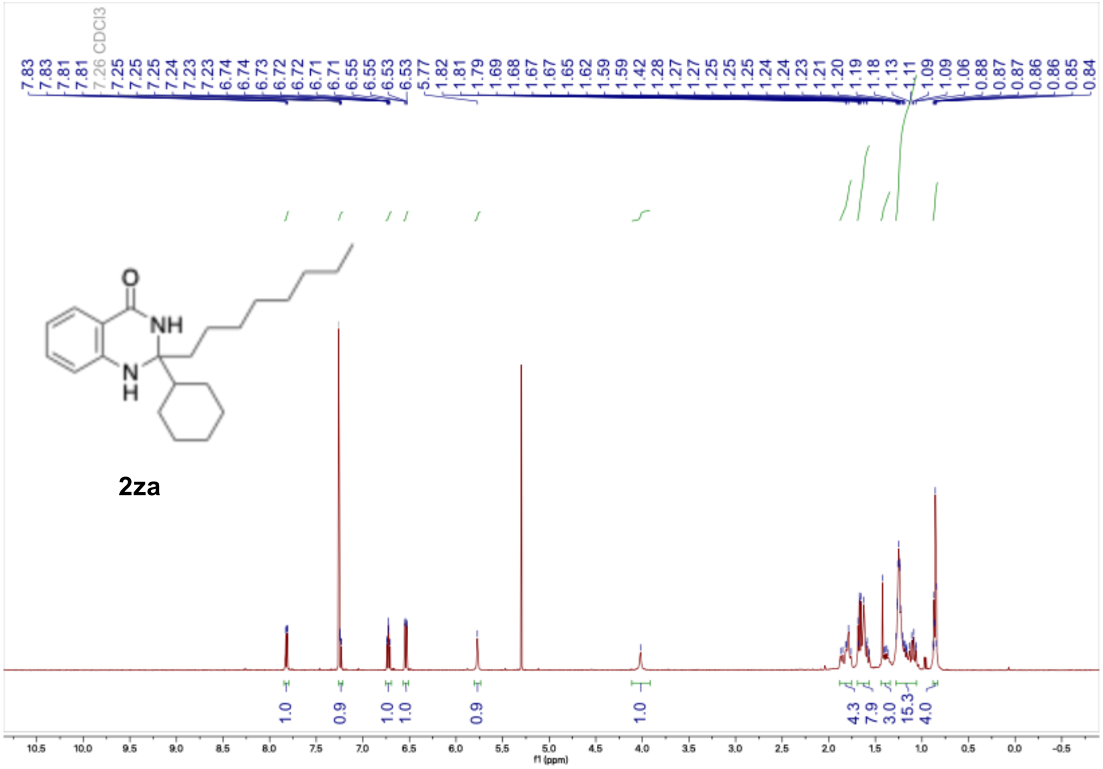

^{13}C NMR of **2za**

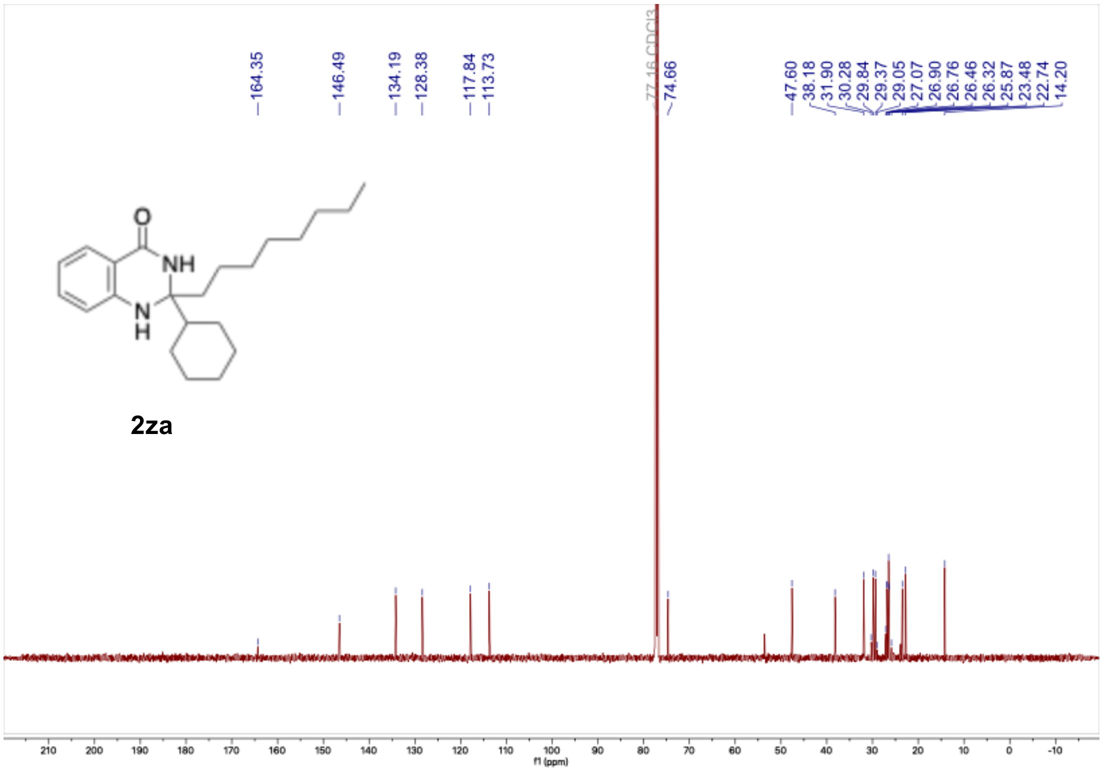

^{1}H NMR of **2zb**

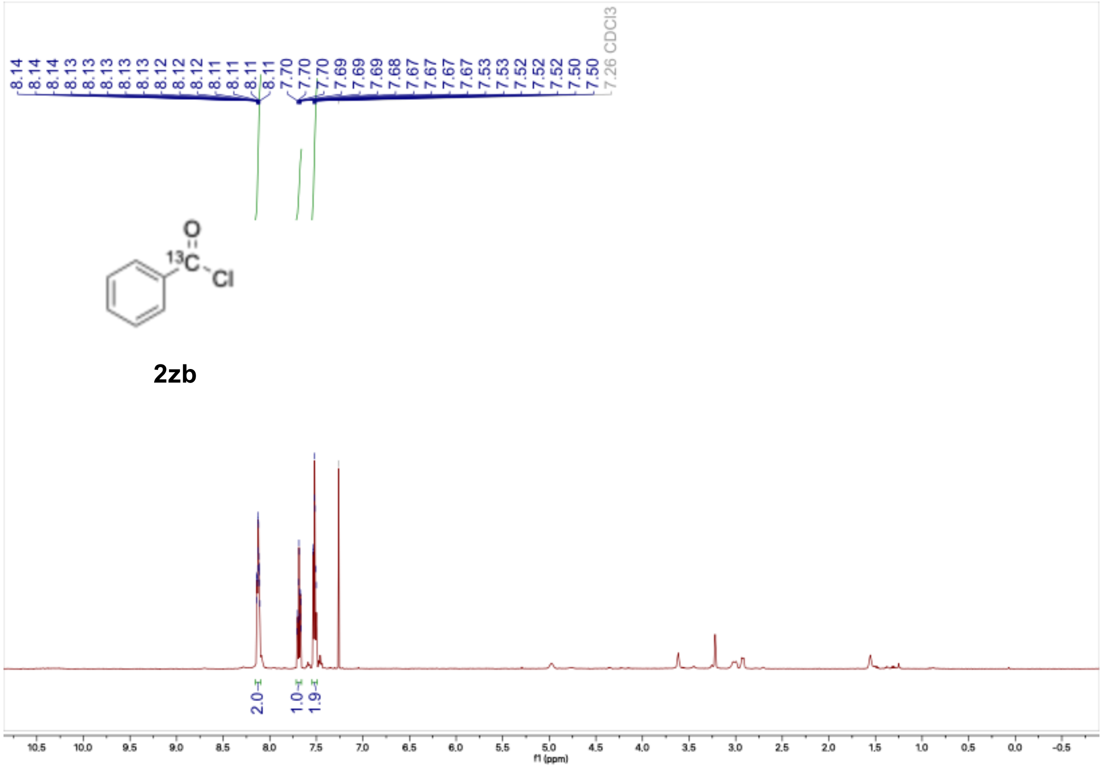

^{13}C NMR of **2zb**

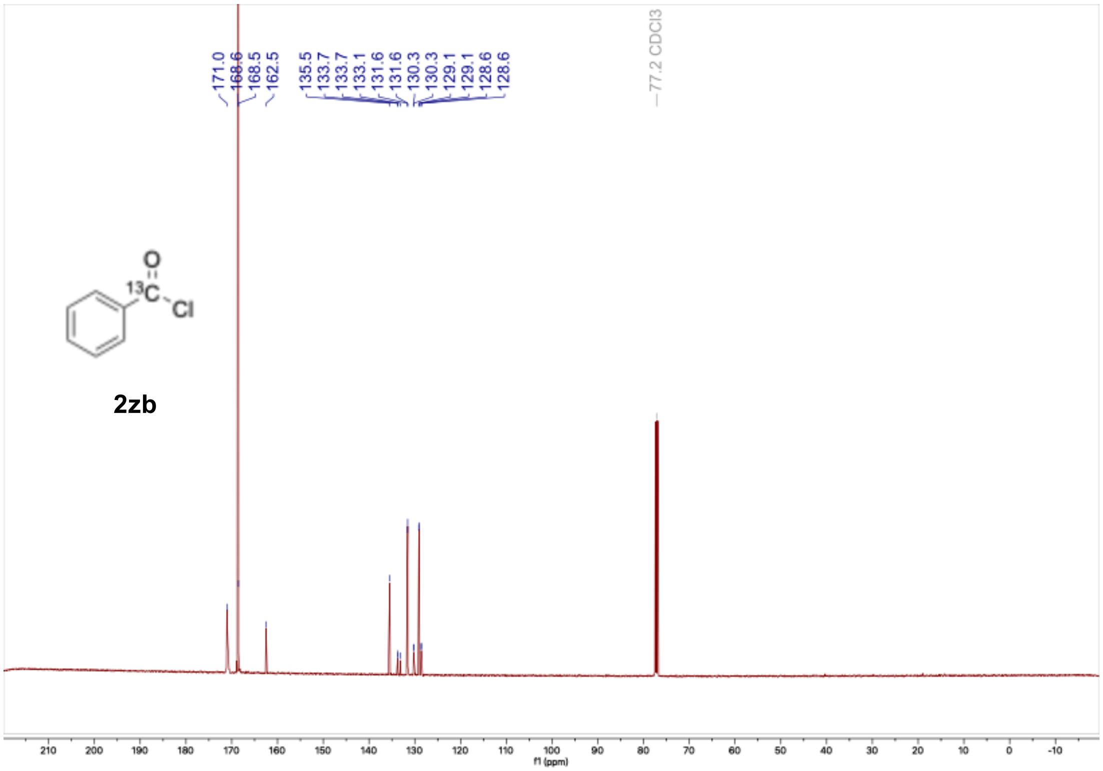

^{1}H NMR of **2zc**

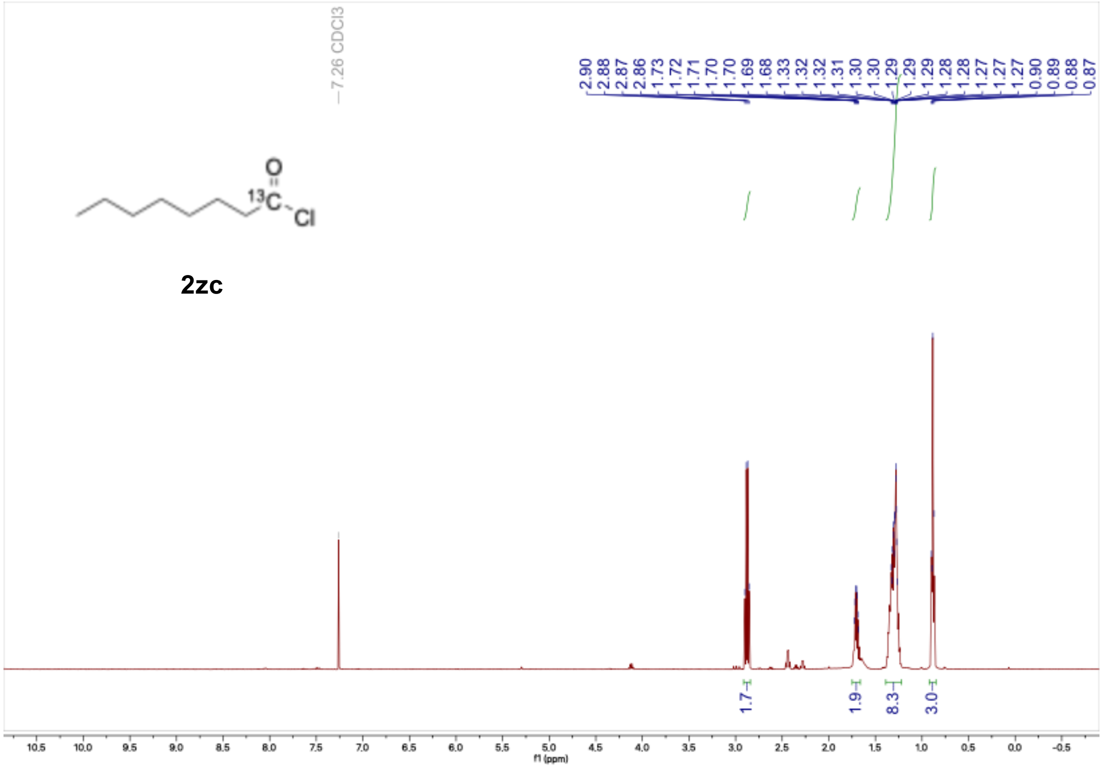

^{13}C NMR of **2zc**

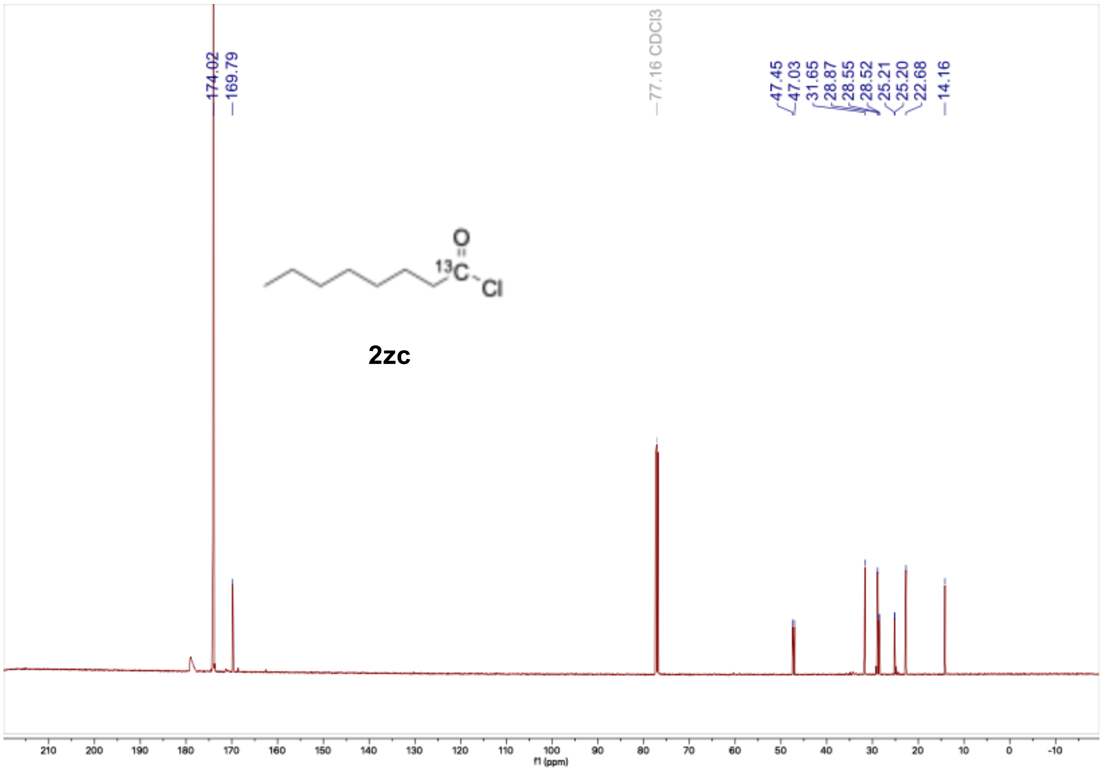

^{1}H NMR of **2zd**

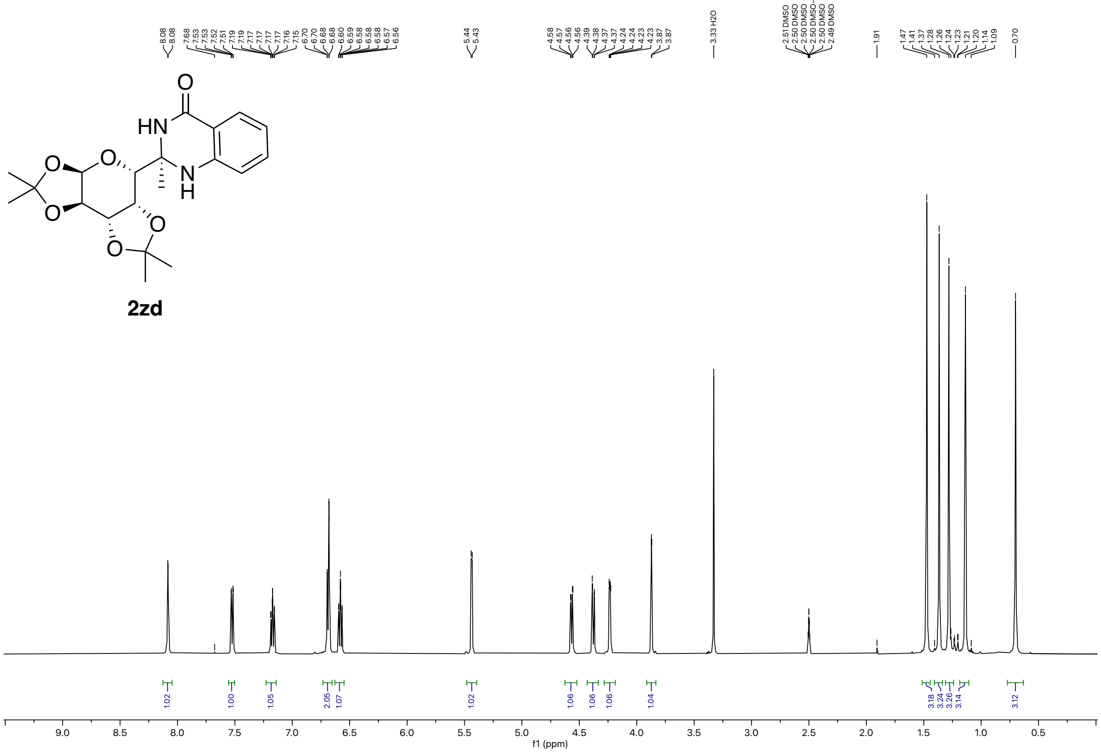

^{13}C NMR of **2zd**

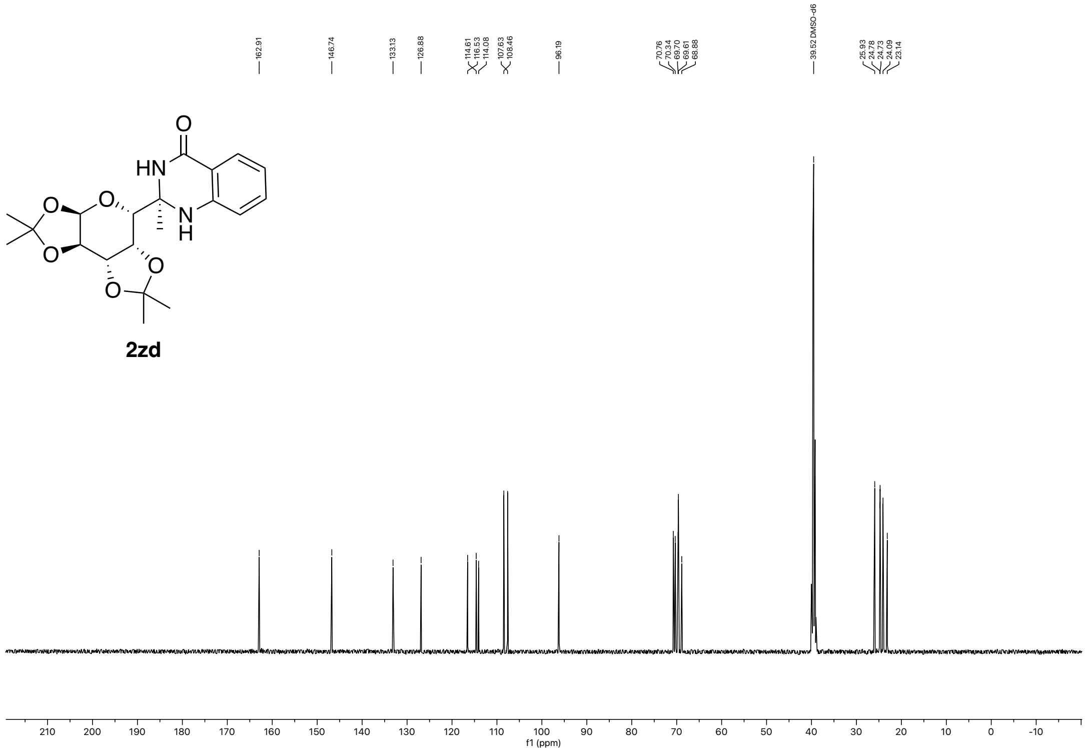

^{1}H NMR of **2ze**

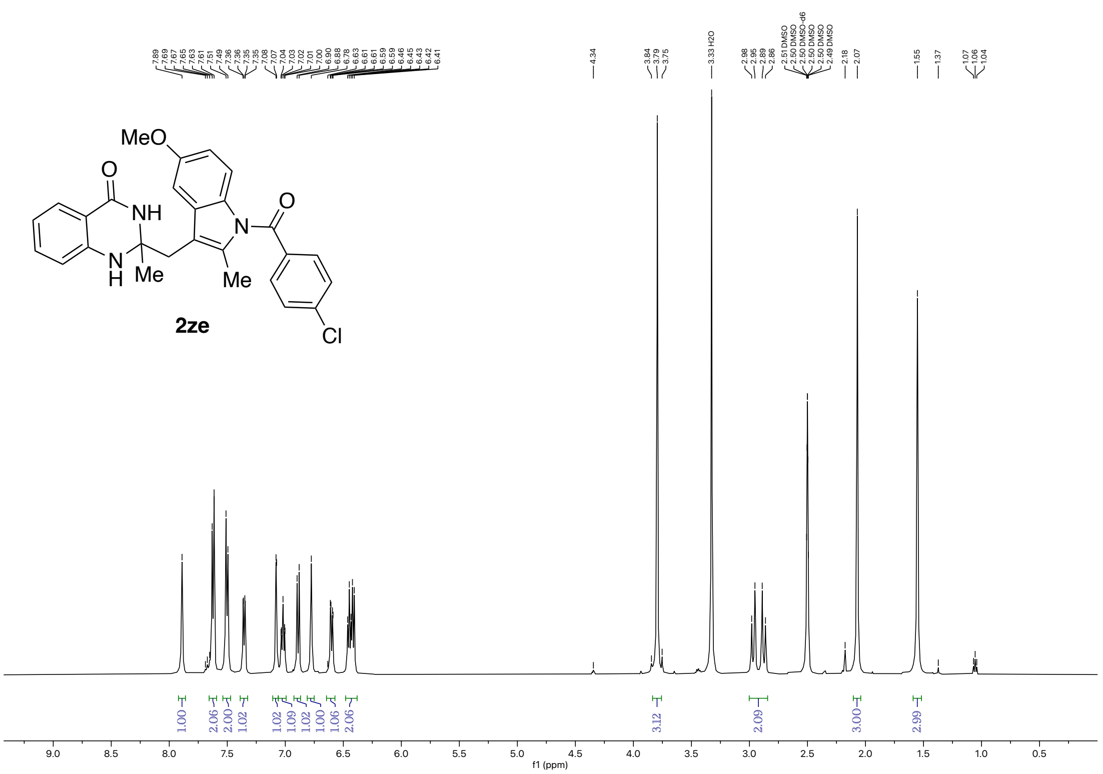

^{13}C NMR of **2ze**

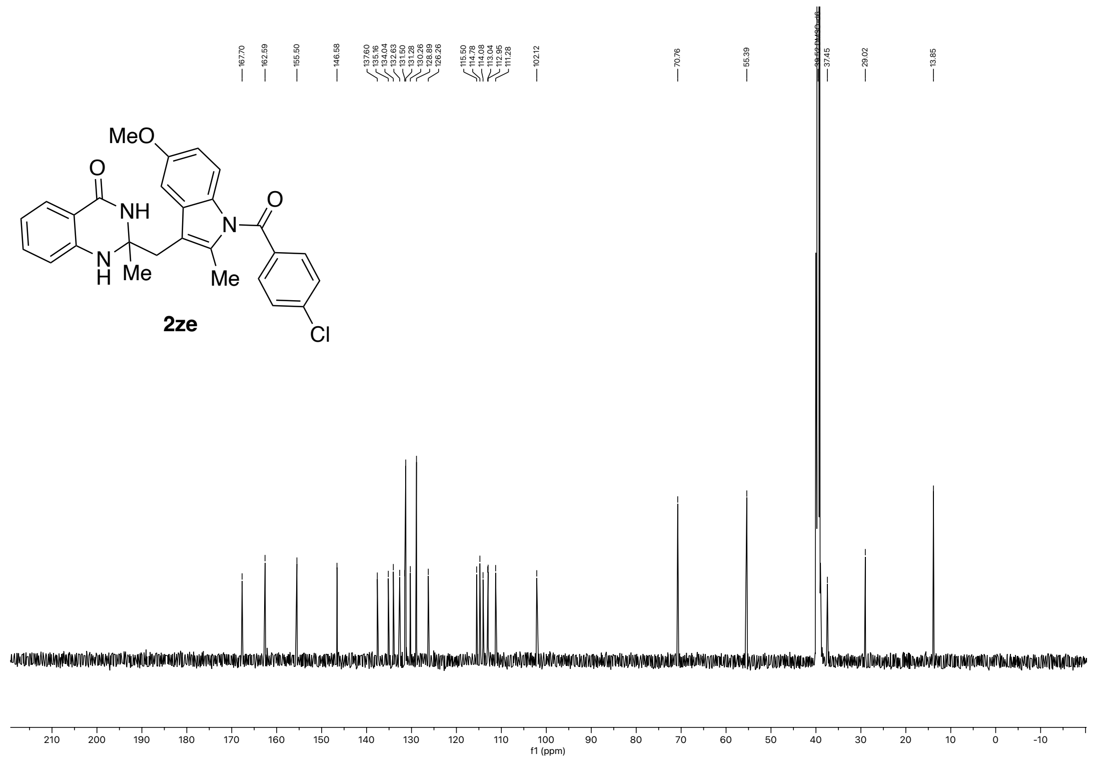

^{1}H NMR of **2zg**

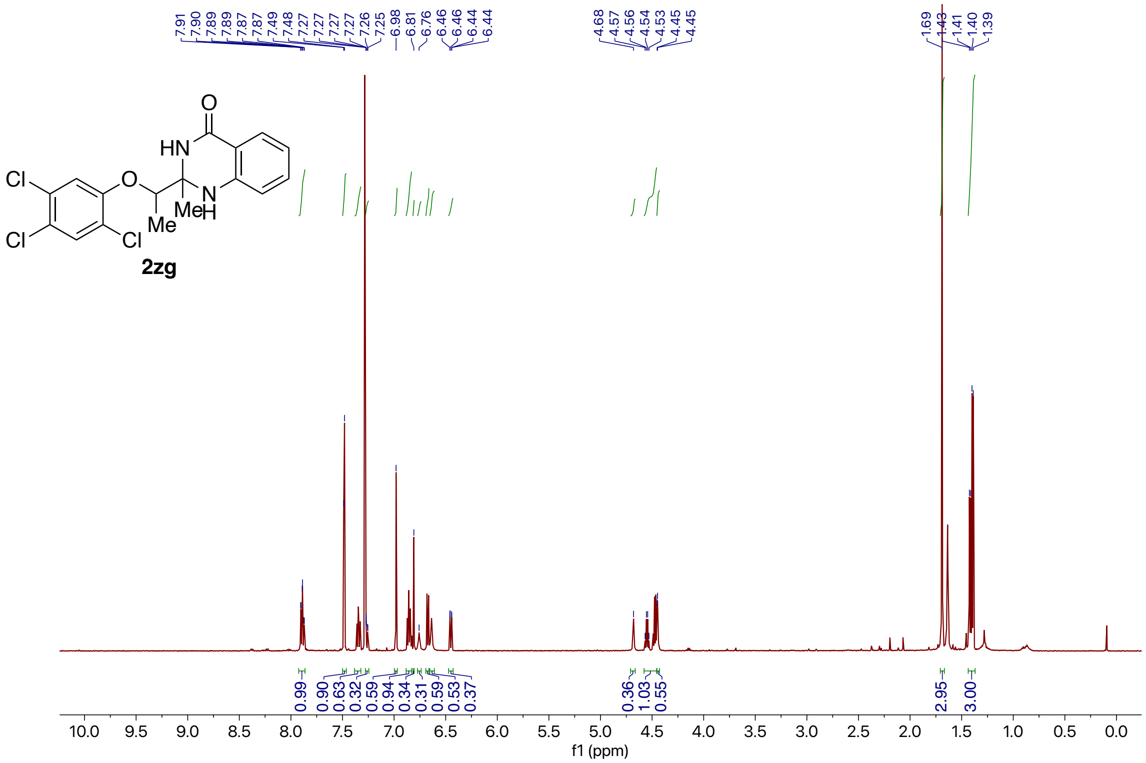

^{13}C NMR of **2zg**

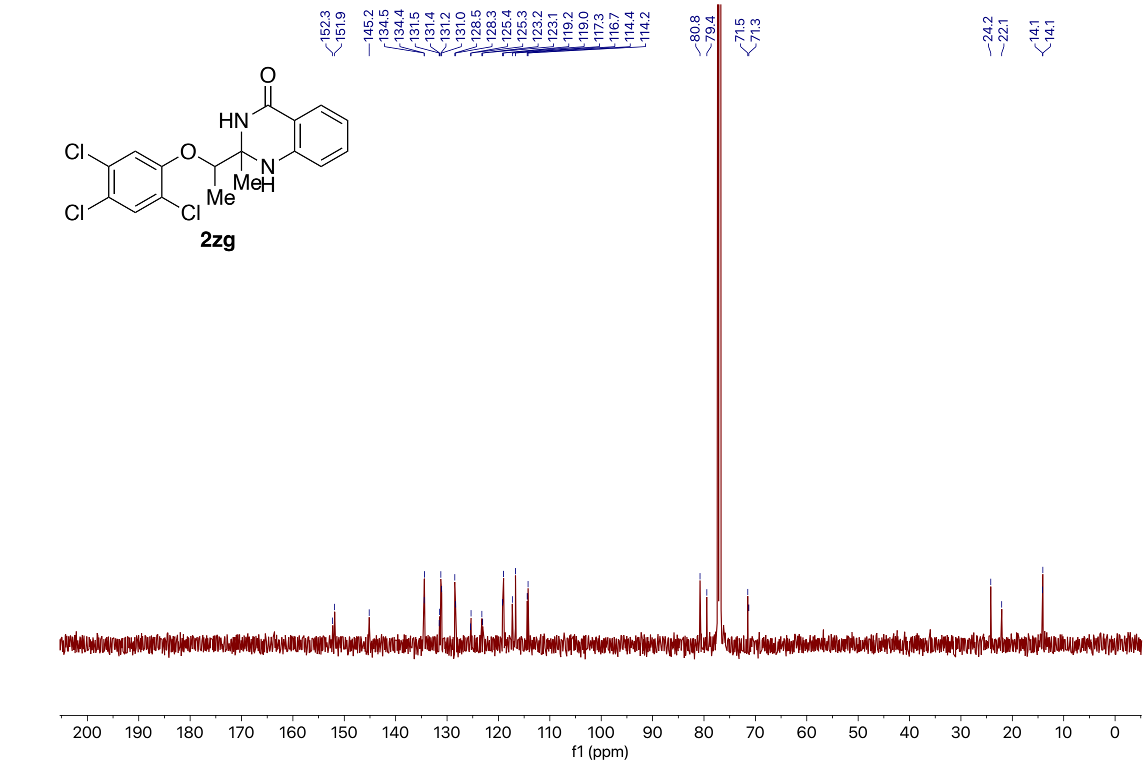

^{1}H NMR of **[13C]1a**

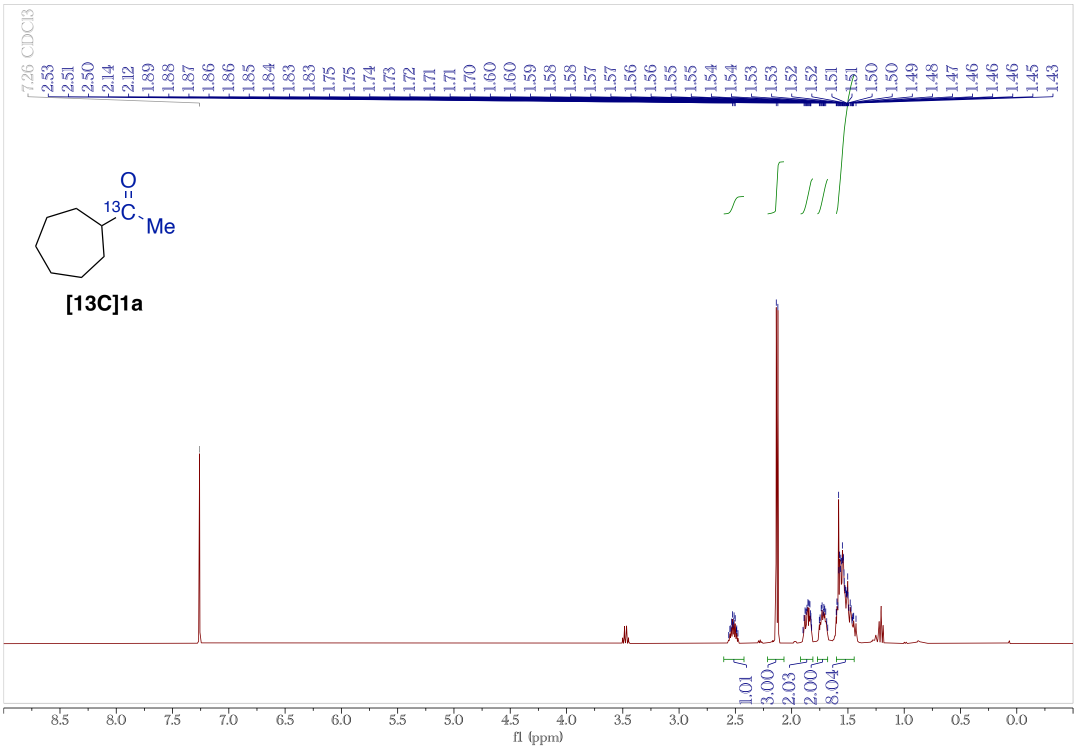

^{13}C NMR of **[13C]1a**

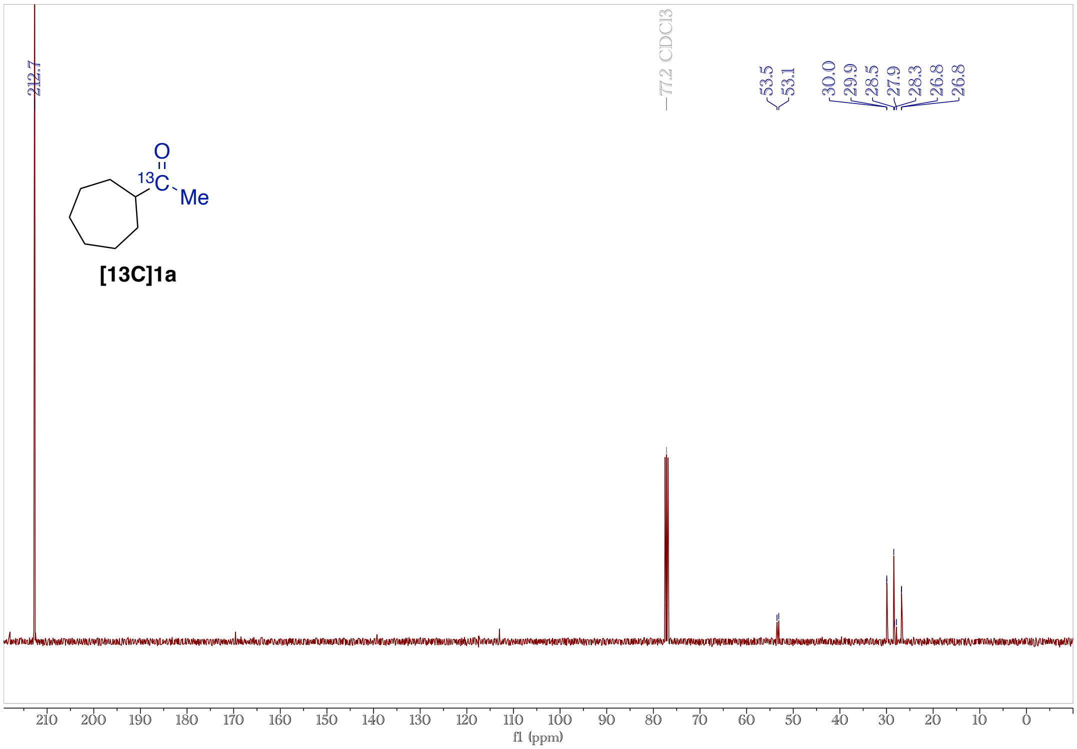

^{1}H NMR of **[13C]1b**

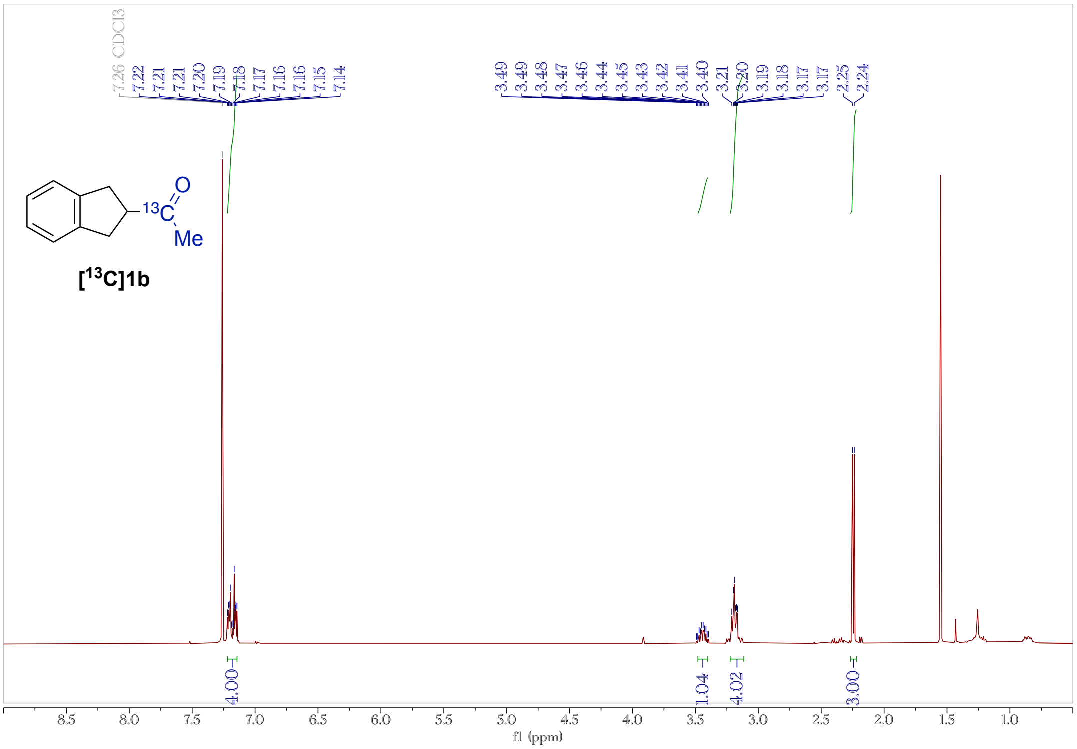

^{13}C NMR of **[13C]1b**

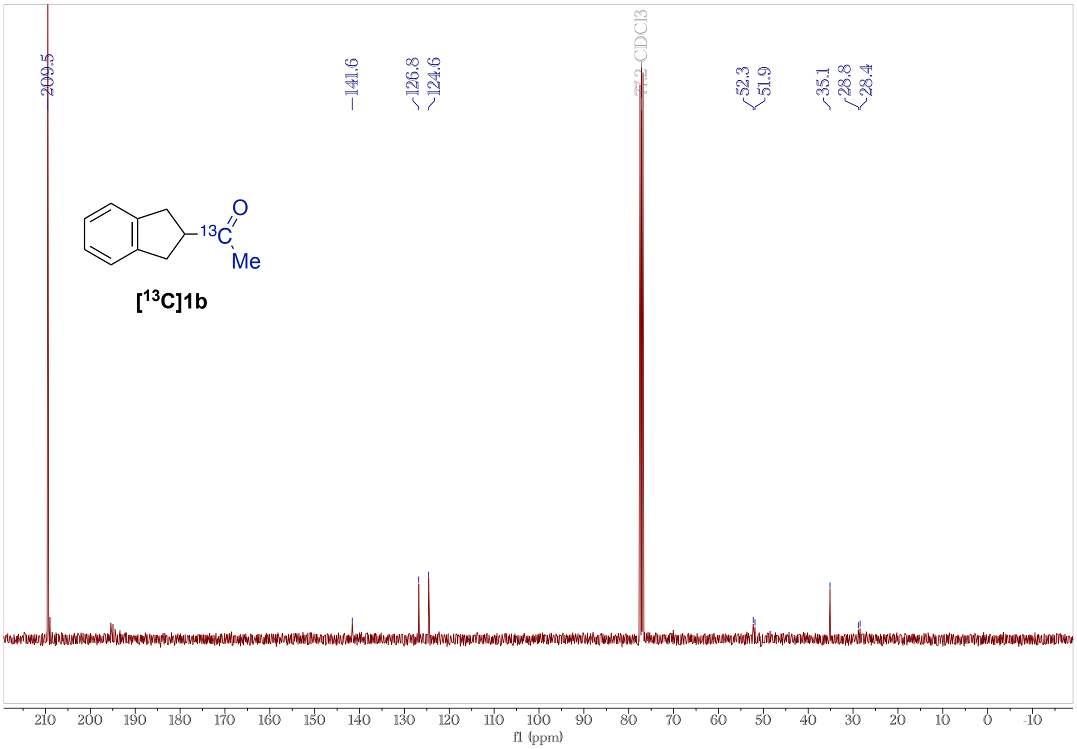

^{1}H NMR of **[13C]1c**

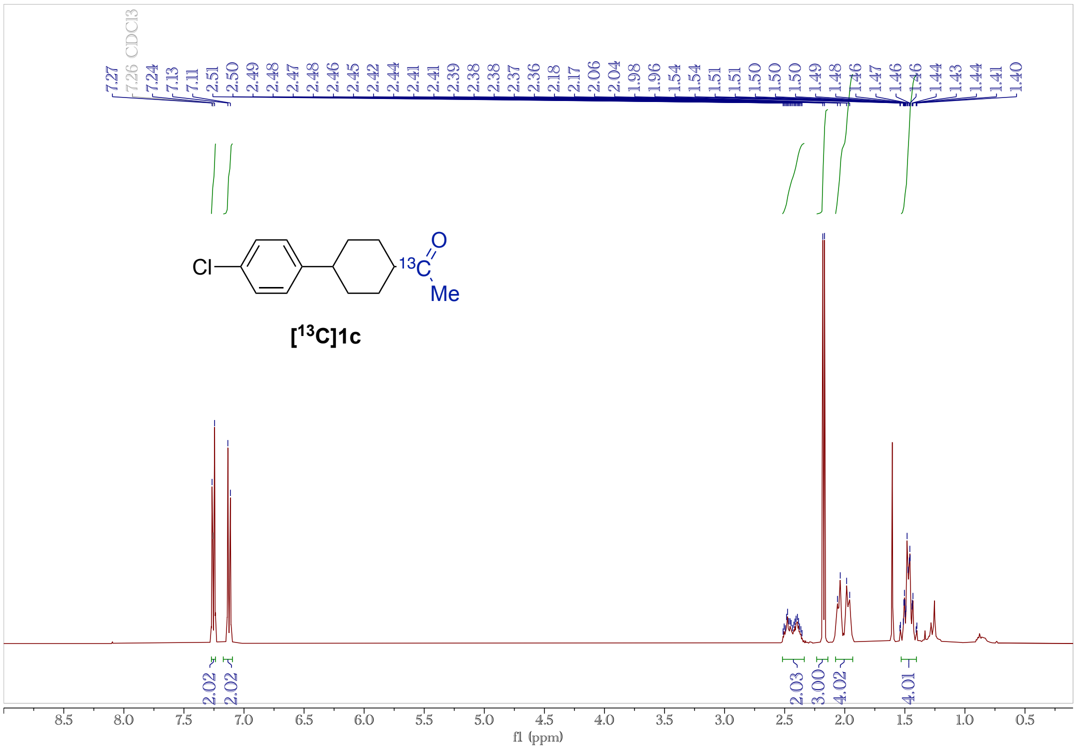

^{13}C NMR of **[13C]1c**

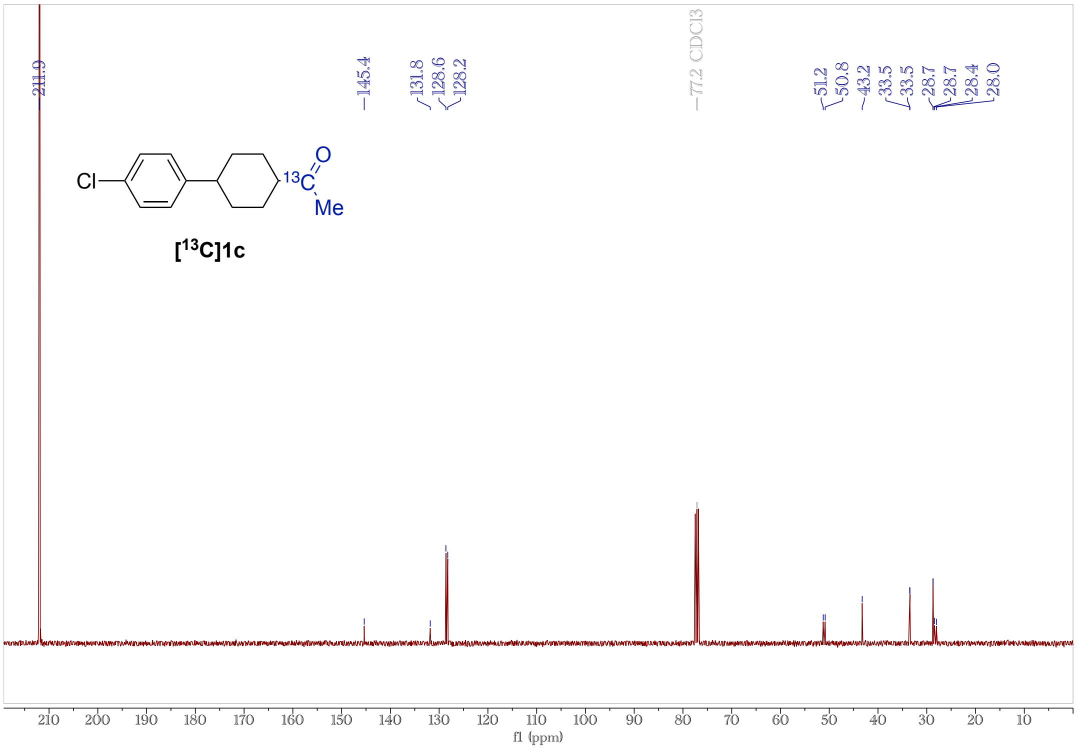

^1H NMR of **[13C]1d**

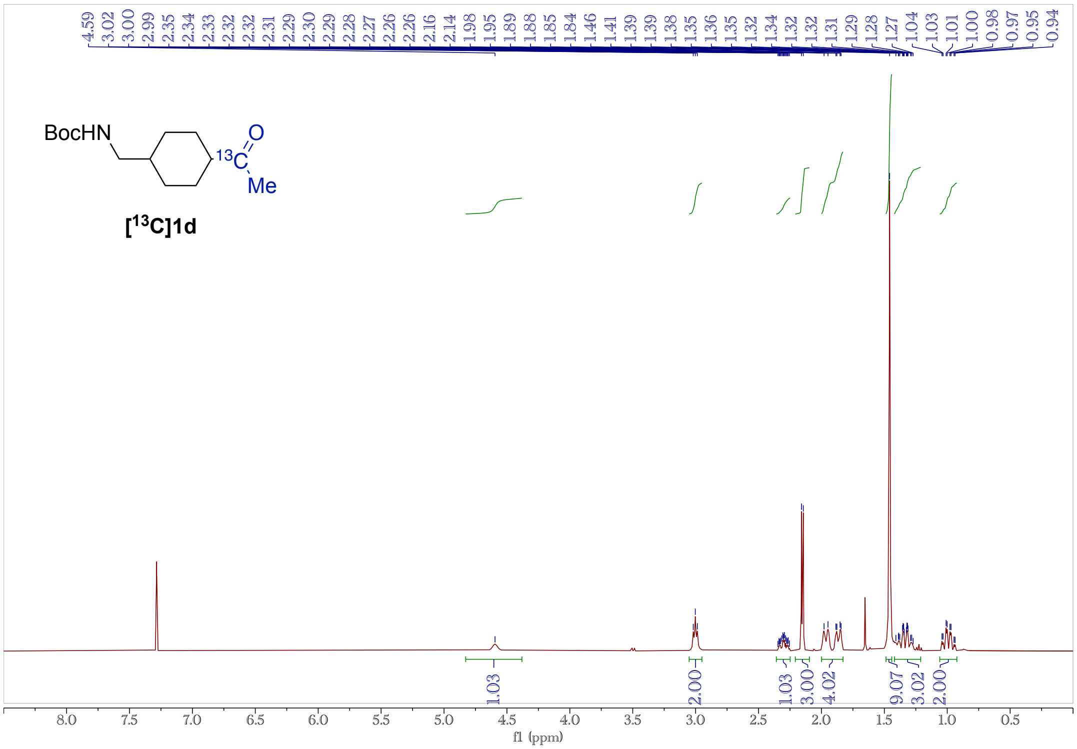

^{13}C NMR of **[13C]1d**

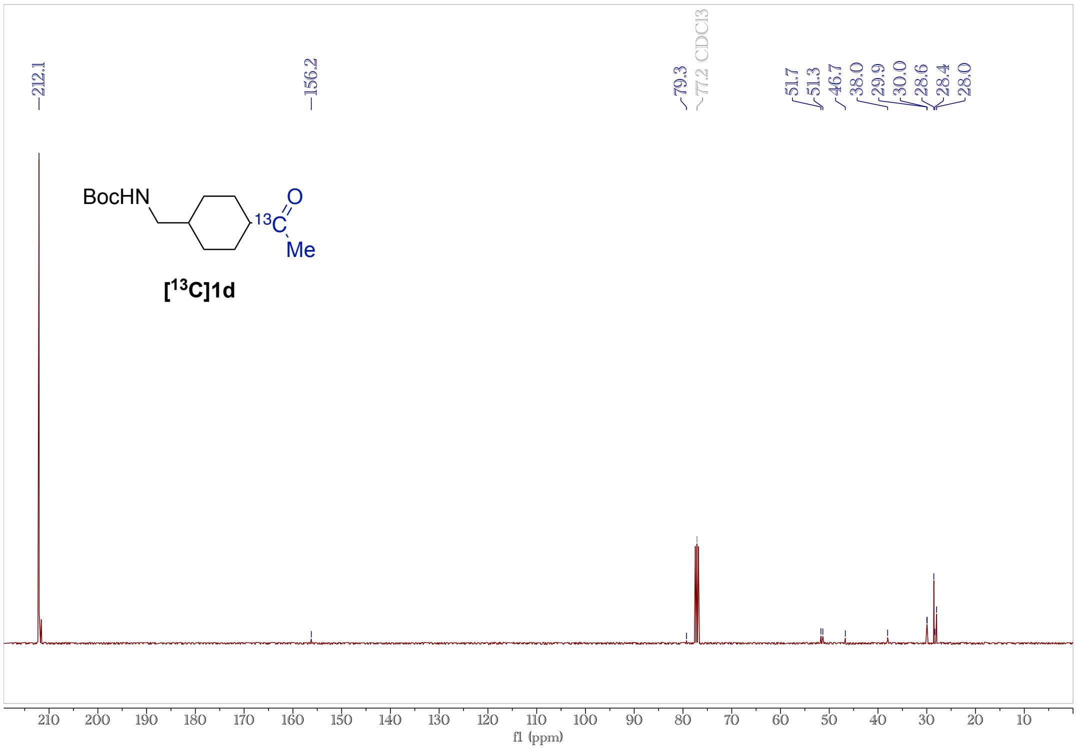

^{1}H NMR of **[13C]1e**

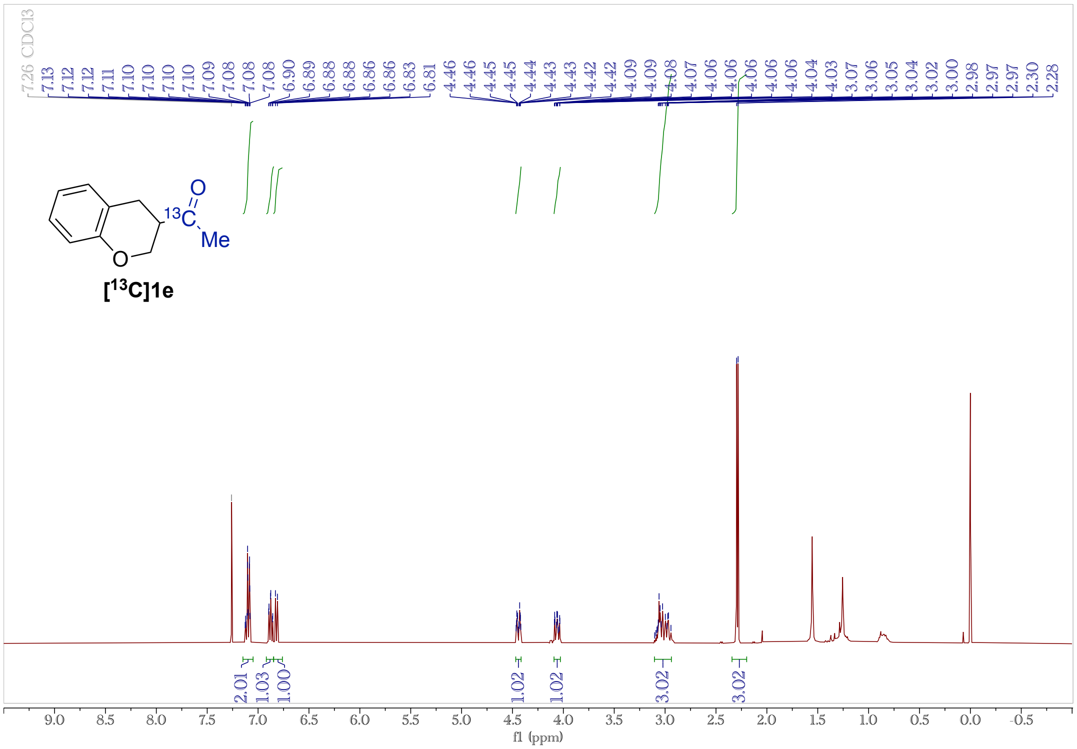

^{13}C NMR of **[13C]1e**

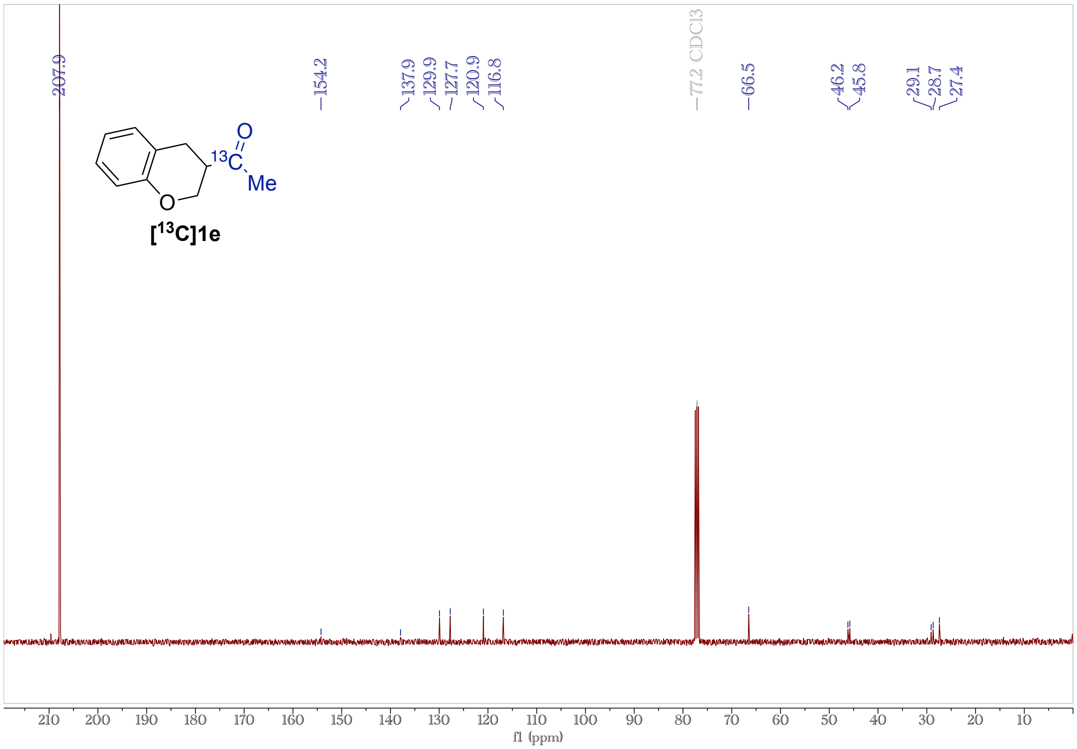

^{1}H NMR of **[13C]1f**

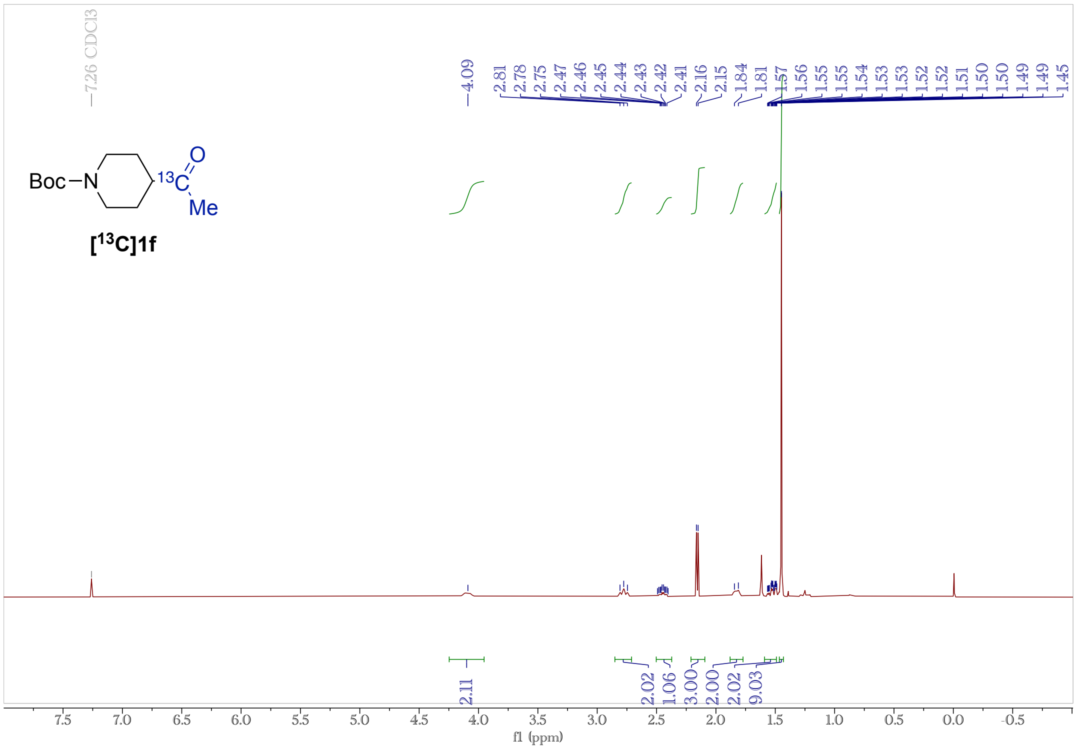

^{13}C NMR of **[13C]1f**

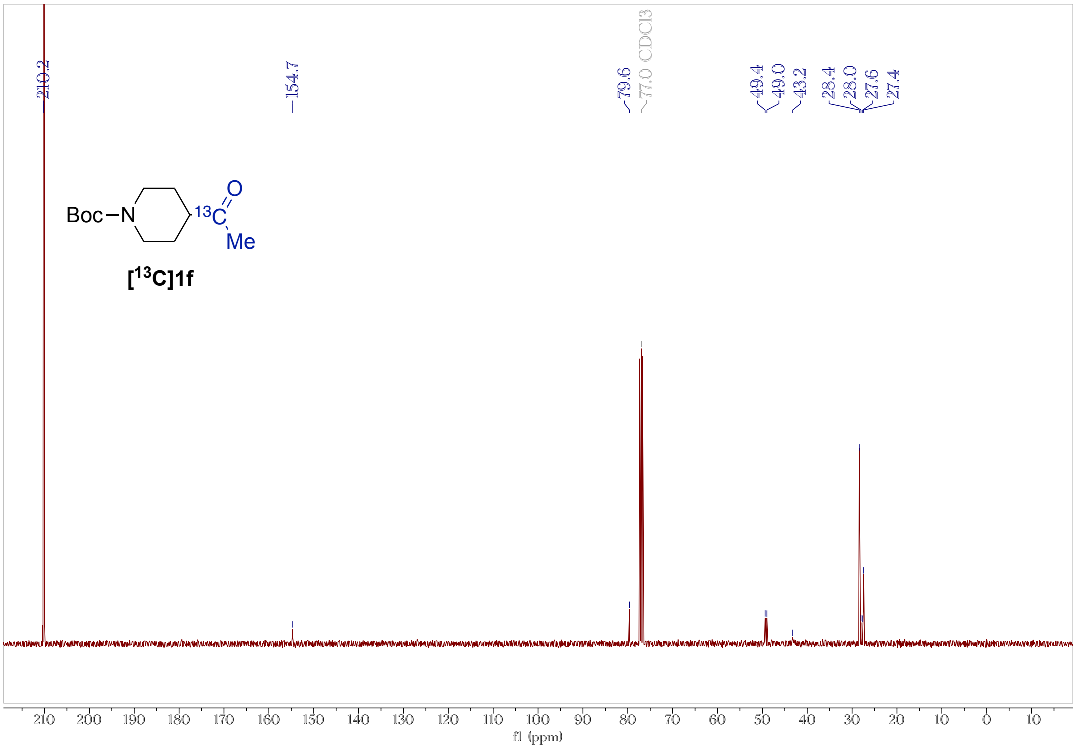

^{1}H NMR of **[13C]1g**

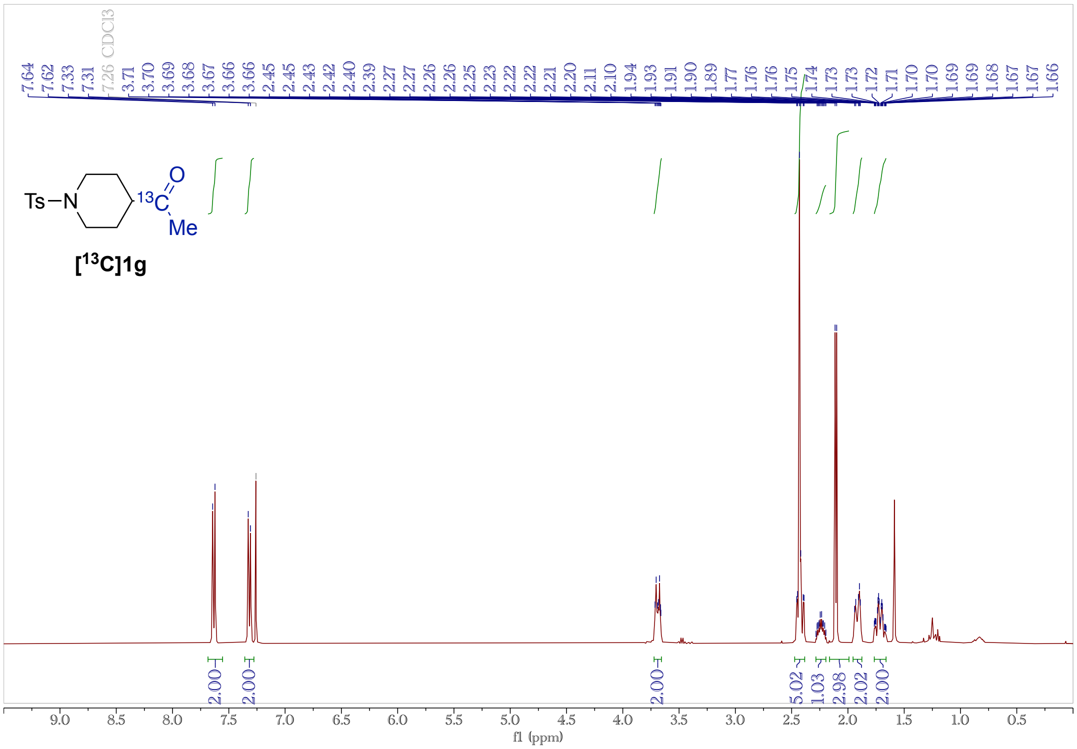

^{13}C NMR of **[13C]1g**

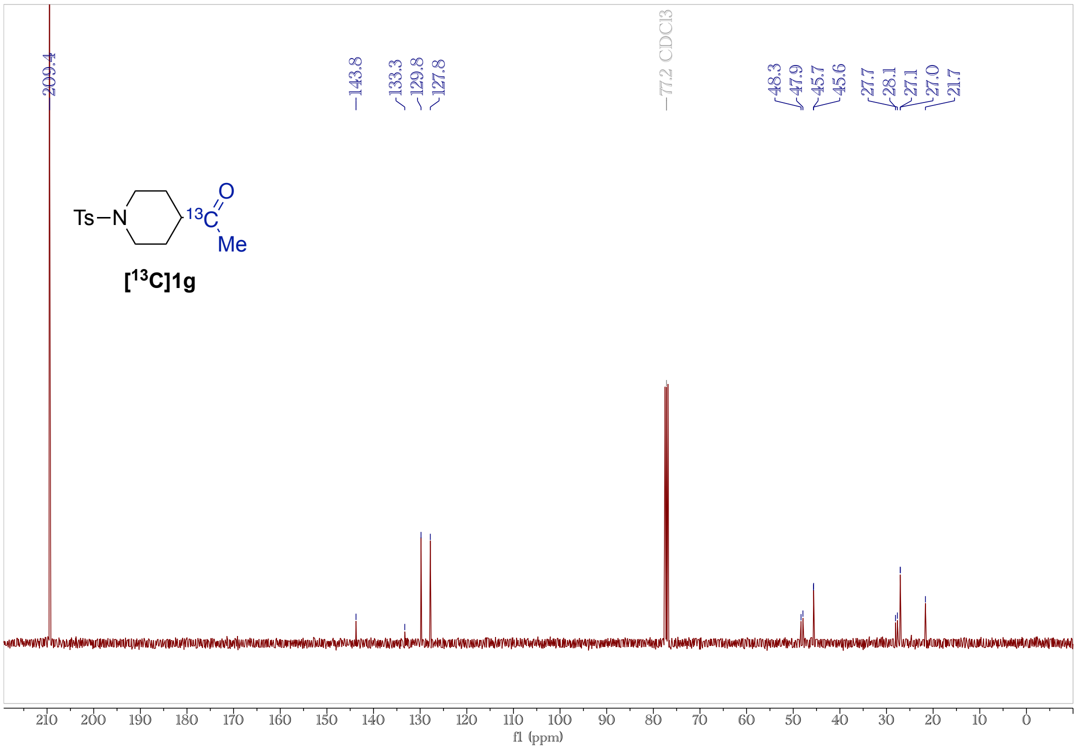

^{1}H NMR of **[13C]1h**

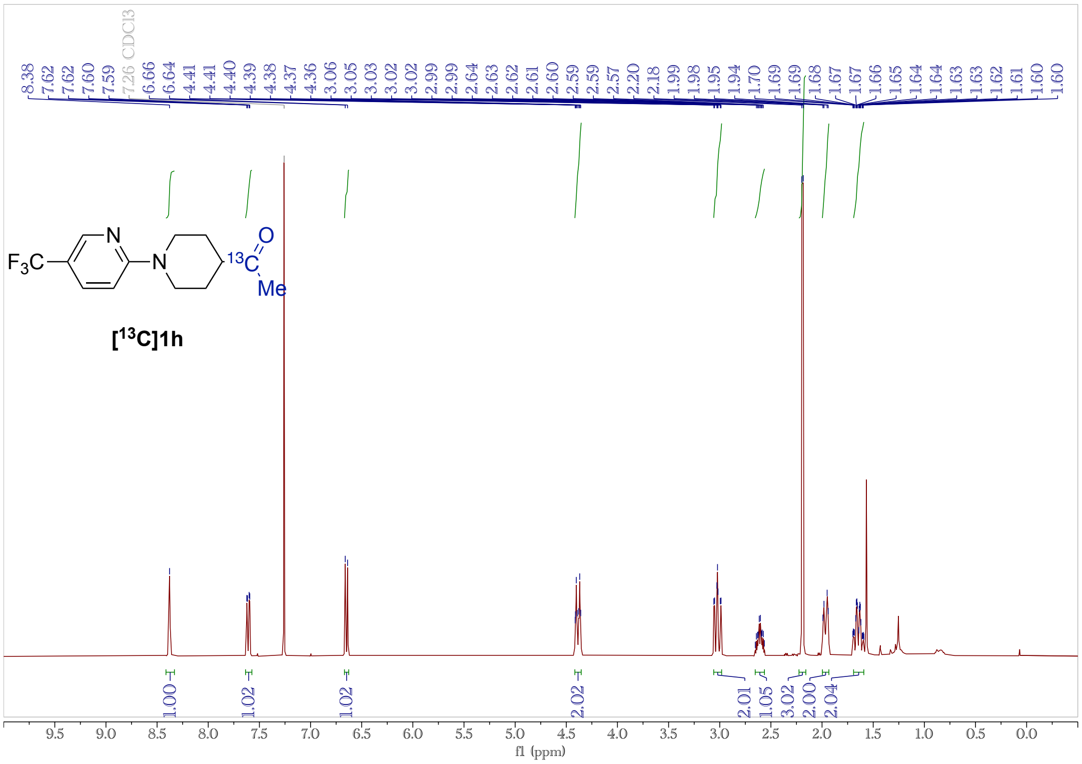

^{13}C NMR of **[13C]1h**

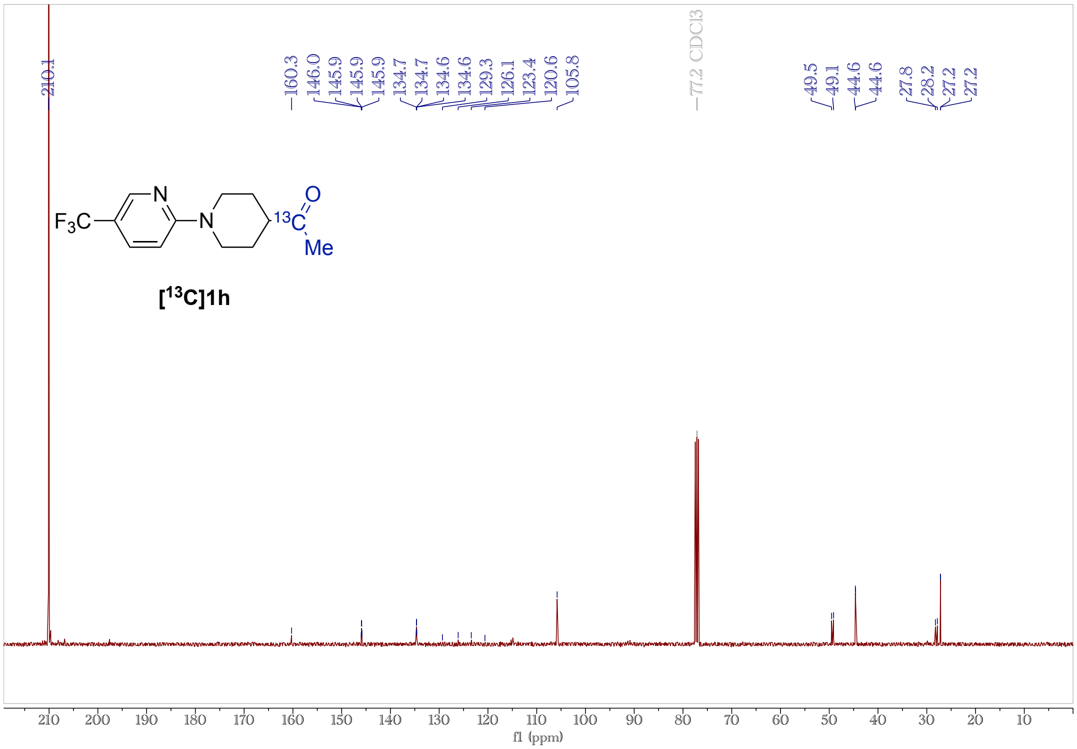

^{19}F NMR of **[13C]1h**

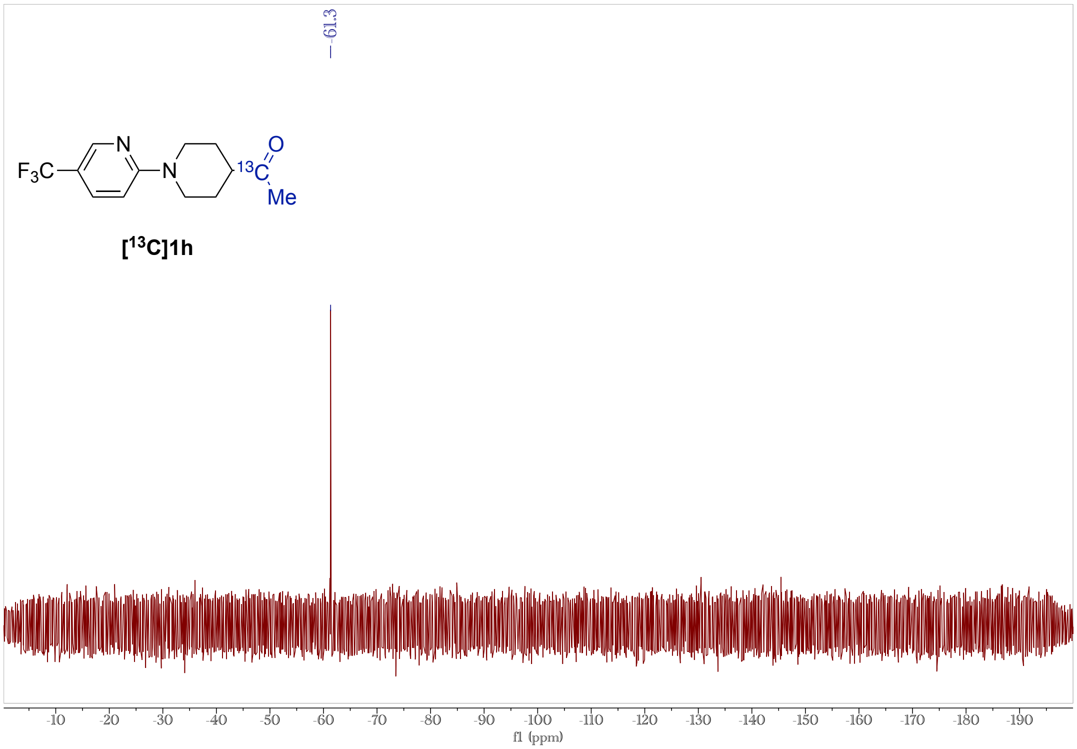

^{1}H NMR of **[13C]1i**

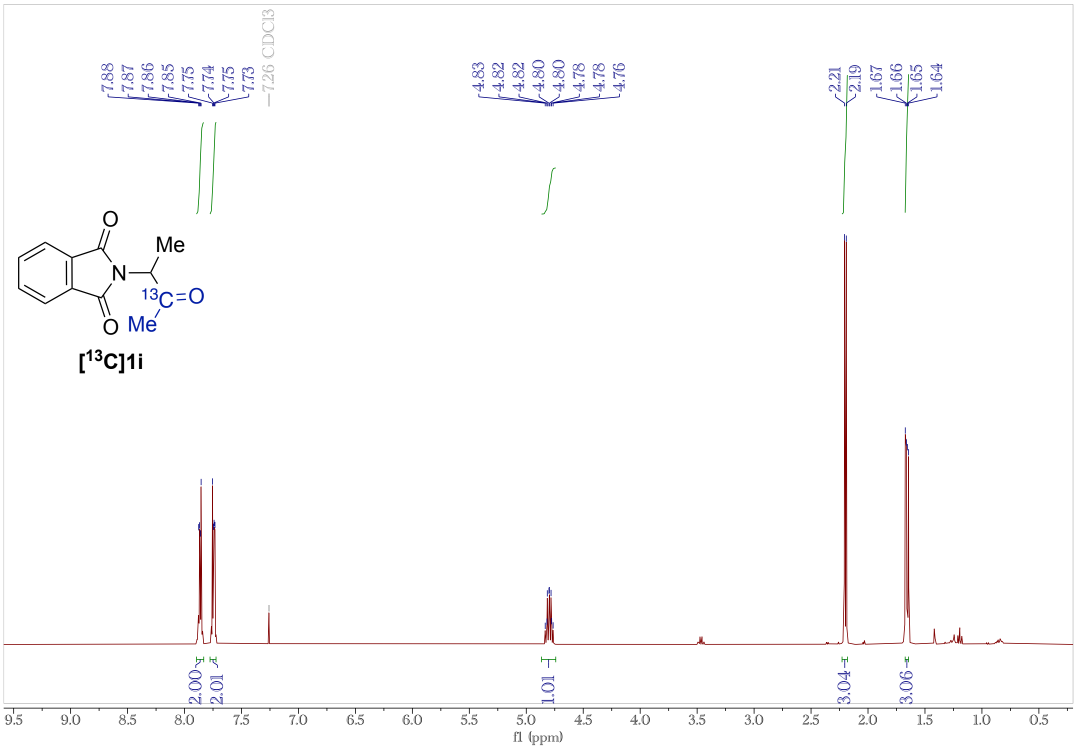

^{13}C NMR of **[13C]1i**

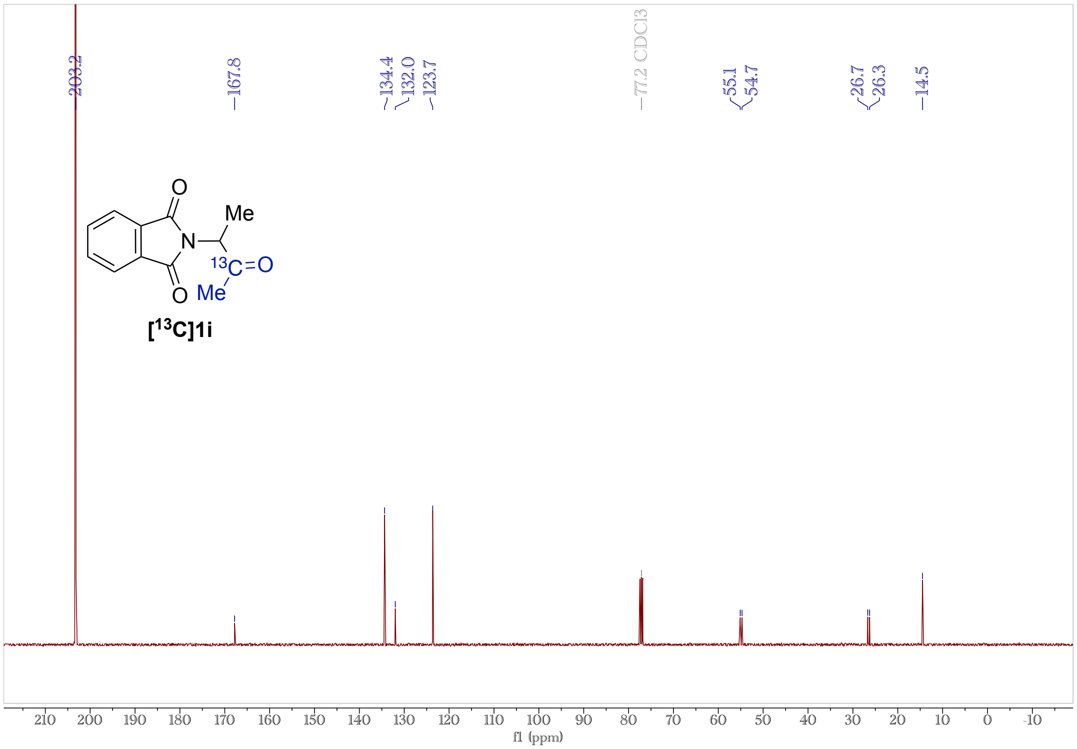

^{1}H NMR of **[13C]1j**

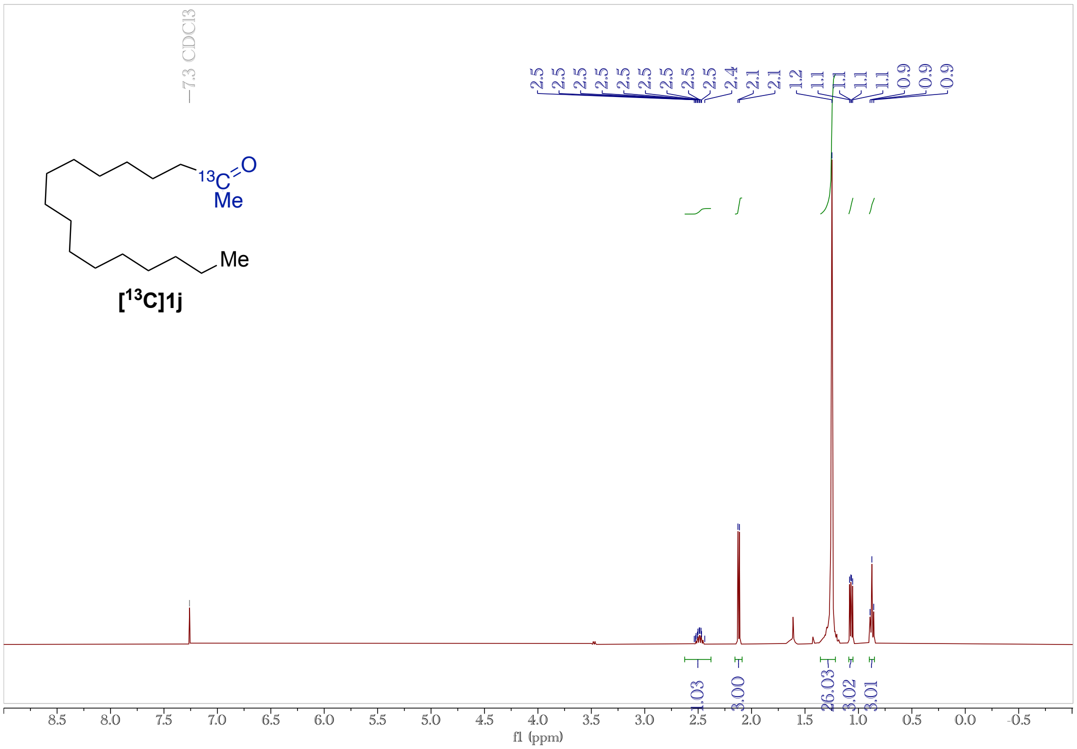

^{13}C NMR of **[13C]1j**

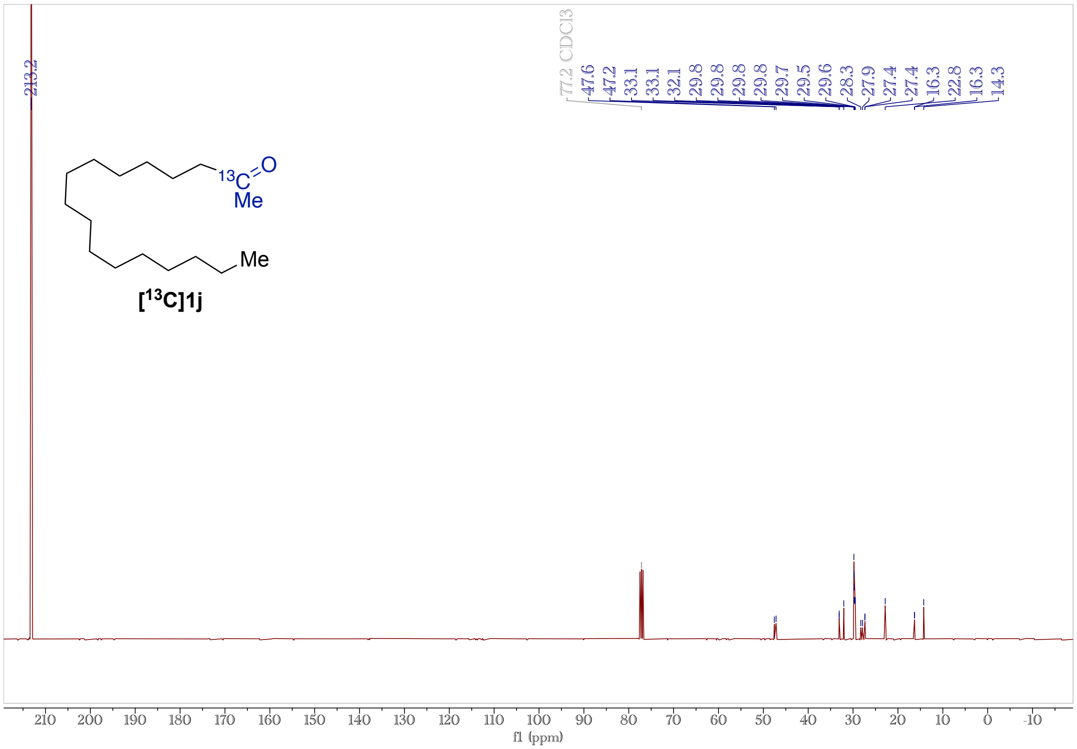

^{1}H NMR of **[13C]1k**

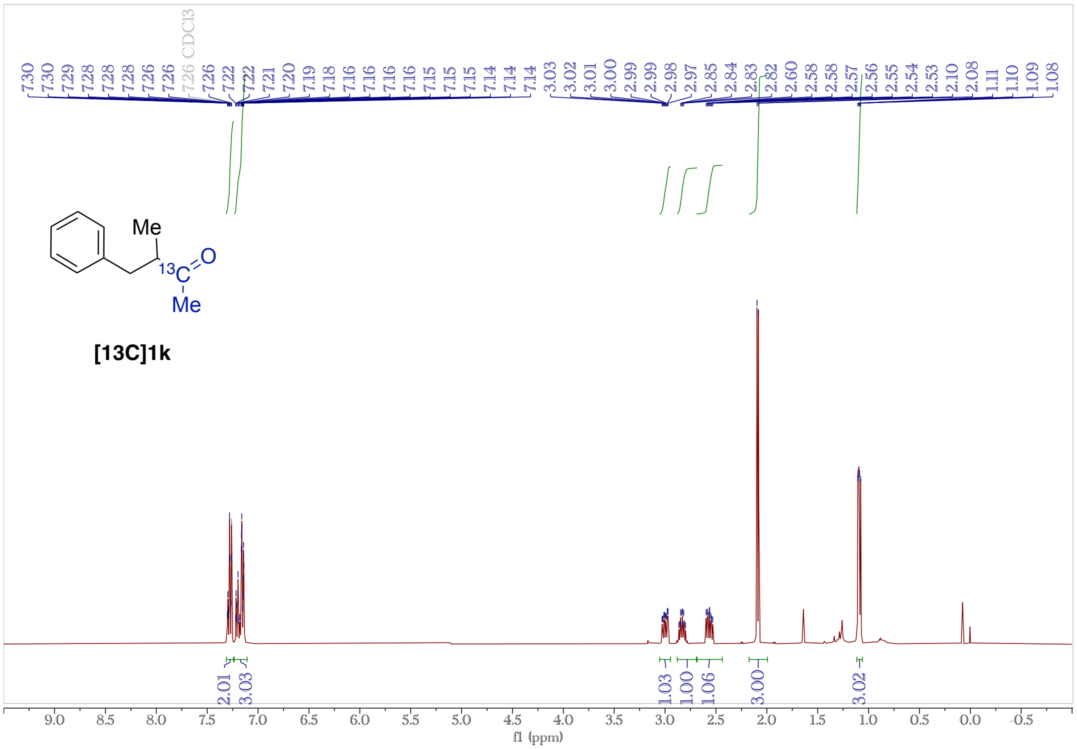

^{13}C NMR of **[13C]1k**

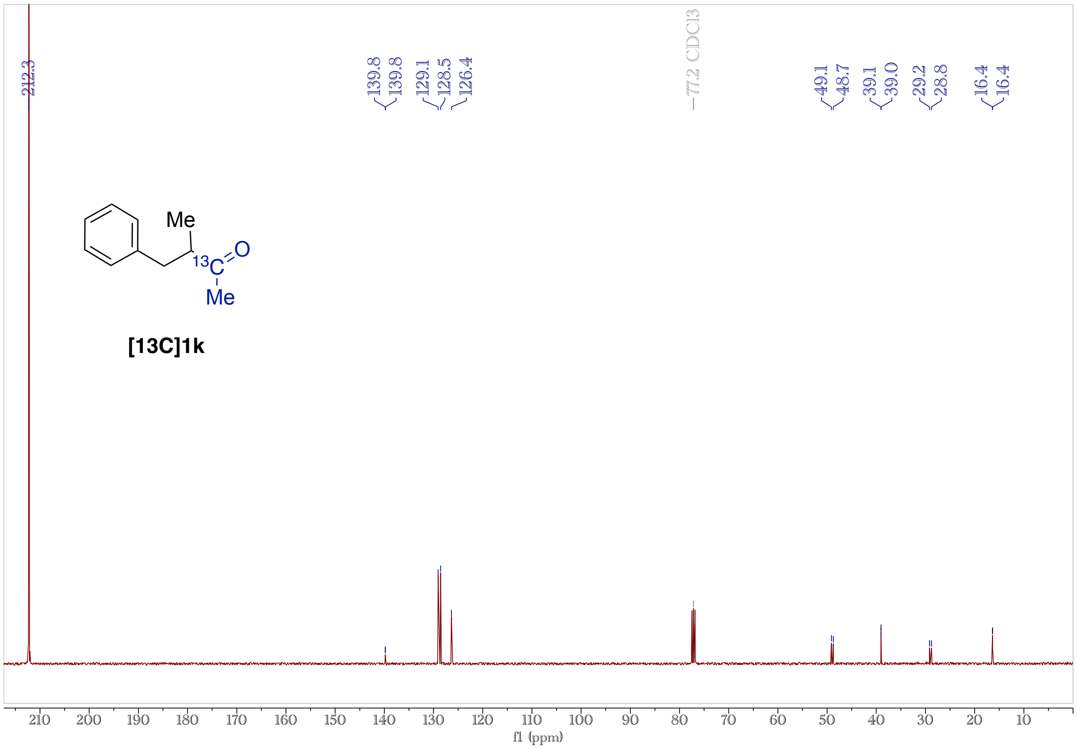

^{1}H NMR of **[13C]1l**

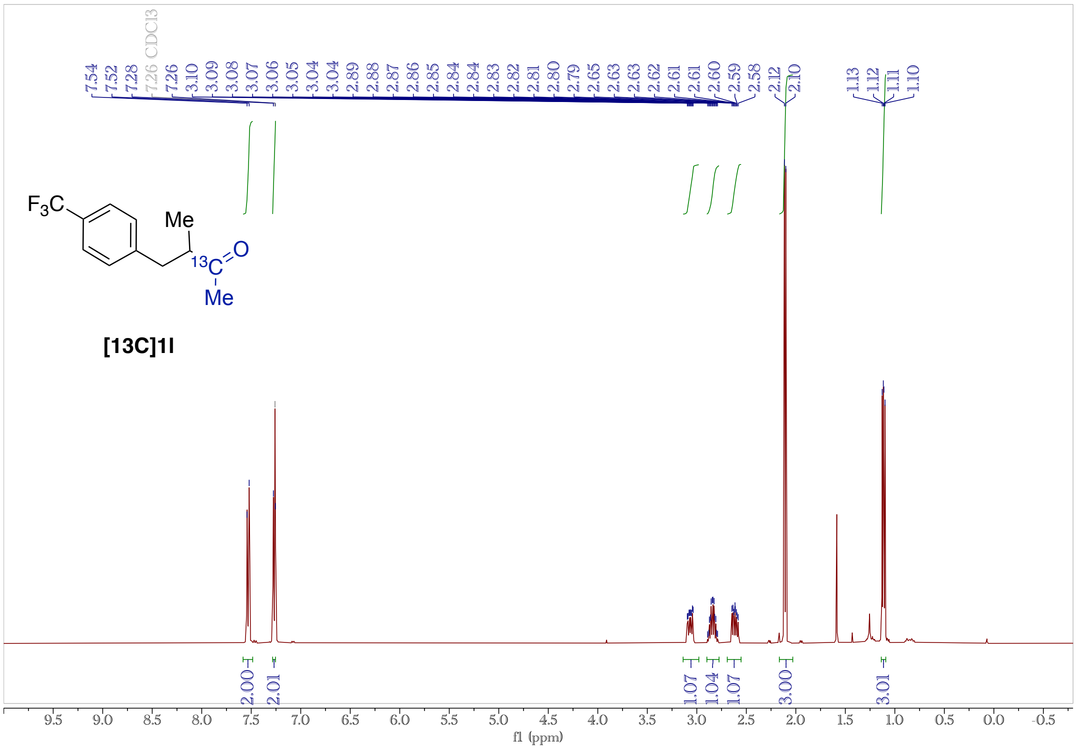

^{13}C NMR of **[13C]1l**

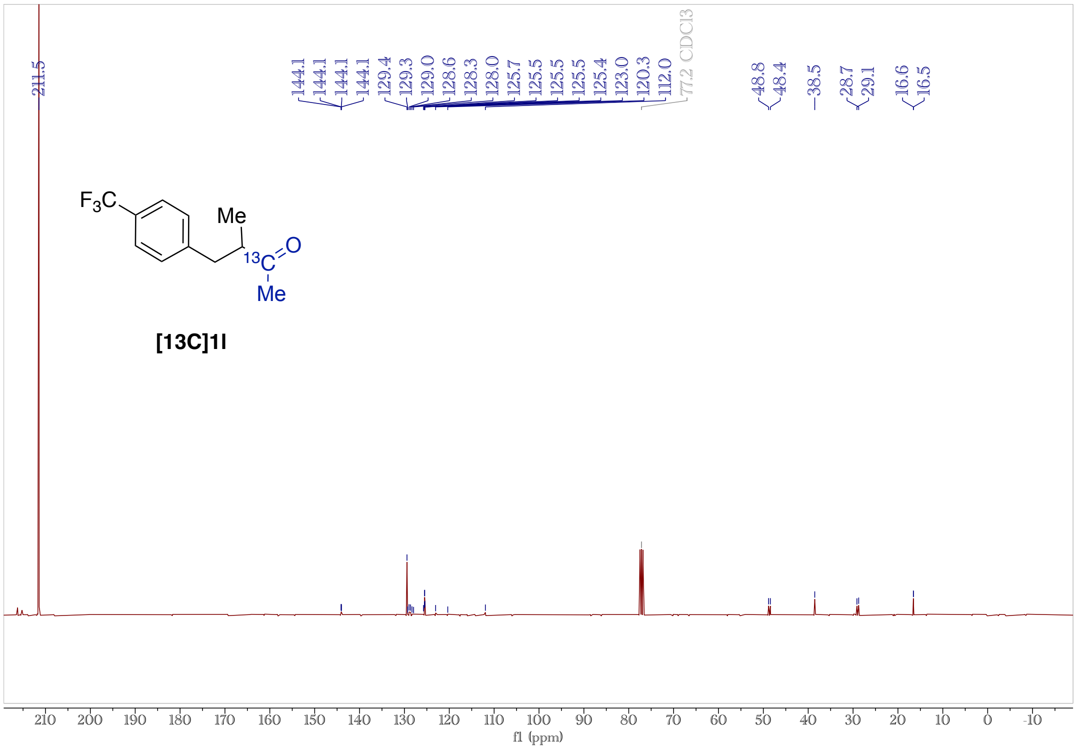

^{19}F NMR of **[13C]1l**

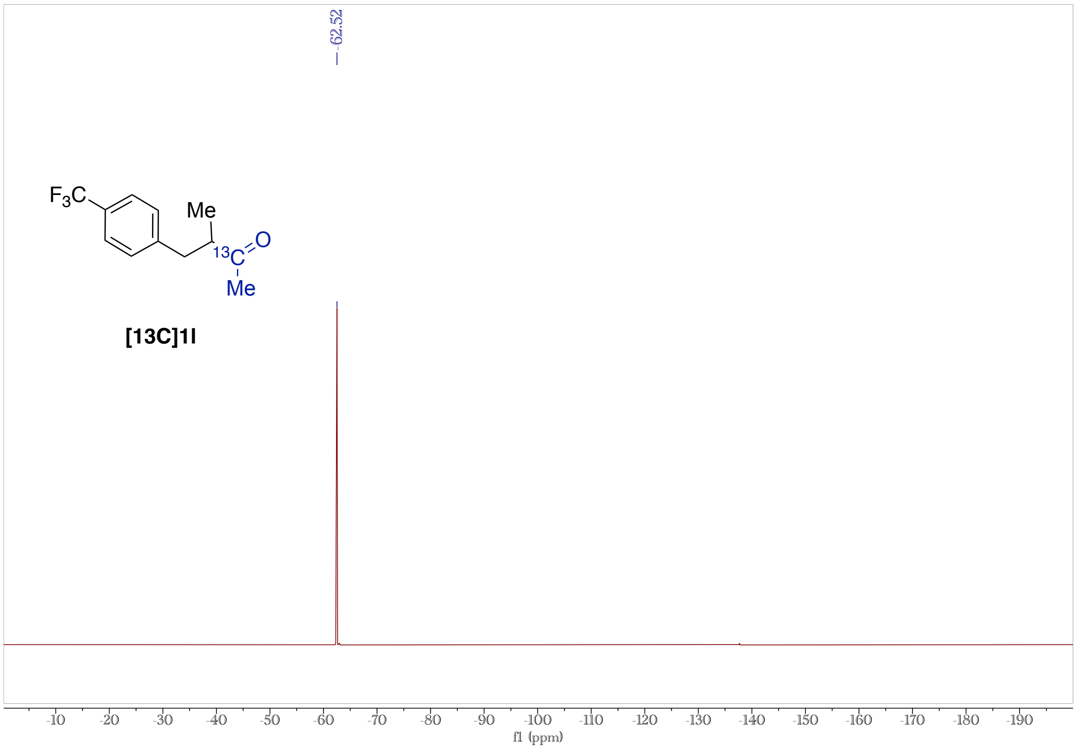

^1H NMR of **[13C]1m**

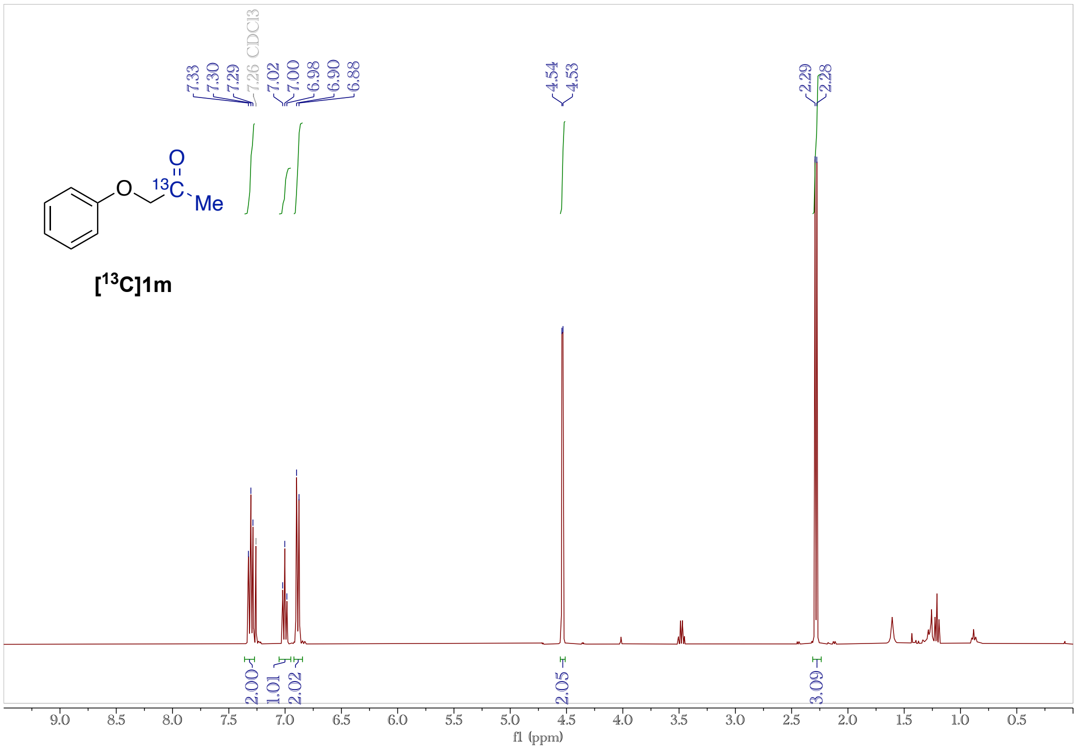

^{13}C NMR of **[13C]1m**

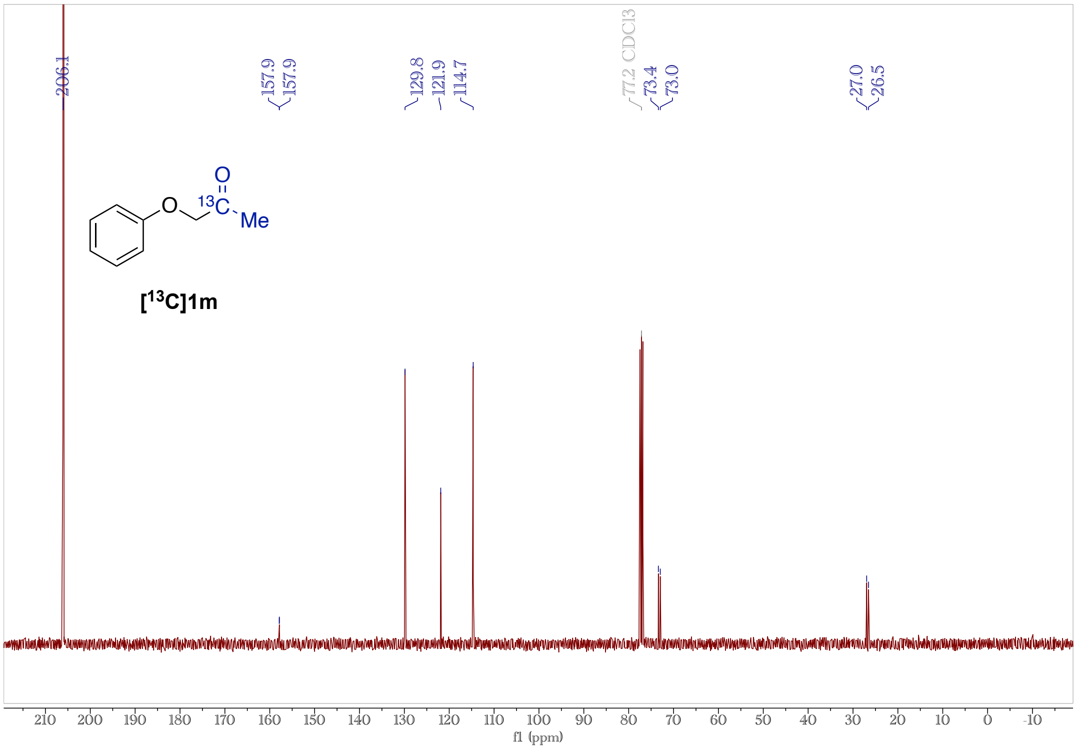

^{1}H NMR of **[13C]1n**

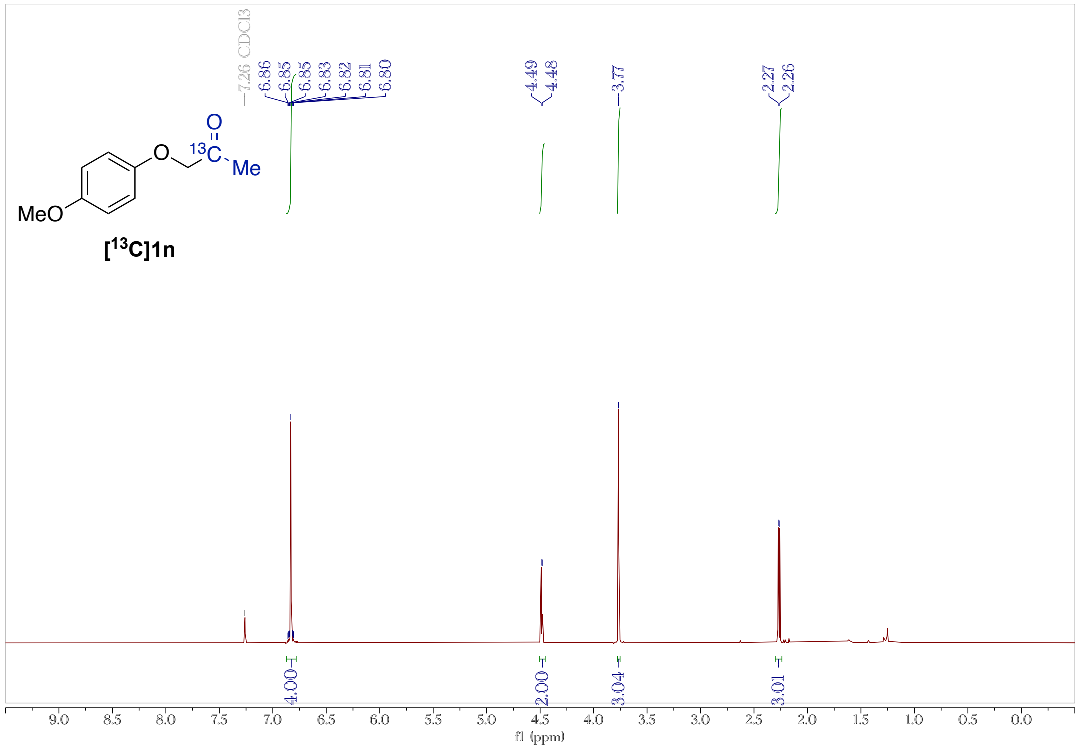

^{13}C NMR of **[13C]1n**

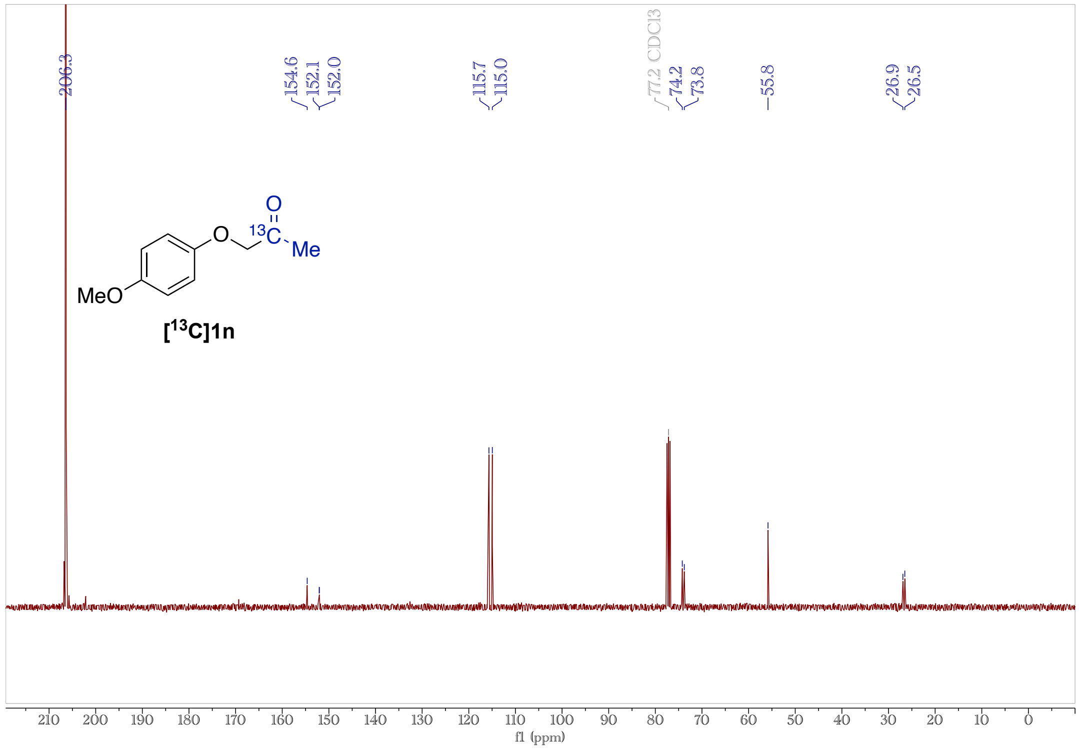

^{1}H NMR of **[13C]1o**

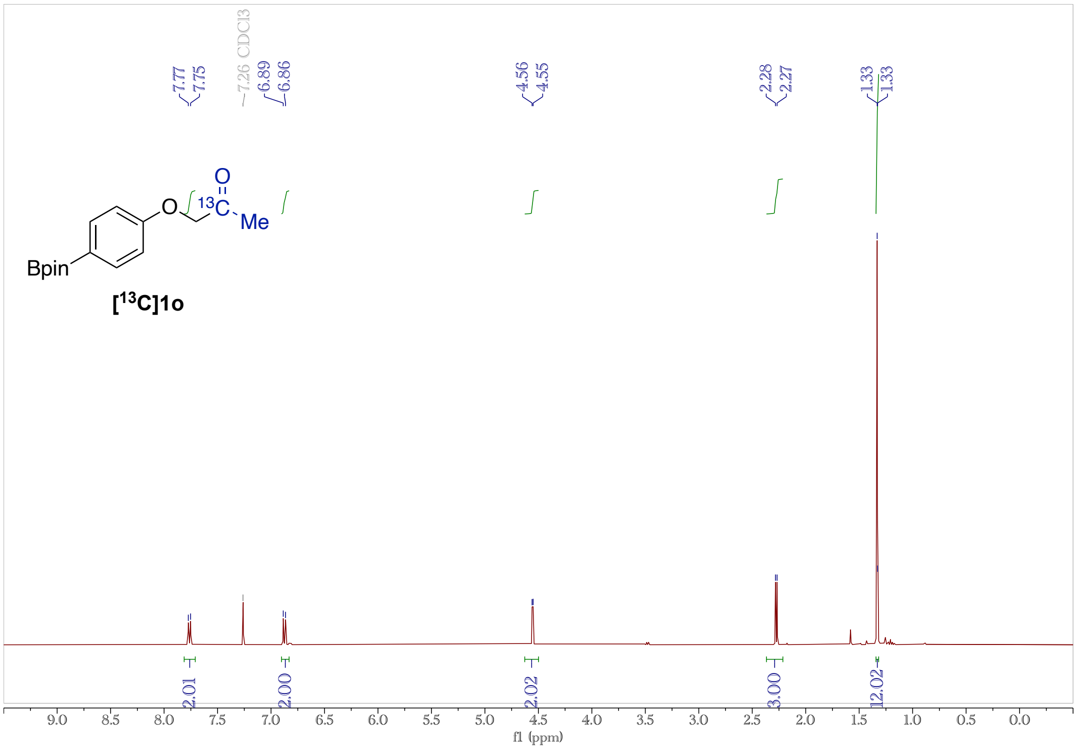

^{13}C NMR of **[13C]1o**

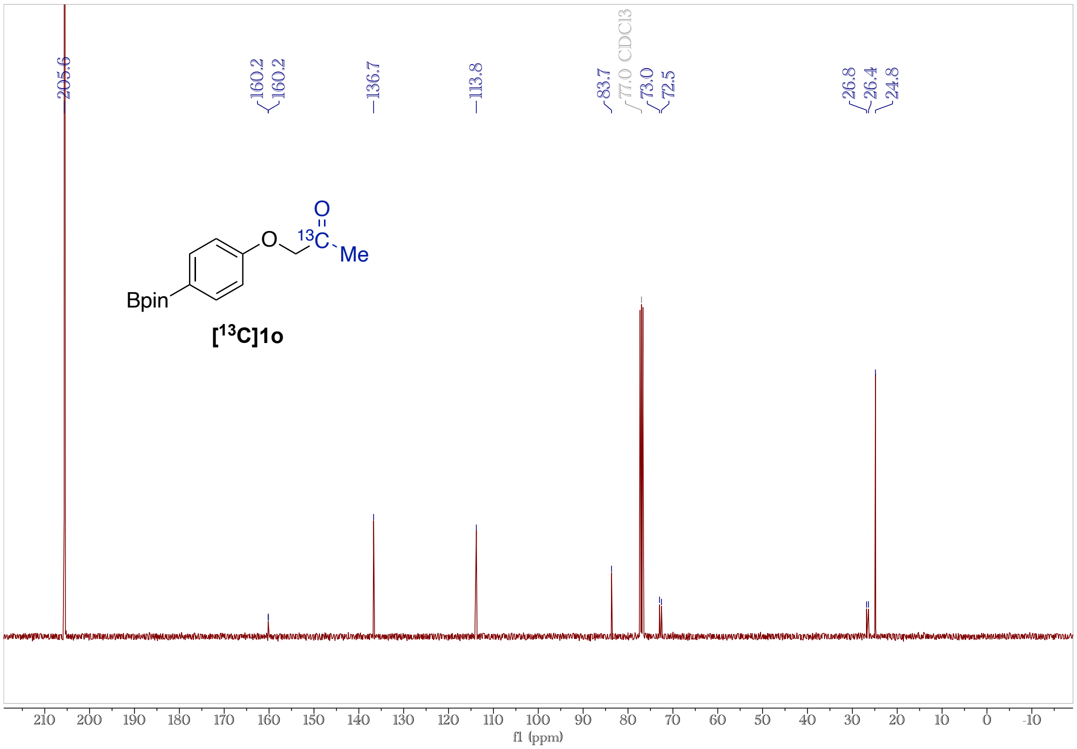

^{11}B NMR of **[13C]1o**

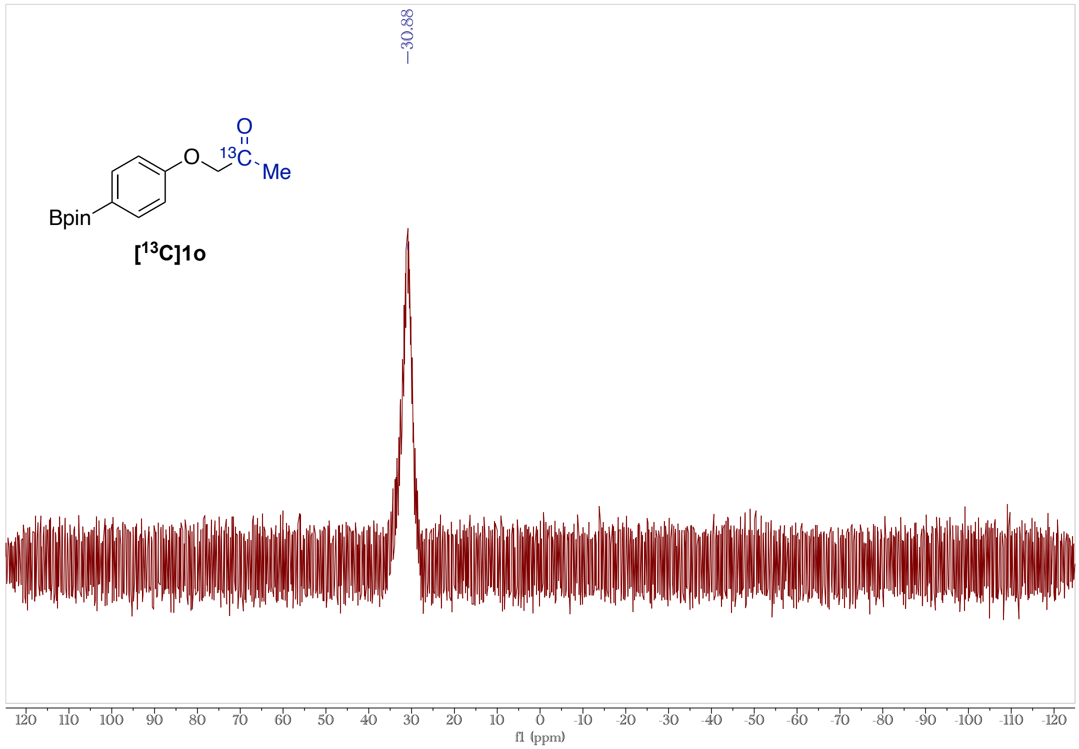

^{1}H NMR of **[13C]1p**

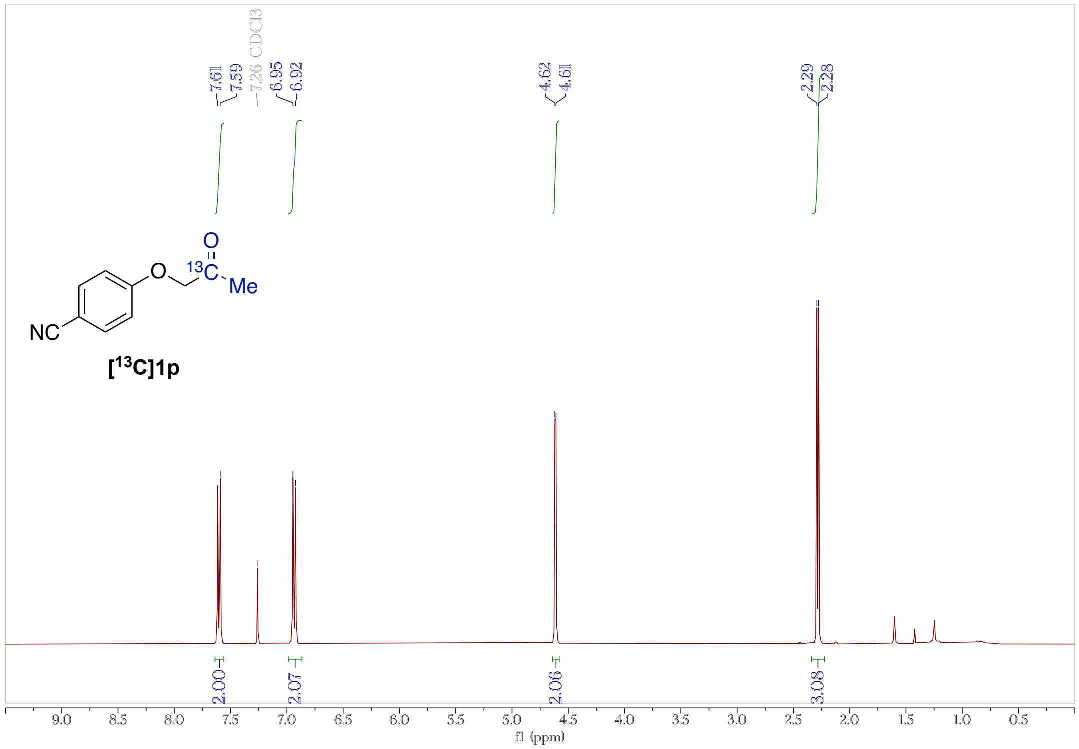

^{13}C NMR of **[13C]1p**

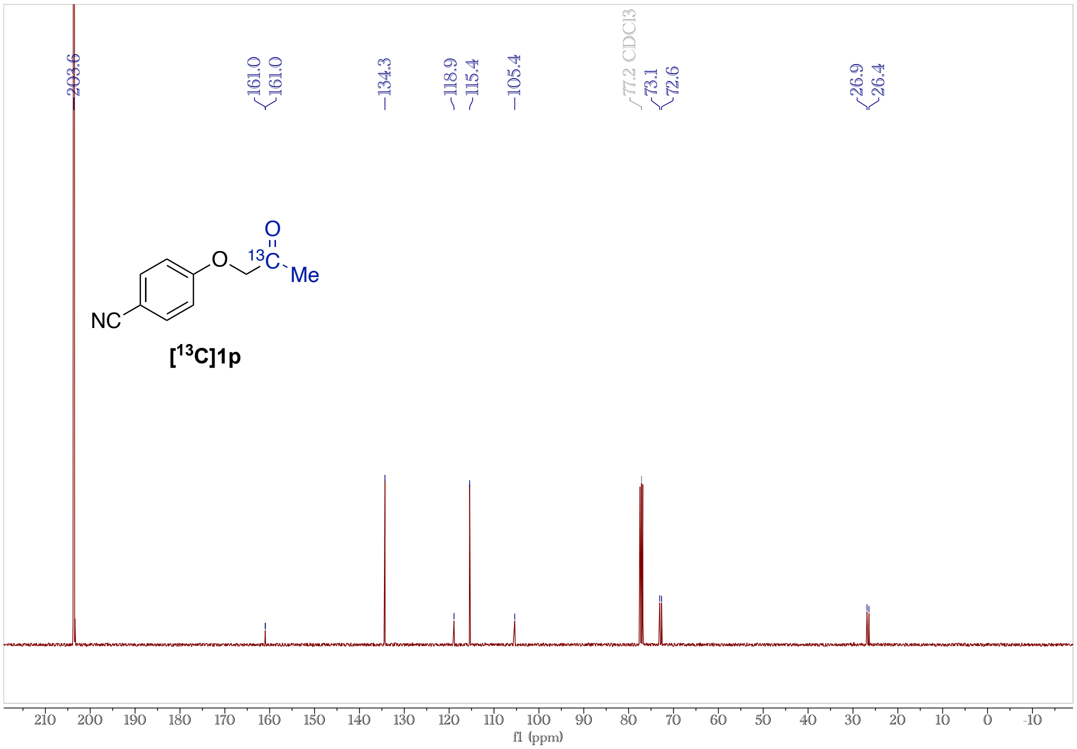

^{1}H NMR of **[13C]1q**

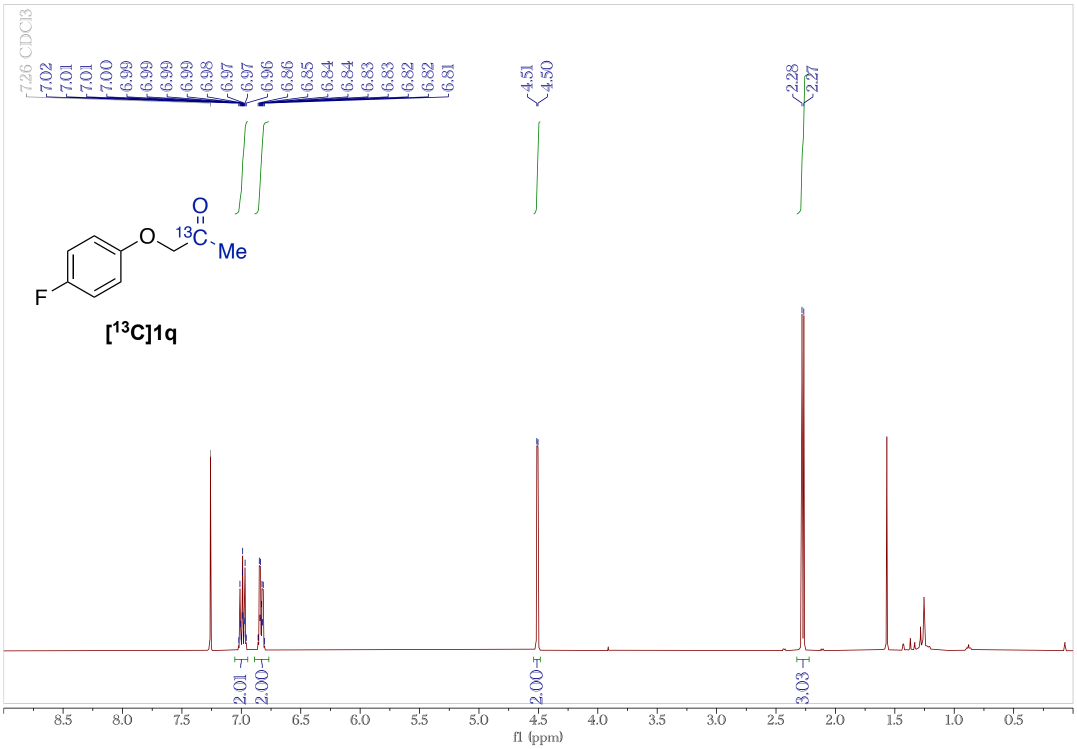

^{13}C NMR of **[13C]1q**

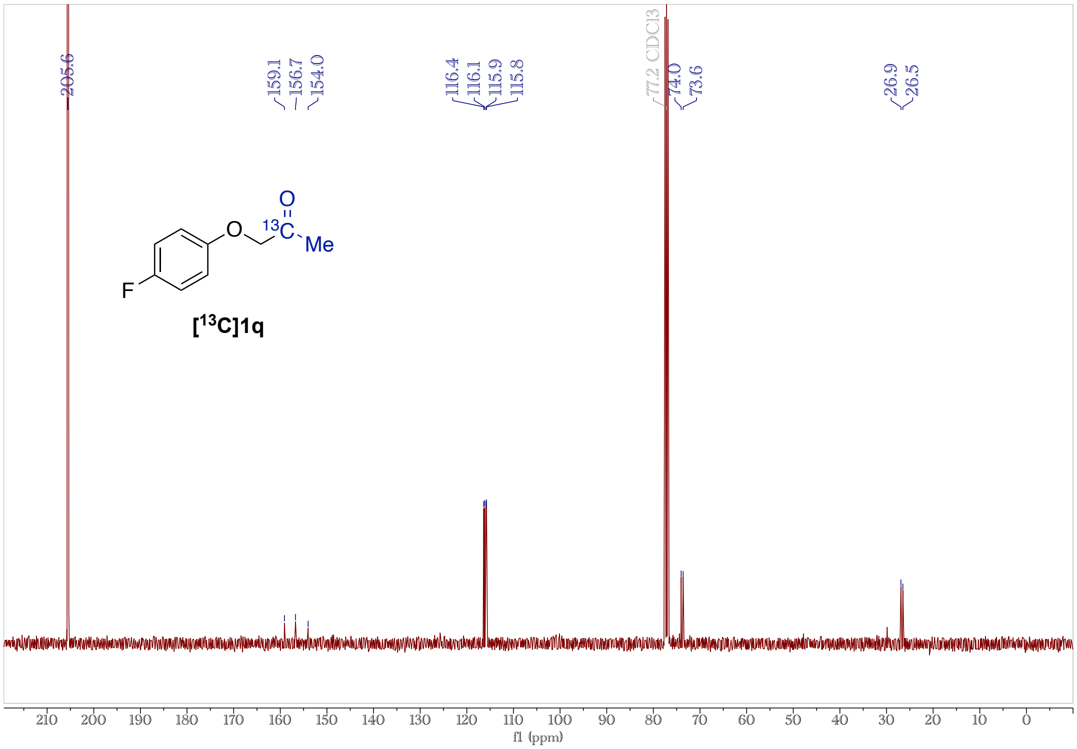

^{19}F NMR of **[13C]1q**

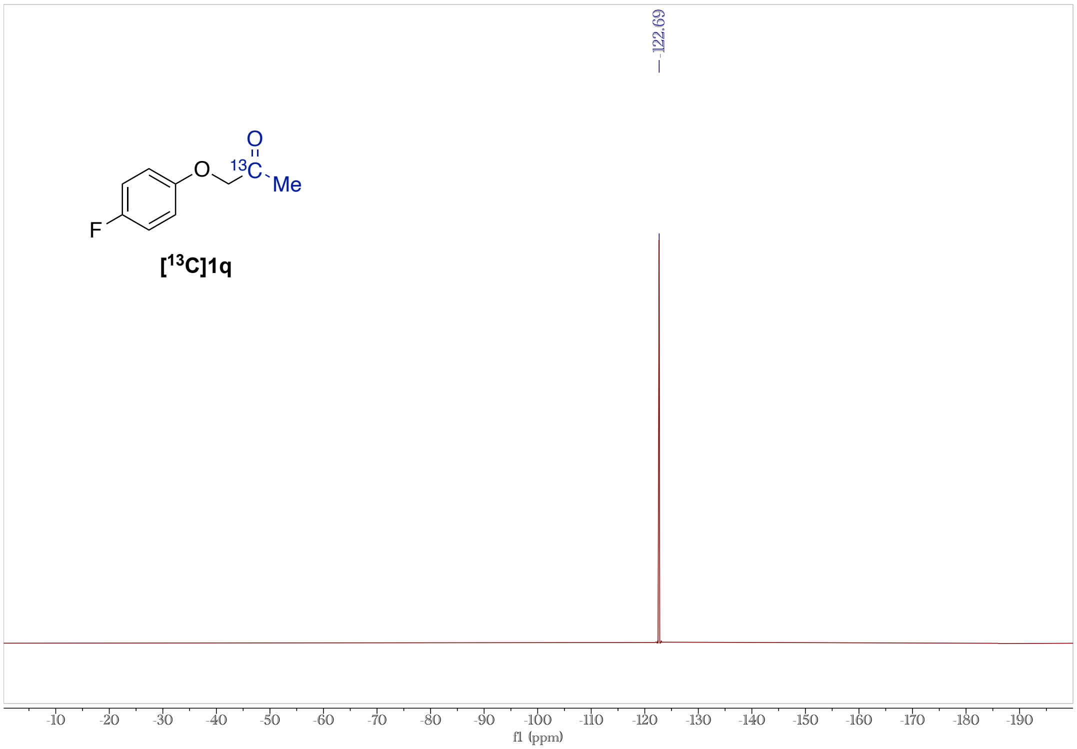

^{1}H NMR of **[13C]1r**

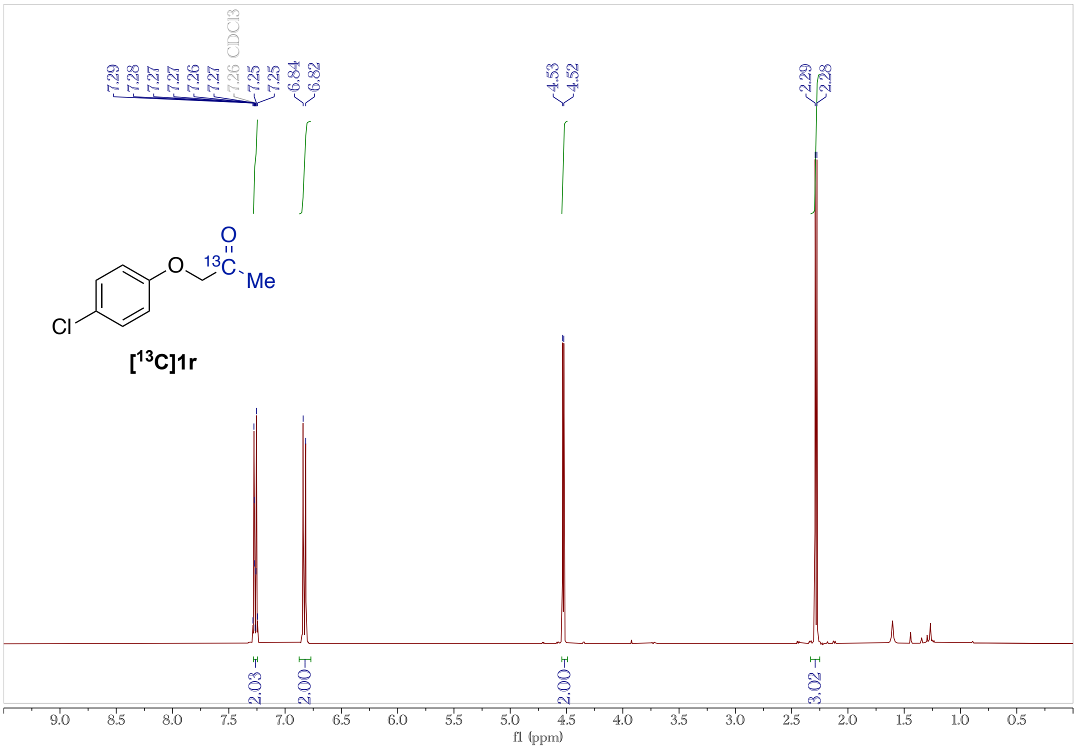

^{13}C NMR of **[13C]1r**

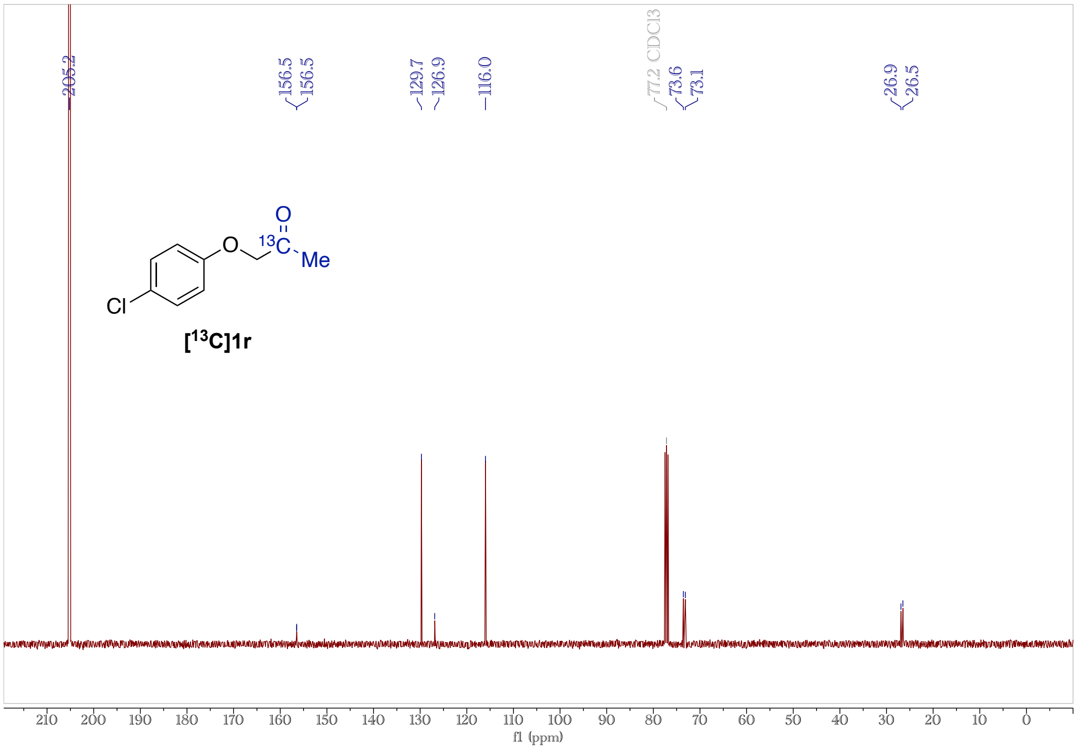

^{1}H NMR of **[13C]1s**

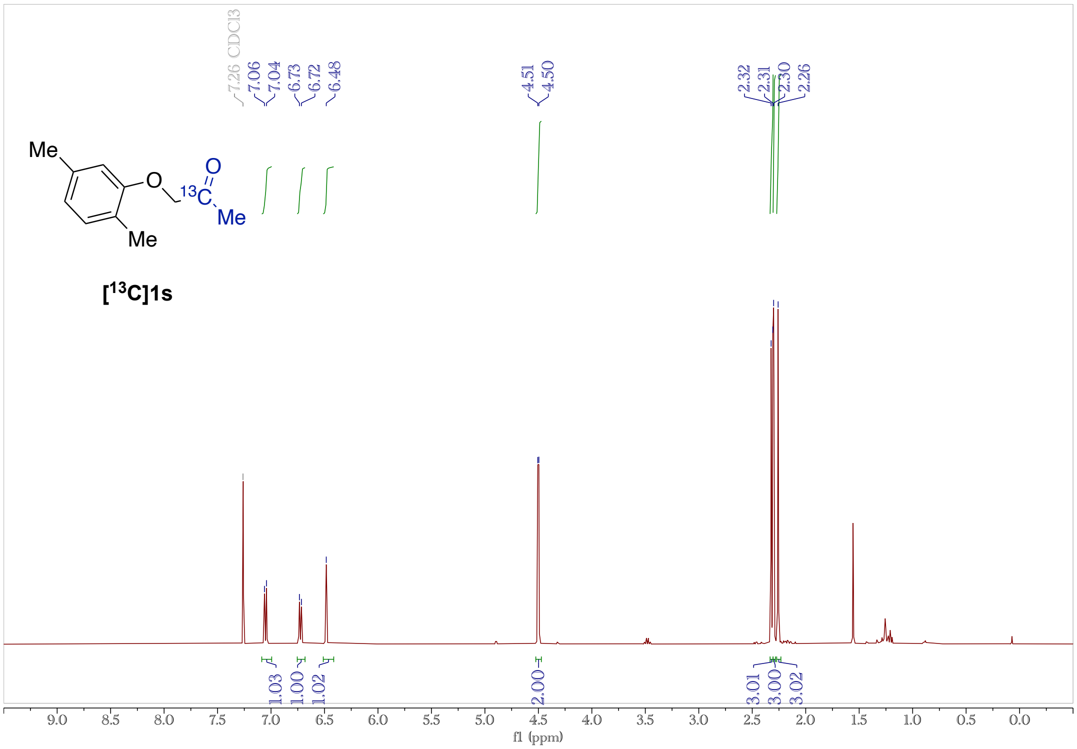

^{13}C NMR of **[13C]1s**

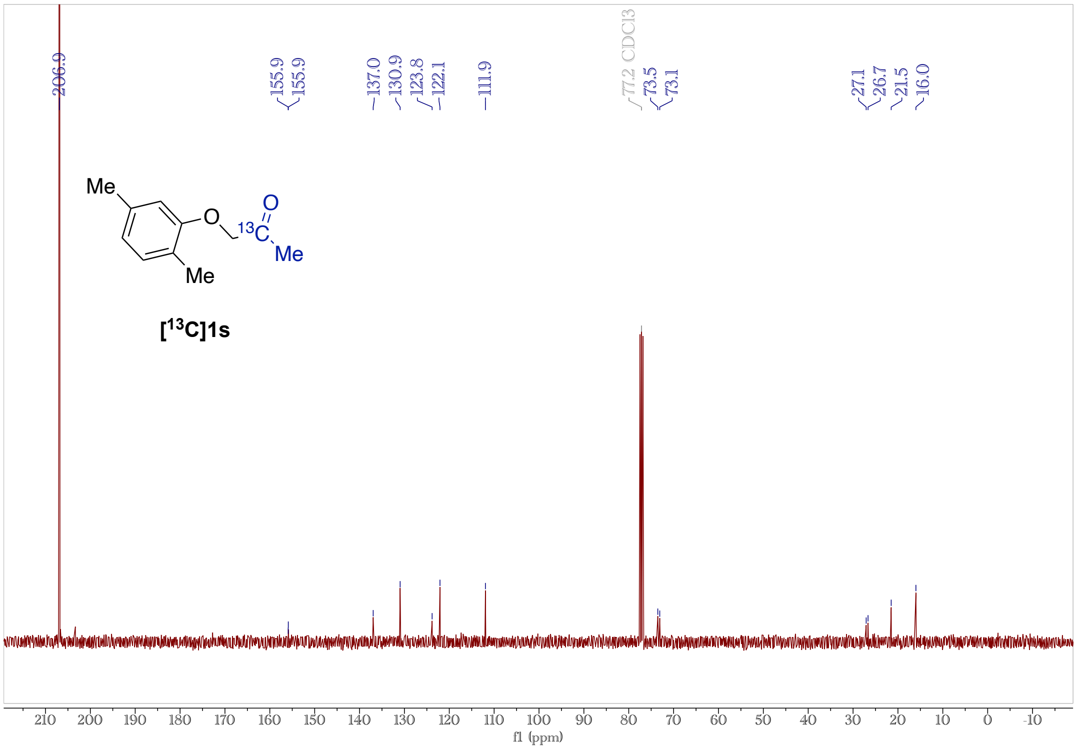

^{1}H NMR of **[13C]1t**

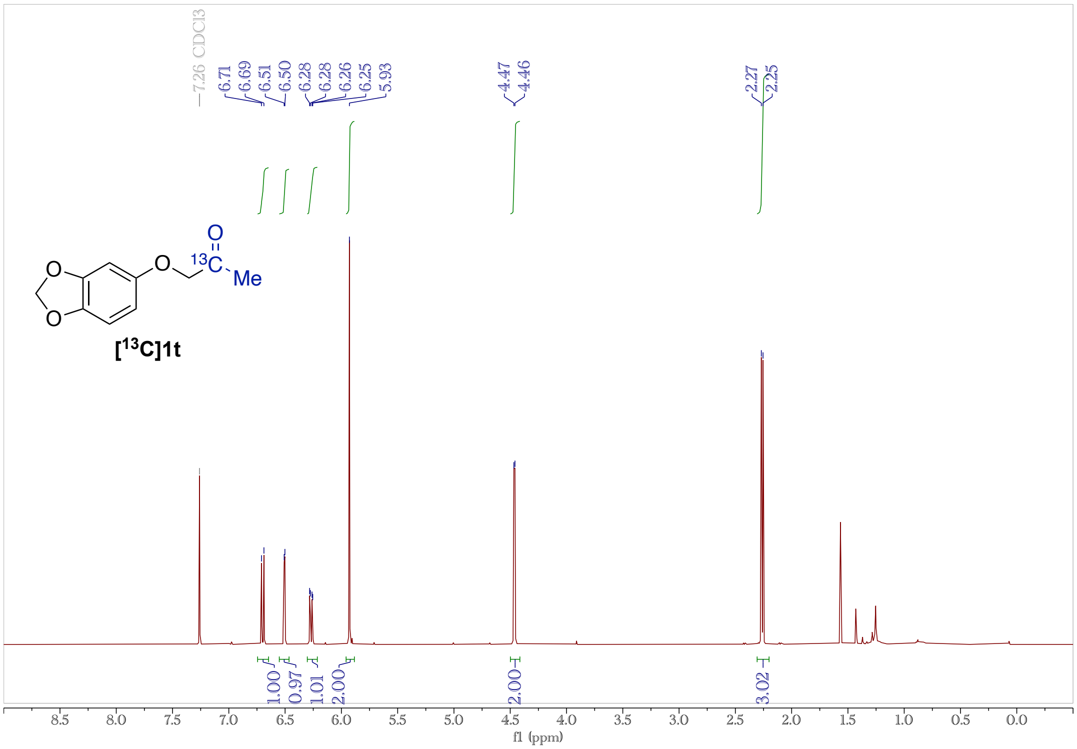

^{13}C NMR of **[13C]1t**

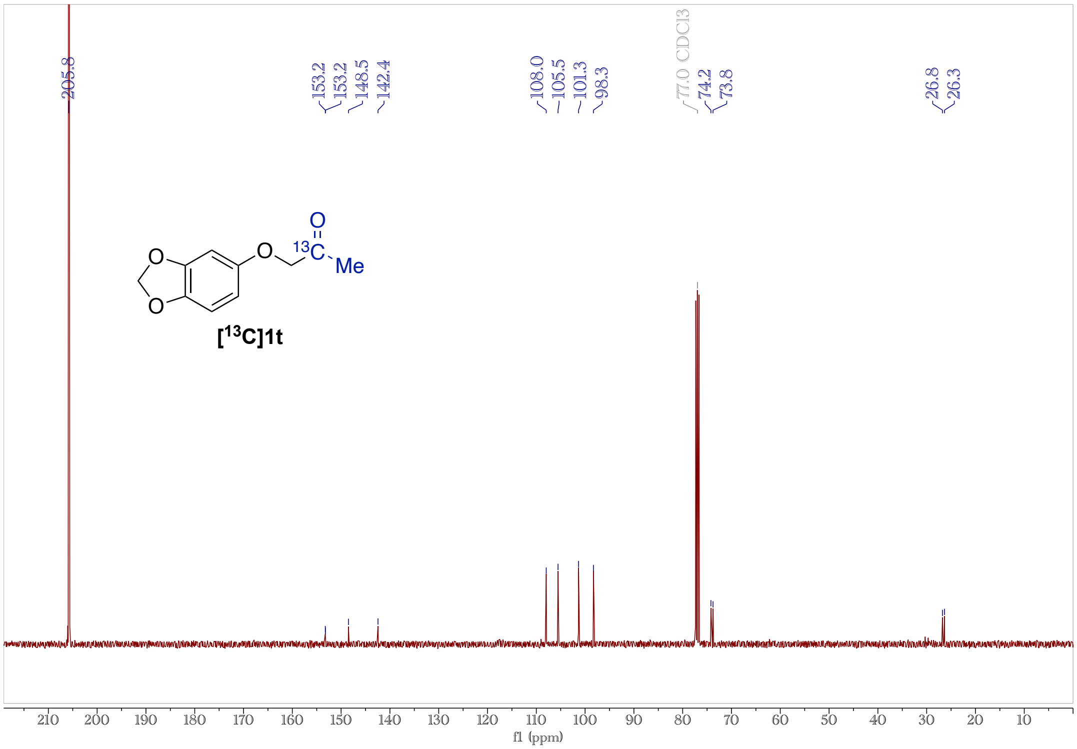

^{1}H NMR of **[13C]1u**

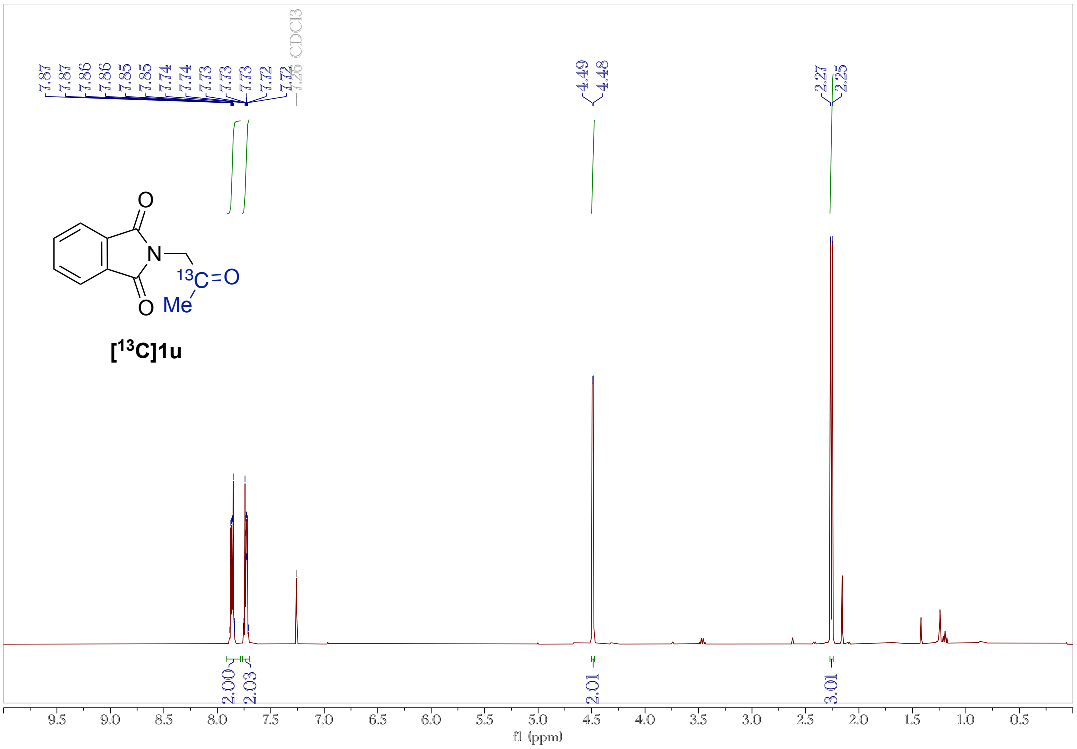

^{13}C NMR of **[13C]1u**

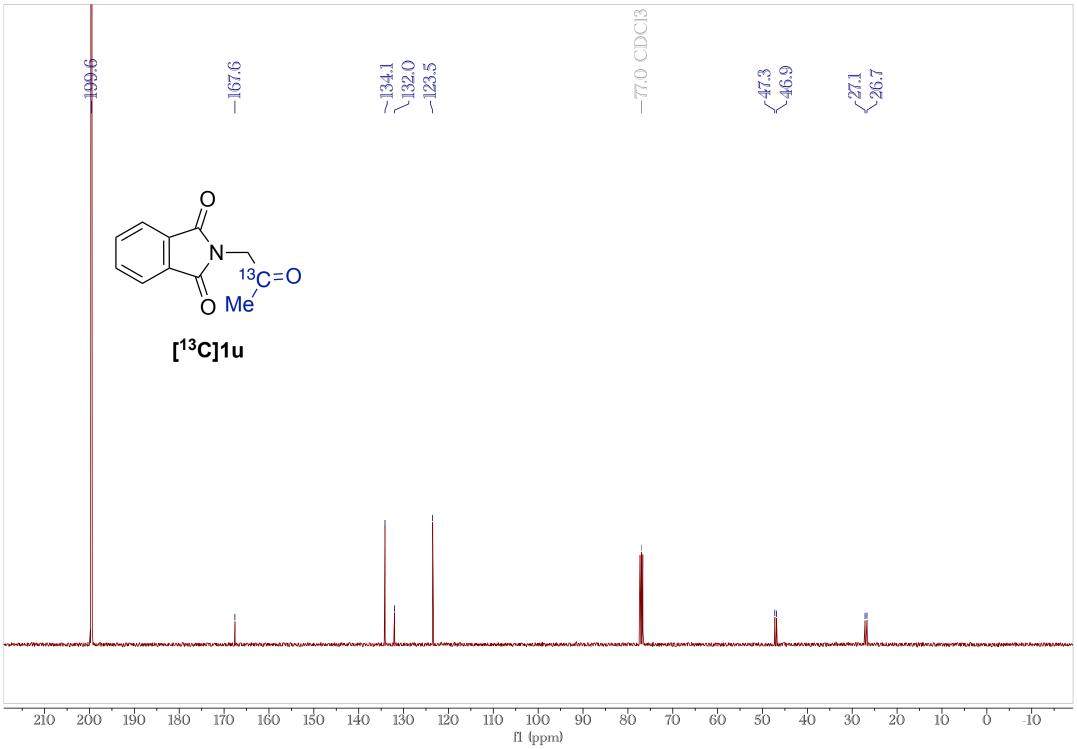

^{1}H NMR of **[13C]1v**

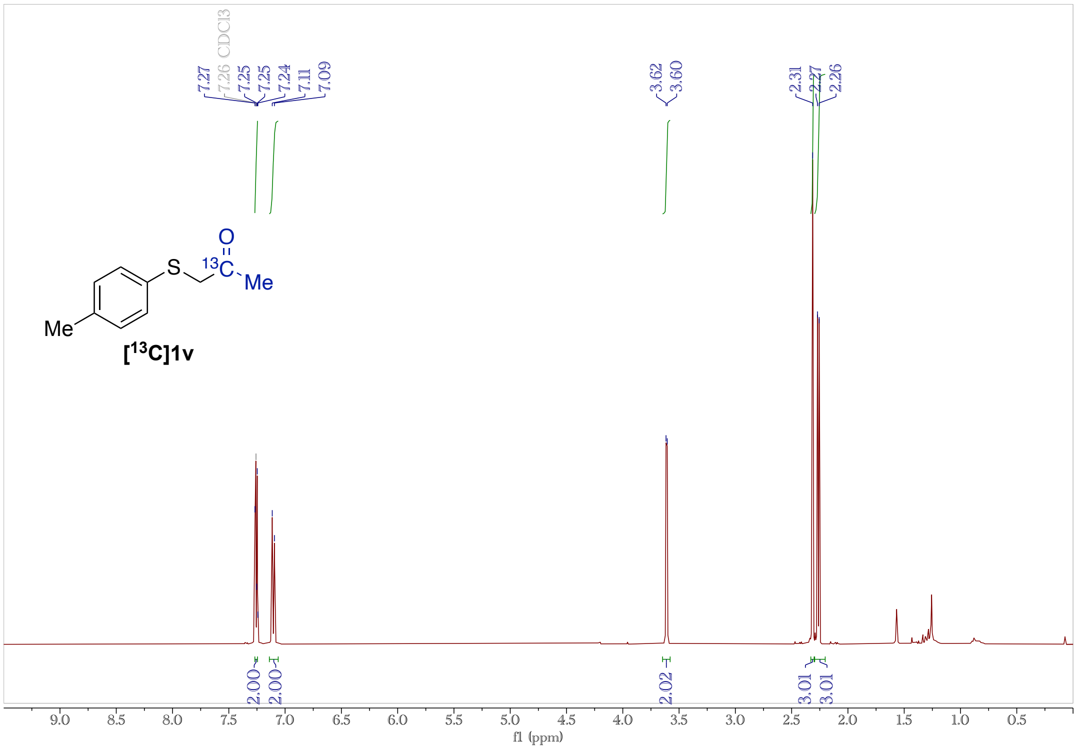

^{13}C NMR of **[13C]1v**

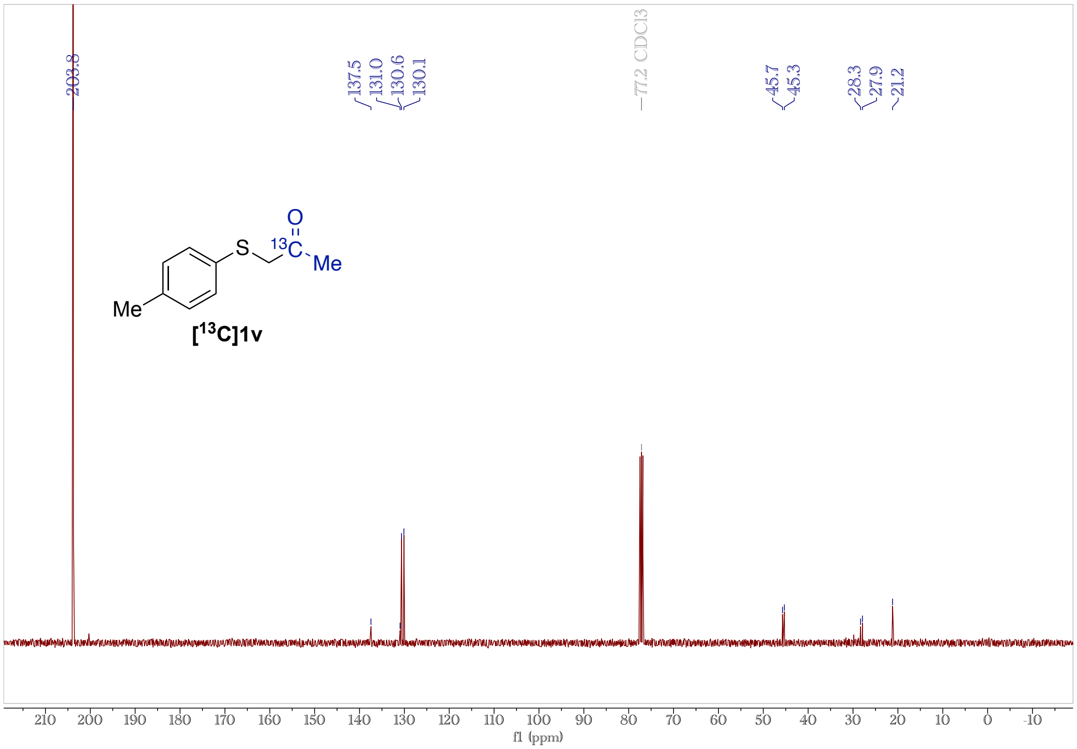

^{1}H NMR of **[13C]1w**

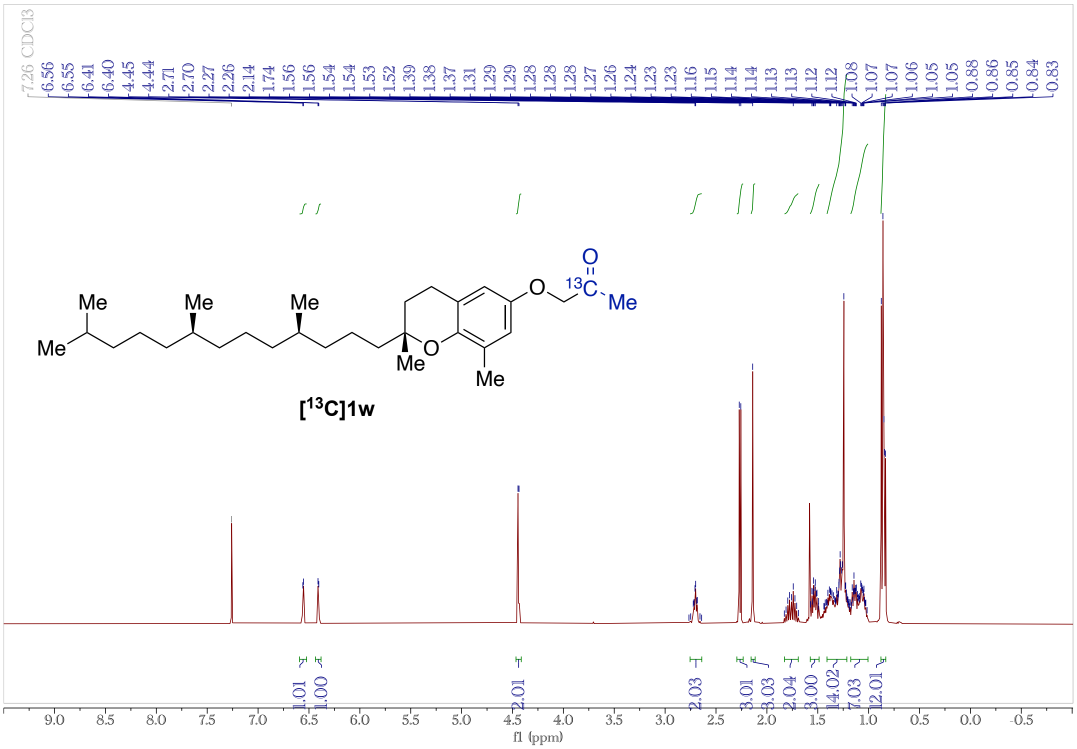

^{13}C NMR of **[13C]1w**

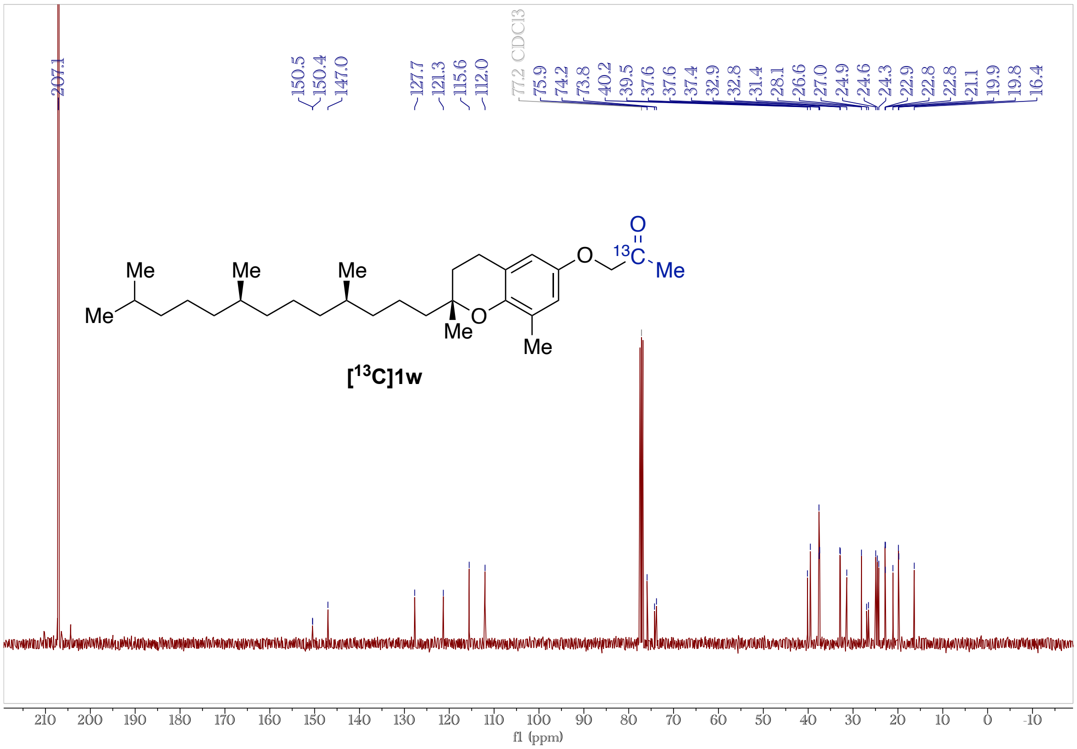

^{1}H NMR of **[13C]1x**

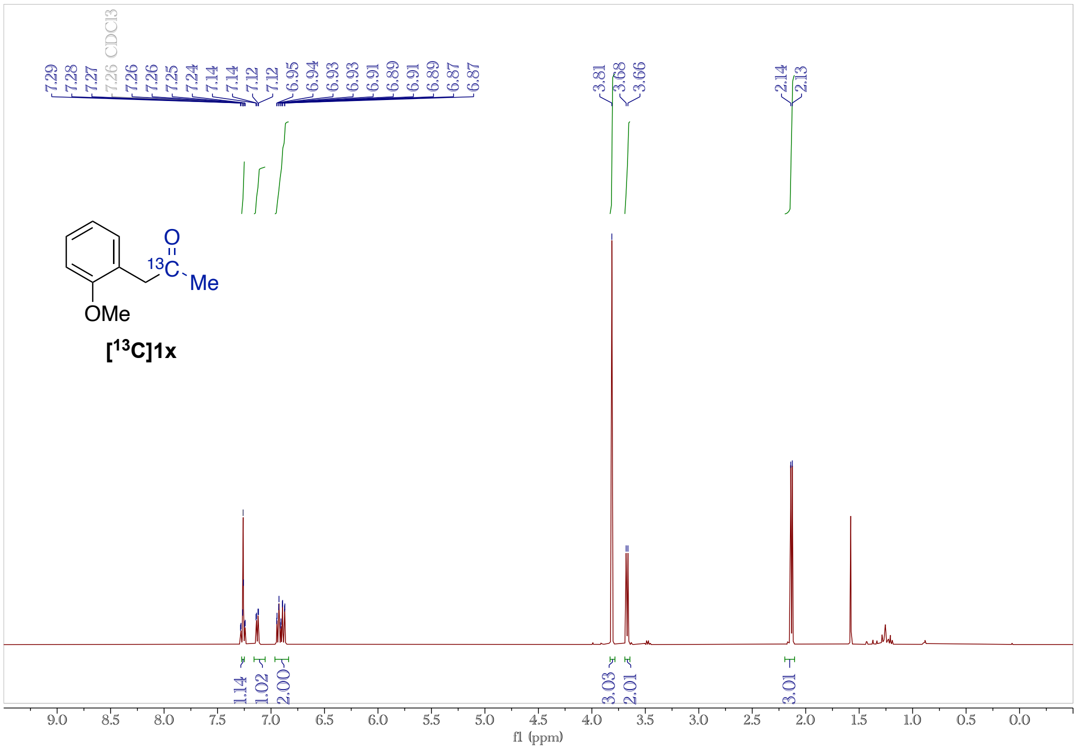

^{13}C NMR of **[13C]1x**

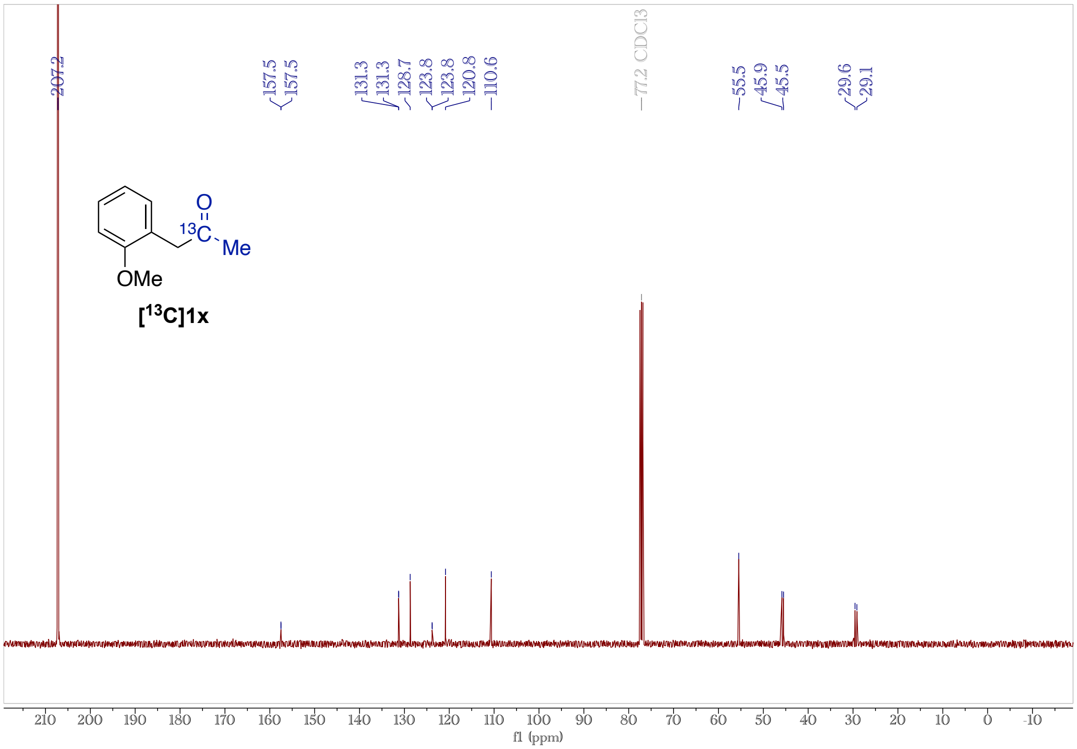

^{1}H NMR of **[13C]1y**

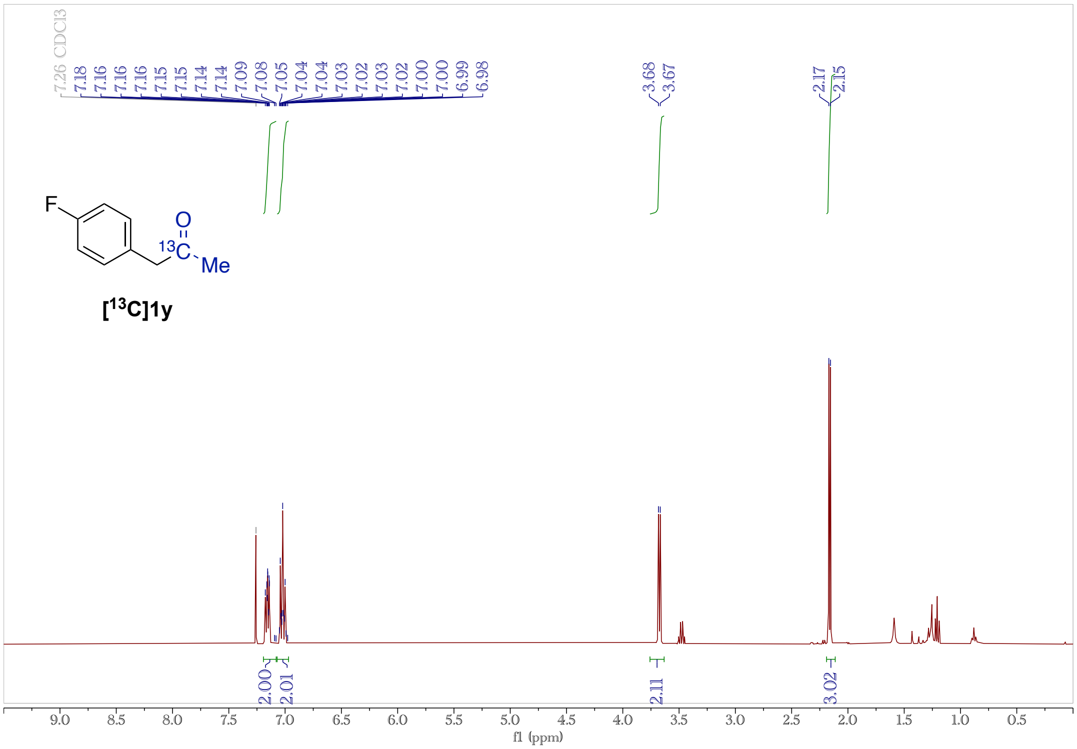

^{13}C NMR of **[13C]1y**

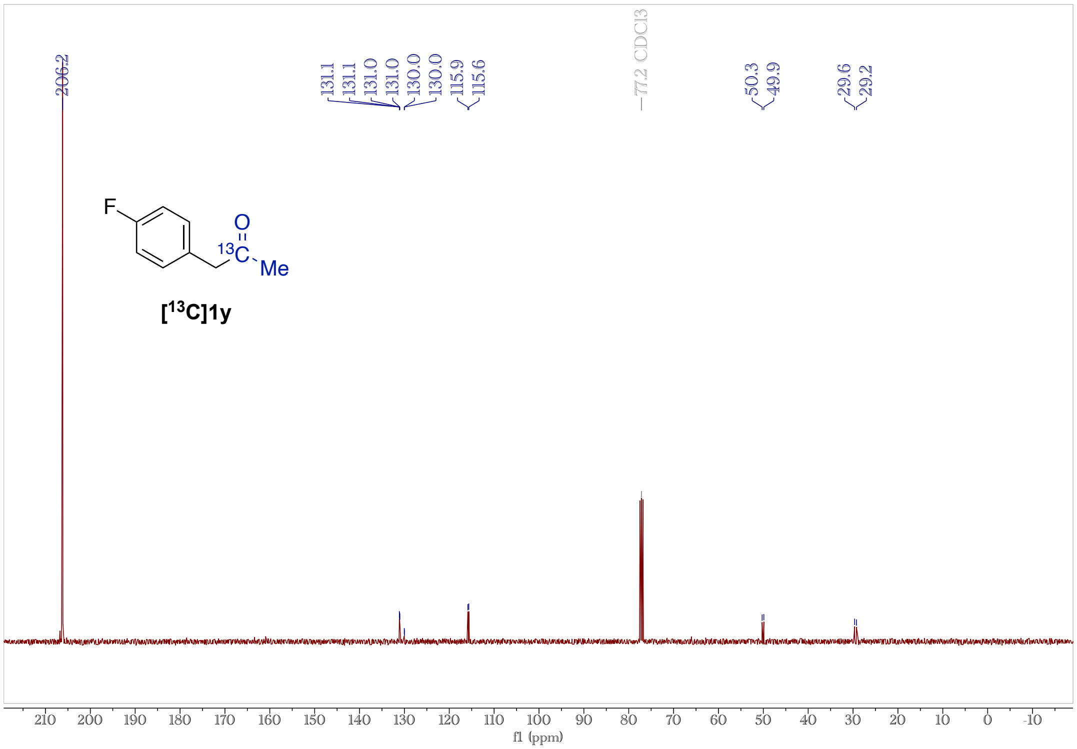

^{19}F NMR of **[13C]1y**

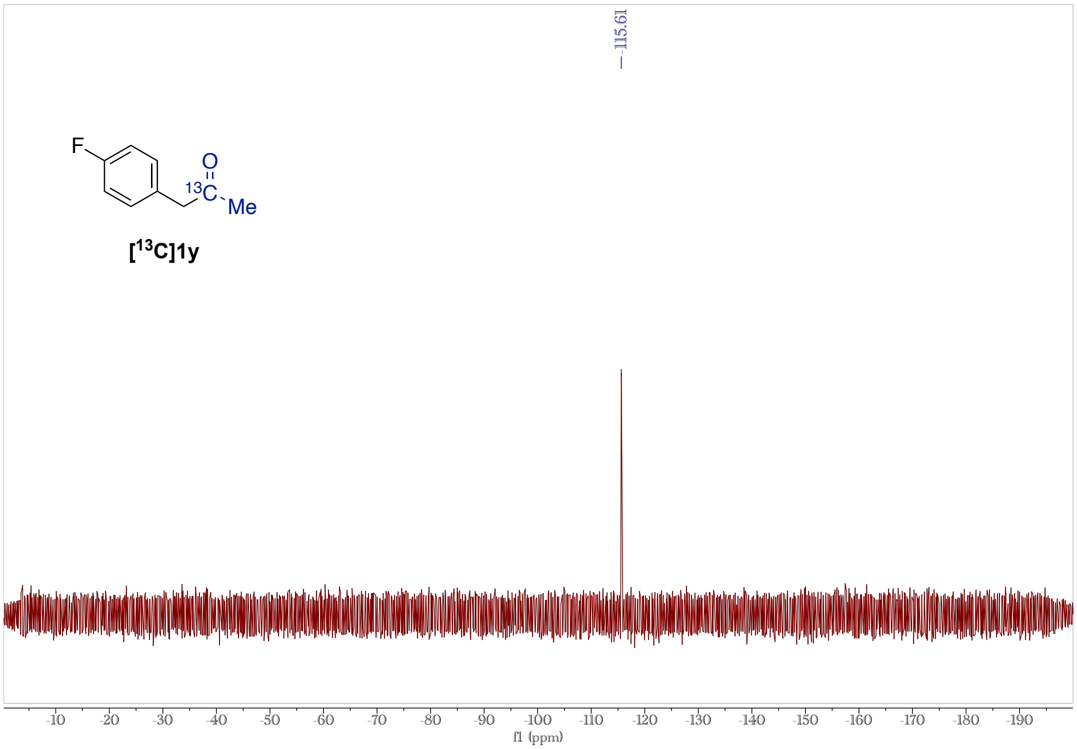

^{1}H NMR of **[13C]1z**

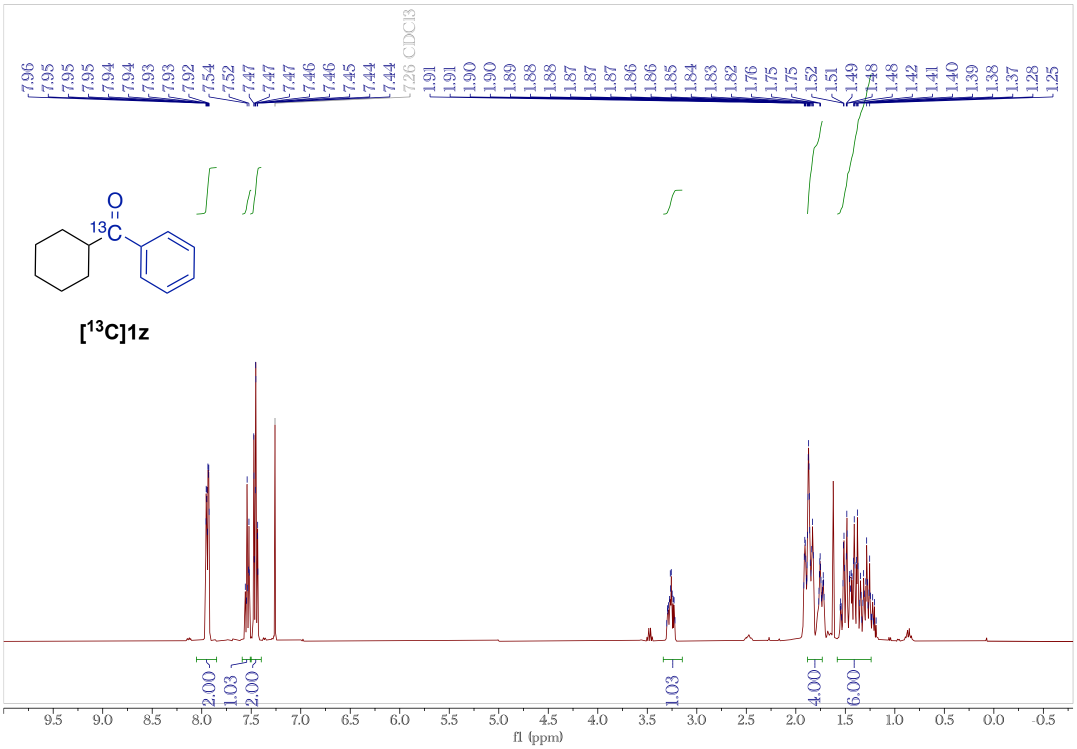

^{13}C NMR of **[13C]1z**

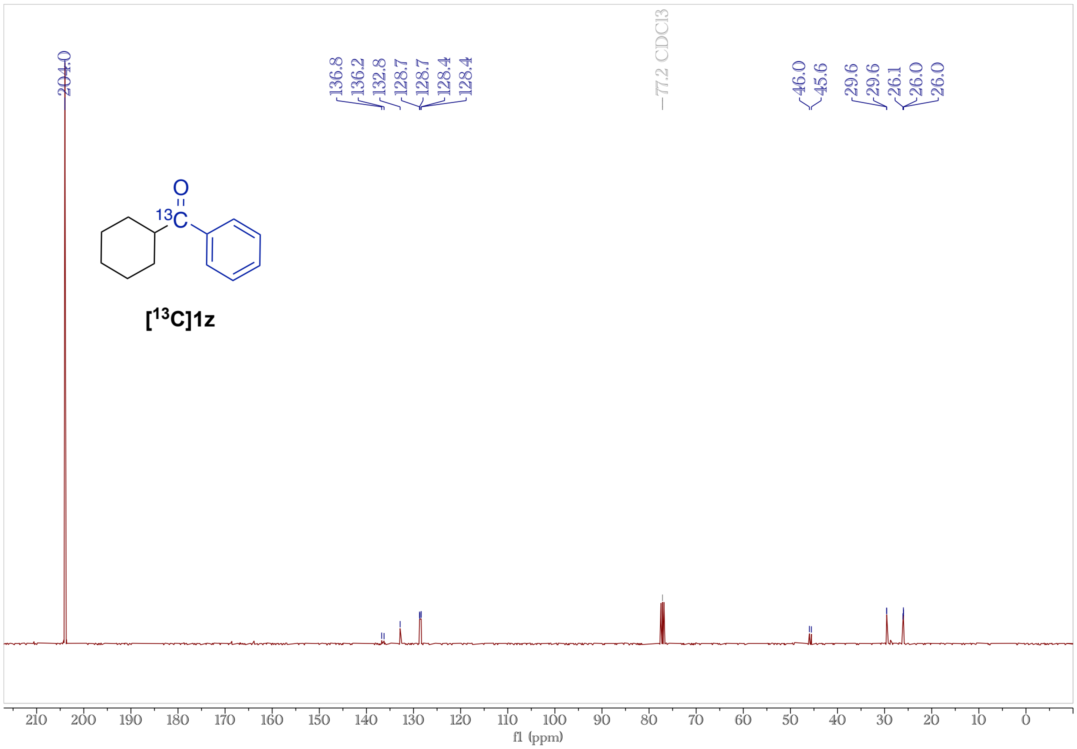

^{1}H NMR of **[13C]1za**

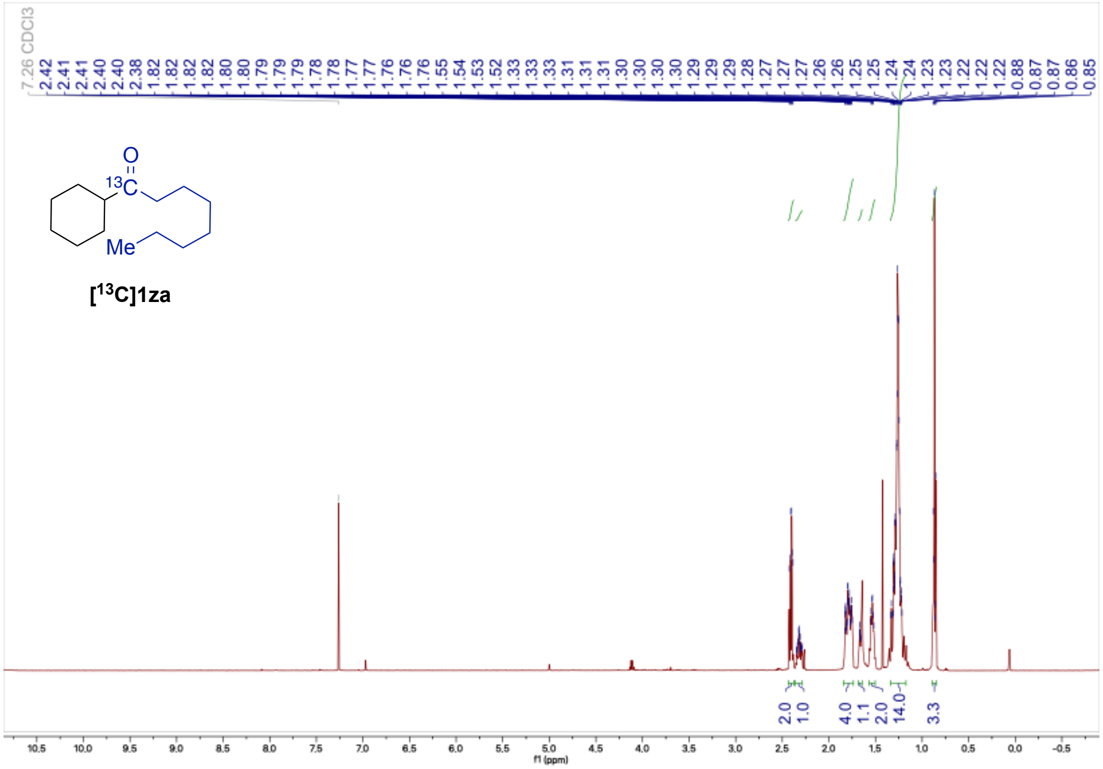

^{13}C NMR of **[13C]1za**

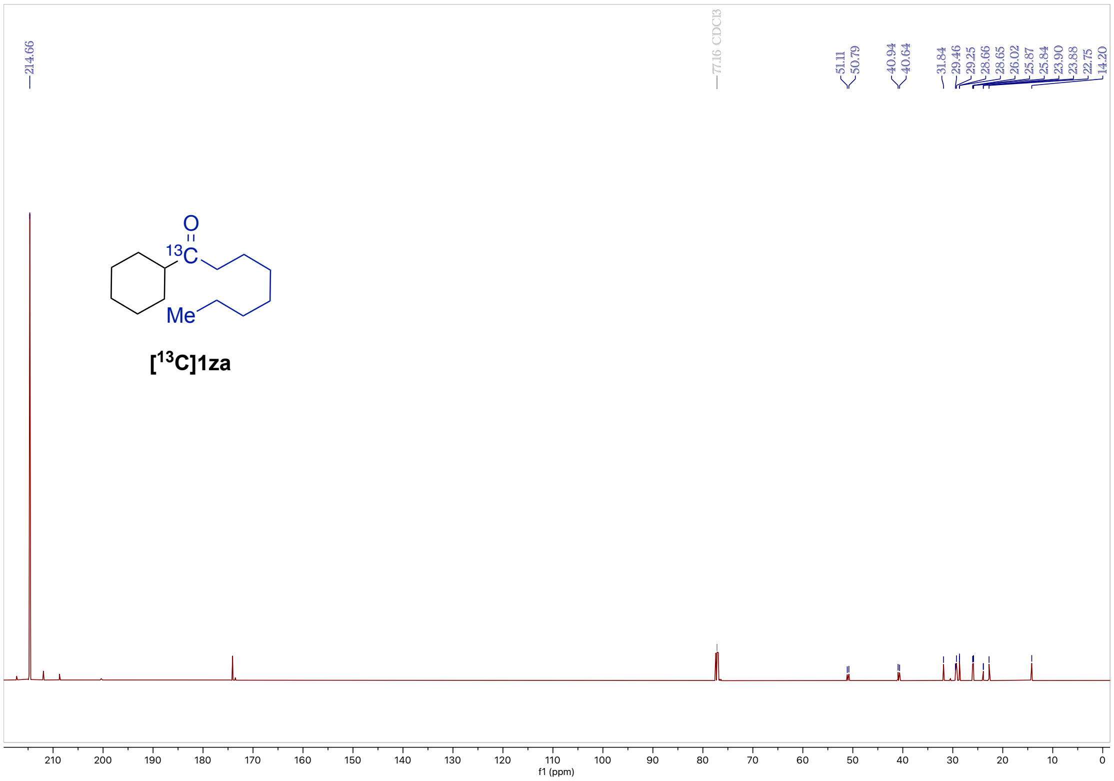

^{1}H NMR of **[13C]1zb**

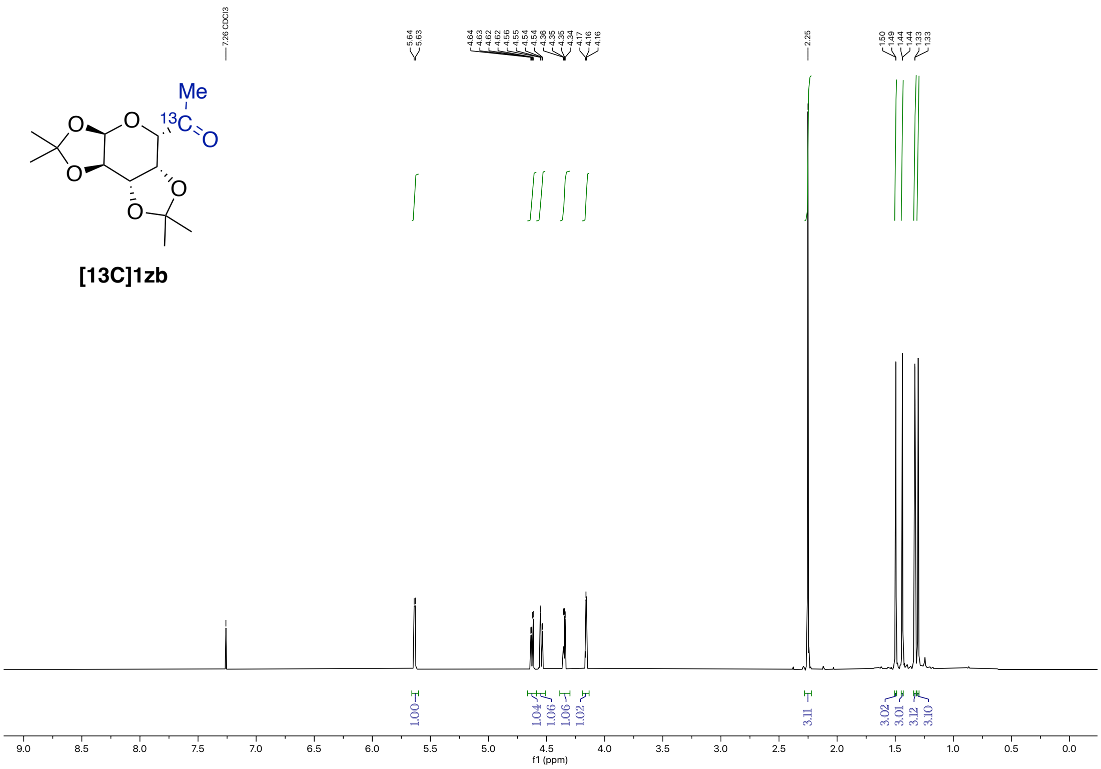

^{13}C NMR of **[13C]1zb**

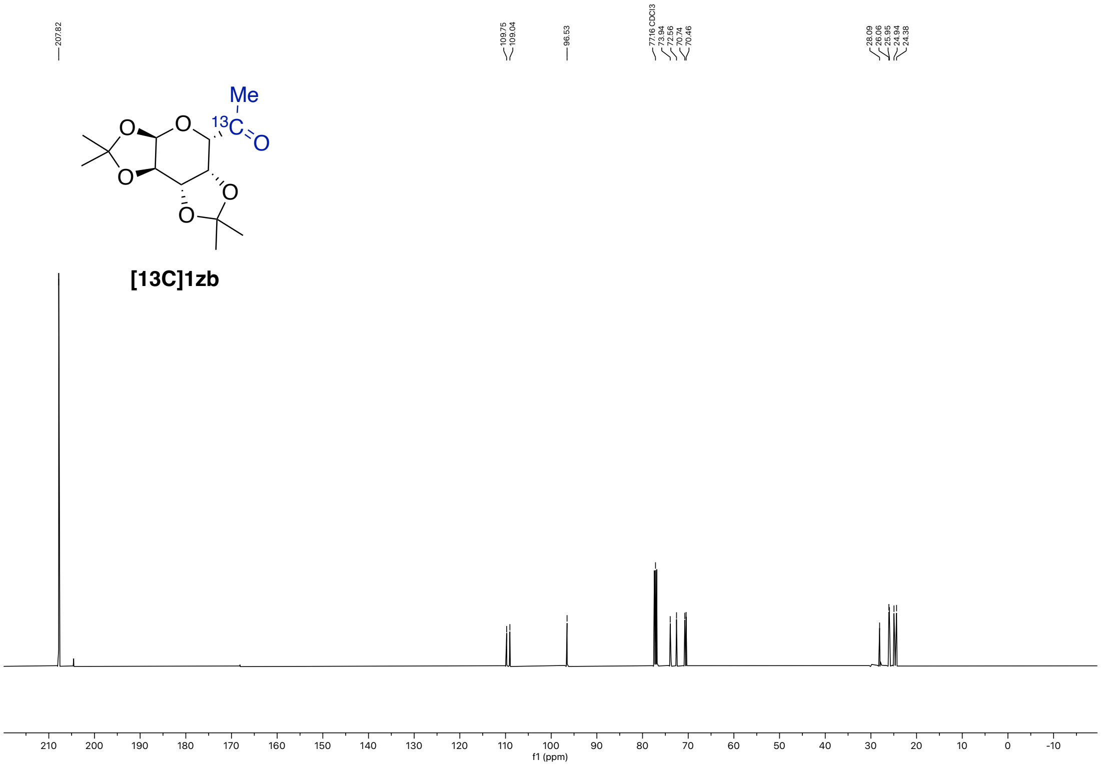

^{1}H NMR of **[13C]1zc**

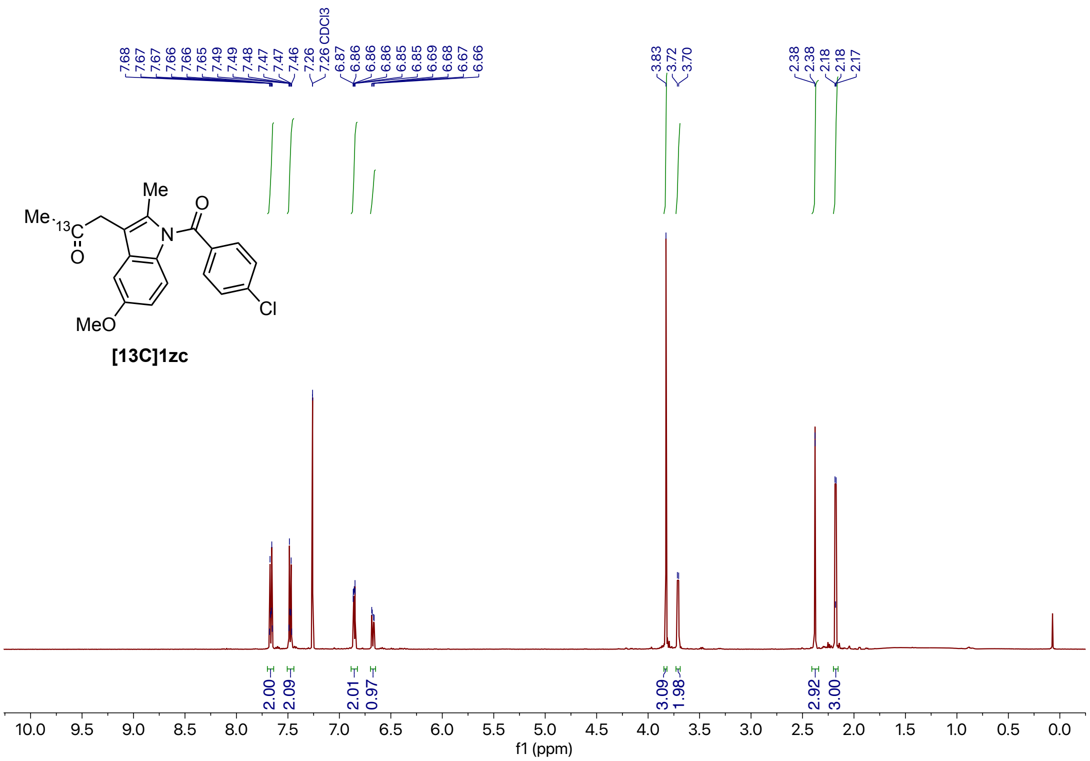

^{13}C NMR of **[13C]1zc**

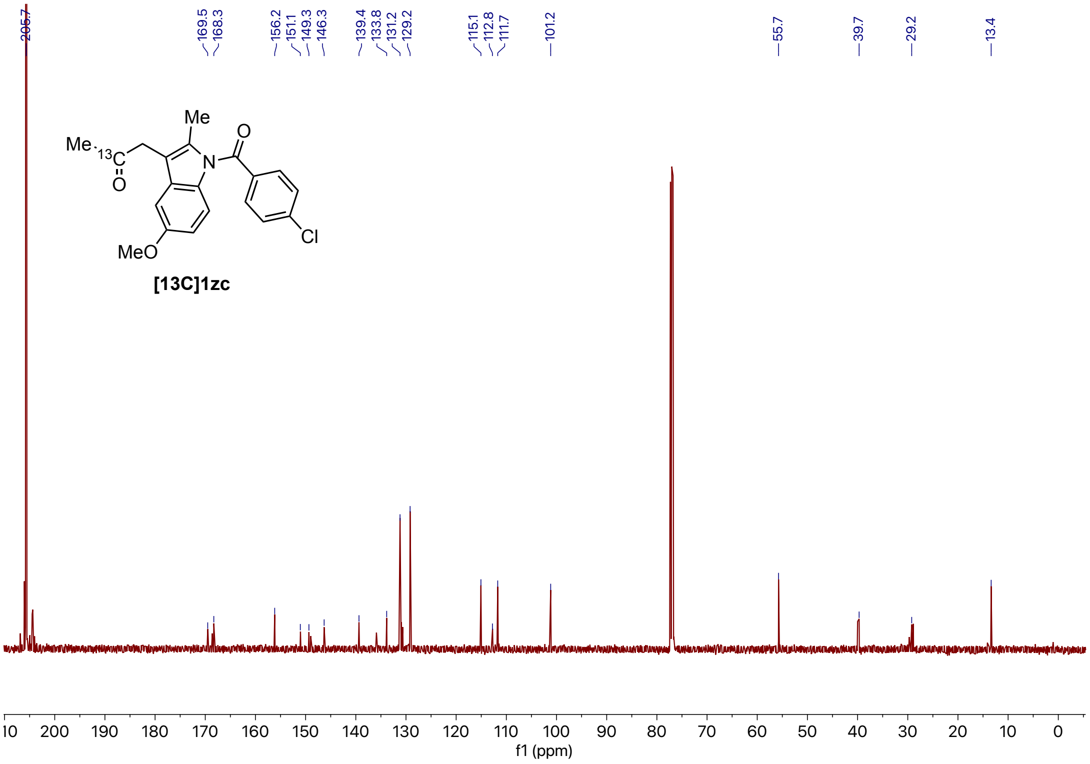

^{1}H NMR of **[13C]1zd**

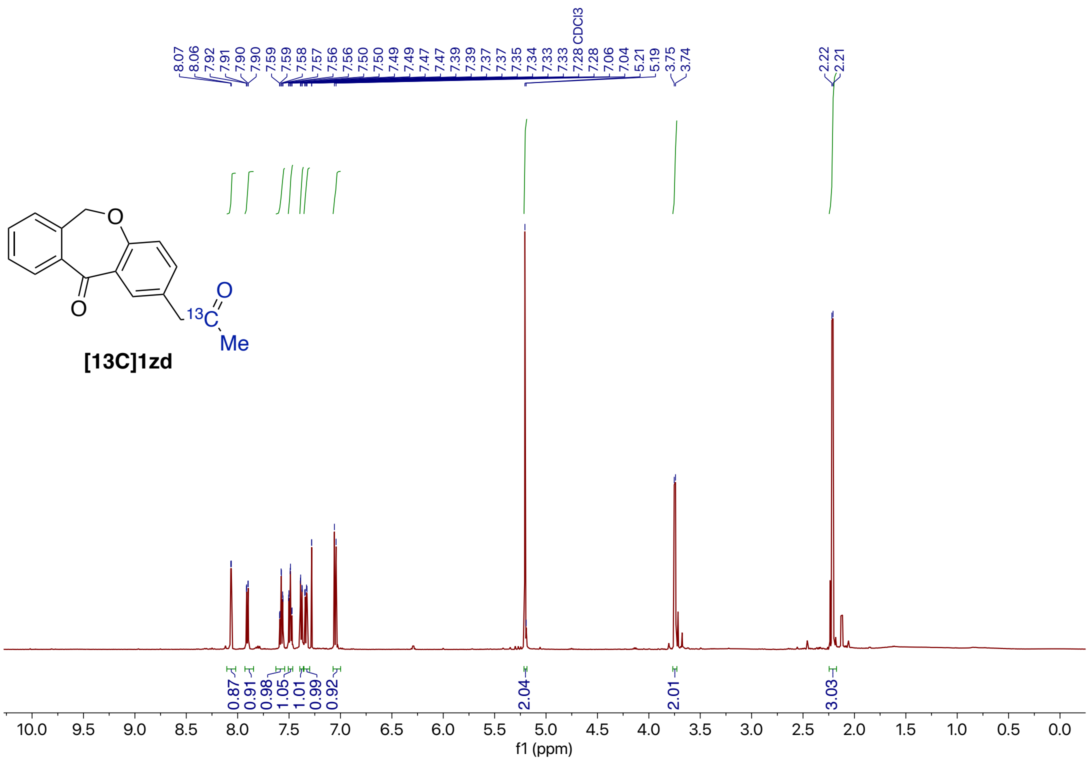

^{13}C NMR of **[13C]1zd**

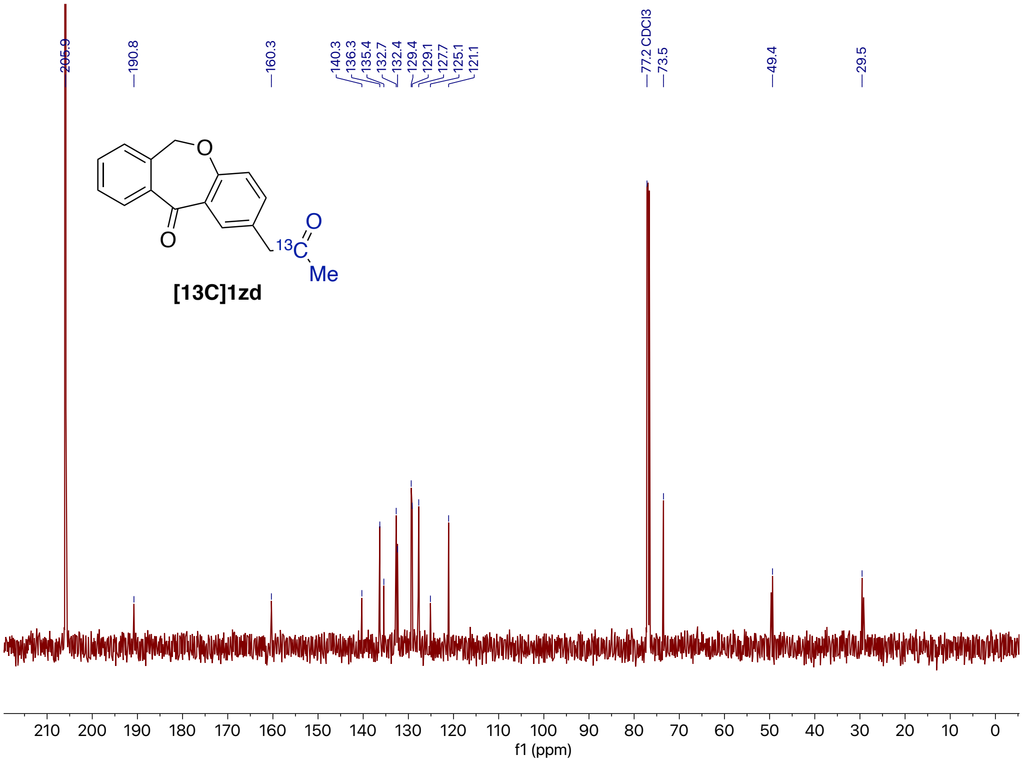

^{1}H NMR of **[13C]1ze**

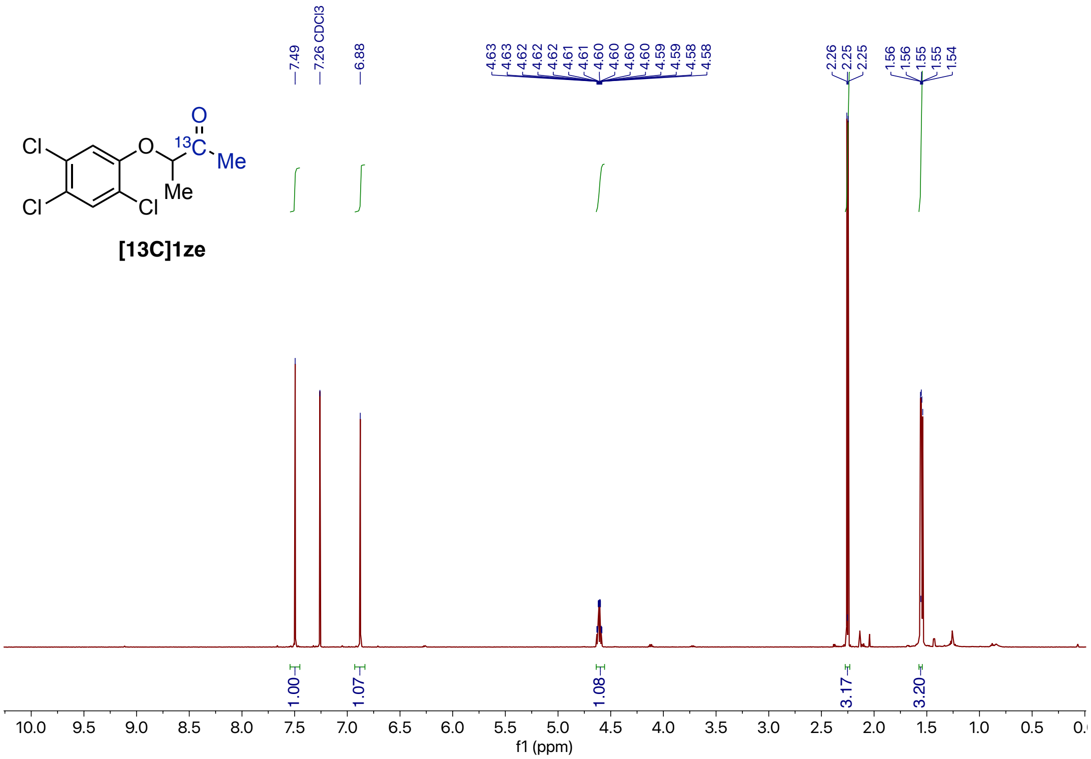

^{13}C NMR of **[13C]1ze**

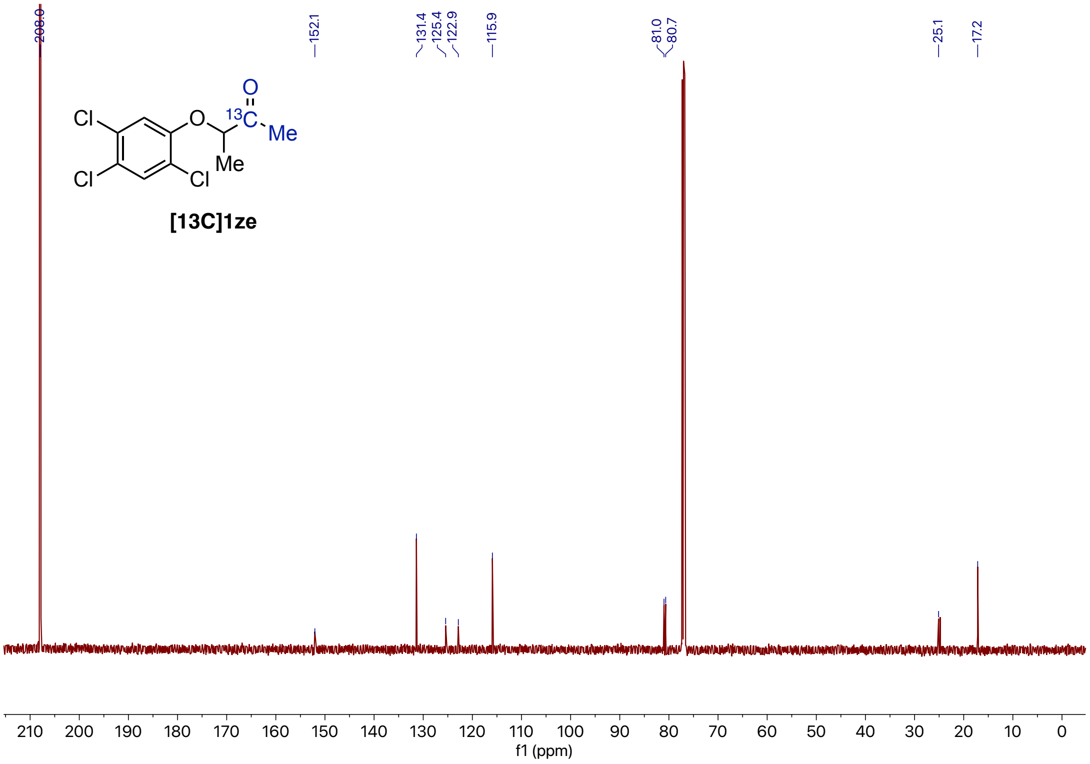

^{1}H NMR of **Ni-I**

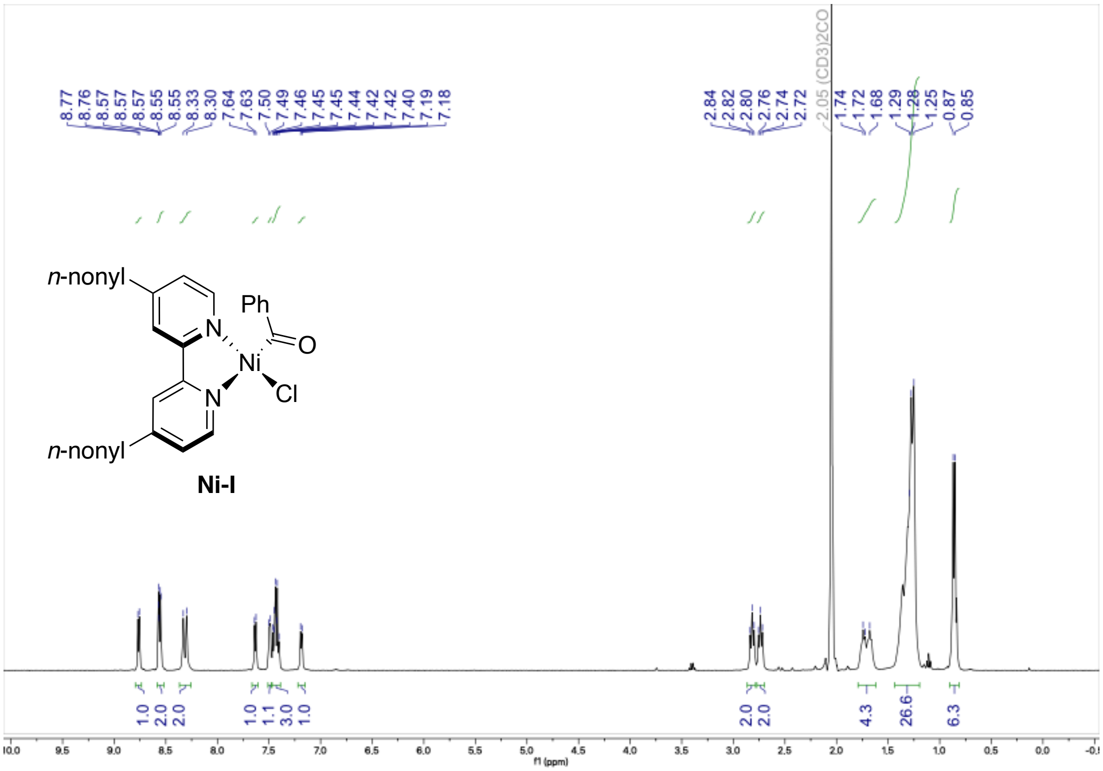

^{13}C NMR of **Ni-I**

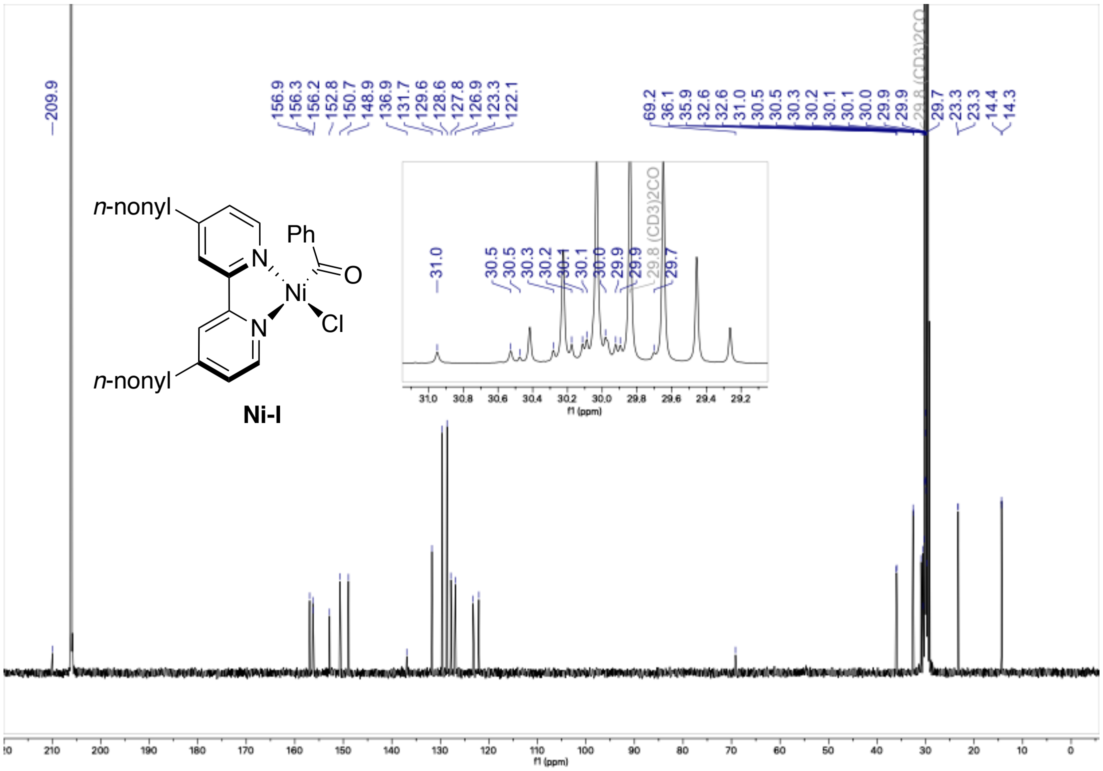

10. References

1. Masson, T. M.; Zondag, S. D.; Schuurmans, J. H.; Noël, T. *React. Chem. Eng*. **2024**, *9*, 2218-2225.

2. Lv, X. Y.; Abrams, R.; & Martin, R. *Angew. Chem. Int. Ed.* **2023**, *62*, e202217386.

3. Wang, T.; Zhang, Z.; Gao, F.; Yan, X. *Org. Lett.* **2024**, *26*, 6915−6920.

4. Cong, F.; Mega, R. S.; Chen, J.; Day, C. S.; Martin, R. *Angew. Chem. Int. Ed.* **2022**, *62*, e202214633.

5. Lv, X. Y.; Abrams, R.; Martin, R. *Nat. Commun.* **2022**, *13*, 2394-2402.

6. Yang, J.; Li, Z.; Huang, Z.; Zhu J. *Chem Eur J.* **2024**, *30*, e202402475.

7. Li, Q.-Z.; He, M.-H.; Rong Zeng, R.; Lei, Y.-Y.; Yu, Z.-Y.; Jiang, M.; Zhang, X.; Li, J.-L. *J. Am. Chem. Soc.* **2024**, *146*, 22829-22839.

8. Kinney, R.G.; Zgheib, J.; Lagueux-Tremblay, P.-L.; Zhou, C.; Yang, H.; Li, J.; Gauthier Jr, D. R.; B. A. *Nature Chem.* **2024**, *16*, 556–563.

9. Bryden, M. A.; Zysman-Colman, E. Chem. Soc. Rev. 2021, 50, 7587-7680.

10. Koo, Y.; Hong, S. *Chem. Sci.* **2024**, *15*, 7707-7713.

11. Gaspa, S.; Porcheddu, A.; De Luca, L. *Org. Lett.* **2015**, *17,* 3666–3669.

12. Pal, M.; Bearne, S. L. *Org. Biomol. Chem.* **2014**, *12*, 9760–9763.

13. Sun, Z.; Kumagai, N.; Shibasaki,N. *Org. Lett.* **2019**, *19*, 3727–3730.
